# A revision of the shore-fly genus *Hydrochasma* Hendel (Diptera, Ephydridae)

**DOI:** 10.3897/zookeys.363.6482

**Published:** 2013-12-16

**Authors:** Wayne N. Mathis, Tadeusz Zatwarnicki

**Affiliations:** 1 Department of Entomology, NHB 169, PO Box 37012; Smithsonian Institution, Washington, D.C. 20013-7012, USA; 2 Department of Biosystematics, Opole University, ul. Oleska 22, 45-052 Opole, Poland

**Keywords:** Diptera, Ephydridae, *Hydrochasma* sp. n., New World

## Abstract

A revision of the shore-fly genus *Hydrochasma* Hendel. The species of the genus *Hydrochasma* Hendel are revised, including 27 new species (type locality in parenthesis): *H. andeum* (Ecuador. Guayas: Boliche (02°07.7'S, 79°35.5'W)), *H. annae* (United States. Utah. Grand: Swasey Beach (15.3 km N Green River; 39°07'N, 110°06.6'W; Green River; 1255 m)), *H. capsum* (Ecuador. Orellana: RíoTiputini (0°38.2'S, 76°8.9'W)), *H. castilloi* (Ecuador. Loja: Catamayo (03°59'S, 79°21'W)), *H. crenulum* (Peru. Cuzco: Paucartambo, Atalaya (Río Alto Madre de Dios; 12°53.3'S, 71°21.6'W; 600 m)), *H. denticum* (Ecuador. Orellana: Río Tiputini (0°38.2'S, 76°8.9'W)), *H. digitatum* (Peru. Madre de Dios: Diamante (Río Alto Madre de Dios; 12°19.9'S, 70°57.5'W; 400 m)), *H. distinctum* (Costa Rica. Limón: Parque Nacional Barbilla, Sector Casas Negras, (10°0.8'N, 83°28.1'W; 300 m)), *H. dolabrutum* (Dominican Republic. Barahona: Barahona (18°12'N, 71°5.3'W)), *H. edmistoni* (Dominican Republic. Azua: near Pueblo Viejo (18°24.8'N, 70°44.7'W)), *H. falcatum* (Peru. Madre de Dios: Río Manu, Erika (near Salvación; 12°50.7'S, 71°23.3'W; 550 m)), *H. glochium* (Dominican Republic. Peravia: San José Ocoa (10 km NE; 18°35'N, 70°25.6'W)), *H. kaieteur* (Guyana. Kaieteur Falls (05°10.5'N, 59°26.9'W)), *H. lineatum* (Trinidad and Tobago. Trinidad. St. George: Filette (1 km SE; 10°47'N, 61°21'W)), *H. miguelito* (Honduras. Cortés: San Pedro Sula (8 km S; 15°25.7'N, 88°01.4'W)), *H. octogonum* (Ecuador. Manabí: Pichincha (01°02.7'S, 79°49.2'W)), *H. parallelum* (Trinidad and Tobago. Trinidad. St. Andrew: Lower Manzanilla (16 km S; 10°22'N, 61°01'W)), *H. peniculum* (Dominican Republic. Pedernales: Pedernales (18°01.8'N, 71°44.7'W)), *H. rictum* (Honduras. Cortés: San Pedro Sula (8 km S; 15°25.7'N, 88°01.4'W)), *H. robustum* (Brazil. São Paulo. Ubatuba, Praia Puruba (23°21'S, 44°55.6'W; beach)), *H. sagittarium* (Trinidad and Tobago. Tobago: St. John: Parlatuvier (creek; 11°17.9'N, 60°35'W)), *H. simplicum* (Costa Rica. Limón: Parque Nacional Barbilla, Sector Casas Negras, (10°01.2'N, 83°26.2'W; 300 m)), *H. sinuatum* (Belize. Stann Creek: Mullins Creek (17 km N Dangriga; 17°06.2'N, 88°17.8'W)), *H. spinosum* (Costa Rica. Limón: Westfalia (4 km S; 09°54.5'N, 82°59'W; beach)), *H. urnulum* (Dominican Republic. Puerto Plata: Río Camu (14 km E Puerto Plata; 19°41.9'N, 70°37.5'W)), *H. viridum* (Guyana. Karanambo, Rupununi River (ox bow; 03°45.1'N, 59°18.6'W)), *H. williamsae* (Belize. Stann Creek: Mullins River (17 km N Dangriga; 17°06.2'N, 88°17.8'W)). All known species are described with an emphasis on structures of the male terminalia, which are fully illustrated. Detailed locality data and distribution maps for all species are provided. A lectotype is designated for *Discocerina incisum* Coquillett and *Hydrochasma zernyi* Hendel. For perspective and to facilitate genus-group and species-group recognition, the tribe Discocerinini is diagnosed and a key to included genera in the New World is provided.

## Introduction

*Hydrochasma* Hendel is one of three genera of the shore-fly tribe Discocerinini (subfamily Gymnomyzinae) that occurs exclusively in the New World. The other two Discocerine genera are *Pectinifer* Cresson, which is known only by its genotype, *Pectinifer aeneus* Cresson, and *Facitrichophora* Mathis & Zatwarnicki, which was recently described and includes four Neotropical species (Mathis and Zatwarnicki 2012a). Within Discocerinini, *Pectinifer* is placed in the *Diclasiopa* group of genera, and *Hydrochasma*, like *Facitrichophora*, is classified in the *Discocerina* group of genera (Zatwarnicki and Mathis 2001).

Among all New World genera of Discocerinini, *Hydrochasma* is perhaps the least well known, as indicated by the dramatic increase in recognized species reported in this revision. Until this revision, *Hydrochasma* included just seven species (Mathis and Zatwarnicki 1995 and updates), and in this revision, we more than quadruple that number by adding 27 previously undescribed species. These additions bring the total number of congeners to 34.

We are revising the biologically diverse *Hydrochasma* to account taxonomically for numerous undescribed species that we have collected and recently discovered. Much of this rather dramatic increase has resulted from a greatly improved sampling of the New World fauna, especially the Neotropical fauna where the first author has focused his fieldwork for nearly four decades. Also contributing to this increase is our use of characters from structures of the male terminalia, which has revealed species complexes for what had been treated in some cases as a single, widespread species. The species of the *incisum* or *leucoproctum* groups are examples of this discovery process.

Although specimens are not generally uncommon in nature, most collections, with few exceptions, have relatively few specimens, and the genus is not generally well known except to specialists. Only a few authors have reported on any included species, and aside from Cresson (1938, 1942, 1946), there are no comprehensive treatments available. The literature on included taxa is not extensive and mostly comprises alpha-level taxonomic treatments, sometimes as isolated species descriptions. In view of recent field work and discovery of numerous undescribed species, the available literature is also inadequate for dealing with species that are found only in the New World. We also know virtually nothing about the ecology and natural history of any of the included species except for brief habitat characterizations where adults have been found (Deonier 1965). With clarification of species–how they can be recognized and where they occur–we hope that additional research on immature stages and other aspects of their natural history and ecology will be fostered and facilitated.

Hendel (1936) first described *Hydrochasma* with *Hydrochasma zernyi* Hendel (= *Discocerina faciale* Williston) as its genotype by monotypy. Cresson (1938, 1946 Neotropical, 1942 Nearctic) published brief synopses of New World species, and in the first synopsis, he included the description of *Hydrochasma capax* (= *Hydrochasma faciale*) and *Hydrochasma patens*. Before Hendel (1936), however, the first four species now placed in *Hydrochasma* were all initially described in the genus *Discocerina*: *Hydrochasma leucoproctum* (Loew 1861), *Hydrochasma faciale* (Williston 1896), *Hydrochasma incisum* (Coquillett 1902), and *Hydrochasma poecilogastrum* (Hendel 1930 = *Hydrochasma incisum*). Aside from these isolated species descriptions, recent catalog entries (Wirth 1965 Nearctic, 1968 Neotropical, Mathis and Zatwarnicki 1995 world) and faunistic listings (Wirth and Stone 1956 California, Cole 1969 Western North America), the species of *Hydrochasma* have essentially not been treated. We (Mathis and Zatwarnicki 2010) described three species, *Hydrochasma aquia*, *Hydrochasma avanae*, and *Hydrochasma garvinorum*, as part of a faunistic treatment of the mid-Atlantic shore-fly fauna of the United States.

Even though there is a paucity of taxonomic information on species of *Hydrochasma*, we know even less about the natural history of any included species. Deonier (1964, 1965) recorded his observations that specimens of *Hydrochasma leucoproctum* (as *Discocerina leucoprocta*) were a common species on sandy shores in Iowa, but only occasional on limnic wrack, and were rare in sedge meadow and marsh reed habitats.

## Methods and materials

The descriptive terminology, with the exceptions noted in Mathis (1986) and Mathis and Zatwarnicki (1990a), follows McAlpine (1981). Because specimens are small, usually less than 2.60 mm in length, study and illustration of the male terminalia required use of a compound microscope. We have followed the terminology for most structures of the male terminalia that other workers in Ephydridae have used (references in Mathis 1986; Mathis and Zatwarnicki 1990a, 1990b), such as surstylus. Zatwarnicki (1996) suggested that the pre- and postsurstylus correspond with the pre- and postgonostylus and that the subepandrial sclerite is the same as the medandrium. The terminology for structures of the male terminalia is provided directly on Figs 2–5. We use the term basal flagellomere for the large antennomere beyond the pedicel. We prefer this term over “first flagellomere” as there may be more than one flagellomere involved, and basal does not imply a number or numbers. We likewise do not use “postpedicel” (Stuckenberg 1999) for this antennomere because at least the multisegmented arista is beyond the pedicel in addition to the large antennomere, and postpedicel is thus ambiguous and lacking in precision.

Dissections of male terminalia were performed following Clausen and Cook (1971) and Grimaldi (1987). Abdomens were removed with microforceps and macerated in a sodium hydroxide solution. Cleared genitalia were then transferred to glycerin for observation, description, and illustration. The dissected abdomen was placed in a plastic microvial filled with glycerin and attached to the pin supporting the remainder of the insect from which it was removed. These structures for species of *Hydrochasma* are minute, and for accurate determinations using them, we often had to use a compound microscope to see them clearly.

The species descriptions are composite and not based solely on holotypes. Head and two venational ratios used in the descriptions are based on three specimens (largest, smallest, and one other): gena-to-eye ratio – genal height (immediately below maximum eye height)/eye height; costal vein ratio – the straight line distance between the apices of R_2+3_ and R_4+5_/distance between the apices of R_1_ and R_2+3_; M vein ratio – the straight line distance along vein M between crossveins dm-cu and r-m/distance apicad of dm-cu.

Distribution maps were made using ESRI ArcView© GIS 3.2. Longitude and latitude coordinates were obtained for the locality where each specimen was collected and entered into a Microsoft Excel© spreadsheet. If unavailable directly from specimen labels, longitude and latitude were estimated using gazetteers and maps to determine the geographical coordinates. Localities of specimens were plotted on a world land projection, presented within ESRI ArcView layouts and exported as encapsulated postscript (EPS) files.

Many specimens examined for this study are in the National Museum of Natural History, Smithsonian Institution, Washington, D.C. (USNM). We also borrowed and studied numerous specimens, especially primary types from the following museums:

ABSF Archbold Biological Station, Lake Placid, Florida, United States (Mark A. Deyrup)

AMNH American Museum of Natural History, New York, New York (David A. Grimaldi and Julian Stark)

ANSP Academy of Natural Sciences of Philadelphia, Pennsylvania (Jon K. Gelhaus and Jason D. Weintraub)

BMNH The Natural History Museum (formerly the British Museum (Natural History)), London, England, United Kingdom (John E. Chainey and Kim Goodger)

CAS California Academy of Sciences, San Francisco, California (Norman D. Penny)

CMP Carnegie Museum of Natural History, Pittsburgh, Pennsylvania (Chen Young)

INBio Instituto Nacional de Biodiversidad, Santo Domingo, Heredia, Costa Rica (Manuel A. Zumbado)

MCZ Museum of Comparative Zoology, Harvard University, Cambridge, Massachusetts (Philip D. Perkins)

NMW Naturhistorisches Museum, Wien, Austria (Peter Sehnal)

SMN Staatliches Museum für Naturkunde in Stuttgart, Stuttgart, Germany (Hans-Peter Tschorsnig)

WSU Maurice T. James Collection, Department of Entomology, Washington State University, Pullman, Washington (Richard S. Zack)

## Systematics

### Tribe Discocerinini

Cresson

Discocerinini (as Discocerini) Cresson 1925: 228. Type genus: *Discocerina*Macquart 1835. Mathis and Zuyin 1989: 435 [description, key to Asian genera]. Mathis and Zatwarnicki 1995: 163–186 [world catalog]. Zatwarnicki and Mathis 2001: 5–51 [revised classification].

#### Diagnosis.

A tribe of the subfamily Gymnomyzinae that is distinguished by the following combination of characters: Small to medium-sized shore flies, body length 1.15–3.50 mm; usually invested with considerable microtomentum, especially frons and mesonotum.

*Head*: Frontal vitta (or ocellar triangle) mostly bare of setulae, not conspicuously setulose; pseudopostocellar setae well developed, length greater than distance between either posterior ocellus and anterior ocellus, generally with proclinate orientation and slightly divergent; ocellar seta inserted anterior to lateral alignment of anterior ocellus, sometimes only slightly so; reclinate fronto-orbital seta inserted in front of proclinate fronto-orbital (if 2 proclinate fronto-orbital setae present, reclinate seta inserted in front of the larger proclinate seta); proclinate fronto-orbital seta subequal to length of reclinate seta. Pedicel bearing a large seta anterodorsally; arista with 5-7 dorsally branching rays evenly along aristal length. Compound eye bearing numerous, interfacetal microsetulae. Face generally smooth, not conspicuously pitted or rugose, in lateral view shallowly carinate between antennal bases and/or very shallowly conically produced, convex. Gena generally short (secondarily high in some species), bearing setulae (including midportion) and 1 large seta, its posterior (postgenal) margin rounded, not sharp. Oral opening and clypeus narrow; mouthparts generally dark colored; clypeus generally microtomentose, similar to microtomentum of face.

*Thorax*: Mesonotum generally microtomentose, usually densely so; supra-alar seta usually evident although sometimes reduced; acrostichal setulae arranged in about 8 irregular rows; prescutellar acrostichal setae approximate and inserted behind level of posteromost dorsocentral setae; scutellum usually moderately densely setulose, bearing more than 20 setulae, these evenly scattered; both anterior and posterior notopleural setae inserted at about the same level from notopleural/anepisternal suture; anepisternum with 2 equal setae along posterior margin. Wing with vein R_2+3_ long, extended nearly to level of apex of vein R_4+5_. Foreleg normally developed, not raptorial with greatly enlarged femur.

*Abdomen*: Five tergites visible, usually not covered with microtomentum. Male terminalia: Structures symmetrical; cerci paired, hemispherical, setose, bearing sides of rectum, sometimes fused with posteroventral margin of epandrium; epandrium U-shaped, encircling cerci, anterior margin rounded, in lateral view with setae mainly on dorsum and along anteroventral margin; presurstylus lacking or fused indistinguishably with epandrium; posterolateral arms of epandrium attached with ventral apex of gonites, middle of posterior margin a base for phallapodeme; phallapodeme situated under aedeagus, associated with hypandrium and with ventral part of base of aedeagus, ventral margin with lobate appendix providing attachment for genital muscles that move aedeagus, sometimes fused with base of aedeagus; gonites paired, connecting sides of base of aedeagus and laterodorsal margin of epandrium, bearing 1 or some setulae; subepandrial plate reduced; aedeagus tubular, tapered anteriorly; ejaculatory apodeme usually lacking, if present as a spatula-shaped structure against background of ductus ejaculatorius.

#### Discussion.

Starting with Cresson (1925), who first described Discocerinini, and including all students of the family until Mathis and Zuyin (1989), the diagnoses, descriptions, and catalogs of this tribe included some taxa that are not closely related phylogenetically, rendering the tribe polyphyletic. Mathis and Zuyin (1989) recharacterized Discocerinini using synapomorphies and resulting in a monophyletic tribe into which Mathis and Zatwarnicki (1995) included eight genera and 143 species in their world catalog. Zatwarnicki and Mathis (2001) then added two additional genera, *Galaterina* and *Orasiopa*, and altered the status of some subgenera in their phylogenetic study of the tribe. Finally, Mathis and Zatwarnicki (2012a) recently described *Facitrichophora*.

#### Phylogenetic relationships.

On a world basis, Zatwarnicki and Mathis (2001) proposed a phylogenetic hypothesis for the higher-level lineages within the tribe Discocerinini, dividing the included genera into three sublineages or groups: the *Gymnoclasiopa*, *Diclasiopa*, and *Discocerina* groups. *Hydrochasma* is included in the *Discocerina* group and is distinguished from other New World genera in the key and generic diagnosis that follow (characters being discussed are synapomorphies unless otherwise specified). Other genera in addition to *Hydrochasma* that are included in the *Discocerina* sublineage are: *Discocerina* Macquart, *Facitrichophora* Mathis & Zatwarnicki, *Galaterina* Zatwarnicki & Mathis, *Lamproclasiopa* Hendel, *Orasiopa* Zatwarnicki & Mathis, and *Polytrichophora* Cresson. *Hydrochasma*, along with other genera of the *Discocerina* sublineage, form a monophyletic lineage within the Discocerinini that is corroborated by two synapomorphies. The first is the setulose notopleuron. In other genera of the subfamily Gymnomyzinae, including other genera in the tribe Discocerinini, the notopleuron is bare except for larger anterior and posterior setae that are inserted near the ventral margin. In taxa of the *Discocerina* group, however, the notopleuron bears a few additional setulae that are usually inserted slightly dorsad and toward the anterior portion of the notopleuron, usually around or just dorsad of the anterior notopleural seta. The second synapomorphy confirming the monophyly of the *Discocerina* group is the shape of the gonite, which is narrowly bar-like, often nearly parallel sided. In other genera of Discocerinini outside of the *Discocerina* group, the gonite is elongate, variously swollen medially, and tapered toward one or both apices. Within the *Discocerina* group, an elongated male terminalia (hypopygium), at least 2.5× longer than wide (the plesiomorphic condition is hypopygium of moderate length), occurs almost exclusively in four genera: *Facitrichophora*, *Galaterina*, *Hydrochasma*, and *Polytrichophora*. The genera *Facitrichophora*, *Hydrochasma* and *Polytrichophora* are characterized by a deeply incised posterior margin of hypandrium (the plesiomorphic condition is for the hypandrium to have a slightly to moderately concave posterior margin). Within the *Discocerina* group, *Hydrochasma* is distinguished by the following synapomorphies: (1) three facial setae (by convergence, some species in related genera also have three setae); (2) parafacial setulose (a species group within *Discocerina* also has a setulose parafacial, apparently by convergence); (3) gena high (the buccata species group within *Discocerina* also has a high gena); (4) cerci usually fused ventrally or ventrolaterally with epandrium (the cerci are similarly fused in many species of *Facitrichophora* and *Polytrichophora*); (5) epandrium with dorsal portion or arch above the cerci weakened, thin, or absent; (6) posterior margin of hypandrium deeply incised.

### Annotated key to New World genera and subgenera of Discocerinini

**Table:** 

1	Notopleuron bare of setulae	2
–	Notopleuron setulose in addition to 2 large setae	6
2	Forefemur slightly enlarged, bearing distinct row of stout, short setae along apical half of posteroventral surface	*Pectinifer* Cresson
	[Monotypic; *Pectinifer aeneus* (Cresson), New World tropics]	
–	Forefemur normally developed, lacking row of short, stout setae along posteroventral surface, any such setae being strictly ventral in position	3
3	Postsutural supra-alar seta strong, distinct, longer than posterior notopleural seta. Face with dorsoclinate seta at lower lateral extremity	*Diclasiopa* Hendel
	[4 species worldwide; a single New World species, *Diclasiopa lacteipennis* (Loew)]	
–	Postsutural supra-alar seta very short or absent, if distinguishable distinctly shorter than posterior notopleural seta. Face without dorsoclinate seta at lower lateral extremity	4
4	Hindtibia with a preapical, ventral, spur-like seta; facial series comprising 2–3 large setae, dorsal seta inserted slightly medially from other setae and arising from distinct, shiny papilla, with a small, slightly dorsoclinate seta laterad of dorsal seta; generally microtomentose, cinereous species, appearing dull	*Hecamedoides* Hendel
	[26 species worldwide; two New World species, *Hecamedoides unispinosus* (Collin) and *Hecamedoides lattini* Mathis & Zatwarnicki, Old World species presently being revised (Zatwarnicki, in preparation)]	
–	Hindtibia lacking a preapical, ventral spur-like seta; facial series comprised of 2 large setae, dorsal seta not arising from a shiny papilla and lacking a smaller seta laterad of dorsal seta; mostly bare to sparsely microtomentose, shiny to subshiny species	5
5	Face rather flattened, antennal grooves not always sharply defined ventrally; facial series of setae inserted very close to parafacial, dorsalmost seta not appreciably more removed medially than ventral seta	*Gymnoclasiopa* Hendel
	[26 species worldwide; 10 New World species (Mathis and Zatwarnicki 2012b)]	
–	Face rather prominent at level of dorsal facial setae, sometimes transversely carinate: antennal grooves generally sharply defined ventrally	*Ditrichophora* Cresson
	[40 species worldwide; 11 Nearctic species]	
6	Face with 2 or more conspicuous rows of setae/setulae on each side, paralleling facial suture setal row medial, row(s) of setulae between setal row and parafacial	7
–	Face with a single row of setae laterally	8
7	Face with secondary series of dorsolaterally inclined setae laterad to primary series	*Polytrichophora* Cresson
	[31 species worldwide; 19 New World species (Mathis and Zatwarnicki 2012a)]	
–	Face with setae and setulae of rows inclinate or ventroinclinate	*Facitrichophora* Mathis & Zatwarnicki
	[4 New World species (Mathis and Zatwarnicki 2012a)] [31 species worldwide; 19 New World species (Mathis and Zatwarnicki 2012a)]	
8	Parafacial bearing setulae	*Discocerina* Macquart
	[20 species worldwide; 12 New World species]	
–	Parafacial lacking setulae	9
9	Gena and lower part of parafacial broad; lateral margin of abdomen usually with gray to whitish microtomentose areas, these usually wedge shaped	*Hydrochasma* Hendel
	[34 New World species; revised herein]	
–	Gena and parafacial rather narrow; abdomen lacking wedge-shaped, light-colored areas laterally	10
10	Postsutural supra-alar and prescutellar acrostichal setae greatly reduced or lacking; facial series of setae 2, these well separated, distance between them subequal to length of basal flagellomere; parafacial very narrow at anteroventral margin of eye	*Lamproclasiopa* Hendel
	[11 species worldwide; 10 New World species]	
–	Postsutural supra-alar and prescutellar acrostichal setae present; facial series of setae 3–4, distance between setae conspicuously less than length of basal flagellomere, if 2 facial setae present, see first character; parafacial evenly wide throughout length	*Orasiopa* Zatwarnicki & Mathis
	[15 species worldwide; one New World species (adventive), *Orasiopa mera* (Cresson)]	

### Genus Hydrochasma

Hendel

http://species-id.net/wiki/Hydrochasma

Hydrochasma Hendel, 1936: 101. Type species: *Hydrochasma zernyi* Hendel, 1936 (= *Discocerina faciale* Williston, 1896), monotypy. Cresson 1938: 25–28 [review, discussion]; 1942: 113 [review of Nearctic species]; 1946: 141–142, 150 [review of Neotropical species]. Wirth 1965: 738–739 [Nearctic catalog]; 1968: 8 [Neotropical catalog]. Mathis and Zatwarnicki 1995: 182–183 [world catalog].

#### Diagnosis.

Small to moderately small shore flies, body length 1.50–3.10 mm; generally densely microtomentose, dull species. *Head* (Figs 18–20, 36–38, 49–51, 62–64, 124–126, 154–156, 172–174): Frons lacking anterior, proclinate, fronto-orbital seta. Face distinctly prominent at level of dorsal facial seta; antennal grooves generally sharply defined ventrally; face lacking secondary series of setae; facial setae 3, sometimes with a smaller 4th seta ventrally, setae generally decreasing in size from dorsum to venter, aligned vertically in a single series, dorsal setae not arising from shiny papilla, lacking an dorsoclinate seta at ventrolateral extremity; parafacial moderately wide to wide, expanded posteroventrally, bearing setulae; gena generally high, although variable. Eye generally oval with slight anteromedial expansion, moderately conspicuously microsetulose, bearing distinct interfacetal setulae. Maxillary palpus yellow apically. *Thorax*: Single presutural and postsutural supra-alar setae well developed; acrostichal setae present; notopleuron bearing several setulae in addition to 2 larger setae; anterior notopleural seta inserted conspicuously closer to posterior notopleural seta than to postpronotal seta. Wings transparent, shiny; costa bearing 4–6 long, dorsal setae between humeral and subcostal breaks. Hindtibia lacking or bearing a preapical, ventral, spur-like seta. *Abdomen*: Tergites variable, unicolorous or bicolorous, often with lighter colored areas laterally, sometimes as lateral wedges (Figs 12, 70–71, 142–143, 184–185). Male tergite 4 only slightly longer than tergite 3. Male terminalia: Epandrium generally elongate, dorsal portion above cerci weakly developed or usually not connected, with a dorsal gap; mid and posterior surface mostly covered with setae; cercus separate or fused ventrolaterally or ventrally with epandrium, in posterior view broadly lunate; gonite symmetrical, separate from hypandrium, bar-like, situated between base of aedeagus and posterior margin of hypandrium, lacking seta; aedeagus symmetrical, mostly tubular, sometimes very elongate, in ventral view elongate, often cigar-like, in lateral view slightly sinuous with rounded or expanded apex, rarely with dorsal projection; hypandrium in ventral view U- or V-shaped, sometimes narrow, with rounded or variously incised anterior margin, in lateral view almost flat or slightly arched; aedeagal apodeme separate from aedeagus, in lateral view elongate with variable ventral projection, usually small; ejaculatory apodeme absent.

#### Distribution.

Specimens of *Hydrochasma* have thus far been found only in the New World, occurring where temperate and tropical climates prevail.

#### Natural history.

Adults of *Hydrochasma* occur on bare, often exposed mud and sand, and many species abound in estuarine habitats, especially those along maritime coasts. Specimens are also common in inland alkaline and saline habitats. The immature stages are unknown.

#### Discussion.

*Hydrochasma* is usually readily recognized. For a few of the included species, however, determining generic assignment can be difficult because external characters are not always wholly concordant with interpretations of structures of the male terminalia (see species excluded near the end of this paper, p. 150).

#### Key to species groups of *Hydrochasma* Hendel

**Table:** 

1	Tergite 4 and sometimes 3 with wedge-shaped, silvery-gray areas laterally, these extended into darker, dorsal coloration (Figs 71, 142–143)	the *incisum* group
–	Tergites 3–4 lacking wedge-shaped, silvery gray areas laterally that extend into darker, dorsal coloration (Figs 12, 70, 184–185)	2
2	Hindtibia bearing a prominent, ventral, spur-like seta near apex (Fig. 11); head subglobose, oral opening comparatively large (Figs 18–20, 36–38, 49–51)	the *faciale* group
–	Hindtibia lacking a prominent, ventral, spur-like seta near apex; head not subglobose, oral opening comparatively small (Figs 154–156, 172–174)	the *leucoproctum* group

### The *faciale* Group

**Species included:** *Hydrochasma castilloi* sp. n., *Hydrochasma crenulum* sp. n., *Hydrochasma digitatum* sp. n., *Hydrochasma faciale* (Williston), *Hydrochasma patens* (Cresson), *Hydrochasma rictum* sp. n., *Hydrochasma sinuatum* sp. n., *Hydrochasma spinosum* sp. n., *Hydrochasma viridum* sp. n., *Hydrochasma williamsae* sp. n.

**Diagnosis.** This species group is distinguished from others within *Hydrochasma* by the following combination of characters: Head: Subglobose; oral opening large, often gaping. Thorax: Hindtibia with prominent, spur-like, ventral, subapical seta. Abdomen: Tergites without sharply demarcated lateral line or lateral wedges, although darker dorsomedially than on lateral margins. Male terminalia: Ventral epandrial extensions usually relatively wide, if narrow, then tapered to apex; ventral margin of epandrium bifurcate, often deeply.

#### Key to species of the *faciale* group

**Table:** 

1	Oral opening comparatively small (Fig. 1); base of epandrium almost as wide as epandrial length (Fig. 57); hypandrium in lateral view deep, pocket-like (Fig. 60)	*Hydrochasma williamsae* sp. n.
–	Oral opening comparatively large; head subglobose (Figs 18–20, 36–38, 49–51); epandrial length much longer than width; hypandrium in lateral view shallow, appearing almost linear	2
2	Mesonotum generally gray but with some light metallic green to blue coloration	3
–	Mesonotum light brown, lacking any metallic coloration	4
3	Mesonotum with extensive metallic green coloration, extended laterally to notopleuron and presutural area; medial surface of basal flagellomere mostly dark colored	*Hydrochasma viridum* sp. n.
–	Mesonotum with faint bluish or slightly greenish metallic coloration, especially on posterior portion, not on notopleuron; medial surface of basal flagellomere mostly yellow	*Hydrochasma patens* Cresson
4	Ventral epandrial extensions elongate, very slender, parallel sided (Figs 13, 39)	5
–	Ventral epandrial extensions moderately slender, tapered, not parallel sided	6
5	Base of epandrial process in lateral view with incised notch (Fig. 40)	*Hydrochasma sinuatum* sp. n.
–	Base of epandrial process narrowed but not notched (Fig. 14)	*Hydrochasma digitatum* sp. n.
6	Epandrial extensions bearing several spine-like setulae in a U-shaped arch subapically (Fig. 44)	*Hydrochasma spinosum* sp. n.
–	Epandrial extension lacking spine-like setulae on U-shaped arch	7
7	Epandrial extensions together as a parallel-sided, apically truncate process in posterior view, in lateral view with a subapical, U-shaped notch (Figs 7–8)	*Hydrochasma crenulum* sp. n.
–	Epandrial extensions tapered, lacking a U-shaped notch in lateral view	8
8	Epandrial extensions twice length of cerci (Fig. 31)	*Hydrochasma rictum* sp. n.
–	Epandrial extensions only slightly longer than length of cerci (Figs 2, 21)	9
9	Epandrial extensions in lateral view (Fig. 3) with apical portion tapered to narrowly rounded point; hypandrium in ventral view with anterior margin as an arrow head (Fig. 4)	*Hydrochasma castilloi* sp. n.
–	Epandrial extensions in lateral view (Fig. 22) with apical portion broadly formed, not tapered; hypandrium in ventral view robustly U-shaped (Fig. 23)	*Hydrochasma faciale* (Williston)

**Figure 1.**
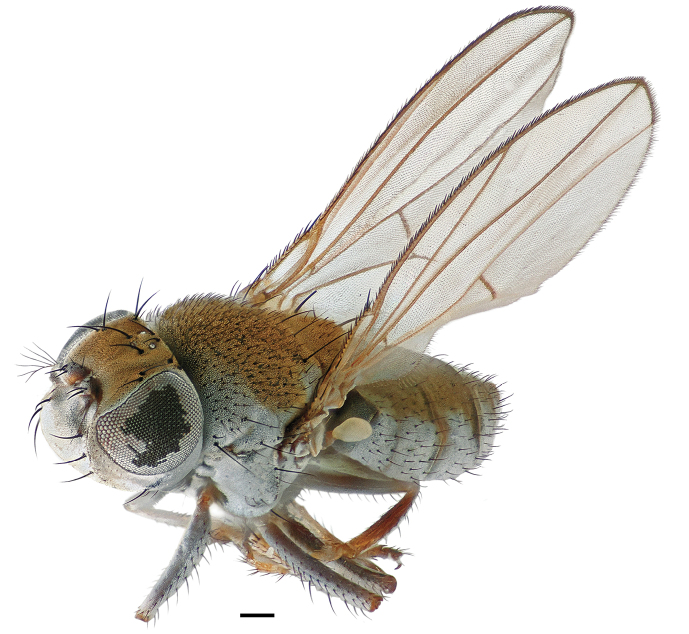
Habitus frontispiece of *Hydrochasma williamsae* sp. n. (Costa Rica. Cartago: La Suiza). Scale bar = 0.1 mm

#### 1. Hydrochasma castilloi sp. n.

http://zoobank.org/23D6E14F-1498-43E3-8E5A-277235EB30B5

http://species-id.net/wiki/Hydrochasma_castilloi

Figs 2 –6

##### Diagnosis.

**Figures 2–5.**
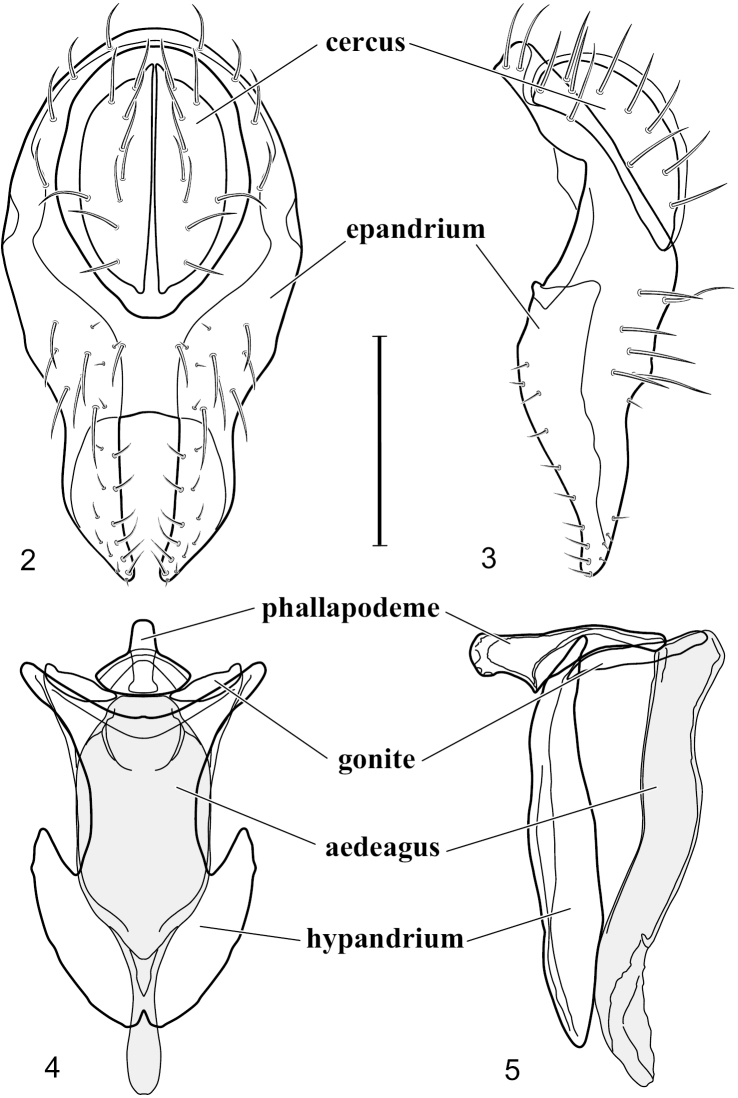
*Hydrochasma castilloi* sp. n. (Ecuador. Loja: Catamayo) **2** epandrium and cerci, posterior view **3** same, lateral view **4** internal structures of male terminalia (aedeagus [shaded], phallapodeme, gonite, hypandrium), ventral view **5** same, lateral view. Scale bar = 0.1 mm.

This species is distinguished from congeners by the following combination of characters: Small shore flies, body length 1.60–1.90 mm. *Head*: Subglobose, very broad ventrally, oral opening comparatively large. Antennal coloration variable, entirely yellow to nearly evenly divided between yellowish and dark gray, dorsal and anterior surfaces of pedicel and basal flagellomere extensively dark gray. Parafacial silvery white, concolorous with face; gena-to-eye ratio 0.20–0.23. *Thorax*: mesonotum yellowish to golden brown; pleural area gray. Wing with costal vein ratio 0.72–0.81; M vein ratio 0.43–0.44. Forefemur bearing a distinctive, comb-like row of stout setulae along anteroventral and posteroventral surfaces; tibiae mostly gray; hindtibia bearing a large, spur-like seta ventroapically. *Abdomen*: Tergites 1–4 with dorsum mostly grayish brown, with lateral margin unevenly demarcated from silvery gray to gray portion, lacking grayish wedges laterally on tergites 2–4; tergite 5 light gray to silvery gray, similar to coloration along lateral margins of preceding tergites but with posterior margin blackish brown to slate black, similar to coloration of medial area on tergites 1–4; medial coloration on tergites 1–4 wide, occupying most of dorsum, dark, grayish to slate black. Male terminalia (Figs 2–5): Combined structures generally moderately elongate, in posterior view (Fig. 2) height less than twice width, generally setulose; epandrium with dorsal arch above cerci narrowly developed, completely connected, in posterior view (Fig. 2) as an inverted, rounded U on dorsal half, ventral half more narrowly developed than dorsal portion, lateral margins shallowly curved medially, deeply bifurcate medially, medial bifurcation as wide as ventral epandrial arm, narrowly pointed apically, bearing longer setulae along dorsal arch and at midheight, in lateral view (Fig. 3) narrowly rectangular on dorsal 1/4, nearly vertical, thereafter ventrally oriented anteroventrally, more thickly developed, apical 1/3 tapered to narrowly rounded apex; cerci moderately long, height about 2.5× width, semi-hemispherical (Fig. 2), not attached lateroventrally with epandrium; aedeagus in lateral view (Fig. 5) elongate, parallel sided, about 8× longer than width, tubular, shallowly sinuous, with apical portion moderately pointed, in ventral view (Fig. 4) mostly parallel sided on basal 2/3 except for immediate base that is tapered medially, apical 1/3 abruptly narrowed, slightly spatulate, apex narrow, rounded; phallapodeme in lateral view (Fig. 5) somewhat narrow, moderately elongate, unevenly bar-like with medial margin shallowly arched, keel skewed toward attachment with hypandrium, narrow but extended, extended margin roundly rectangular, in ventral view (Fig. 4) broadly as a short T with arms short and triangular; gonite in lateral view (Fig. 5) narrow, elongate, bar-like, shallowly sinuous, in ventral view (Fig. 4) very shallowly curved; hypandrium in lateral view (Fig. 5) elongate, shallow, tapered at both apices, more narrowly so posteriorly, in ventral view (Fig. 4) with posterior half to 2/3 spindle shaped, becoming wider with short extended lateral processes just before posterior margin, posterior margin shallowly emarginated, anterior portion as an arrowhead with posteriorly directed robust extensions and with lateral and anterior margins shallowly arched, roundly pointed.

##### Type material.

The holotype male of *Hydrochasma castilloi* is labeled “Ecuador[.]Loja Catamayo Dec. 1955/Collr.Levi-Castillo/USNM ENT 00118297 [plastic bar code label]/HOLOTYPE ♂ *Hydrochasma castilloi* Mathis & Zatwarnicki, USNM [red].” The holotype is double mounted (glued to a paper triangle)), is in excellent condition, and is deposited in the USNM. Sixteen paratypes (11♂, 9♀; USNM) bear the same label data as the holotype.

##### Type locality.

Ecuador. Loja: Catamayo (03°59'S, 79°21'W), Dec 1955.

##### Other specimens examined.

ECUADOR. **El Oro:** Puerto Bolivar (03°16'S, 79°60'W), Dec 1955, R. Levi-Castillo (1♂, 2♀; USNM). **Guayas:** Cone (02°10.1'S, 79°38'W), Jun 1955, R. Levi-Castillo (1♀; USNM).

##### Distribution

**Figure 6.**
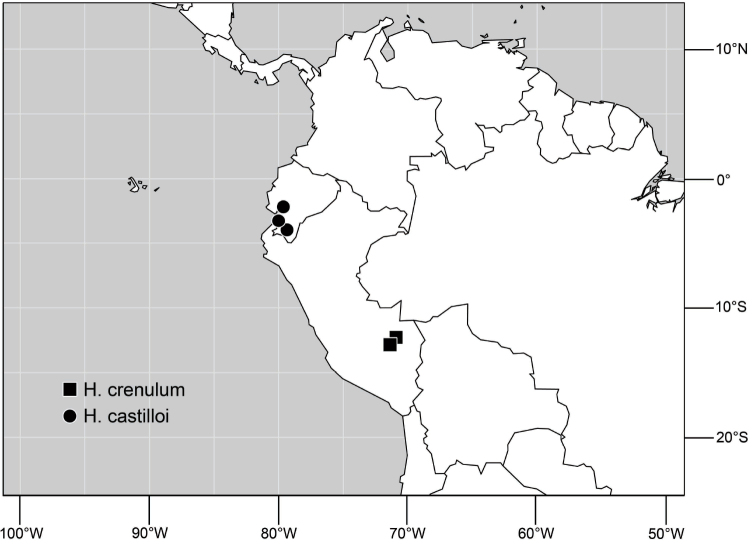
Distribution of *Hydrochasma castilloi* sp. n. and *Hydrochasma crenulum* sp. n.

(Fig. 6). Neotropical: Ecuador (El Oro, Guayas, Loja).

##### Etymology.

The specific epithet, *castilloi*, is a genitive patronym to honor Roberto Levi-Castillo, the collector of the type series and many other shore flies from Ecuador.

##### Remarks.

This species has been frequently misidentified as *Hydrochasma patens* in collections. Our study of the holotype of *Hydrochasma patens*, however, reveals it to be a separate and distinct, although closely related species. Fortunately, the structures of the male terminalia are very diagnostic and distinctive. These structures in *Hydrochasma castilloi* are generally much more robust and shorter in both posterior and lateral views (Figs 2–5). In addition, the aedeagus in lateral view (Fig. 5) is shallowly sinuous, and the hypandrium in ventral view (Fig. 4) has much shorter, posterolateral processes. In *Hydrochasma patens*, the aedeagus in lateral view is shallowly arched, not sinuous, and the posterolateral hypandrial processes are three to four times longer than those of *Hydrochasma castilloi*. In general form, structures of the male terminalia of this species are more similar to those of *Hydrochasma faciale* and *Hydrochasma williamsae*, being relatively short and robustly developed. The dorsum of the epandrium in *Hydrochasma castilloi* is complete though thinly developed; whereas in *Hydrochasma faciale* and *Hydrochasma williamsae*, the dorsal band is incomplete resulting in an open dorsal margin to the cercal cavity.

#### 2. Hydrochasma crenulum sp. n.

http://zoobank.org/13CB8B02-1EB0-4F42-8E44-2CC0E9E78246

http://species-id.net/wiki/Hydrochasma_crenulum

Figs 6 –10

##### Diagnosis.

**Figures 7–10.**
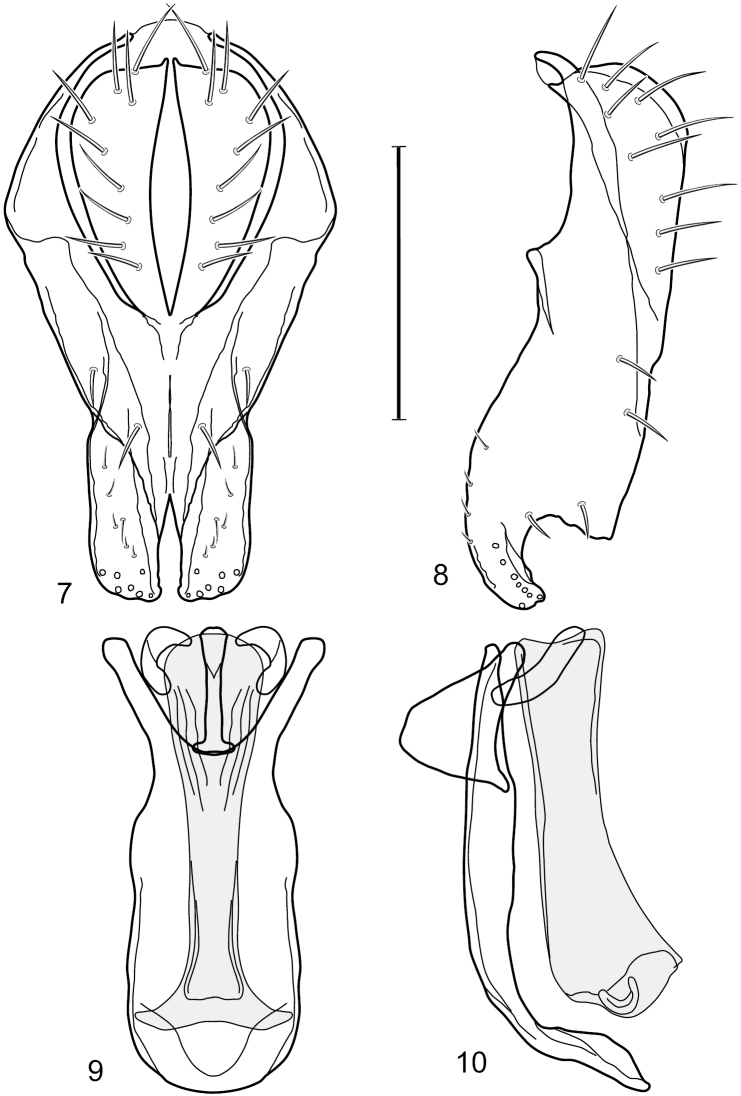
*Hydrochasma crenulum* sp. n. (Peru. Cuzco: Paucartambo, Atalaya) **7** epandrium and cerci, posterior view **8** same, lateral view **9** internal structures of male terminalia (aedeagus [shaded], phallapodeme, gonite, hypandrium), ventral view **10** same, lateral view. Scale bar = 0.1 mm.

This species is distinguished from congeners by the following combination of characters: Small shore flies, body length 1.25–1.90 mm. *Head*: Subglobose generally, very broad ventrally, oral opening comparatively large. Antenna mostly dark gray; at least pedicel black. Parafacial silvery white, concolorous with facial coloration; gena-to-eye ratio 0.21–0.24. *Thorax*: Mesonotum yellowish to golden brown; pleural area gray. Wing with costal vein ratio 0.93–0.96; M vein ratio 0.44–0.46. Forefemur bearing a distinctive, comb-like row of stout setulae along anteroventral surface; tibiae mostly gray; hindtibia bearing a long, spur-like seta ventroapically. *Abdomen*: Tergites broadly darker gray medially, becoming lighter gray laterally but unevenly, lacking wedge-shaped gray to silvery gray areas or a sharply contrasted demarcation between dorsal and lateral coloration; tergite 5 of male gray. Male terminalia (Figs 7–10): Combined structures generally moderately elongate, in posterior view height not quite twice width; epandrium with dorsal arch interrupted medially, not connected above cerci; generally sparsely setulose; in posterior view (Fig. 7) as an inverted, divergent U on dorsal third, medial portion with lateral margins angled medially, robustly developed, ventral apical portion somewhat rectangular, lateral margins (height less than that of cerci) parallel sided, partially bifurcate, narrowly and deeply incised medially, broadly truncate apically, in lateral view (Fig. 8) widely notched and with a robust, curved, partially hook-like ventral process; cerci moderately long, height about 1.5× width (Fig. 7), tenuously attached ventrolaterally with epandrium; aedeagus in lateral view (Fig. 10) moderately elongate, about 2.5× longer than greatest width, tubular, slightly wider basally and more so apically, with apical portion unevenly rounded, in ventral view (Fig. 9) moderately wide basally, thereafter tapered medially but just before apex, apex distinctly but narrowly extended laterally; phallapodeme in lateral view (Fig. 10) broad and short with width subequal to length, keel wide and long, extended margin irregularly rounded, in ventral view (Fig. 9) narrowly T-shaped with arms short and right angled; gonite in lateral view (Fig. 10) narrow, moderately elongate, bar-like, slightly arched, in ventral view (Fig. 9) more conspicuously curved; hypandrium in lateral view (Fig. 10) elongate, shallow, anterior 1/3 obtusely angled, parallel sided, in ventral view (Fig. 9) with posterior third divergently U-shaped, anterior portion slightly narrower, plate-like, anterior margin broadly rounded.

##### Type material.

The holotype male of *Hydrochasma crenulum* is labeled “PERU. Cuzco: Pau-cartambo, Atalaya,R[ío]. AltoMadredeDios,600m,4Sep1988,WNMathis/USNM ENT 00092795 [plastic bar code label]/HOLOTYPE ♂ *Hydrochasma crenulum* Mathis & Zatwarnicki, USNM [red].” The holotype is double mounted (minuten in a block of plastic), is in excellent condition, and is deposited in the USNM. Fifty paratypes (34♂, 16♀; USNM) bear the same label data as the holotype.

##### Type locality.

Peru. Cuzco: Paucartambo, Atalaya (Río Alto Madre de Dios; 12°53.3'S, 71°21.6'W; 600 m).

##### Other specimens examined.

Neotropical. PERU. **Madre de Dios:** Diamante (Río Alto Madre de Dios; 12°19.9'S, 70°57.5'W; 400 m), 7 Sep 1988, W. N. Mathis (4♂, 10♀; USNM); Río Manu, Erika (near Salvación; 12°50.7'S, 71°23.3'W; 550 m), 5-6 Sep 1988, W. N. Mathis (4♂, 2♀; USNM).

##### Distribution

(Fig. 6). Neotropical: Peru (Cuzco, Madre de Dios).

##### Etymology.

The species epithet, *crenulum*, is of Latin derivation and means notched, referring to the conspicuous notch in the epandrium that is best seen in lateral view.

##### Remarks.

The shapes of structures of the male terminalia of *Hydrochasma crenulum* are unmistakable and readily distinguish this species from congeners, especially those of the *faciale* group. This is especially evident in the ventral portion of the epandrium in lateral view that is generally robustly developed, is conspicuously and widely notched, and terminates as an extended, curved process.

#### 3. Hydrochasma digitatum sp. n.

http://zoobank.org/8F13EFB9-BD3E-4EC4-B1CF-66A3A8F1FC18

http://species-id.net/wiki/Hydrochasma_digitatum

Figs 12 – 17

##### Diagnosis.

This species is distinguished from other congeners by the following combination of characters: Small shore flies, body length 1.40–1.90 mm. *Head*: Antenna mostly dark gray; parafacial silvery white, concolorous with facial coloration; gena-to-eye ratio 0.15–0.17. *Thorax*: Wing with costal vein ratio 0.77–0.81; M vein ratio 0.47–0.49. Forecoxa whitish gray to gray; hindtibia with prominent ventroapical, shallowly curved, spur-like seta. *Abdomen*: Tergites broadly brown medially, becoming lighter gray laterally but unevenly, almost shallowly wedge-like, tergite 5 of male gray with a thin, medial brown stripe (Fig. 12). Male terminalia (Figs 13–16): Combined structures generally moderately elongate, in posterior view height over 2× width, generally setulose on cerci, setulae sparse or minute ventrally; epandrium with dorsal arch above cerci not interrupted, narrowly connected, in posterior view (Fig. 13) with basal 1/2 somewhat rectangular with angles rounded, apical 1/2 narrowed, extended as separate, narrow, almost parallel sided processes with a deep and moderately wide gap between processes, lateral margins very shallowly arched, each process rounded apically, in lateral view (Fig. 14) generally shallowly arched with curvature relatively even, widest just ventrad of cercus, apical third abruptly narrowed, tapered, curved anteriorly subapically, apex narrowly rounded, bearing minute setulae; aedeagus in lateral view (Fig. 16) elongate, narrow, irregularly tubular, shallowly curved, gradually tapered on basal 3/4, thereafter widened to form a boxlike apex (angles rounded), in ventral view (Fig. 15) mostly parallel sided on basal 2/3, apical 1/3 more narrowed but still parallel sided, apex rounded; phallapodeme in lateral view (Fig. 16) oriented perpendicular to aedeagus, narrowly elongate, keel very evident as an extension on portion toward hypandrium, portion toward aedeagal base rod-like in ventral view (Fig. 15) as a short, rounded, isosceles triangle with a circular structure at base, foreshortened; gonite in lateral view (Fig. 16) rod-like, straight, narrow, in ventral view (Fig. 15) short due to foreshortening; hypandrium in lateral view (Fig. 16) elongate, comparatively wide, anterior margin narrowly rounded, posterior margin as 2 narrow, elongate processes, which form a deep gap between, posterior projection acutely pointed and shorter than anterior projection, in ventral view (Fig. 15) as an arrowhead that is deeply incised posterolaterally with a tapered, elongate, acutely pointed, posterolateral projection, base extended posteriorly, parallel sided along middle portion, thereafter flared posterolaterally as 2 symmetrical processes, forming a wide, V-shaped posterior margin.

**Figures 11–12.**
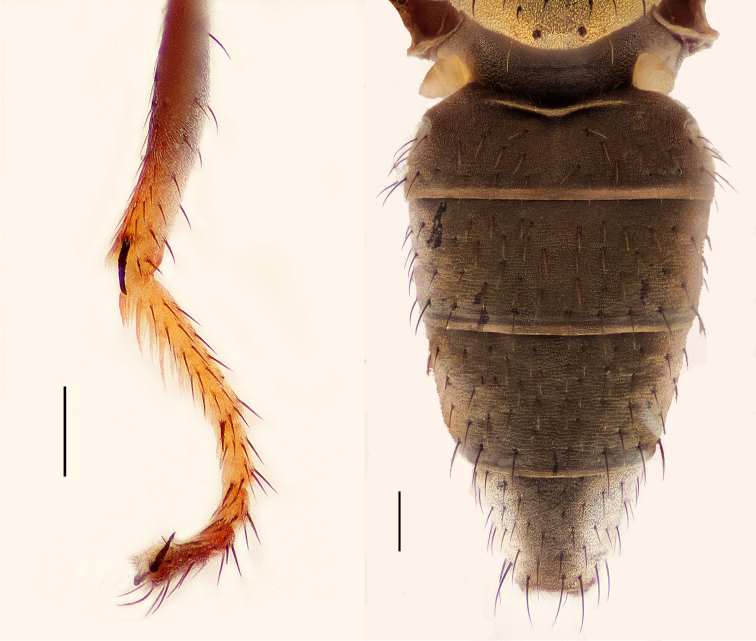
**11** *Hydrochasma williamsae* sp. n. (USA. New Mexico. Grant: Mimbres River) hindtibia and hindtarsus, posterior view **12** *Hydrochasma digitatum* sp. n. (Peru. Madre de Dios: Río Manu, Erika), abdomen of male, dorsal view. Scale bar = 0.1 mm.

**Figures 13–16.**
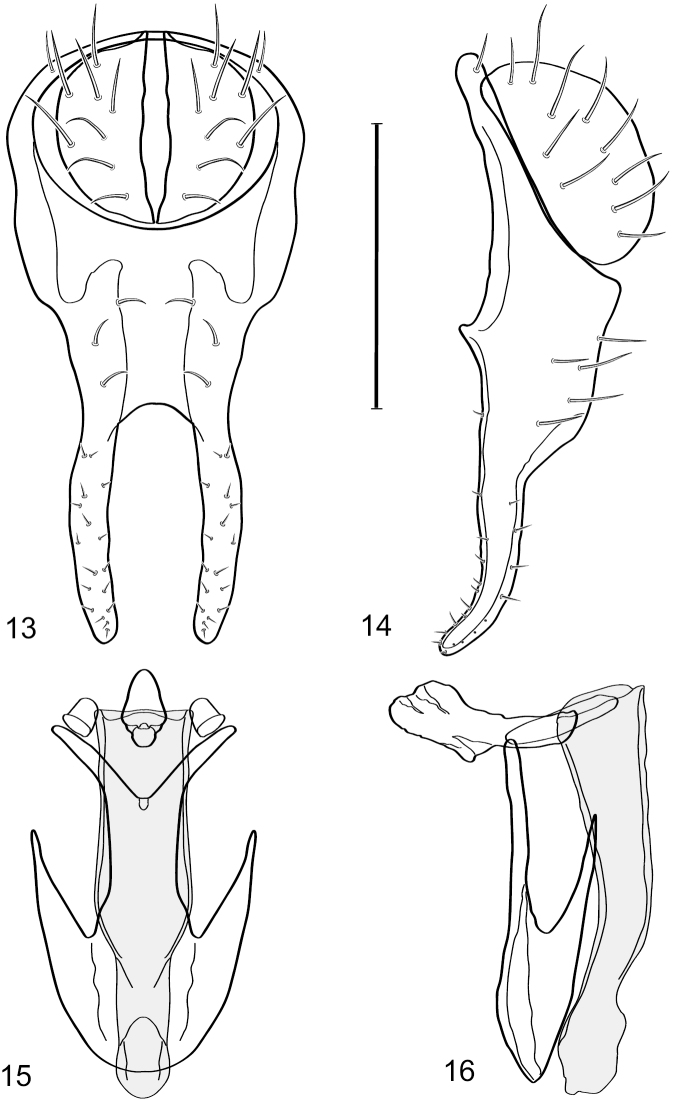
*Hydrochasma digitatum* sp. n. (Peru. Madre de Dios: Diamante) **13** epandrium and cerci, posterior view **14** same, lateral view **15** internal structures of male terminalia (aedeagus [shaded], phallapodeme, gonite, hypandrium), ventral view **16** same, lateral view. Scale bar = 0.1 mm.

##### Type material.

The holotype male of *Hydrochasma digitatum* is labeled “PERU. Madre de Dios: Manu, Diamante, 400 m, 12°25' [sic 19.9']S, 70°57[.5]'W, R[ío]. AltoMadre de Dios, 7 Sep 1988, W.N.Mathis/USNM ENT 00285974 [plastic bar code label]/HOLOTYPE ♂ *Hydrochasma digitatum* Mathis & Zatwarnicki, USNM [red].” The holotype is double mounted (minuten in a block of plastic), is in excellent condition, and is deposited in the USNM. Thirteen paratypes (3♂, 10♀; USNM) bear the same label data as the holotype.

##### Type locality.

Peru. Madre de Dios: Diamante (Río Alto Madre de Dios; 12°19.9'S, 70°57.5'W; 400 m).

##### Other specimens examined.

Neotropical. PERU. **Cuzco:** Paucartambo, Atalaya (Río Alto Madre de Dios; 12°53.3'S, 71°21.6'W; 600 m), 4 Sep 1988, W. N. Mathis (1♂; USNM). **Madre de Dios:** Río Manu, Erika (near Salvación; 12°50.7'S, 71°23.3'W; 550 m), 5–6 Sep 1988, A. Freidberg, W. N. Mathis (2♂, 1♀; USNM).

##### Distribution

**Figure 17.**
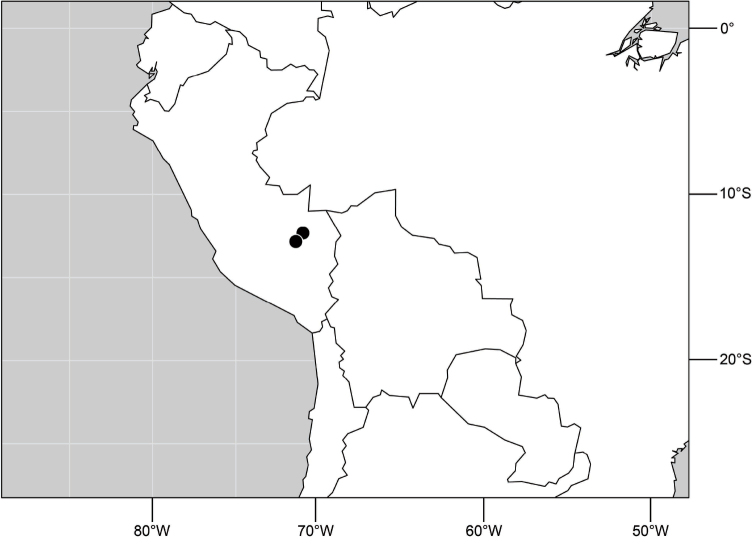
Distribution of *Hydrochasma digitatum* sp. n.

(Fig. 17). Neotropical: Peru (Cuzco, Madre de Dios).

##### Etymology.

The species epithet, *digitatum*, is of Latin derivation and refers to the finger-like ventral extensions of the epandrium.

##### Remarks.

This species, as implied by its name, is distinguished from congeners by the elongated, slender, very shallowly curved, digitiform, ventral epandrial processes. These digitiform processes are separated from each other in posterior view by a deep, narrowly U-shaped pocket or gap. In lateral view, these processes are tapered and slightly curved subapically.

#### 4. Hydrochasma faciale

(Williston)

http://species-id.net/wiki/Hydrochasma_faciale

Figs 18 – 25

Discocerina faciale Williston, 1896: 396 [West Indies. St. Vincent; LT ♂ (designated by Mathis and Edmiston 1991: 825–826), BMNH].
Hydrochasma faciale Wirth, 1968: 8 [generic combination; Neotropical catalog]. Mathis and Edmiston 1991: 825–826 [review of Williston’s St. Vincent species]. Mathis and Zatwarnicki 1995: 182 [world catalog]. Mathis 1997: 36 [review, Belize].
Hydrochasma zernyi Hendel, 1936: 103 [Brazil. Pará: Santarém; ST ♂, NMW]. Cresson 1938: 25 [review]; 1946: 141 [review]. Wirth 1968: 8 [synonymy with *Hydrochasma faciale*].
Hydrochasma capax Cresson, 1938: 26; 1942: 113 [list, Arizona and California]; 1946: 141 [review]. Wirth 1965: 738 [Nearctic catalog]; 1968: 8 [synonymy with *Hydrochasma faciale*]. Cole 1969: 398 [list, Western North America].

##### Diagnosis.

**Figures 18–20.**
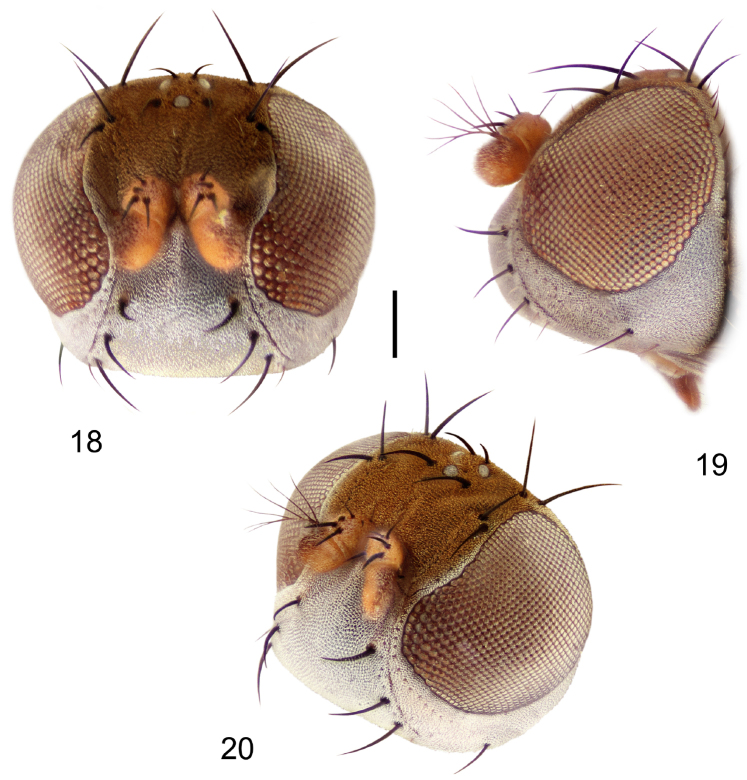
*Hydrochasma faciale* (Williston) (St. Vincent. St. Patrick: Cumberland Bay) **18** head, anterior view **19** same, lateral view **20** same, oblique view. Scale bar = 0.1 mm.

**Figures 21–24.**
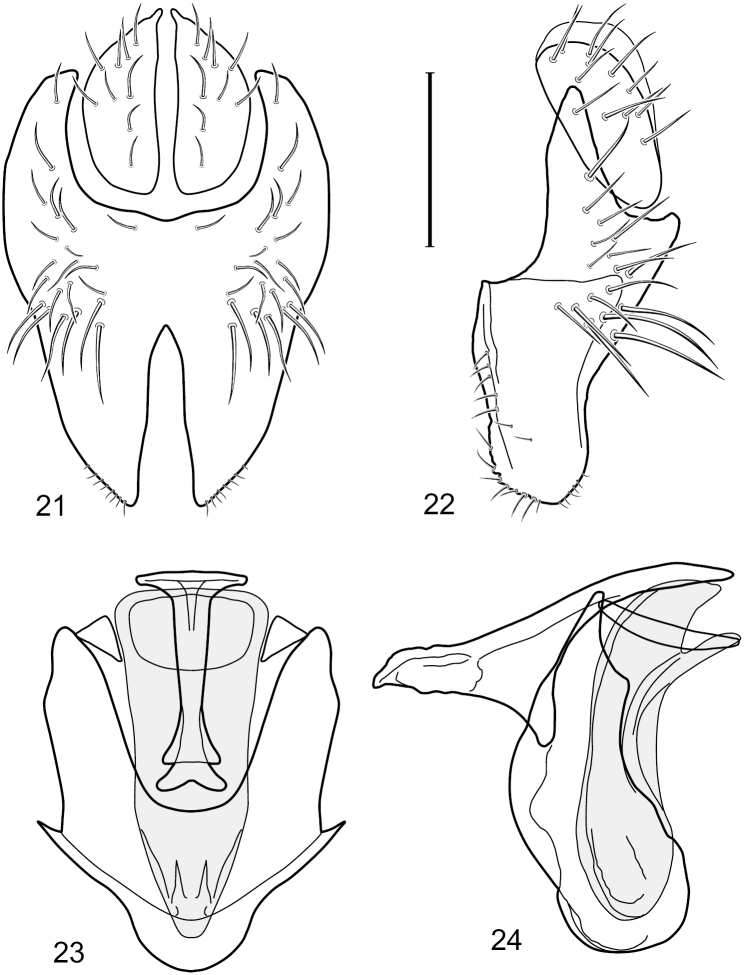
*Hydrochasma faciale* (Williston) (Dominica: Layou R.) **21** epandrium and cerci, posterior view **22** same, lateral view **23** internal structures of male terminalia (aedeagus [shaded], phallapodeme, gonite, hypandrium), ventral view **24** same, lateral view. Scale bar = 0.1 mm.

This species is distinguished from congeners by the following combination of characters: Small to moderately small shore flies, body length 1.40–2.50 mm. *Head*: Head subglobose, very broad ventrally, oral opening comparatively large; antennal coloration variable, entirely yellow to nearly evenly divided between yellowish and dark gray, dorsal and anterior surfaces of pedicel and basal flagellomere extensively dark gray; parafacial silvery white, concolorous with face (Figs 18–20); gena-to-eye ratio 0.25–0.28. *Thorax*: Mesonotum yellowish to golden brown; pleural area gray. Wing with costal vein ratio 0.71–0.75; M vein ratio 0.54–0.56. Forefemur bearing a distinctive, comb-like row of stout setulae along anteroventral surface; tibiae mostly gray; hindtibia bearing a large, spur-like seta ventroapically. *Abdomen*: Tergites broadly brown medially, lacking wedge-shaped, gray to silvery gray areas laterally; tergite 5 of male brown, concolorous with tergites 2–4. Male terminalia (Figs 21–24): Combined structures generally slightly elongate, in posterior view height about 1.5× width, generally setulose, especially dorsally but also along ventral margins; epandrium with dorsal arch above cerci attenuated, not connected, in posterior view (Fig. 21) with cercal cavity forming a U, with arms of U robustly developed, ventral portion with each lateral half robustly developed, tapered to ventral apex, apex pointed, deeply and narrowly incised medially, medial incision 3× longer than wide, widest at apex, in lateral view (Fig. 22) with ventral portion robust, apex obtusely pointed; cerci moderately long, height more than twice width, widely semi-hemispherical (Fig. 21), pointed dorsally, not attached lateroventrally or ventrally with epandrium; aedeagus in lateral view (Fig. 24) elongate, almost 4× longer than width, tubular, shallowly curved, apex rounded, in ventral view (Fig. 23) mostly tapered from base to pointed apex, apical third more abruptly tapered; phallapodeme in lateral view (Fig. 24) triangular with extended keel skewed and pointed on portion toward attachment with hypandrium, in ventral view (Fig. 23) an elongate, moderately robust T with double crosses, arms of cross short; gonite in lateral view (Fig. 24) narrow, moderately elongate, bar-like, very shallowly curved, in ventral view (Fig. 23) shallowly curved; hypandrium in lateral view (Fig. 24) moderately elongate, sack-like, moderately wide, tapered to point posteriorly, anterior margin broadly rounded, in ventral view (Fig. 23) very broadly and robustly U-shaped, with tiny, pointed lateral extensions from base laterally.

##### Type material.

The lectotype male of *Discocerina faciale* Williston (designated by Mathis and Edmiston 1991: 825) is labeled “Co-type [circular label with a yellow border]/Windward side St. Vincent, W.I. H. H. Smith./W.Indies. 1907-66./Discocerina facialis Will. [handwritten, two red submarginal borders]/LECTOTYPE Discocerina facialis Will. ♂ By W. N.Mathis [handwritten except for “LECTOTYPE” and “By”, black sub-border].” The lectotype is double mounted (pin in rectangular piece of cardboard), is in poor condition (right foreleg, mid legs missing), and is deposited in the BMNH. There is also one female paralectotype (BMNH). Williston, in the original description, noted “Five specimens. St. Vincent.” The other syntypes (1♂, 2♀; BMNH) are apparently representatives of *Hydrochasma incisum* (no silvery triangular patches on dorsum of abdomen).

The lectotype male of *Hydrochasma zernyi* Hendel, here designated to preserve stability and make more universal the use of this name, is labeled “Unt. Amazonas [Brazil. Pará] Santarem 18. VIII 27. [18 Aug 1927] Zerny [“18. VIII” handwritten]/Hydrochas-ma Zernyi H. [handwritten]/Type [red]/LECTOTYPE ♂ Hydrochasma zernyi Hendel By Mathis & Zatwarnicki [handwritten except for “LECTOTYPE” and “By”; black sub-border].” The lectotype is double mounted (minuten in a rectangular block of foam plastic), is in good condition, and is deposited in NMW. There are also four (3♂, 1♀) paralectotypes in the NMW that bear the same locality label.

The holotype female of *Hydrochasma capax* Cresson is labeled “Gualan Guatemala 2–15 [handwritten] [19]05/1096/TYPE Hecamedoides CAPAX E.T. Cresson, Jr. [species name handwritten].” The holotype is double mounted (paper triangle), is in good condition (mid- and hindlegs on right side missing), and is deposited in the ANSP (6533).

##### Type locality.

West Indies. St. Vincent (13°10'N, 61°14'W).

##### Other specimens examined.

Nearctic. UNITED STATES. ARIZONA. **Pima:** near Kits Peak, Baboquivari Mountains (32°00N, 111°36'W), 7-8 Aug 1916 (1♀; ANSP).

CALIFORNIA. **San Diego:** San Diego (32°42.9'N, 117°09.4'W; lakeside), 4 Aug 1931, J. M. Aldrich (1♂; ANSP).

Neotropical. BELIZE. **Stann Creek:** Dangriga (16°58'N, 88°13'W), 3-4 Apr 1993, W. N. Mathis (3♂, 2♀; USNM); Twin Cays (south end of East Island; 16°49.4'N, 88°06.3'W), Mar 1988, W. N. Mathis (1♂; USNM).

BRAZIL. **Paraná:** Morretes (25°28'S, 48°59.1'W), 29 Aug 2000, W. N. and D. Mathis (9♂, 3♀; USNM). **São Paulo:** Ubatuba, Praia Puruba (23°21'S, 44°55.6'W; beach), 29 Mar 2010, D. and W. N. Mathis (9♂, 1♀; DZUP, USNM).

GUYANA. Karanambo, Rupununi River (ox bow; 03°45.1'N, 59°18.6'W), 2 Apr 1994, W. N. Mathis (1♂, 1♀; USNM); Kato, Chiung River (04°39.7'N, 59°50.0'W), 1 May 1995, W. N. Mathis (2♂; USNM); Moco-Moco (30 km E Lethem in Kanuku Mountains; 03°18.2'N, 59°39.0'W), 29 Apr 1995, W. N. Mathis (2♂; USNM); Pirara Ranch and River (03°32.1'N, 59°40.5'W), 24–25 Apr 1995, W. N. Mathis (9♂, 13♀; USNM).

TRINIDAD and TOBAGO. Tobago. **St. John:** Speyside (11°18'N, 60°32'W), 13-15 Jun 1993, W. N. Mathis (2♂, 2♀; USNM).

West Indies. CUBA. **Pinar del Rio:** Soroa (22°47.7'N, 83°W), 4-6 Dec 1994, W. N. Mathis (6♂, 12♀; USNM); Soroa (2 km E; 22°47.7'N, 83°W), 29 Apr 1983, W. N. Mathis (1♀; USNM).

DOMINICA. Cabrits Swamp (15°35'N, 61°29'W), 22-25 Mar 1965, W. W. Wirth (1♀; USNM); Layou River mouth (15°23.6'N, 61°25.5'W), 9 Jan-24 Mar 1965, W. W. Wirth (43♂, 44♀; USNM); Macoucheri (15°26.6'N, 61°27'W; seashore), 1 Feb-8 Mar 1965, W. W. Wirth (2♂, 2♀; USNM); Rosalie (15°22.3'N, 61°15.3'W), 23 Mar 1989, W. N. Mathis (2♂, 2♀; USNM).

DOMINICAN REPUBLIC. **La Vega:** Constanza (ca. 16 km SE; 18°50.6'N, 70°40.7'W; 1580 m), 15 May 1998, D. and W. N. Mathis (1♂; USNM); El Rio (9.5 km E; 19°0.9'N, 70°33.5'W; 980 m), 6-7 May 1995, W. N. Mathis (1♂, 1♀; USNM); Jarabacoa (1-2 km S; 19°06.9'N, 70°37'W; 520 m), 8-21 May 1995, W. N. Mathis (1♂; USNM); Rio Camu (3.5 km NW La Vega; 19°13.7'N, 70°35.2'W; 100 m), 10 May 1995, W. N. Mathis (9♂, 4♀; USNM); Salto Baiguate (near Jarabacoa; 19°05.5'N, 70°36.9'W; 570 m), 9 May 1995, W. N. Mathis (1♂; USNM). **Peravia:** San José Ocoa (10 km NE; 18°35'N, 70°25.6'W), 21 May 1998, D. and W. N. Mathis (1♀; USNM). **Puerto Plata:** Rio Camu (14 km E Puerto Plata; 19°41.9'N, 70°37.4'W), 17 May 1995, 1997, D. and W. N. Mathis (6♂, 3♀; USNM); Rio Pérez (near Imbert; 19°44.1'N, 70°50.2'W), 24 May 1998, D. and W. N. Mathis (6♂, 1♀; USNM).

GRENADA. **St. George:** Beauséjour Bay (12°05.5'N, 61°44.9'W), 21 Sep 1996, W. N. Mathis (1♂; USNM). **St. John:** Palmiste (12°08.7'N, 61°44.4'W), 21 Sep 1996, W. N. Mathis (3♂, 1♀; USNM). **St. Patrick:** Levera Bay (12°13.6'N, 61°36.6'W), 18 Sep 1996, W. N. Mathis (2♂; USNM).

JAMAICA. **Clarendon:** Grantham (18°09.3'N, 77°23.8'W; 340 m), 16 Apr 2000, W. N. Mathis (5♂; USNM). **Portland:** Berridale (18°06.5'N, 76°20'W), Rio Grande River, 25 Apr 2000, W. N. Mathis (6♂; USNM). **St. Andrew:** Mavis Bank (1.7 km E; 18°02.4'N, 77°39.5'W; 575 m), Yallahs River, 21-22 Apr-1 May 2000, W. N. Mathis (3♂, 4♀; USNM); Mavis Bank (4.3 km SE; 18°01.4'N, 76°38.1'W; 480 m); Yallahs River, 22-23 Apr 2000, W. N. Mathis (1♂; USNM). **St. Thomas:** Bath Fountain Spring (17°57.6'N, 76°21.3'W), 15 May 1996, D. and W. N. Mathis, H. B. Williams (3♀; USNM); Bath River, Bath (17°56.8'N, 76°21.6'W), 16 May 1996, D. and W. N. Mathis, H. B. Williams (8♂, 2♀; USNM); Hagley Gap (1 km E; 18°00.1'N, 76°36.7'W), 16 May 1996, D. and W. N. Mathis, H. B. Williams (1♂, 1♀; USNM); Mt. Lebanus (17°58.2'N, 76°32.7'W), 16 May 1996, D. and W. N. Mathis, H. B. Williams (2♂; USNM); Yallahs River (mouth; 17°53'N, 76°35.6'W), 14 May 1996, D. and W. N. Mathis, H. B. Williams (1♂, 2♀; USNM).

PUERTO RICO. Adjuntas (18°09.8'N, 66°43.2'W), 22 Sep 1995, D. and W. N. Mathis (10♂, 5♀; USNM).

ST. LUCIA. Dauphin Boguis (1.6 km S Marquis; 14°01'N, 60°55'W), 17 Jun 1991, D. and W. N. Mathis (3♂, 5♀; USNM); Micoud (13°49'N, 60°54'W), 15 Jun 1991, D. and W. N. Mathis (1♂; USNM); Soufrière (beach; 13°51'N, 60°54'W), 11-12 Jun 1991, D. and W. N. Mathis (3♂, 1♀; USNM).

ST. VINCENT. **Charlotte:** Colonarie (13°14.4'N, 61°06.9'W; beach), 29 Mar 1989, W. N. Mathis (2♂; USNM); Spring (13°11.1'N, 61°08.5'W), 6 Sep 1997, W. N. Mathis (2♂, 2♀; USNM). **St. Andrew:** Buccament Bay (near beach; 13°11'N, 61°16'W), 25-28 Mar-8 Jun 1989, 1991, D. and W. N. Mathis (20♂, 23♀; USNM); Layou (13°12'N, 61°17'W), 8 Jun 1991, D. and W. N. Mathis (2♂, 1♀; USNM). **St. David:** Richmond Beach (13°18.6'N, 61°14.1'W), 28 Mar 1989, W. N. Mathis (1♀; USNM). **St. Patrick:** Cumberland Bay (13°16'N, 61°16'W), 28 Mar-8-15 Sep 1989, 1991, 1997, A. Freidberg, D. and W. N. Mathis (20♂, 10♀; USNM); Cumberland River (3 km E Spring Village; 13°15'N, 61°14'W), 10 Jun 1991, D. and W. N. Mathis (19♂, 2♀; USNM); Wallilabou (beach; 13°15'N, 61°16'W), 27 Mar 1989, W. N. Mathis (5♂, 10♀; USNM).

##### Distribution

**Figure 25.**
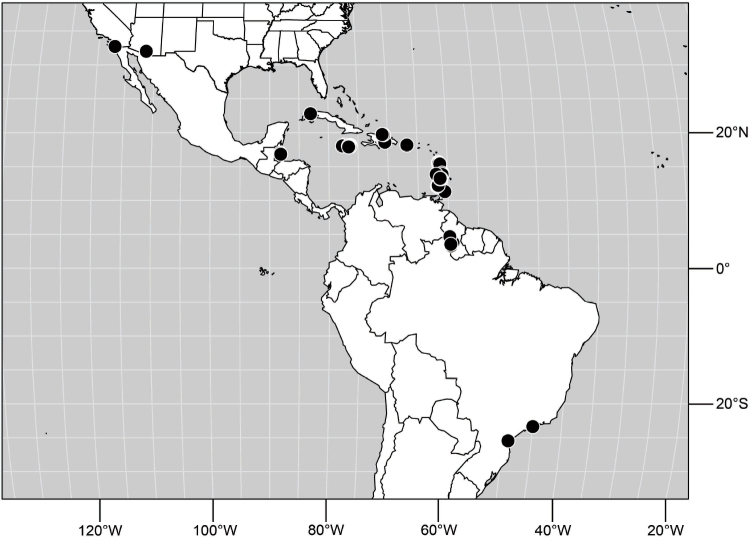
Distribution of *Hydrochasma faciale* (Williston).

(Fig. 25). Nearctic: United States (Arizona, California). Neotropical: Belize (Stann Creek), Brazil (Pará, Paraná, São Paulo), Guatemala, Guyana, Trinidad and Tobago, West Indies (Cuba, Dominica, Dominican Republic, Grenada, Jamaica, Puerto Rico, St. Lucia, St. Vincent).

Natural History.-This species occurs along freshwater streams and rivers or sometimes brackish water systems (where a freshwater stream or river is entering the ocean) that have sandy areas that are mostly bare of vegetation.

##### Remarks.

We follow Wirth (1968) in recognizing the two junior synonyms of *Hydrochasma faciale*, as noted in the synonymy.

Although this species is externally similar to *Hydrochasma williamsae*, as are structures of the male terminalia, they are distinguished from each other in details of the latter structures. The epandrium of this species, for example, is broadly rounded dorsolaterally, not angulate as in *Hydrochasma williamsae*, and the ventral margin in lateral view is robustly developed and broadly but unevenly rounded apically, not tapered and pointed as in *Hydrochasma williamsae*.

#### 5. Hydrochasma patens

Cresson

http://species-id.net/wiki/Hydrochasma_patens

Figs 26 –30

Hydrochasma patens Cresson, 1938: 27 [Uruguay. Montevideo; HT ♂, BMNH]; 1946: 141 [review]. Wirth 1968: 8 [Neotropical catalog]. Mathis and Zatwarnicki 1995: 183 [world catalog].

##### Diagnosis.

**Figures 26–29.**
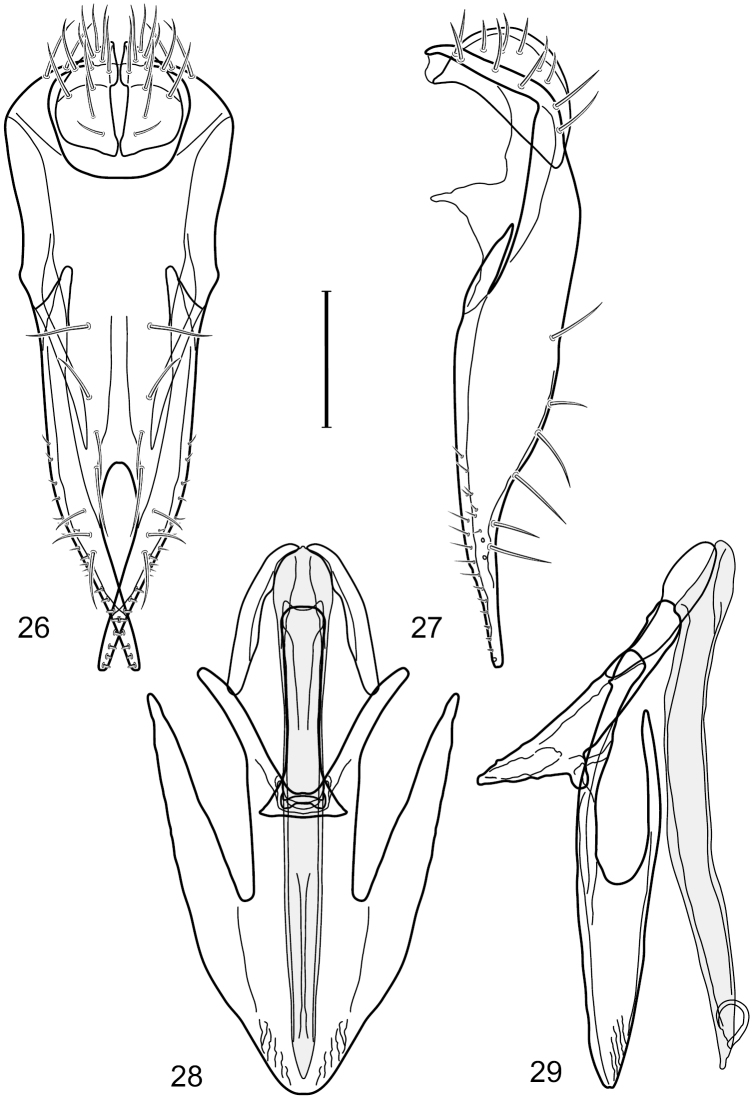
*Hydrochasma patens* Cresson (Chile. Talca: Rio Lircay, 11 km N Talca) **26** epandrium and cerci, posterior view **27** same, lateral view **28** internal structures of male terminalia (aedeagus [shaded], phallapodeme, gonite, hypandrium), ventral view **29** same, lateral view. Scale bar = 0.1 mm.

This species is distinguished from congeners by the following combination of characters: Small to moderately small shore flies, body length 1.85–2.60 mm. *Head*: Subglobose, very broad ventrally, oral opening comparatively large. Pedicel mostly black; medial surface of basal flagellomere extensively yellow, lateral surface with some blackish coloration. Parafacial silvery white, concolorous with face; gena-to-eye ratio 0.32–0.35. *Thorax*: Mesonotum generally gray but with faint bluish or slightly greenish metallic coloration, especially on posterior portion, including scutellum, not on notopleuron or lateral margins of scutellum; pleural area gray. Wing with costal vein ratio 0.50–0.62; M vein ratio 0.49–0.51. Femora and tibiae gray, tarsi yellow; forefemur lacking a distinctive, comb-like row of stout setulae along anteroventral surface but with a posteroventral row of 5–7 short, spine-like setae; hindtibia bearing a long, spur-like seta ventroapically. *Abdomen*: Tergites generally dull gray, becoming brownish gray medially; lacking wedge-shaped, gray to silvery gray areas; tergite 5 gray. Male terminalia (Figs 26–29): Combined structures generally moderately elongate, in posterior view height slightly less than 3× width, generally sparsely setulose, especially dorsally; epandrium with dorsal arch above cerci relatively well developed, thin, completely connected, in posterior view (Fig. 26) as an inverted U on dorsal third to half, ventral portion with lateral margins shallowly concave medially, cruciate subapically, deeply bifurcate medially, medial bifurcation almost as wide as ventral process of epandrium at same level, extended epandrial process tapered, apex rounded; cerci moderately long, height nearly twice width, widely semi-hemispherical (Fig. 26), not attached lateroventrally with epandrium; aedeagus in lateral view (Fig. 29) elongate, about 5× longer than wide, tubular, shallowly curved, slightly tapered toward apex with apical portion moderately pointed and with a subapical, rounded projection, in ventral view (Fig. 28) mostly tapered from base to pointed apex; phallapodeme in lateral view (Fig. 29) narrowly triangular with extended keel skewed and pointed on portion toward attachment with hypandrium, in ventral view (Fig. 28) an elongate, robust T with crossbar very short; gonite in lateral view (Fig. 29) narrow, elongate, bar-like, very shallowly curved and spatulate, in ventral view (Fig. 28) shallowly curved; hypandrium in lateral view (Fig. 29) elongate, moderately shallow, tapered at both apices and with lateral, posteriorly directed process evident, in ventral view (Fig. 28) with posterior half spindle shaped, becoming wider with long extended lateral processes just before posterior margin, posterior margin deeply emarginated, emargination V-shaped, anterior portion as an arrowhead with posteriorly directed narrow, elongate, lateral extensions and with lateral and anterior margins shallowly arched, forming a V-shaped anterior margin.

##### Type material.

The holotype male of *Hydrochasma patens* is labeled “Holo- type [round label with red margin]/9100/Uruguay: Montevideo, 21–22. i. 1927 [21–22 Jan 1927]. F. & M. Edwards. B. M. 1927–63/TYPE Hydrochasma PATENS E. T. Cresson, Jr. [carmine; Hydrochasma PATENS handwritten]”. The holotype is double mounted (minuten in a plastic rectangle), is generally in good condition (lacking left foretarsus), and is deposited in the BMNH.

##### Type locality.

Uruguay. Montevideo: Montevideo (34°53.3'S, 56°11'W).

##### Other specimens examined.

Neotropical. CHILE. **Osorno:** Lago Puyehue (SE shore; 40°45'S, 72°25.2'W), 10 Feb 1978, W. N. Mathis (3♂, 10♀; USNM). **Talca:** Rio Lircay (11 km N Talca; 35°23'S, 71°39'W; 85 m), 23 Jan1978, W. N. Mathis (22♂, 31♀; USNM).

##### Distribution

**Figure 30.**
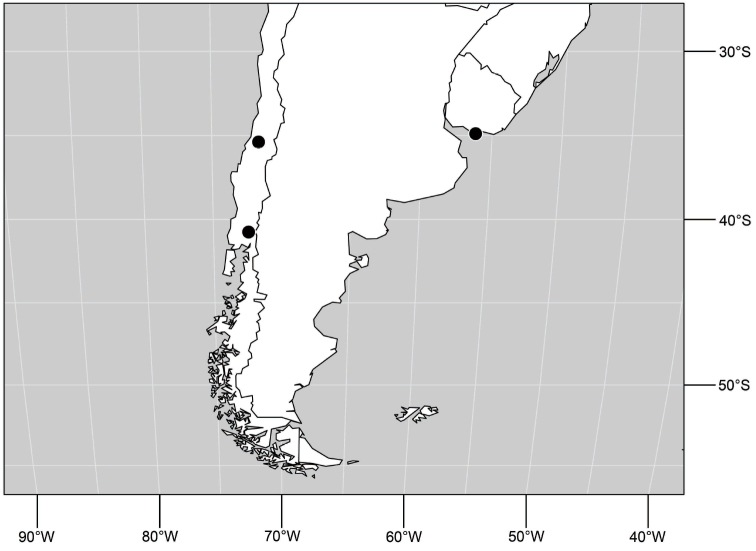
Distribution of *Hydrochasma patens* Cresson,

(Fig. 30). Neotropical: Uruguay, Chile (Osorno, Talca).

##### Remarks.

The identity of this species has been confused, resulting in misidentifications in collections that we have examined. Structures of the male terminalia of this species are distinctive, however, and distinguish it from congeners, especially the elongated and slender general conformation of these structures, which can only be mistaken with those of *Hydrochasma viridum*. Both of these species have elongated, very slender, ventrolateral epandrial processes that are very similar. The hypandrium, especially in ventral view, however, has more elongated, posterolaterally directed processes in *Hydrochasma patens*. Additionally, the dorsum of the head and the mesonotum are mostly gray with only faint bluish or greenish coloration.

#### 6. Hydrochasma rictum sp. n.

http://zoobank.org/3DFFF259-D419-4134-9DED-96B0C2DC420F

http://species-id.net/wiki/Hydrochasma_rictum

Figs 31 –35

##### Diagnosis.

**Figures 31–34.**
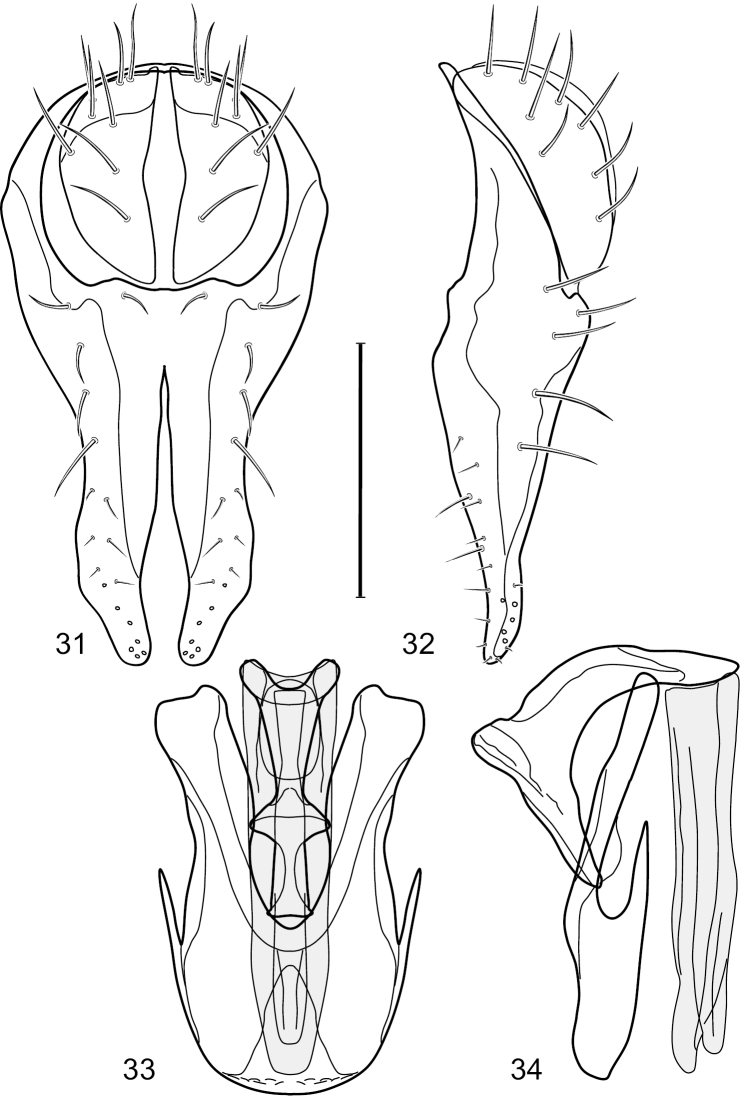
*Hydrochasma rictum* sp. n. (Costa Rica. Guanacaste: Playa Puerto Soley) **31** epandrium and cerci, posterior view **32** same, lateral view **33** internal structures of male terminalia (aedeagus [shaded], phallapodeme, gonite, hypandrium), ventral view **34** same, lateral view. Scale bar = 0.1 mm.

This species is distinguished from other congeners by the following combination of characters: Small to moderately small shore flies, body length 1.55–2.00 mm. *Head*: Antenna mostly dark gray; parafacial silvery white, concolorous with facial coloration; gena-to-eye ratio 0.27–0.30. *Thorax*: Wing with costal vein ratio 0.62–0.65; M vein ratio 0.57–0.60. Forefemur with comb-like row of distinct, short, stout setulae along apical half of posteroventral margin; hindtibia bearing a short seta, length subequal to width at same level. *Abdomen*: Tergites broadly dark gray medially, lacking wedge-shaped, gray to silvery gray areas laterally; tergite 5 of male mostly gray but with vague, slate gray medial stripe. Male terminalia (Figs 31–34): Combined structures generally moderately elongate, in posterior view height less than twice width (1.85×), generally setulose on cerci, setulae sparse or minute ventrally; epandrium with dorsal arch above cerci not interrupted, narrowly connected, in posterior view (Fig. 31) with basal 1/2 somewhat ovate, apical 1/2 moderately narrowed, extended as 2 separate, moderately narrow, almost parallel sided processes with a deep, narrow gap between processes, lateral margins sinuous, each process narrowly rounded apically, in lateral view (Fig. 32) generally narrowly triangular on ventral 2/3, tapered, straight, widest just ventrad of cercus, apical third tapered, straight, apex narrowly rounded, bearing minute setulae; aedeagus in lateral view (Fig. 34) simple, straight, tubular, in ventral view (Fig. 33) mostly parallel sided on basal 2/3–3/4, apical 1/4 tapered to bluntly rounded apex; phallapodeme in lateral view (Fig. 34) oriented perpendicular to aedeagus, robustly elongate, keel evident as an extension on portion toward hypandrium, portion toward aedeagal base tapered, in ventral view (Fig. 33) pedunculate with double T with arms short and thin, perpendicular; gonite apparently lacking; hypandrium in lateral view (Fig. 34) elongate, comparatively narrow, deeply hook-like, anterior margin narrowly rounded, extension narrow, parallel sided, elongate, in ventral view (Fig. 33) more or less rectangular, with deeply incised posterolateral, short, narrow projections, posterior extensions robust, slightly flared posterolaterally, with deep pocket between, forming a U-shaped posterior margin.

##### Type material.

The holotype male of *Hydrochasma rictum* is labeled “**HONDURAS.** Cortés:San Pedro Sula (8 km S) 15°25.7'N, 88°01.4'W[,] 25–26 September1995[,] Dianne & W.N.Mathis/USNM ENT 00138958 [plastic bar code label]/HOLOTYPE ♂ *Hydrochasma rictum* Mathis & Zatwarnicki, USNM [red].” The holotype is double mounted (minuten in a block of plastic), is in good condition (abdomen and hindlegs removed, abdomen dissected, parts in an attached microvial), and is deposited in the USNM. A female paratype bears the same label data as the holotype.

##### Type locality.

Honduras. Cortés: San Pedro Sula (8 km S; 15°25.7'N, 88°01.4'W).

##### Other specimens examined.

Neotropical. COSTA RICA. **Guanacaste:** Playa Puerto Soley (11°02.5'N, 85°40.1'W; beach), 16 Jun 2003, D. and W. N. Mathis (1♂, 1♀; USNM). **Limón:** R. V. S. Gandoca-Manzanillo, desembocadura de Laguna Gandoca (09°35.4'N, 82°35.8'W), 18 May 2004, D. Briceño (1♂; INBIO).

##### Distribution

**Figure 35.**
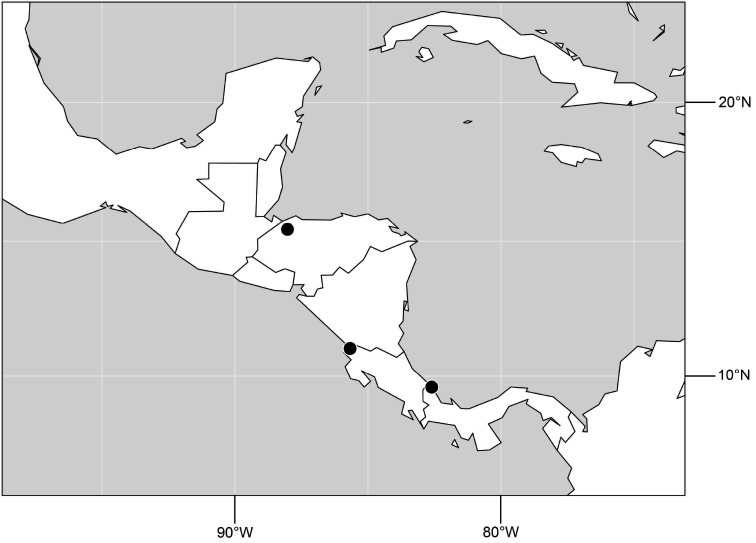
Distribution of *Hydrochasma rictum* sp. n.

(Fig. 35). Neotropical: Costa Rica (Guanacaste, Limon), Honduras (Cortés).

##### Etymology.

The species epithet, *rictum*, is of Latin derivation and means open mouth, referring to the large, open mouth of this species.

##### Remarks.

Structures of the male terminalia of this species are similar to those of *Hydrochasma digitatum* but are distinguished from that species in having more robustly developed ventral epandrial processes that are moderately angulate laterally in posterior view. Moreover, the hypandrium of this species in ventral view is much more robustly developed with a broad, bluntly rounded anterior margin.

#### 7. Hydrochasma sinuatum sp. n.

http://zoobank.org/A5946E37-A8F0-41D1-89A3-B41CCD84BBC1

http://species-id.net/wiki/Hydrochasma_sinuatum

Figs 36 – 43

Hydrochasma species Mathis, 1997:182 [review, Belize].

##### Diagnosis.

This species is distinguished from other congeners by the following combination of characters: Small shore flies, body length 1.50–1.95 mm. *Head*: Antenna mostly dark gray; parafacial silvery white, concolorous with facial coloration; gena-to-eye ratio 0.18–0.20. *Thorax*: Wing with costal vein ratio 0.75–0.76; M vein ratio 0.47–0.49. Forecoxa whitish gray to gray. Hindtibia with prominent ventroapical, shallowly curved, spur-like seta. *Abdomen*: Tergites broadly slate gray medially, lacking wedge-shaped, gray to silvery gray areas laterally; tergite 5 of male gray with faint brown lateral margins, concolorous with tergites 2–4. Male terminalia (Figs 39–42): Combined structures generally moderately elongate, in posterior view height less than twice width (1.83×), dorsal half with setulae twice length of those on ventral half; epandrium with dorsal arch above cerci narrowly connected, in posterior view (Fig. 39) with dorsal margin arched, dorsal half as wide as high, apical half as 2 elongate, narrow, almost parallel sided processes, each oriented slightly medially, forming a deep, medial incision, in lateral view (Fig. 40) with anterior margin conspicuously sinuous, widest ventrad of cercus with a notch-like incision at base of ventral epandrial extension, ventral epandrial extension slightly tapered to rounded apex; cerci moderately elongate, height nearly twice width in posterior view, mostly semi-hemispherical (Fig. 39); aedeagus in lateral view (Fig. 42) elongate, narrow, nearly straight, length about 4× width, tubular, in ventral view (Fig. 41) shallowly emarginate at base, mostly parallel sided on basal half, apical half tapered to narrowly rounded apex; phallapodeme in lateral view (Fig. 42) narrowly elongate, keel very shallow and short, barely evident, in ventral view (Fig. 41) an elongate T with arms very short and thin, perpendicular; gonite in lateral view (Fig. 42) very narrowly elongate, bar-like, nearly straight, in ventral view (Fig. 41) as a robust, short comma; hypandrium in lateral view (Fig. 42) elongate, thin, very shallowly arched, in ventral view (Fig. 41) with elongate, narrow, lateral processes, each slightly more than half length of aedeagus, anterior margin shallowly incised, incision shallowly bifurcate.

**Figures 36–38.**
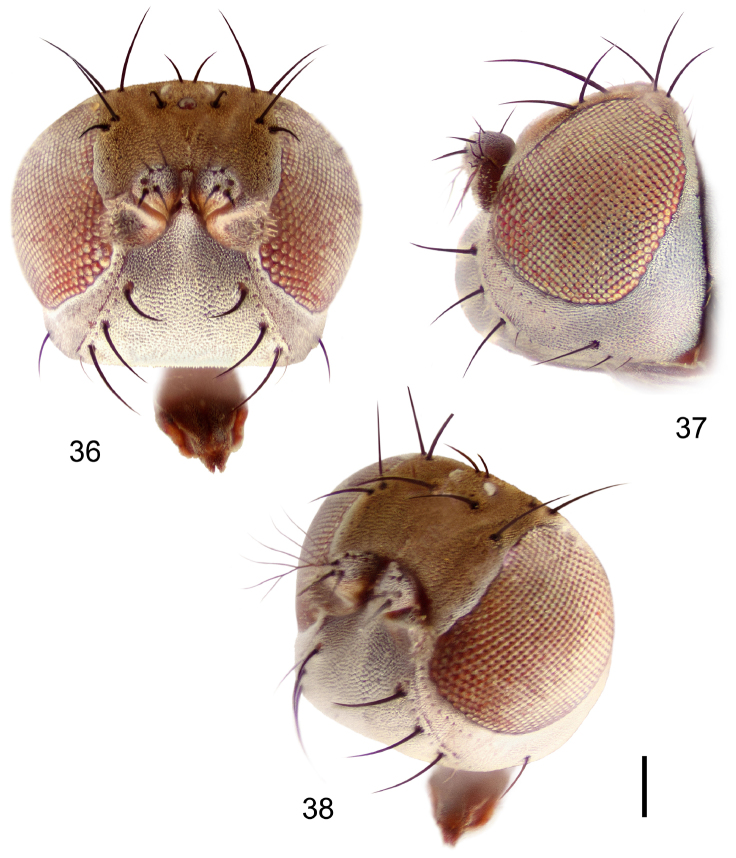
*Hydrochasma sinuatum* sp. n. (Honduras. Cortés: Omoa) **36** head, anterior view **37** same, lateral view **38** same, oblique view. Scale bar = 0.1 mm.

**Figures 39–42.**
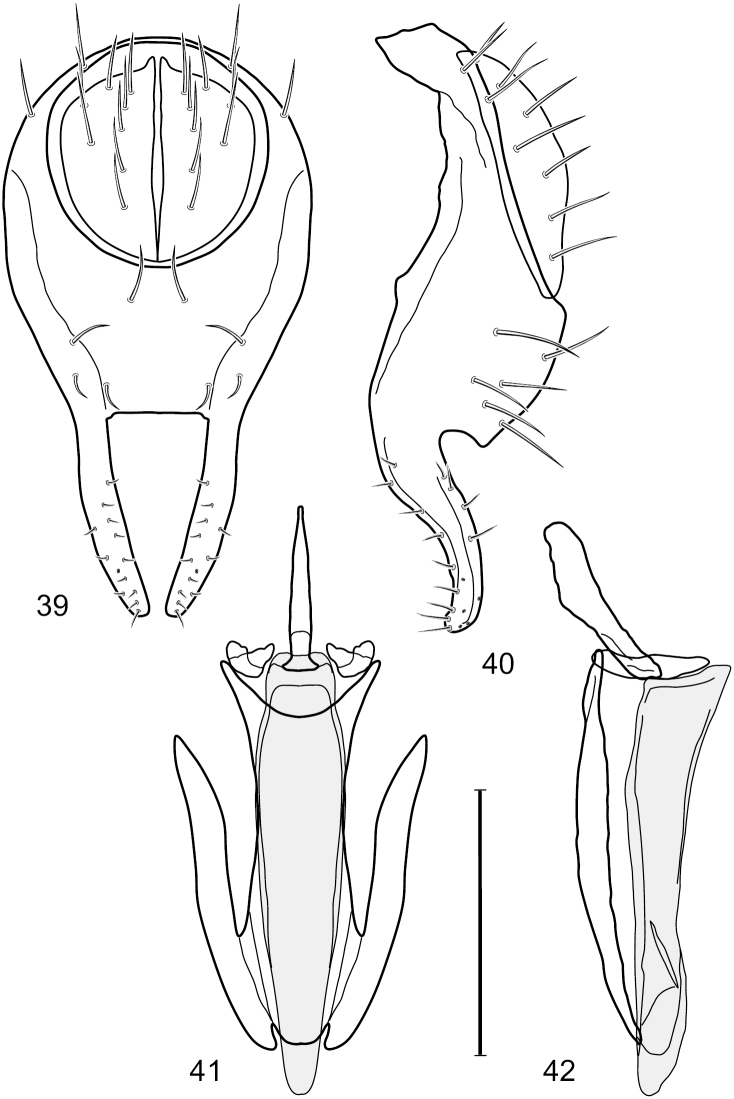
*Hydrochasma sinuatum* sp. n. (Mexico. Sinaloa: 13 km NE of Concordia) **39** epandrium and cerci, posterior view **40** same, lateral view **41** internal structures of male terminalia (aedeagus [shaded], phallapodeme, gonite, hypandrium), ventral view **42** same, lateral view. Scale bar = 0.1 mm.

##### Type material.

The holotype male of *Hydrochasma sinuatum* is labeled “**BELIZE**. Stann Cr[ee]k. Dist[rict]. MullinsRiver[,] (17km N Dangriga)[,] 29 March 1988, Wayne N. Mathis/USNM ENT 00285977 [plastic bar code label]/HOLOTYPE ♂ *Hydrochasma sinuatum* Mathis & Zatwarnicki, USNM [red].” The holotype is double mounted (minuten in a block of plastic), is in excellent condition, and is deposited in the USNM. Fifteen paratypes (6♂, 9♀; USNM) bear the same label data as the holotype.

##### Type locality.

Belize. Stann Creek: Mullins Creek (17 km N Dangriga; 17°06.2'N, 88°17.8'W).

##### Other specimens examined.

Neotropical. BELIZE. **Stann Creek:** Dangriga (16°58'N, 88°13'W), 3–4 Apr 1993, W. N. Mathis (18♂, 8♀; USNM); Silk Grass Creek (16°58'N, 88°13'W), 3 Apr 1993, W. N. Mathis (1♂, 1♀; USNM); Twin Cays, Aanderaa Flats (16°50'N, 88°06'W), 17–21 Mar 1988, W. N. Mathis (1♀; USNM); Twin Cays, West Bay (16°50'N, 88°06'W), 22 May 1988,W. N. Mathis (1♂; USNM).

COSTA RICA. **Guanacaste:** Santa Cruz (14 km S; 10°10.4'N, 85°35.7'W; 180 m), 23 Jun 2001, W. N. Mathis (1♂; USNM).

HONDURAS. **Cortés:** Omoa (16°47.8'N, 87°58.4'W), 26 Sep 1995, D. and W. N. Mathis (2♂, 2♀; USNM).

MEXICO. **Sinaloa:** Concordia (13 km NE; 23°20.7'N, 105°58.4'W), 15 Aug 1960, P. H. Arnaud, Jr., D. C. Rentz, E. D. Ross (7♂, 12♀; CAS, USNM).

PERU. **Madre de Dios:** Río Manu, Erika (near Salvación; 12°50.7'S, 71°23.3'W; 550 m), 5-6 Sep 1988, A. Freidberg, W. N. Mathis (2♂, 1♀; USNM).

##### Distribution

**Figure 43.**
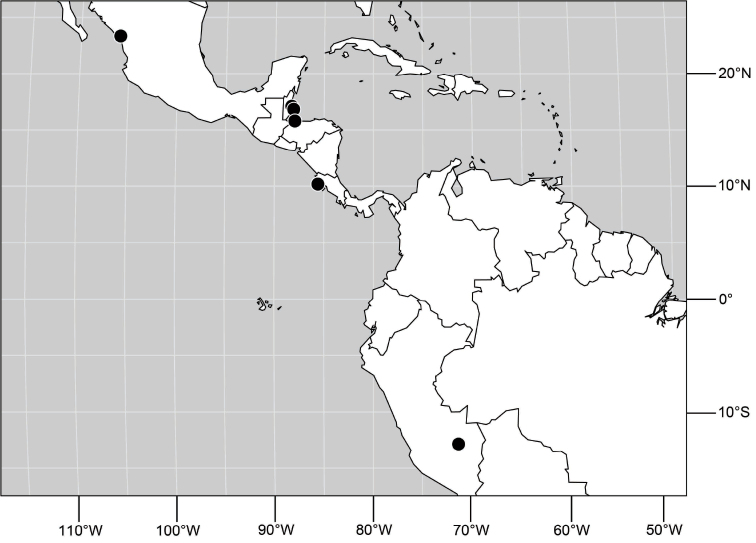
Distribution of *Hydrochasma sinuatum* sp. n.

(Fig. 43). Neotropical: Belize (Stann Creek), Costa Rica (Guanacaste), Honduras (Cortés), Mexico (Sinaloa), Peru (Madre de Dios).

##### Etymology.

The species epithet, *sinuatum*, is of Latin derivation and means winding or bent, referring to the shape of the extended epandrium in lateral view.

##### Remarks.

Although structures of the male terminalia of this species are similar to those of *Hydrochasma digitatum*, they consistently differ in detail. This is particularly evident in the shape of the ventral, epandrial processes where in lateral view there is a basal, posterior notch, and the processes themselves are sinuous and not shallowly arched, as in *Hydrochasma digitatum*. The sinuous profile of the ventral, epandrial process is the basis for the name of this species.

#### 8. Hydrochasma spinosum sp. n.

http://zoobank.org/1A0F2830-E034-4842-9A62-2184C1AF556E

http://species-id.net/wiki/Hydrochasma_spinosum

Figs 44 –48 , 70

##### Diagnosis.

**Figures 44–47.**
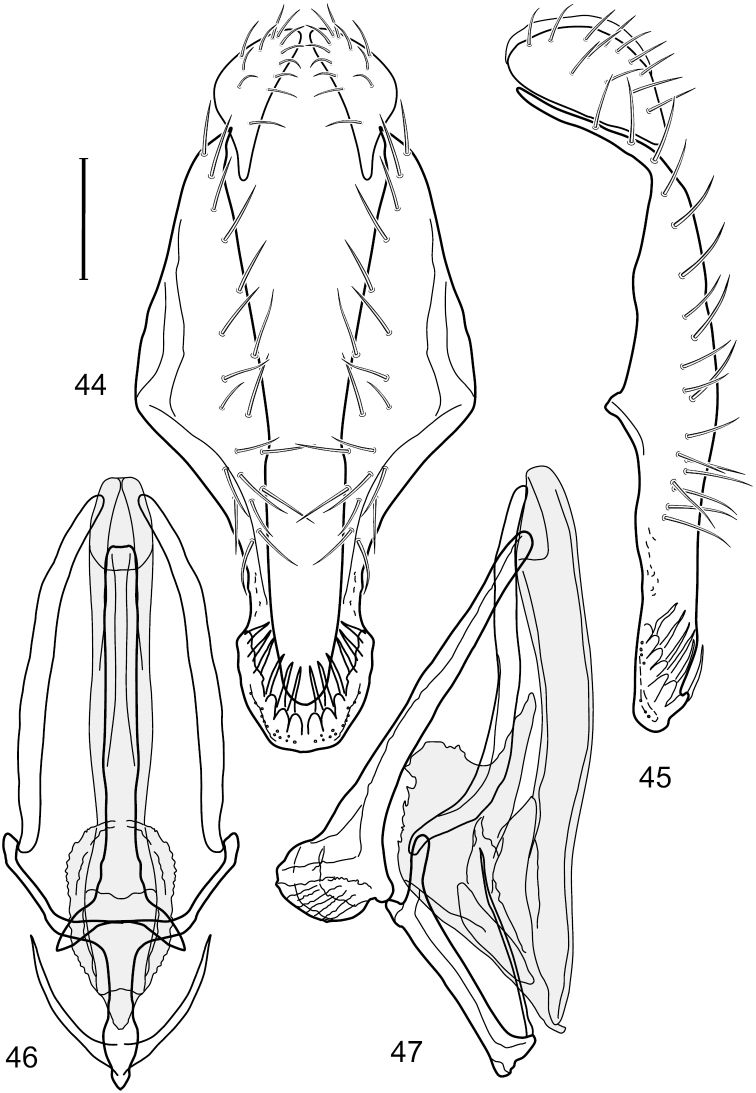
*Hydrochasma spinosum* sp. n. (Costa Rica. Puntarenas: Playa Jacó) **44** epandrium and cerci, posterior view **45** same, lateral view **46** internal structures of male terminalia (aedeagus [shaded], phallapodeme, gonite, hypandrium), ventral view **47** same, lateral view. Scale bar = 0.1 mm.

This species is distinguished from congeners by the following combination of characters: Small to moderately small shore flies, body length 1.50-2.30 mm. *Head*: Antenna mostly dark gray; parafacial silvery white, concolorous with facial coloration; gena comparatively high, gena-to-eye ratio 0.47–0.50. *Thorax*: Wing with costal vein ratio 0.73–1.76; M vein ratio 0.42–0.46. *Abdomen*: Tergites lacking with broad, shallow, wedge-like marking laterally, otherwise tergites 2–4 with wide medial area extensively dark slate gray (Fig. 71); tergite 5 of male light gray to silvery gray anterior margin and thin, medial stripe brownish gray. Male terminalia (Figs 44–47): Combined structures moderately elongate, in posterior view height about twice width; epandrium with dorsal arch above cerci attenuate, not connected, dorsal 2/3 somewhat angulate laterally, gradually expanded to just ventrad of midheight, thereafter narrowed on ventral third, parallel sided, broadly rounded apically, rounded margin of apex with numerous, medially oriented, relatively robust setulae (Fig. 44), other epandrial setulae oriented medially, in lateral view very narrow dorsally (Fig. 45), lateral of cerci, thereafter ventrally nearly straight with slight, anterior angulate expansion at midheight, narrowly rounded apically; cerci short, in lateral view height about twice width (Fig. 45), narrowly semicircular, not attached with epandrium, narrowed ventrally; aedeagus in lateral view (Fig. 47) elongate, with length of sclerotized portion about 10× width, greatly enlarged ventroapically, membranous portion, shape irregular, in ventral view (Fig. 46) also showing expanded apex, spatulate, basal 2/3 narrowed, essentially parallel sided; phallapodeme in lateral view (Fig. 47) narrow, elongate, nearly straight, keel moderately weakly developed, evident at end toward attachment with hypandrium, in ventral view (Fig. 46) with hypandrial end T-shaped with crossbars forming concavity apically, thereafter toward base of aedeagus very gradually expanded to blunt apex; gonite in lateral view (Fig. 47) narrow, elongate, bar-like, obviously arched toward hypandrial end, in ventral view (Fig. 46) very narrow, shallowly arched; hypandrium in lateral view (Fig. 47) short, length about half that of phallapodeme, narrow, straight, bar-like, in ventral view (Fig. 46) widely U-shaped with very thin, pointed arms.

##### Type material.

The holotype male of *Hydrochasma spinosum* is labeled “**COSTA RICA.** Limón: Westfalia (4 km S; 9°54.5'N, 82°59'W; beach), 27 Jun 2001, Amnon Freidberg/USNM ENT 00189486 [plastic bar code label]/HOLOTYPE ♂ *Hydrochasma spinosum* Mathis & Zatwarnicki, USNM [red].” The holotype is double mounted (minuten in a block of plastic), is in excellent condition, and is deposited in the USNM. Fifteen paratypes (10♂, 5♀; USNM) bear the same label data as the holotype or with W. N. Mathis as the collector.

##### Type locality.

Costa Rica. Limón: Westfalia (4 km S; 09°54.5'N, 82°59'W; beach).

##### Other specimens examined.

Neotropical. COSTA RICA. **Guanacaste:** Bagaces Fortuna Z. P. Miravalles (10°43.1'N, 84°51.3'W; Sendero Cabro Muco; 980 m), 8–31 Jul 2002, J. D. Gutierrez (1♂, 2♀; INBio); Murciélago (10°56.5'N, 85°40.9'W), 1 Apr 1988, J. M. Hill, J. M. Mitchell, W. E. Steiner, J. M. Swearingen (2♂, 2♀; USNM); Nandayure, Estero Caletas (09°59.5'N, 85°15.2'W), 21 Mar-22 Oct 2003, D. Briceño (5♂, 1♀; INBio); Nandayure, Corozalito (10°0.1'N, 85°08.7'W), 23 Oct 2003, D. Briceño (2♀; INBio); Nandayure, Costa de Oro (09°55'N, 85°17'W; 11 m), 20 Oct 2003, D. Briceño (1♂, 1♀; INBio); Nandayure, Río Bejuco (09°50'N, 85°20'W; playa), 21 Oct 2003, D. Briceño (5♂, 1♀; INBio); Playa de Cuajiniquil (10°56.1'N, 85°42.2'W; beach), 16 Jun 2003, D. and W. N. Mathis (1♂; USNM). **Limón:** Guandoca, Manzanillo (09°36.8'N, 82°40.9'W), 19–25 May 2004, D. Briceño (1♂, 1♀; INBio); Talamanca, Cahuita (09°43.8'N, 82°50.7'W), 9–10 Dec 2001, E. Rojas (1♀; INBio). **Puntarenas:** Dominical (09°14.8'N, 83°51.4'W; 0–2 m), 11 Jun 2003, D. and W. N. Mathis (8♂, 1♀; USNM); Drake (08°41.4'N, 83°40.1'W; beach), 12 Aug 2001, D. and W.N. Mathis (1♀; USNM); Malpais (09°37.6'N, 85°09.1'W; beach), 21 Jun 2001, D. and W. N. Mathis (1♂, 2♀; USNM); Playa Jacó (09°36.5'N, 84°37.4'W; beach), 13 Jun 2003, D. and W. N. Mathis (5♂; USNM); Pochotal (09°31.4'N, 84°28.4'W; beach), 12–13 Jun 2003, D. and W. N. Mathis (8♂, 3♀; USNM); San Pedrillo (08°37.2'N, 83°44.1'W), 12–14 Aug 2001, D. and W.N. Mathis (3♂, 1♀; USNM).

##### Distribution

**Figure 48.**
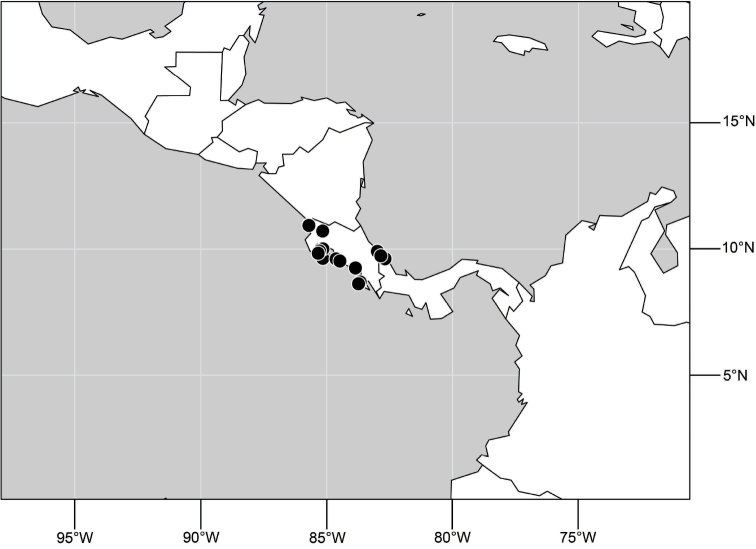
Distribution of *Hydrochasma spinosum* sp. n.

(Fig. 48). Neotropical: Costa Rica (Guanacaste, Limón, Puntarenas).

##### Etymology.

The species epithet, *spinosum*, is of Latin derivation and refers to the spinose apical portion of the epandrium.

##### Remarks.

As implied by its species’ name, *spinosum*, this species is distinguished from congeners by the numerous spine-like structures on the U-shaped, ventroapical portion of the epandrial process. The angulate, lateral epandrial margins are also distinctive.

#### 9. Hydrochasma viridum sp. n.

http://zoobank.org/491CEEA6-5C26-45ED-9AFF-FC829C5D8F1F

http://species-id.net/wiki/Hydrochasma_viridum

Figs 49 – 56

##### Diagnosis.

**Figures 49–51.**
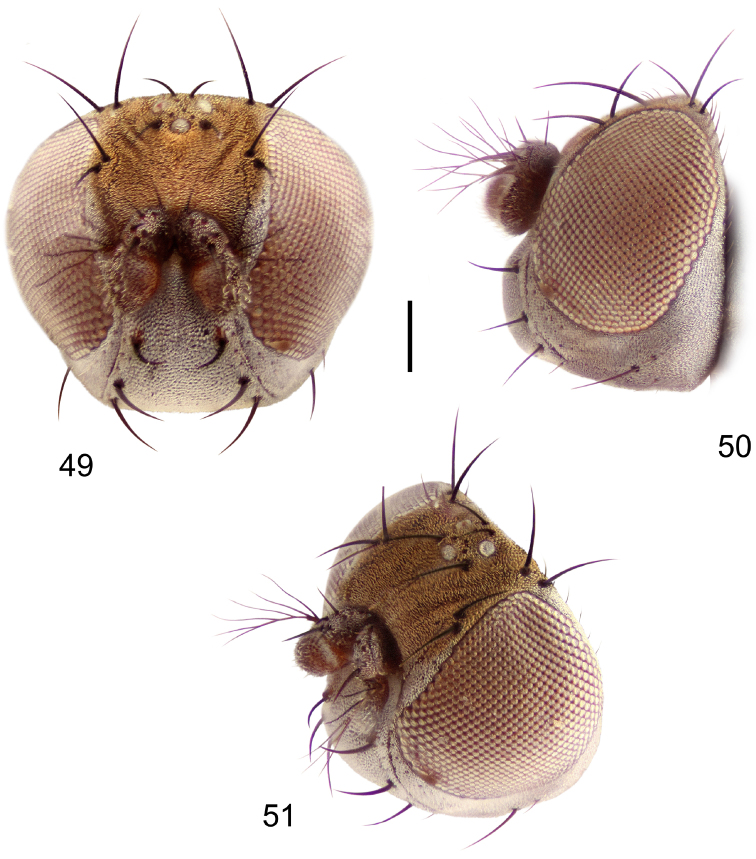
*Hydrochasma viridum* sp. n. (Guyana. Moco-Moco, Lethem) **49** head, anterior view **50** same, lateral view **51** same, oblique view. Scale bar = 0.1 mm.

**Figures 52–55.**
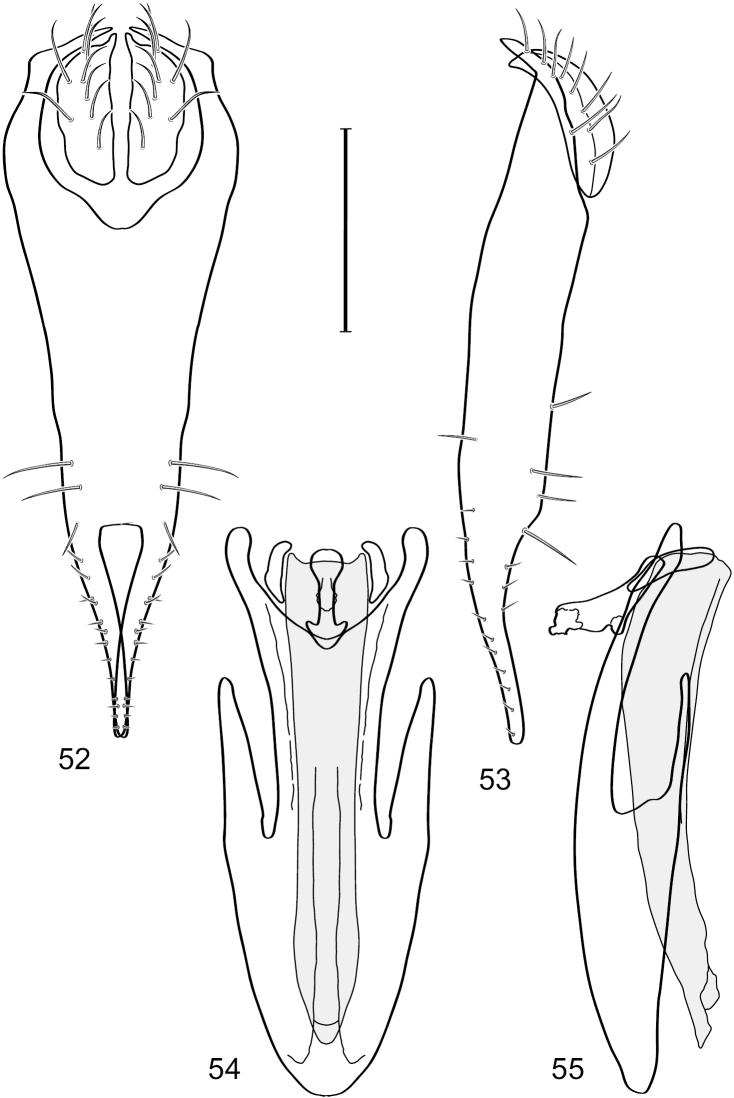
*Hydrochasma viridum* sp. n. (Guyana. Pirara Ranch and River) **52** epandrium and cerci, posterior view **53** same, lateral view **54** internal structures of male terminalia (aedeagus [shaded], phallapodeme, gonite, hypandrium), ventral view **55** same, lateral view. Scale bar = 0.1 mm.

**Figure 56.**
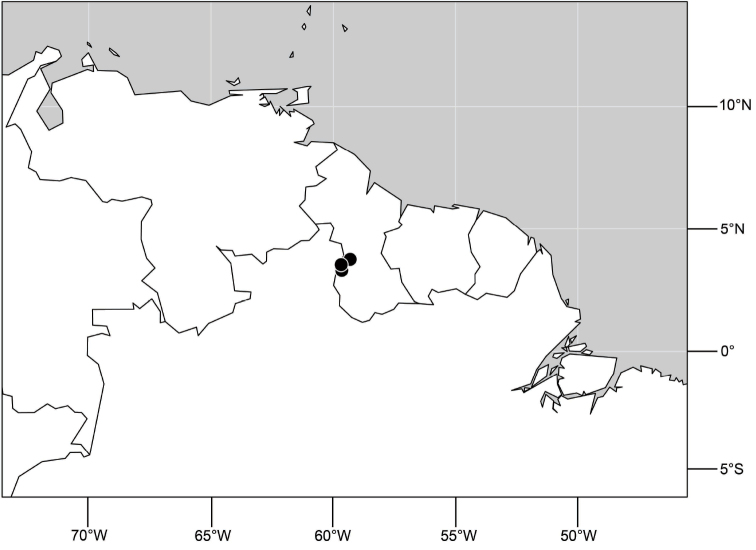
Distribution of *Hydrochasma viridum* sp. n.

This species is distinguished from other congeners by the following combination of characters: Small shore flies, body length 1.25–1.45 mm. *Head*: Antenna mostly blackish gray; medial surface of basal flagellomere mostly dark colored; parafacial silvery white, concolorous with facial coloration (Figs 49–51); gena-to-eye ratio 0.23–0.25. *Thorax*: Mesonotum with extensive metallic green coloration, extended laterally to notopleuron and presutural area. Wing with costal vein ratio 0.80–0.82; M vein ratio 0.46–0.48. Forefemur lacking a distinctive, comb-like row of stout setulae along anteroventral surface; tibiae mostly gray; hindtibia bearing a long, spur-like seta ventroapically. *Abdomen*: Tergites 1–4 greenish gray, subshiny, lacking wedge-shaped, gray to silvery gray areas, tergite 5 of male more gray colored than preceding tergites. Male terminalia (Figs 52–56): Combined structures generally moderately elongate, in posterior view height slightly more than 3× width, generally sparsely setulose dorsally, bearing large to small setulae ventrally; epandrium with dorsal arch above cerci relatively thinly developed, incompletely connected medially, in posterior view (Fig. 52) as an inverted U on dorsal third with disconnect dorsomedially, thereafter ventrally widest sub-basally, then tapered with lateral margins shallowly curved medially, ventral third as very thin, elongate, digitiform, parallel-sided projections, these cruciate subapically, deeply bifurcate medially, medial bifurcation wider basally than width of ventral projections at same level, ventral epandrial projections minutely setulose, apical setulae stout, apex rounded; cerci moderately long, height nearly twice width, widely semi-hemispherical (Fig. 53), not attached lateroventrally with epandrium; aedeagus in lateral view (Fig. 55) elongate, over 5× longer than wide, tubular, shallowly curved, slightly tapered toward apex with apical portion moderately and irregularly pointed, in ventral view (Fig. 54) nearly straight sided, slightly thinner medially than basally or subapically, apical portion tapered to narrowly rounded apex; phallapodeme in lateral view (Fig. 55) generally narrow to very shallowly and broadly triangular, extended keel only a slight bump toward attachment with hypandrium, in ventral view (Fig. 54) an elongate, thin T with crossbar very short; gonite in lateral view (Fig. 55) narrow, elongate, bar-like, very shallowly curved, in ventral view (Fig. 54) shallowly curved, tapered at both ends; hypandrium in lateral view (Fig. 55) elongate, as long or slightly longer than aedeagus, moderately thin, mostly parallel sided, anterior portion evenly tapered to narrowly rounded anterior apex, in ventral view (Fig. 54) generally as a gently curved arrowhead with posterior half generally narrower, posterior margin widely and moderately deeply emarginate with narrow, lateral arms, tapered to anterior portion of base, anterior portion elongate, V-shaped with lateral margins shallowly curved, with elongate, narrow, lateral, posterior extended processes that are aligned parallel to overall orientation of hypandrium, anterior margin tapered to bluntly rounded apex.

##### Type material.

The holotype male of *Hydrochasma viridum* is labeled “**GUYANA.** Karanambo[,] Rupununi Riv[er],ox bow[,] 3°45.1'N, 59°18.6'W[,] 2Apr1994, W. Mathis/HOLOTYPE ♂ *Hydrochasma viridum* Mathis & Zatwarnicki, USNM [red]/USNM ENT 00089395 [plastic bar code label].” The holotype is double mounted (minuten in a block of plastic), is in good condition (some dirt specks on specimen), and is deposited in the USNM. Paratype are as follows: GUYANA. Moco-Moco, Lethem (30 km E in Kanuku Mountains; 03°18.2'N, 59°39.0'W), 3–6 Apr 1994, W. N. Mathis (1♂, 1♀; USNM); Pirara Ranch and River (03°32.1'N, 59°40.5'W), 24–25 Apr 1995, W. N. Mathis (1♂, 1♀; USNM).

##### Type locality.

Guyana. Karanambo, Rupununi River (ox bow; 03°45.1'N, 59°18.6'W).

##### Distribution

(Fig. 56). Neotropical: Guyana.

##### Etymology.

The species epithet, *viridum*, is of Latin derivation, meaning green, and refers to the metallic green coloration of the mesonotum.

##### Remarks.

Externally, the dorsum of the head and mesonotum of this species is unique among congeners, especially species in the *faciale* group, in having a pronounced, subshiny to shiny, with mostly greenish to bluish luster. Internally, this species could only be confused with *Hydrochasma patens*, and indeed, structures of the male terminalia of these two species are similar, although differing in details (compare Figs 26–29 with Figs 52–55).

#### 10. Hydrochasma williamsae sp. n.

http://zoobank.org/01EE2E2E-0913-4C01-8A71-525CEC06E796

http://species-id.net/wiki/Hydrochasma_williamsae

**Figures 57–60.**
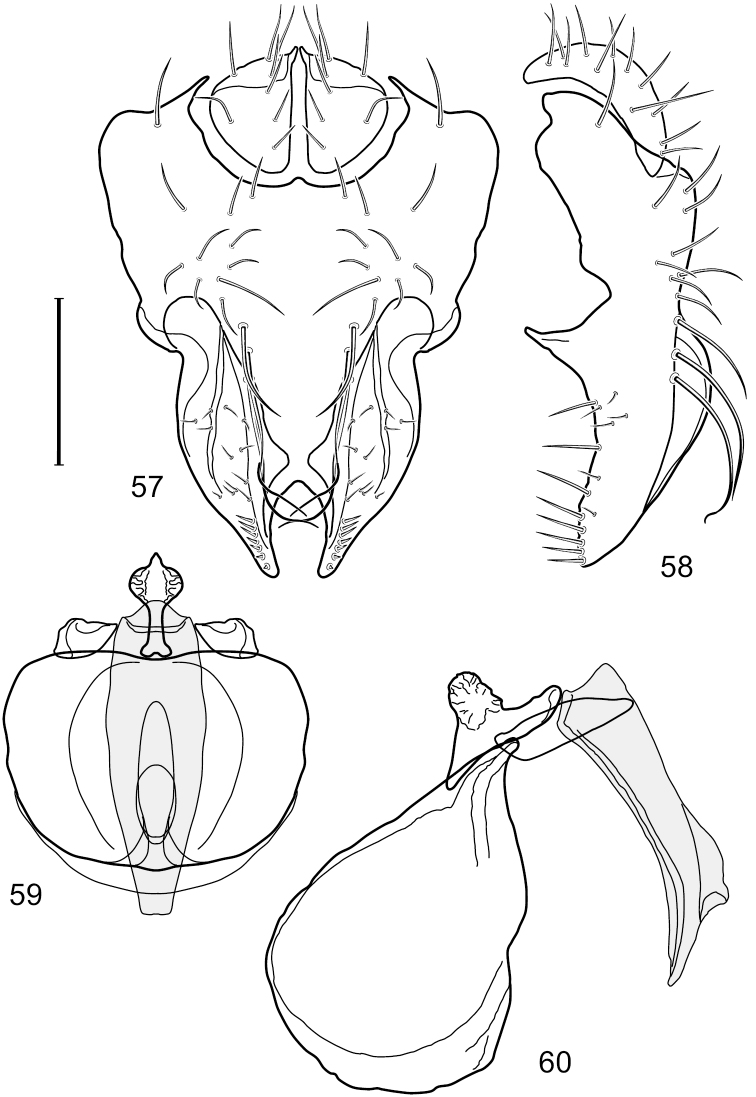
*Hydrochasma williamsae* sp. n. (USA. New Mexico. Grant: Mimbres River) **57** epandrium and cerci, posterior view **58** same, lateral view **59** internal structures of male terminalia (aedeagus [shaded], phallapodeme, gonite, hypandrium), ventral view **60** same, lateral view. Scale bar = 0.1 mm.

**Figure 61.**
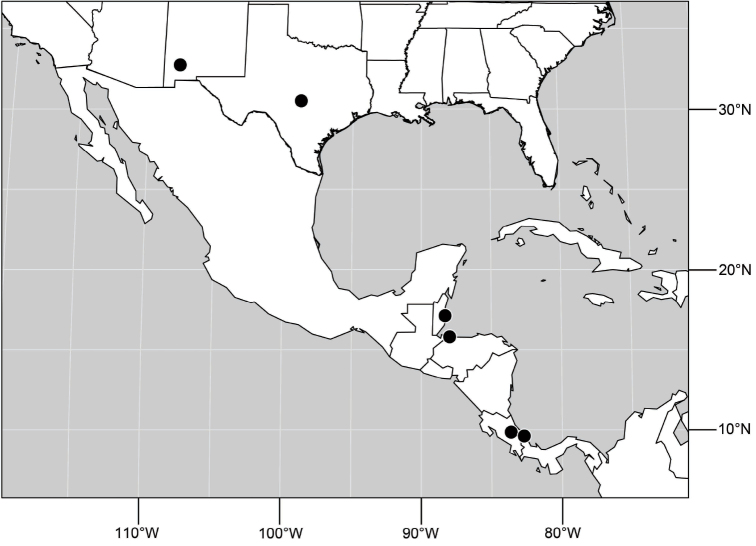
Distribution of *Hydrochasma williamsae* sp. n.

Figs 1 , 11 , 57 –61

##### Diagnosis.

This species is distinguished from congeners by the following combination of characters: Small to moderately small shore flies, body length 1.50–2.30 mm. *Head*: Subglobose, very broad ventrally, oral opening comparatively large. Antennal coloration variable, entirely yellow to nearly evenly divided between yellowish and dark gray, dorsal and anterior surfaces of pedicel and basal flagellomere extensively dark gray. Parafacial silvery white, concolorous with facial coloration; parafacial silvery white, concolorous with face; mesonotum yellowish to golden brown; gena-to-eye ratio 0.25–0.27. *Thorax*: Pleural area gray (Fig. 1). Wing with costal vein ratio 0.63–0.65; M vein ratio 0.52–0.53. Forefemur bearing a distinctive, comb-like row of stout setulae along anteroventral and posteroventral surfaces; tibiae mostly gray; hindtibia bearing a large, spur-like seta ventroapically (Fig. 11). *Abdomen*: Tergites 1–4 with broad, medial brown stripe, uneven lateral margin, lacking wedge-shaped, gray to silvery gray areas laterally (Fig. 1); tergite 5 of male truncate apically, mostly gray with faint brown, medial stripe. Male terminalia (Figs 57–60): Combined structures generally moderately elongate and appearing angulate, in posterior view height about 1.3× width, generally setulose, especially medially at midlength but also along ventral margins; epandrium with dorsal arch above cerci attenuated, not connected, in posterior view (Fig. 57) with cercal cavity forming a broad U, with arms of U robustly developed, ventral portion of arms with each lateral half robustly developed, tapered to dorsal apex, apex pointed medially, dorsal portion of epandrium squarish, especially prominent dorsolateral, rounded angles; ventral portion of epandrium ventrad of midlength, shallow, V-shaped notch, as 2 robust, ventral projections that are tapered to a narrow point ventroapically, forming a deeply and narrowly incised medial incision or narrowly U-shaped pocket, in lateral view (Fig. 58) with very irregular anterior and posterior margins, generally robust, apex narrowly pointed; cerci moderately long, height more than twice width, widely semi-hemispherical (Fig. 57), pointed dorsally, in lateral view (Fig. 58) evenly lunate, not attached lateroventrally or ventrally with epandrium; aedeagus in lateral view (Fig. 60) elongate, length slightly more than 3.5× width, tubular, shallowly curved, especially apically, apex narrowly pointed, in ventral view (Fig. 59) elongate, widest sub-basally, thereafter apically tapered to narrowly truncate apex; phallapodeme in lateral view (Fig. 60) irregularly triangular, short, with extended, medial keel short and narrow, not skewed, apex of keel rounded, in ventral view (Fig. 59) a slightly elongate, moderately robust T with keel portion as a symmetrical, lateral bulge; gonite in lateral view (Fig. 60) moderately narrow, moderately elongate, bar-like, nearly straight, end toward aedeagus tapered to point, in ventral view (Fig. 59) small, width only slightly longer than length, tapered medially; hypandrium in lateral view (Fig. 60) like a deep, globularly rounded pocket, wide, tapered to point posteriorly, anterior margin broadly rounded, in ventral view (Fig. 59) very broadly and robustly developed, width only slightly longer than length, posterior margin very shallowly emarginated, anterior margin broadly rounded.

##### Type material.

The holotype male of *Hydrochasma williamsae* is labeled “**BELIZE**. Stann Cr[ee]k. Dist[rict]. MullinsRiver[,] (17km N Dangriga)[,] 29 March 1988, Wayne N. Mathis/HOLOTYPE ♂ *Hydrochasma williamsae* Mathis & Zatwarnicki, USNM [red]/USNM ENT 00094378 [plastic bar code label].” The holotype is double mounted (minuten in a block of plastic), is in excellent condition, and is deposited in the USNM. Sixteen paratypes (5♂, 11♀; USNM) bear the same label data as the holotype.

##### Type locality.

Belize. Stann Creek: Mullins River (17 km N Dangriga; 17°06.2'N, 88°17.8'W).

##### Other specimens examined.

Nearctic: UNITED STATES. NEW MEXICO. **Grant:** Mimbres River (32°43.8'N, 107°52'W; 1665 m), 13-22 Aug 2007, 2009, D. and W. N. Mathis, T. Zatwarnicki (2♂; USNM).

TEXAS. **Llano:** Enchanted Rock (30°30.4'N, 98°49.1'W), 15 Jun 1953, W. W. Wirth (1♂; USNM).

Neotropical. COSTA RICA. **Cartago:** La Suiza (09°51.5'N, 83°37.5'W), 28 Jun 2001, W. N. Mathis (2♂, 4♀; USNM). **Limón:** Talamanca (Estación Gandoca; 9°37.4'N, 82°41.7'W), 18 May 2004, W. Porras (1♂; INBIO).

HONDURAS. **Cortés:** Omoa (16°47.8'N, 87°58.4'W), 26 Sep 1995, D. and W. N. Mathis (1♂, 1♀; USNM).

##### Distribution

(Fig. 61). Nearctic: United States (New Mexico, Texas). Neotropical: Belize (Stann Creek), Costa Rica (Cartago, Limón), Honduras (Cortés).

##### Etymology.

The specific epithet, *williamsae*, is a genitive patronym to honor our colleague and cherished friend, the late Ms Hollis Barton Williams, who provided technical support to us for nearly 35 years. Holly passed away August 23, 2009.

##### Remarks.

This species, as noted previously in the “Remarks” section of *Hydrochasma faciale*, is distinguished by structures of the male terminalia, especially the generally short and robust epandrium in posterior and lateral views. The more angulate dorsolateral shoulders (best seen in posterior view) and the tapered and pointed ventral epandrial process are unique to this species and distinguish it from congeners.

### The *incisum* Group

**Species included:** *Hydrochasma denticum* sp. n., *Hydrochasma distinctum* sp. n., *Hydrochasma dolabrutum* sp. n., *Hydrochasma falcatum* sp. n., *Hydrochasma glochium* sp. n., *Hydrochasma incisum* (Coquillett), *Hydrochasma kaieteur* sp. n., *Hydrochasma miguelito* sp. n., *Hydrochasma octogonum* sp. n., *Hydrochasma parallelum* sp. n., *Hydrochasma peniculum* sp. n., *Hydrochasma simplicum* sp. n., *Hydrochasma urnulum* sp. n.

**Diagnosis.** This species group is distinguished from others within *Hydrochasma* by the following combination of characters: Head: Oral opening small, not gaping. Thorax: Hindtibia lacking a prominent, spur-like, ventral, subapical seta. Abdomen: Tergites with at least 1 lateral wedge; lacking a sharply demarcated, straight lateral line. Male terminalia: Ventral epandrial extensions usually somewhat narrow and parallel-sided, at least at base; ventral margin of epandrium variable, if bifurcate, gap narrow and relatively shallow.

#### Key to species of the *incisum* group

**Table:** 

1	Hindtibia with apicoventral seta	2
–	Hindtibia lacking apicoventral seta	3
2	Hindtibia with apicoventral seta large, spur-like	*Hydrochasma falcatum* sp. n.
–	Hindtibia with apicoventral seta tiny, setula-like	*Hydrochasma urnulum* sp. n.
3	Ventral epandrial extensions bearing subapical tooth-like setae	4
–	Ventral epandrial extension not bearing tooth-like setae	5
4	Ventral epandrial extensions subequal or as long as quadrate-like base surrounding cerci (Fig. 65)	*Hydrochasma denticum* sp. n.
–	Ventral epandrial extensions about half length as quadrate-like base surrounding cerci (Fig. 72)	*Hydrochasma distinctum* sp. n.
5	Apical portion of ventral epandrial extensions with lateral extensions of various shapes	6
–	Apical portion of ventral epandrial extensions essentially parallel sided	11
6	Apical portion of ventral epandrial extensions with lateral projections wider at base, narrowed toward apex, arrow-like	7
–	Apical portion of ventral epandrial extensions without arrow-like lateral extensions	9
7	Width of lateral projections of ventral epandrial extensions greater than length (Fig. 101)	*Hydrochasma miguelito* sp. n.
–	Width of lateral projections of ventral epandrial extensions subequal to length	8
8	Lateral projections of ventral epandrial extensions pointed (Fig. 86); hypandrium in ventral view (Fig. 88) V-shaped	*Hydrochasma glochium* sp. n.
–	Lateral projections of ventral epandrial extensions not pointed (Fig. 81); hypandrium in ventral view (Fig. 83) robustly U-shaped	*Hydrochasma falcatum* sp. n.
9	Lateral projections of ventral epandrial extensions and extensions phallic-like with hood-like apex (Fig. 115)	*Hydrochasma peniculum* sp. n.
–	Lateral projections of ventral epandrial extensions and extensions not phallic-like with hood-like apex	10
10	Lateral projections of ventral epandrial extensions forming a rectangular apex (Fig. 127)	*Hydrochasma urnulum* sp. n.
–	Lateral projections of ventral epandrial extensions simply flared ventrolaterally, not forming a rectangular apex (Fig. 91)	*Hydrochasma incisum* (Coquillett)
11	Ventral epandrial extensions parallel sided throughout length with rounded apex (Fig. 111)	*Hydrochasma parallelum* sp. n.
–	Ventral epandrial extensions not parallel sided throughout length or if nearly parallel sided, then with broadly emarginate apex	12
12	Apex of ventral epandrial extension in lateral view with angulate, anterior projection (Fig. 77)	*Hydrochasma dolabrutum* sp. n.
–	Apex of ventral epandrial extension in lateral view tapered, lacking anterior projection	13
13	Lateral margins of epandrium in posterior view distinctly angulate at midheight, then parallel sided, then tapered to apex (Fig. 106)	*Hydrochasma octogonum*, n. sp.
–	Lateral margins of epandrium in posterior view rounded or very shallowly angulate	14
14	Ventral epandrial extensions in lateral view pointed apically (Fig. 97)	*Hydrochasma kaieteur* sp. n.
–	Ventral epandrial extensions in lateral view rounded (Fig. 121)	*Hydrochasma simplicum* sp. n.

#### 11. Hydrochasma denticum sp. n.

http://zoobank.org/67363C11-5AA7-4505-B987-0FD186FCB6B8

http://species-id.net/wiki/Hydrochasma_denticum

Figs 62 – 69 , 71

##### Diagnosis.

**Figures 62–64.**
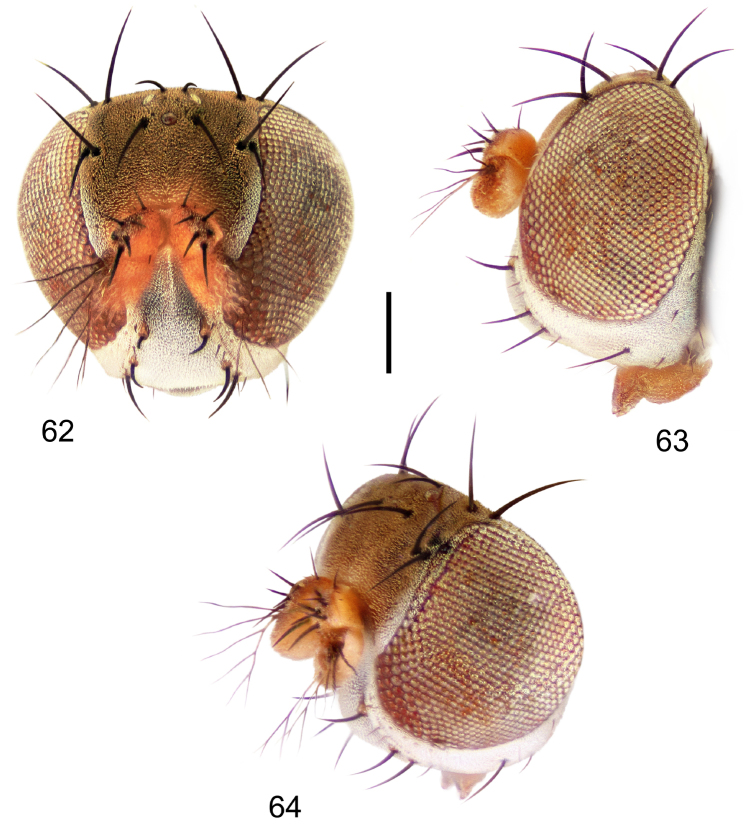
*Hydrochasma denticum* sp. n. (Ecuador. Orellana: Río Tiputini) **62** head, anterior view **63** same, lateral view **64** same, oblique view. Scale bar = 0.1 mm.

**Figures 65–68.**
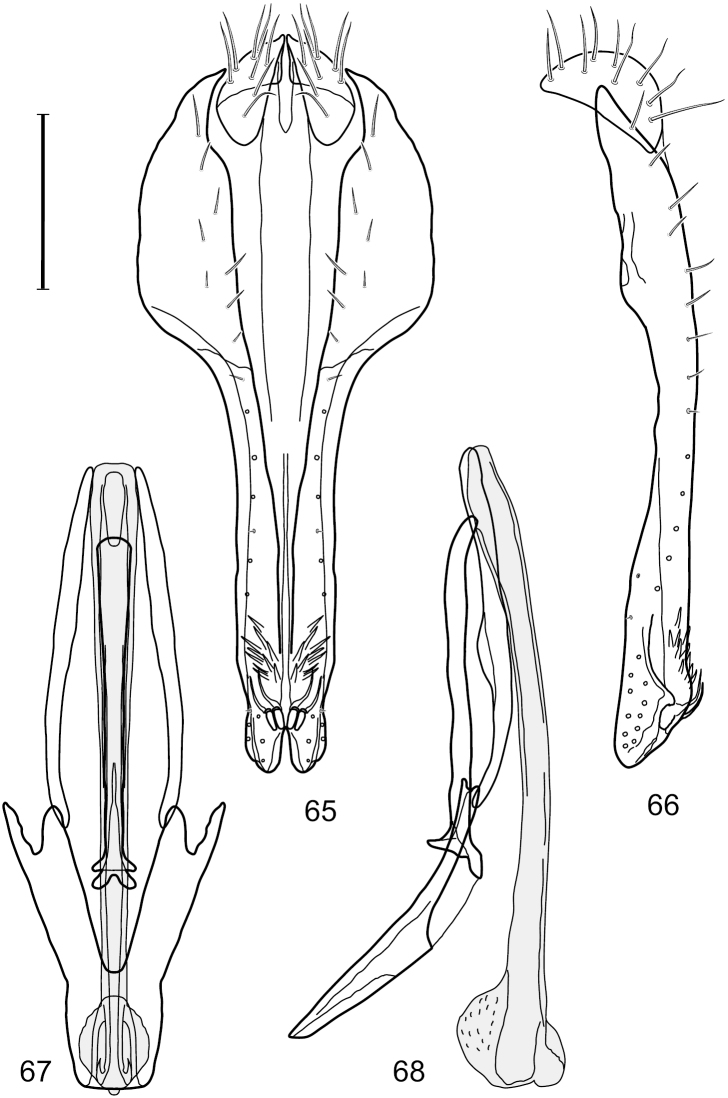
*Hydrochasma denticum* sp. n. (Ecuador. Orellana: Río Tiputini) **65** epandrium and cerci, posterior view **66** same, lateral view **67** internal structures of male terminalia (aedeagus [shaded], phallapodeme, gonite, hypandrium), ventral view **68** same, lateral view. Scale bar = 0.1 mm.

This species is distinguished from congeners by the following combination of characters: Small shore flies, body length 1.15–1.70 mm. *Head*: Antenna mostly dark gray; parafacial silvery white, concolorous with facial coloration (Figs 62–64); gena-to-eye ratio 0.0.19–0.21. *Thorax*: Wing with costal vein ratio 0.68–0.71; M vein ratio 0.54–0.56. Forecoxa whitish gray, yellowish at apices; forefemur lacking a distinctive, comb-like row of stout setulae along anteroventral surface; tibiae mostly gray. *Abdomen*: Tergites 2–3with wedge-shaped silvery-gray areas on slate black dorsum (Fig. 71); tergite 5 of male mostly gray with blackish posterior margin. Male terminalia (Figs 65–68): Combined structures generally moderately elongate, in posterior view height 2.25× width, generally sparsely setulose dorsally, setulae sparse or lacking ventrally; epandrium lacking dorsal arch above cerci, in posterior view (Fig. 65) with apical 1/2–2/3 abruptly narrowed, parallel sided, apical process not wider than apical 1/2, apex with very narrow apicomedial cleft, in lateral view (Fig. 66) very elongate, narrow with basal 3/4 straight, apical 1/3 becoming slightly wider, slightly expanded, apex narrowly rounded, with paired subapical tooth-like structures on each process; aedeagus in lateral view (Fig. 68) very elongate and very narrow, mostly parallel sided, apical 1/8 expanded anteriorly and to a less degree posteriorly, apex irregularly rounded, in ventral view (Fig. 67) very narrow and elongate, apical 1/8 bulbously expanded, apex broadly V-shaped; phallapodeme in lateral view (Fig. 68) very narrow and elongate, rod-like, hypandrial end with narrowly pointed keel, aedeagal end very shallowly curved, in ventral view (Fig. 67) elongate, narrow, truncate, slightly and gradually expanded at aedeagal end, hypandrial end with 2 narrow crossbars; gonite in lateral view (Fig. 68) as a very shallowly curved, rod-like process, about equal in length to phallapodeme, in ventral view (Fig. 67) shallowly curved to straight, tapered at both apices; hypandrium in lateral view (Fig. 68) narrowly developed, anterior half narrowly angled, gradually expanded toward midlength, thereafter narrowed to parallel-sided, elongate extension, in ventral view (Fig. 67) moderately deeply and thickly V-shaped, with base of V rectangular, robustly developed, extended lateral arms thick, elongate, each narrowly cleft apically, bifurcate.

##### Type material.

The holotype male of *Hydrochasma denticum* is labeled “**ECUADOR.** Prt. Or[e][l]lana: RioTiputini(0°38.2'S, 76°8.9'W), 12–26 Aug 1999, W.N.Mathis, A. Baptista, M. Kotrba/USNM ENT 00117964 [plastic bar code label]/HOLOTYPE ♂ *Hydrochasma denticum* Mathis & Zatwarnicki, USNM [red].” The holotype is double mounted (minuten in a block of plastic), is in excellent condition, and is deposited in the USNM. Seventeen paratypes (15♂, 2♀; USNM) bear the same label data as the holotype.

##### Type locality.

Ecuador. Orellana: Río Tiputini (0°38.2'S, 76°8.9'W).

##### Other specimens examined.

Neotropical. BOLIVIA. **El Beni:** Cavinas (12°31'S, 66°49'W), Jan 1922, W. M. Mann (2♀; USNM); Huachi (14°13.8'S, 63°32.1'W), 21 Sep, W. M. Mann (13♂, 1♀; ANSP, USNM). **La Paz:** Guanay (15°29.8'S, 67°52.7'W), 460 m), 13 Mar 2001, W. N. Mathis (2♂; USNM).

HONDURAS. **Cortés:** San Pedro Sula (8 km S; 15°25.7'N, 88°01.4'W), 25–26 Sep 1995, D. and W. N. Mathis (1♂; USNM).

PERU. **Madre de Dios:** Río Manu, Pakitza (11°56.6'S, 71°16.9'W; 250 m), 9 Sep 1988, W. N. Mathis (10♂, 15♀; USNM).

##### Distribution

**Figure 69.**
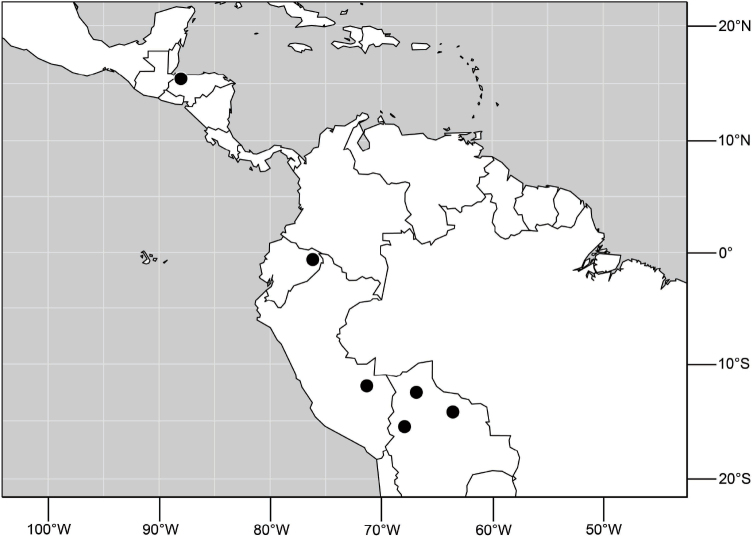
Distribution of *Hydrochasma denticum* sp. n.

(Fig. 69). Neotropical: Bolivia (El Beni, La Paz), Ecuador (Orellana), Honduras (Cortés), Peru (Madre de Dios).

**Figures 70–71.**
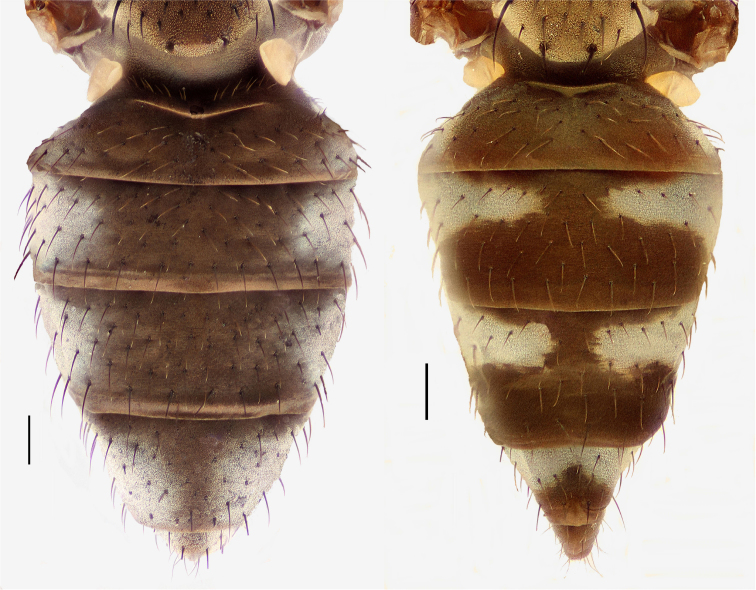
Abdomens of females, dorsal view. **70** *Hydrochasma spinosum* sp. n. (Costa Rica. Puntarenas: Dominical) **71** *Hydrochasma denticum* sp. n. (Ecuador. Orellana: Río Tiputini). Scale bar = 0.1 mm.

##### Etymology.

The species epithet, *denticum*, is of Latin derivation and means tooth, referring to the tooth-like subapical structures toward the apex of the extended epandrium.

##### Remarks.

As implied by its species’ name, *denticum*, this species is distinguished from congeners by the paired, tooth-like structures located subapically on each of the ventral epandrial processes. In addition, the hypandrium in ventral view is V-shaped with robust, posterior arms that in turn are bifurcate apically.

#### 12. Hydrochasma distinctum sp. n.

http://zoobank.org/4A1D6F9C-A2C5-456A-9EE9-74BF61C84718

http://species-id.net/wiki/Hydrochasma_distinctum

Figs 72 –76

##### Diagnosis.

**Figures 72–75.**
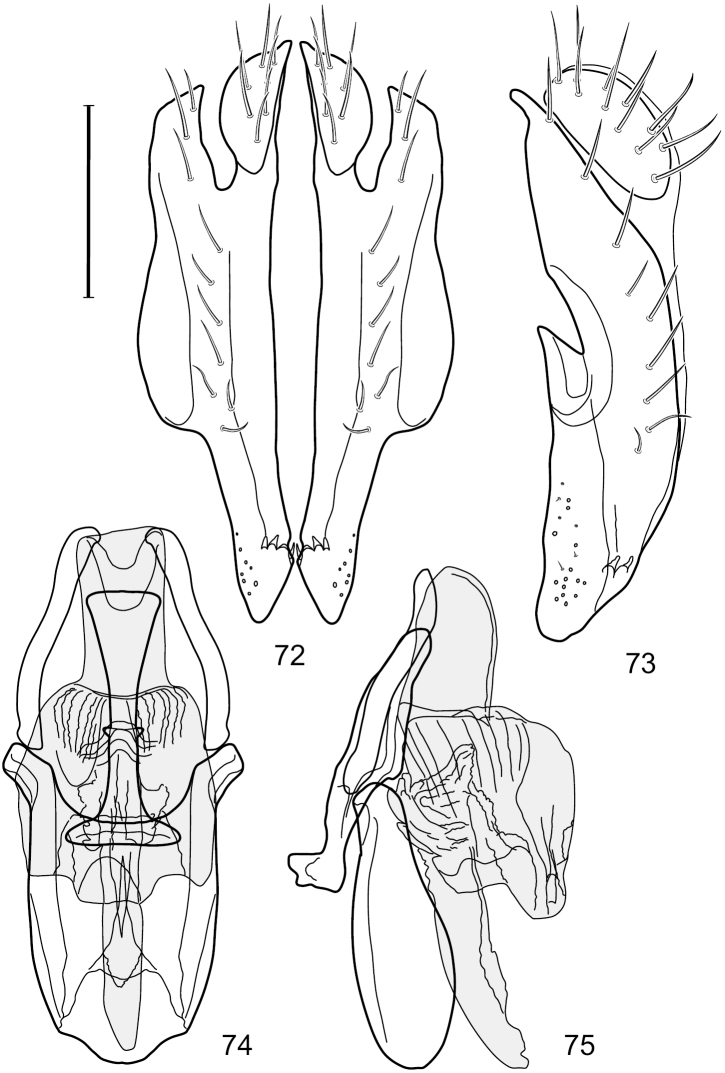
*Hydrochasma distinctum* sp. n. (Costa Rica. Limón: Parque Nacional Barbilla, Sector Casas Negras) **72** epandrium and cerci, posterior view **73** same, lateral view **74** internal structures of male terminalia (aedeagus [shaded], phallapodeme, gonite, hypandrium), ventral view **75** same, lateral view. Scale bar = 0.1 mm.

This species is distinguished from congeners by the following combination of characters: Moderately small shore flies, body length 2.20 mm. *Head*: Antenna mostly dark gray; parafacial silvery white, concolorous with facial coloration; gena-to-eye ratio 0.13. *Thorax*: Wing with costal vein ratio 0.82; M vein ratio 0.51. Forefemur lacking a distinctive, comb-like row of stout setulae along anteroventral surface; tibiae mostly gray. *Abdomen*: Tergites 3 and 4 with wedge-shaped silvery-gray areas; otherwise, tergites dark brown to black. Male terminalia (Figs 72–75): Combined structures generally moderately compact and wide, in posterior view height 1.8× width, generally moderately setulose dorsally, setulae sparse or lacking ventrally; epandrium lacking dorsal arch above cerci, in posterior view (Fig. 72) with basal 2/3 rectangular, lateral margins of basal portion shallowly sinuous, ventral epandrial process conspicuously narrowed, tapered, each process digitiform, bifurcate apically with inverted V-shaped gap between, in lateral view (Fig. 73) much higher than wide, posterior margin arched, anterior margin with a midlength, shallow but elongate concavity, apex obtusely rounded; each ventral epandrial process with 2–3 dentate structures subapically; aedeagus in lateral view (Fig. 75) basically elongate, slender, tubular, tapered toward apex but with a large, somewhat quadrate structure at midlength, in ventral view (Fig. 74) elongate, slender, tubular, with quadrate structure at midlength, quadrate structure with basal margin shallow emarginate, apical margin with wide, shallow projection, truncate apically; phallapodeme in lateral view (Fig. 75) elongate, narrow, shallowly sinuous, keel as irregular, apical knob, in ventral view (Fig. 74) elongate, narrow, T-shaped, truncate at each end, basal half gradually expanded; gonite in lateral view (Fig. 75) a very shallowly sinuous, rod-like process, about 3/4 length of phallapodeme, in ventral view (Fig. 74) shallowly sinuous, bluntly rounded at both apices; hypandrium in lateral view (Fig. 75) narrowly developed, generally narrowly ovate, anterior margin moderately narrowly rounded, gradually tapered toward posterior margin, in ventral view (Fig. 74) longer than wide, more sclerotized and darker laterally, posterior margin widely emarginate, anterior margin membranous.

##### Type material.

The holotype male of *Hydrochasma distinctum* is labeled “COSTA RICA. Prov. Limón. P[arque]. N[acional]. Barbilla, Sector Casas Negra, 1.5 Km NO[RTH] de la Estación, 300m, 13 JUL 2002, E. Rojas, Libre, L_N_ 219900_598400 #70494/INB0003513948 INBIOCRI COSTA RICA [plastic bar code label]/HOLOTYPE ♂ *Hydrochasma distinctum* Mathis & Zatwarnicki, USNM [red].” The holotype is double mounted (minuten in a block of plastic), is in good condition (some setae of the head are misoriented or missing; abdomen removed and dissected), and is deposited in INBio.

##### Type locality.

Costa Rica. Limón: Parque Nacional Barbilla, Sector Casas Negras, (10°0.8'N, 83°28.1'W; 300 m).

##### Distribution

**Figure 76.**
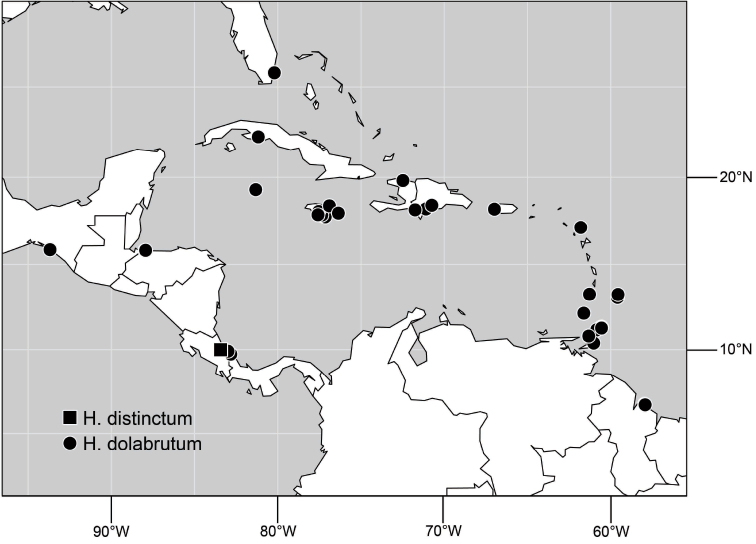
Distribution of *Hydrochasma distinctum* sp. n. and *Hydrochasma dolabrutum* sp. n.

(Fig. 76). Neotropical: Costa Rica (Limón).

##### Etymology.

The species epithet, *distinctum*, is of Latin derivation and means separate or different, referring to the unique and distinct structures of the male terminalia.

##### Remarks.

Thus far, this species is known only by the holotype male. As implied by its species’ name, *distinctum*, this species is readily distinguished from congeners by the distinctive structures of the male terminalia, which are unique among congeners. The shape of the epandrium with a somewhat large and rectangular base and the short, tapered, ventral epandrial processes that bear tooth-like structures subapically are unique, as is the aedeagus that has a relatively large, quadrate structure at its midlength. Only *Hydrochasma denticum*, which also has subapical tooth-like structures on the ventral epandrial processes, is similar. The ventral epandrial processes of *Hydrochasma denticum*, however, are much longer than their base, the aedeagus does not have a quadrate structure at its midlength, and the hypandrium is V-shaped.

#### 13. Hydrochasma dolabrutum sp. n.

http://zoobank.org/15AF0B2A-A060-4D62-A830-43823AB95C0E

http://species-id.net/wiki/Hydrochasma_dolabrutum

Figs 77–80 , 142

##### Diagnosis.

**Figures 77–80.**
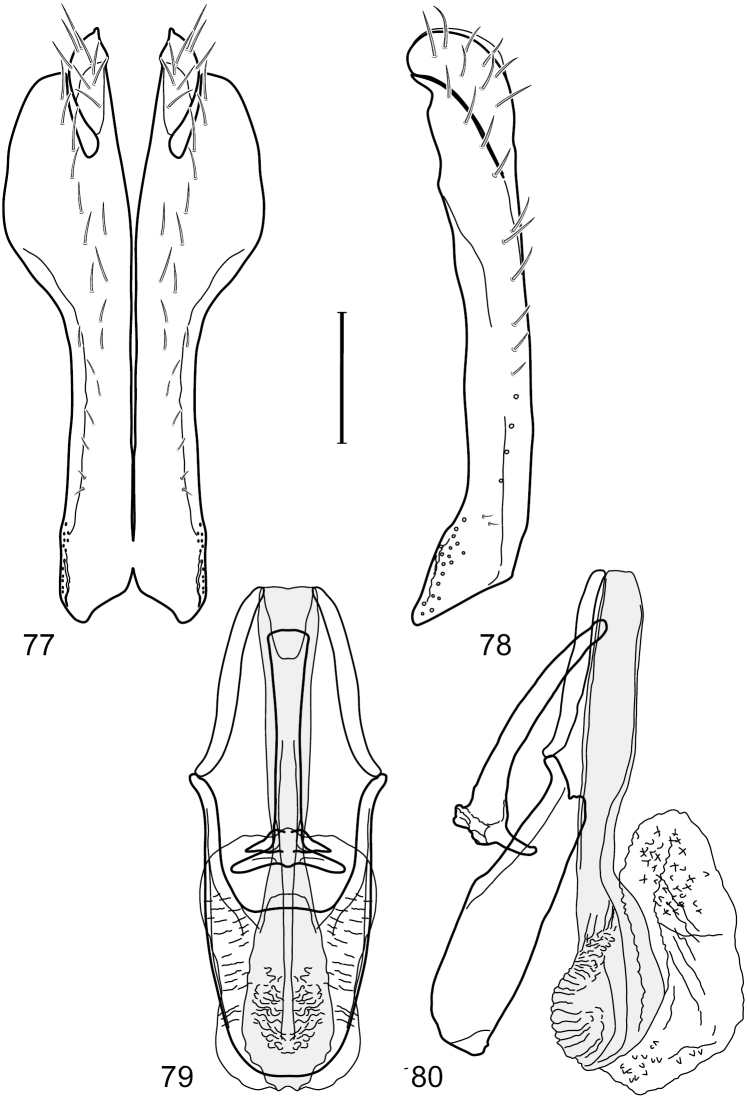
*Hydrochasma dolabrutum* sp. n. (Jamaica. Clarendon: Barnswell Beach) **77** epandrium and cerci, posterior view **78** same, lateral view **79** internal structures of male terminalia (aedeagus [shaded], phallapodeme, gonite, hypandrium), ventral view **80** same, lateral view. Scale bar = 0.1 mm.

This species is distinguished from congeners by the following combination of characters: Small shore flies, body length 1.65–1.90 mm. *Head*: Antenna mostly dark gray; parafacial silvery white, concolorous with facial coloration; gena-to-eye ratio 0.21–0.23. *Thorax*: Wing with costal vein ratio 0.69–0.71; M vein ratio 0.49–0.52. Forecoxa yellowish to tan; forefemur lacking a distinctive, comb-like row of stout setulae along anteroventral surface; tibiae mostly gray. *Abdomen*: Tergites 3–4 with distinct, deep, wedge-like marking laterally but tergites 2–4 with wide medial area extensively dark slate gray to black; tergite 5 light gray to silvery gray with undifferentiated posterior margin, uniformly colored (Fig. 142); medial coloration on tergites 1–4 narrow, sometimes only a stripe, slightly darker than color of lateral margins. Male terminalia (Figs 77–80): Combined structures generally elongate, in posterior view height nearly 2.5× width; epandrium (Fig. 77) with dorsal arch above cerci attenuate, not connected, dorsal 1/3–1/2 somewhat quadrate with angles rounded, ventral portion as 2 digitiform, thick, abutting, parallel lobes that connect subapically for a short distance, ventral margin concave with a medial, narrow incision, dorsal half bearing longer setulae than ventrally, ventral setulae lacking on apical portion, in lateral view (Fig. 78) mostly parallel sided and nearly straight, apical 1/8 angled anteroventrally as a tapered, narrowly pointed apex; cerci relatively very short, in lateral view (Fig. 77) height about twice width, narrowly semicircular, attached to epandrium ventrally; aedeagus in lateral view (Fig. 80) elongate, with length of sclerotized portion about 8× width, basal portion parallel sided, apical half greatly enlarged, bulbous, membranous, with apex broad, membrane bearing sub-basally numerous scale-like spicules, in ventral view (Fig. 79) also showing expanded apex, basal 1/2 comparatively narrowed, slightly wider at base, thereafter slightly tapered to expanded, broadly obovate apical half; phallapodeme in lateral view (Fig. 80) narrow, very elongate, evenly shallowly arched, keel very weakly developed, barely evident at end toward attachment with hypandrium, in ventral view (Fig. 79) with hypandrial end T-shaped with 2 crossbars, thereafter toward base of aedeagus gradually expanded to blunt apex; gonite in lateral view (Fig. 80) narrow, elongate, bar-like with slight, sinuous curvature, in ventral view (Fig. 79) moderately narrow, elongate, rod-like, apices narrowed; hypandrium in lateral view (Fig. 80) elongate, roughly rectangular, moderately shallow, width slightly more than 1/4 length, very slightly angulate, in ventral view (Fig. 79) generally U-shaped with base very thick, posterior margin deeply U-shaped, anterior margin moderately narrowly rounded.

##### Type material.

The holotype male of *Hydrochasma dolabrutum* is labeled “**DOM[INICAN]. REP[UBLIC].** Barahona: Barahona (18°12'N, 71°5.3'W), 20 May 1998, D. and W. N. Mathis/USNM ENT 00087042 [plastic bar code label]/HOLOTYPE ♂ *Hydrochasma dolabrutum* Mathis & Zatwarnicki, USNM [red].” The holotype is double mounted (minuten in a block of plastic), is in excellent condition, and is deposited in the USNM. Nine paratypes (7♂, 2♀; USNM) bear the same label data as the holotype. Other paratypes are as follows: DOMINICAN REPUBLIC. **Azua:** near Pueblo Viejo (18°24.8'N, 70°44.7'W), 19 May 1998, D. and W. N. Mathis (6♂, 6♀; USNM). **Barahona:** Barahona (18°12'N, 71°5.3'W), 25 Mar 1999, W. N. Mathis (1♂; USNM). **Pedernales:** Pedernales (19 km N; 18°09.2'N, 71°44.8'W; 230 m), 20 Mar 1999, W. N. Mathis (4♂; USNM).

##### Type locality.

Dominican Republic. Barahona: Barahona (18°12'N, 71°5.3'W).

##### Other specimens examined.

Nearctic. UNITED STATES. FLORIDA. **Miami-Dade:** Miami (25°46'N, 80°12'W), 20 Apr 1930, A. L. Melander (2♂; ANSP).

Neotropical. COSTA RICA. **Limón:** R. V. S. Gandoca-Manzanillo, desembocadura de Laguna Gandoca (09°35.4'N, 82°35.8'W), 18 May 2004, D. Briceño (1♂, 3♀; INBIO); Parque Nacional Cahuita, Sector Puerto Vargas (09°44'N, 82°49'W; 1 m), 27–28 Jun 2001, J. D. Gutierrez (1♂; INBio); Puerto Vargas (09°43.9'N, 82°48.9'W; beach), 28 Jun 2001, W. N. Mathis (1♂; USNM); Westfalia (4 km S; 9°54.5'N, 82°59'W; beach), 27 Jun 2001, A. Freidberg, W. N. Mathis (10♂, 5♀; USNM).

GUYANA. Hope Beach (06°44.7'N, 57°57.3'W), 20 Aug 1997, W. N. Mathis (1♂; USNM); Mahaica (6°42.8'N, 57°55.6'W), 22 Apr 1995, W. N. Mathis (1♂, 2♀; USNM).

HONDURAS. **Cortés:** Omoa (16°47.8'N, 87°58.4'W), 26 Sep 1995, D. and W. N. Mathis (2♂; USNM); Puerto Cortés/Omoa (15°49'N, 87°56.2'W), 26 Sep 1995, D. and W. N. Mathis (6♂, 3♀; USNM).

MEXICO. **Chiapas:** Boca de Cielo (17 km S Puerto Arista; 15°51.1'N, 93°40.4'W), 18 May 1985, A. Freidberg, W. N. Mathis (6♂, 4♀; USNM).

TRINIDAD and TOBAGO. Tobago. **St. John:** Charlotteville (beach; 11°19.5'N, 60°32.9'W), 16–18 Apr 1994, D. and W. N. Mathis (3♂; USNM); Charlotteville (5 km S; Hermitage River and beach; 11°18.9'N, 60°34.5'W), 22 Apr-11 Jun 1993, 1994, D. and W. N. Mathis (1♂, 2♀; USNM); Speyside (Doctor River; 11°18.2'N, 60°31'W), 19 Apr 1994, D. and W. N. Mathis (1♂; USNM). **St. Patrick:** Pigeon Point (beach; 11°9.7'N, 60°50'W), 19 Apr 1994, D. and W. N. Mathis (6♂, 2♀; USNM). **St. Paul:** Delaford, Kings Bay (11°16'N, 60°32.8'W), 13 Jun 1993, W. N. Mathis (2♂, 2♀; USNM). Trinidad. **St. Andrew:** Lower Manzanilla (14 km S; 10°23'N, 61°01'W), mouth of Nariva River, 20 Jun 1993, W. N. Mathis (1♂; USNM). **St. George:** Blanchisseuse (beach; 10°48'N, 61°19'W), 25 Jun 1993, W. N. Mathis (3♂; USNM).

West Indies: ANTIGUA. near airport (17°08.2'N, 61°47.6'W), 19 Mar 1989, W. N. Mathis (6♂, 4♀; USNM).

BARBADOS. **Christ Church:** Rockley Beach (13°04.3'N, 59°35.2'W), 21 May-11 Sep 1996, 1997, D. and W. N. Mathis, H. B. Williams (1♂, 1♀; USNM). **St. Andrew:** Long Pond (13°15.1'N, 59°33.3'W), 10–11 Sep 1996, W. N. Mathis (12♀; USNM).

CUBA. **Matanzas:** Playa Larga (22°15.9'N, 81°09.9'W), 1 May 1983, W. N. Mathis (4♂, 18♀; USNM).

GRAND CAYMAN. Spotts (2 km N; 19°18'N, 81°19'W), 20 Feb 1993, F. J. Burton, W. E. Steiner, J. M. Swearingen (3♂, 1♀; USNM).

GRENADA. **St. Andrew:** Pearls Airport (12°08.7'N, 61°36.6'W), 17 Sep 1996, W. N. Mathis (2♂, 3♀; USNM).

HAITI. Baie de Chouchou (19°49'N, 72°28.5'W), 8 Jun 1978, L. Raccurtt, R. Lowrie (2♂, 1♀; USNM).

JAMAICA. **Clarendon:** Barnswell Beach (17°45.'N, 77°08.5'W), 13 May 1996, D. and W. N. Mathis, H. B. Williams (1♀; USNM); Farquhars Beach (17°50.9'N, 77°22.8'W), 9 May 1996, D. and W. N. Mathis, H. B. Williams (1♂, 4♀; USNM); Milk River Bath (17°51'N, 77°22'W; mangroves), 11 Mar 1970, T. Farr, W. W. Wirth (2♂, 11♀; USNM). **Manchester:** Mandeville (18°03.5'N, 77°31.9'W), 7–13 May 1996, D. and W. N. Mathis, H. B. Williams (4♀; USNM). **St. Elizabeth:** near Port Kaiser (17°52.3'N, 77°34.9'W), 8 May 1996, D. and W. N. Mathis, H. B. Williams (1♀; USNM). **St. Mary:** Port Maria (1 km W; 18°22.8'N, 76°53.6'W), 18 May 1996, D. and W. N. Mathis, H. B. Williams (2♂, 1♀; USNM). **St. Thomas:** Bath Fountain Spring (17°57.6'N, 76°21.3'W), 15 May 1996, D. and W. N. Mathis, H. B. Williams (1♂, 1♀; USNM).

PUERTO RICO. Maricao (18°11.1'N, 66°58.9'W), 21 Sep 1995, D. and W. N. Mathis (1♂; USNM).

ST. VINCENT. **St. Patrick:** Cumberland Bay (13°16'N, 61°15.6'W), 8–10 Jun 1991, D. and W. N. Mathis (1♂; USNM).

##### Distribution

(Fig. 76). United States (Florida). Neotropical: Costa Rica (Limón), Guyana, Honduras (Cortés), Mexico (Chiapas), Trinidad and Tobago, West Indies (Antigua, Barbados, Cuba, Dominican Republic, Grand Cayman, Haiti, Jamaica, Puerto Rico, St. Vincent).

##### Etymology.

The species epithet, *dolabrutum*, is of Latin derivation and means axe, referring to the shape of the extended epandrium in lateral view.

##### Remarks.

Structures of the male terminalia of this species are similar to those of *Hydrochasma parallelum* but are distinguished from the latter species by having a comparatively more robust extended epandrial process (best seen in posterior view). Moreover, the hypandrium in general is wider, more robustly developed, and the anterior margin is broadly rounded rather than truncate as in *Hydrochasma parallelum*.

#### 14. Hydrochasma falcatum sp. n.

http://zoobank.org/4505C221-1346-4802-B608-DE5A81F24839

http://species-id.net/wiki/Hydrochasma_falcatum

Figs 80 – 85

##### Diagnosis.

**Figures 81–84.**
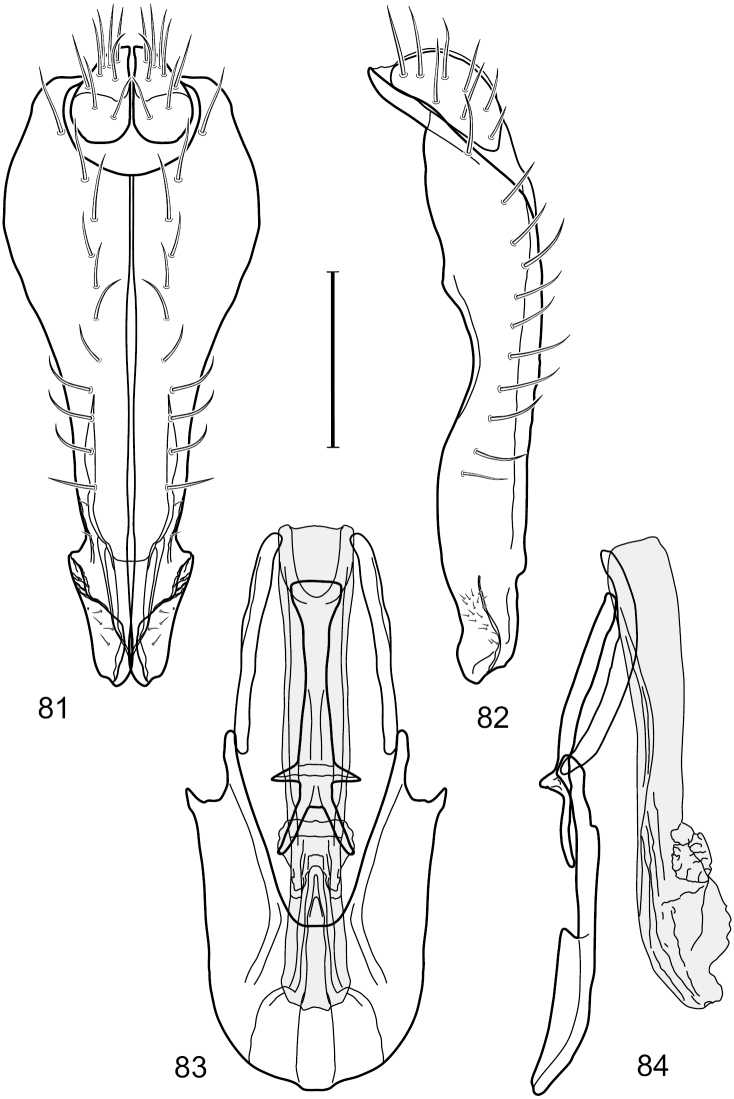
*Hydrochasma falcatum* sp. n. (Peru. Madre de Dios: Río Manu, Erika) **81** epandrium and cerci, posterior view **82** same, lateral view **83** internal structures of male terminalia (aedeagus [shaded], phallapodeme, gonite, hypandrium), ventral view **84** same, lateral view. Scale bar = 0.1 mm.

This species is distinguished from congeners by the following combination of characters: Small to moderately small shore flies, body length 1.25–2.10 mm. *Head*: Antenna mostly dark gray; parafacial silvery white, concolorous with facial coloration; gena-to-eye ratio 0.20–0.22. *Thorax*: Wing with costal vein ratio 0.83–0.85; M vein ratio 0.53–0.54. Forecoxa silvery white; forefemur lacking a distinctive, comb-like row of stout setulae along anteroventral surface; tibiae mostly gray; hindtibia bearing an ventroapical, spur-like seta. *Abdomen*: Tergites 3–4 with distinctive, deep, gray wedge-like marking along lateral margin of darkened, dorsal coloration; tergite 5 of male gray with posteromedial area darkened. Male terminalia (Figs 81–84): Epandrium generally elongate, setulae moderately sparse, on medial portion, in posterior view (Fig. 81) with dorsal arch attenuate, not connected, dorsal 2/3–3/4 somewhat diamond shaped, widest just ventrad of cerci, thereafter ventrally tapered to arrow-shaped apex, arrow-shaped apex with width subequal to length, apex with narrowly incised medially, in lateral view (Fig. 82) narrowly elongate, mostly parallel sided, anterior margin shallowly sinuous, apex somewhat bluntly rounded; cerci short, length in lateral view (Fig. 82) slightly more than twice width, hemispherical; aedeagus in lateral view (Fig. 84) relatively simple, narrowly tubular, length 5× width, in ventral view (Fig. 83) also tubular, narrow, elongate; phallapodeme in lateral view (Fig. 84) linear, very shallowly curved, extended keel narrow and short, as a slight bump, in ventral view (Fig. 83) narrowly Y-shaped with apical arms short and with short, subapical crossbar; gonite in lateral view (Fig. 84) narrowly elongate, bar-like, more curved than phallapodeme, in ventral view (Fig. 83) narrowly bar-like, elongate, nearly straight; hypandrium in lateral view (Fig. 84) elongate, length about 2/3 that of aedeagus, straight, rod-like, slightly expanded on apical half, in ventral view (Fig. 83) with anterior margin broadly truncate, lateral margins nearly parallel sided, posterior margin deeply incised with bifurcate, short, processes sublaterally.

##### Type material.

The holotype male of *Hydrochasma falcatum* is labeled “PERU. Madre de Dios: Manu, Erika (near Salvación; 12°47[six, 50.7]'S, 71°13[six, 23.3]'W; 550 m), 5–6 Sep 1988, W. N. Mathis/USNM ENT 00285976 [plastic bar code label]/HOLOTYPE ♂ *Hydrochasma falcatum* Mathis & Zatwarnicki, USNM [red].” The holotype is double mounted (minuten in a block of plastic), is in excellent condition, and is deposited in the USNM. Twenty-six paratypes (19♂, 7♀; USNM) bear the same label data as the holotype. Other paratypes are as follows: PERU. **Madre de Dios:** Quebrada Romero (near; Rio Manu; 12°07'S, 70°58'W), 8 Sep 1988, W. N. Mathis (1♂, 2♀; USNM); Río Manu, Cocha Salvador (11°59.9'S, 71°13.9'W 300m), 14 Sep 1988, W. N. Mathis (3♂, 10♀; USNM).

##### Type locality.

Peru. Madre de Dios: Río Manu, Erika (near Salvación; 12°50.7'S, 71°23.3'W; 550 m).

##### Other specimens examined.

Neotropical. BOLIVIA. **La Paz:** Guanay (15°29.8'S, 67°52.7'W), 460 m), 13 Mar 2001, W. N. Mathis (3♂, 3♀; USNM); Guanay (3 km E; 15°30.2'S, 67°52.3'W; 500 m), 14 Mar 2001, W. N. Mathis (1♂; USNM); Puente Villa (17 km W; 16°20.9'S, 67°49'W; 2070 m), 11 Mar 2001, W. N. Mathis (1♂, 1♀; USNM); San Pedro (3 km NE; 16°S, 67°35.3'W; 780 m), 12 Mar 2001, W. N. Mathis (12♂, 4♀; USNM).

BRAZIL. **Amazonas:** Igarapé Cabeça Branca (ca. 40 km N Manaus; 02°35.1'S, 60°01.9'W; 65 m), 8 May 2010, D. and W. N. Mathis (3♂; USNM).

ECUADOR. Loja: **Loja:** Catamayo (03°59'S, 79°21'W), Dec 1955, R. Levi-Castillo (1♂; USNM).

HONDURAS. **Cortés:** San Pedro Sula (8 km S; 15°25.7'N, 88°01.4'W), 25–26 Sep 1995, D. and W. N. Mathis (1♂; USNM).

MEXICO. **Chiapas:** Cascadas de Agua Azul (17°15.3'N, 92°06.9'W), 7 May 1985, W. N. Mathis (1♂, 1♀; USNM). **Veracruz-Llave:** Fortin de las Flores (18°54'N, 97°W; 952 m), 2 May 1985, W. N. Mathis (4♂, 6♀; USNM).

TRINIDAD and TOBAGO. Tobago. **St. John:** Parlatuvier (creek; 11°17.9'N, 60°35'W), 14 Jun 1993, W. N. Mathis (1♂; USNM).

##### Distribution

**Figure 85.**
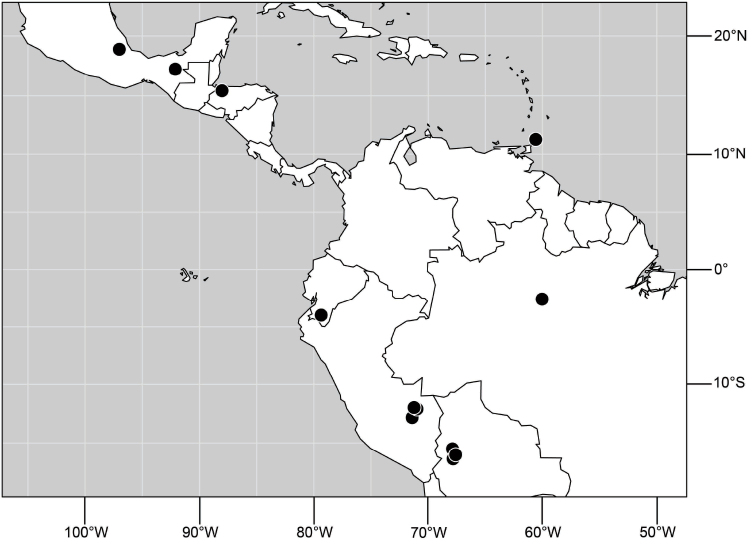
Distribution of *Hydrochasma falcatum* sp. n.

(Fig. 85). Neotropical: Bolivia (La Paz), Brazil (Amazonas), Ecuador (Loja), Honduras (Cortés), Mexico (Chiapas, Veracruz-Llave), Peru (Madre de Dios), Trinidad and Tobago.

##### Etymology.

The species epithet, *falcatum*, is of Latin derivation and means sickle shaped, referring to the sickle-shaped gonite in lateral view.

##### Remarks.

**Figures 86–89.**
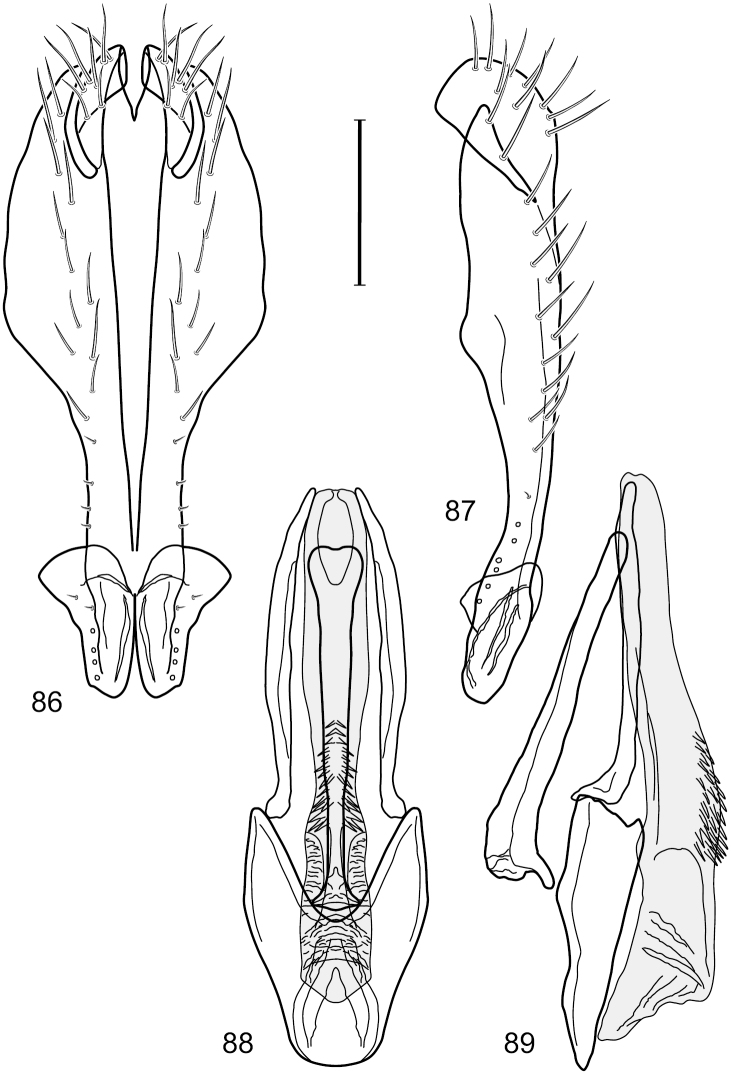
*Hydrochasma glochium* sp. n. (Dominican Republic. Puerto Plata (14 km W), Rio Camu) **86** epandrium and cerci, posterior view **87** same, lateral view **88** internal structures of male terminalia (aedeagus [shaded], phallapodeme, gonite, hypandrium), ventral view **89** same, lateral view. Scale bar = 0.1 mm.

Like *Hydrochasma glochium* and to a lesser degree like *Hydrochasma urnulum*, this species has an expanded apical portion of the extended epandrial process (best seen in posterior view). That process, however, has only moderate, lateral extensions (Fig. 81). The hypandrium of *Hydrochasma falcatum* is somewhat rectangular in ventral view with a distinct, V-shaped posteromedial emargination (Fig. 83). In males of *Hydrochasma glochium*, the expanded apical portion is more pronounced (Fig. 86), and the hypandrium in robustly V-shaped (Fig. 88). This species is also like those of the *faciale* group in having a spur-like seta ventroapically on the hindtibiae.

#### 15. Hydrochasma glochium sp. n.

http://zoobank.org/ED79E8CD-D5A4-4110-BDAD-AAF85CC24439

http://species-id.net/wiki/Hydrochasma_glochium

Figs 86 –90

##### Diagnosis.

This species is distinguished from congeners by the following combination of characters: Small shore flies, body length 1.65–1.95 mm. *Head*: Antenna mostly dark gray; parafacial silvery white, concolorous with facial coloration; gena-to-eye ratio 0.19–0.21. *Thorax*: Wing with costal vein ratio 0.72–0.74; M vein ratio 0.50–0.53. Forecoxa whitish gray basally, apical 2/3–3/4 yellow; forefemur lacking a distinctive, comb-like row of stout setulae along anteroventral surface; tibiae mostly yellow; hindtibia with silvery gray, broad band medially. *Abdomen*: Tergites 3–4 with deep, silvery gray wedges laterally; tergite 5 mostly silvery gray, only anterior brownish black, and with faint blackish coloration posteriorly. Male terminalia (Figs 86–89): Combined structures generally moderately elongate, in posterior view height slightly less than 3× width, generally sparsely setulose, more so dorsally, setulae sparse medially, setulae becoming smaller ventrally; epandrium lacking dorsal arch above cerci, in posterior view (Fig. 86) with apical 1/2 abruptly narrowed, mostly parallel sided, apical process conspicuously wider than apical 1/2, flared laterally subapically, apex with very narrow apicomedial V-shaped cleft, in lateral view (Fig. 87) elongate, narrow, generally shallowly curved, apical process with subapical expansion, apex bluntly rounded; aedeagus in lateral view (Fig. 89) very elongate and narrow, generally expanding toward apex, apical 1/2 with tiny, cuticular, narrow projections, apical 1/4 with complex folding, apex skewed anteriorly, narrowly pointed, in ventral view (Fig. 88) generally narrow and elongate, slightly narrowed at midlength, apical 1/3 slightly expanded, moderately bulbous, apex broad; phallapodeme in lateral view (Fig. 89) very narrow and elongate, rod-like to narrowly clavate, gradually expanded from aedeagal end to hypandrial end, latter with very narrowly pointed keel, aedeagal end straight, in ventral view (Fig. 88) elongate, narrow, truncate at both ends, slightly and gradually expanded at aedeagal end, hypandrial end with 2 narrow crossbars; gonite in lateral view (Fig. 89) as nearly straight, rod-like process, almost equal in length to phallapodeme, in ventral view (Fig. 88) nearly straight, very slightly wider medially, tapered at both apices; hypandrium in lateral view (Fig. 89) narrowly developed, posterior half narrowly angled, tapered toward anterior margin, narrowly angulate anteriorly, in ventral view (Fig. 88) moderately deeply and narrowly V-shaped, arms of V generally thick, pointed posteriorly, anteromedially margin bluntly rounded.

##### Type material.

The holotype male of *Hydrochasma glochium* is labeled “**DOMIN[ICAN]. REP[UBLIC].** Peravia: San J[osé] Ocoa,RioOcoa[,] 18°31.7'N, 70°30.4'W[,] 21May1998, WNMathis/USNM ENT 00087391 [plastic bar code label]/HOLOTYPE ♂ *Hydrochasma glochium* Mathis & Zatwarnicki, USNM [red].” The holotype is double mounted (minuten in a block of plastic), is in excellent condition (abdomen removed and dissected, parts in an attached microvial), and is deposited in the USNM. Paratypes are as follows: DOMINICAN REPUBLIC. **La Vega:** Salto Baiguate (near Jarabacoa; 19°05.5'N, 70°36.9'W; 570 m), 9 May 1995, W. N. Mathis (1♀; USNM). **Pedernales:** Cabo Rojo (26 km N; 18°06'N, 71°38'W; 730 m), 31 Jul 1990, J. E. Rawlins, S. Thompson, C. Young (1♂, 1♀; CMP).

##### Type locality.

Dominican Republic. Peravia: San José Ocoa (10 km NE; 18°35'N, 70°25.6'W).

##### Other specimens examined.

Neotropical. BOLIVIA. **La Paz:** Guanay (15°29.8'S, 67°52.7'W), 460 m), 13 Mar 2001, W. N. Mathis (2♂; USNM).

BRAZIL. **São Paulo:** Ubatuba, Praia Puruba (23°21'S, 44°55.6'W; beach), 29 Mar 2010, D. and W. N. Mathis (5♂; USNM).

COSTA RICA. **Limón:** Parque Nacional Cahuita, Sector Puerto Vargas (09°44'N, 82°49'W; 1 m), 27-28 Jun 2001, J. D. Gutierrez (1♂, 1♀; INBio).

ECUADOR. **Orellana:** RíoTiputini (0°38.2'S, 76°8.9'W), 12-26 Aug 1999, W. N. Mathis, A. Baptista, M. Kotrba (1♂; USNM).

VENEZUELA. **Zulia:** Carrasquero (11°02.1'N, 72°0.3'W), 29-30 May 1976, A. S. Menke, D. Vincent (1♂, 2♀; USNM).

West Indies. BARBADOS. **Christ Church:** Graeme Hall Swamp (13°04.2'N, 59°34.7'W), 2 Sep 1997, D. and W. N. Mathis (1♂; USNM).

##### Distribution

**Figure 90.**
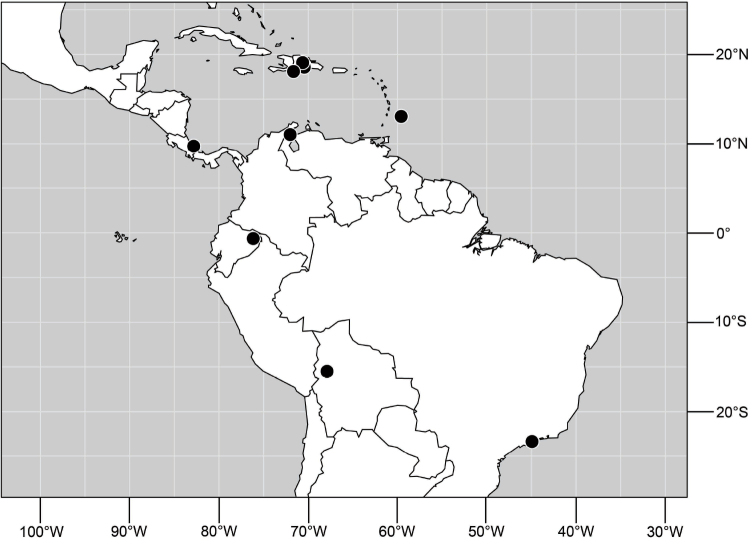
Distribution of *Hydrochasma glochium* sp. n.

(Fig. 90). Neotropical: Bolivia (La Paz), Brazil (São Paulo), Costa Rica (Limón), Ecuador (Orellana), Venezuela (Zulia), West Indies (Barbados, Dominican Republic).

##### Etymology.

The species epithet, *glochium*, is of Greek derivation and means arrow head, referring to the arrow-like apex of the ventral extension of the epandrium.

##### Remarks.

In the remarks section of the previous species (p. 61), we noted characters to distinguish this species from *Hydrochasma falcatum*, particularly the more pronounced, lateral extensions of the apical portion of the ventral, the epandrial process, and the robust, V-shaped, hypandrium. (Figs 86-88).

#### 16. Hydrochasma incisum

(Coquillett)

http://species-id.net/wiki/Hydrochasma_incisum

Figs 91 –95

Discocerina incisa Coquillett, 1902: 182.
Discocerina leucoprocta incisa Cresson, 1918: 58 [subspecies status].
Hydrochasma incisum . Cresson 1946: 142 [review]. Wirth 1968: 8 [Neotropical catalog]. Mathis and Zatwarnicki 1995: 182-183 [world catalog]. Mathis 1997: 36 [review, Belize].
Discocerina (Gymnoclasiopa) poecilogastra Hendel, 1930: 138. Cresson 1938: 29 [synonymy].

##### Diagnosis.

**Figures 91–94.**
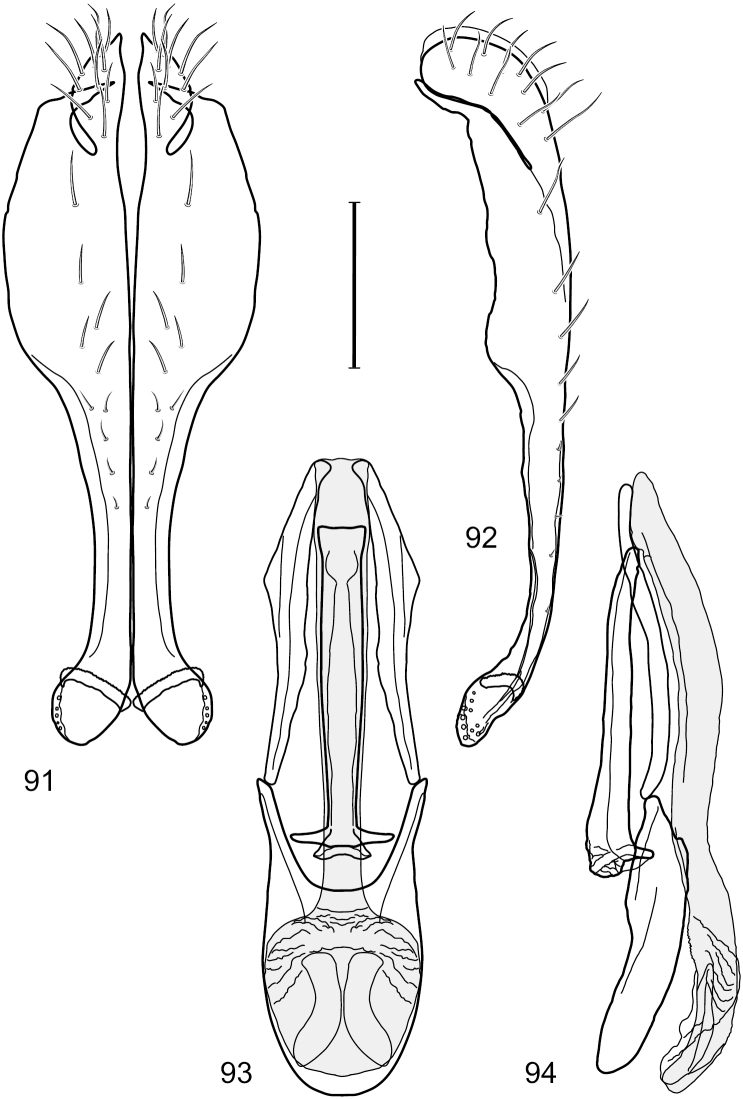
*Hydrochasma incisum* (Coquillett) (Peru. Cuzco: Paucartambo, Atalaya) **91** epandrium and cerci, posterior view **92** same, lateral view **93** internal structures of male terminalia (aedeagus [shaded], phallapodeme, gonite, hypandrium), ventral view **94** same, lateral view. Scale bar = 0.1 mm.

This species is distinguished from congeners by the following combination of characters: Small to moderately small shore flies, body length 1.35–2.05 mm. *Head*: Antenna mostly dark gray; parafacial silvery white, concolorous with facial coloration; gena-to-eye ratio 0.18–0.20. *Thorax*: Wing with costal vein ratio 0.56–0.58; M vein ratio 0.50–0.51. Forecoxa with base silvery white to gray, apical 2/3 yellowish; forefemur lacking a distinctive, comb-like row of stout setulae along anteroventral surface; tibiae mostly gray. *Abdomen*: Tergites 3–4 with distinctive, deep, wedge-like lateral margin of darkened dorsum; tergite 5 of male mostly gray with posterior margin blackish. Male terminalia (Figs 91–94): Combined structures generally elongate, in posterior view height less than 3× width (2.8×), generally sparsely setulose, especially dorsally, setulae sparse medially along dorsal 2/3; epandrium lacking dorsal arch above cerci, in posterior view (Fig. 91) with apical 1/2 abruptly narrowed, mostly parallel sided, apical process wider than apical 1/2 and angled ventrolaterally, apex with very narrow apicomedial cleft, in lateral view (Fig. 92) very elongate, narrow with basal 3/4 straight, apical 1/2 narrow to just before slightly broadened and curved apex, apex narrowly rounded; aedeagus in lateral view (Fig. 94) very elongate and narrow, shallowly sinuous, mostly parallel sided, apical 1/4 [or 1/3] with complex folding and secondary structures, apex rounded, in ventral view (Fig. 93) very narrow and elongate, apical 1/4 [or 1/3] very bulbously expanded, apex broad; phallapodeme in lateral view (Fig. 94) very narrow and elongate, rod-like, hypandrial end with very narrowly pointed keel, aedeagal end straight, in ventral view (Fig. 93) elongate, narrow, truncate at both ends, slightly and gradually expanded at aedeagal end, hypandrial end with 2 narrow crossbars; gonite in lateral view (Fig. 94) as a very shallowly sinuous, rod-like process, about equal in length to phallapodeme, in ventral view (Fig. 93) nearly straight but expanded at basal 1/3 to an obtuse point, tapered at both apices; hypandrium in lateral view (Fig. 94) narrowly developed, posterior half narrowly angled, gradually expanded toward midlength, thereafter cigar-like, in ventral view (Fig. 93) moderately deeply and thickly U-shaped, with base of U rounded rectangular, robustly developed, extended lateral arms tapered, thin apically, moderately elongate.

##### Type material.

The lectotype male of *Discocerina incisa* Coquillett, here designated to preserve stability and make more universal the use of this name, is labeled “Mayaguez Jan 1899./Porto Rico Aug[ust] Busck/Type No 6642 U.S.N.M. [red; “6642” handwritten]/Discocerina incisa Coq. [name and author handwritten, apparently by Coquillett; black submargin]/LECTOTYPE ♂ *Discocerina incisa* by Mathis & Zatwarnicki, USNM (6642) [red]. The lectotype is double mounted (glued to a paper triangle), is in good condition (structures of male terminalia exposed), and is deposited in the USNM (6642).” Coquillett (1902: 183) noted that the type series comprised eight specimens with no single specimen designated as the type or holotype. Two of the eight specimens are missing (pins with labels still extant, specimens missing). From the other six specimens (Vieques Island, Fajaro, Mayaquez, Aguadillo, Utuado and Arroyo), we selected the male from Mayaquez list above as the lectotype because it is in good condition and is easily identified to species. The other specimens, here designated as paralectotypes, appear to be conspecific (we did not make dissections of the other males), but one paralectotype from Vieques Island appears to be a male of *Hydrochasma leucoproctum*.

The holotype male of *Discocerina (Gymnoclasiopa) poecilogastra* Hendel is labeled “Fortin Esteros Bolivia. III.26. D.Chaco-Exped [black border]/Discocerina poecilogastra ♀ Hend. [handwritten]/Type Hendel 1930 [red lettering; black border].” The holotype is double mounted (minuten in a paper card), is in good condition, and is deposited in the SMN.

##### Type locality.

Puerto Rico. Mayaguez (18°12.1'N, 67°08.7'W).

##### Other specimens examined.

Nearctic: UNITED STATES. FLORIDA. **Highlands:** Archbold Biological Station (27°12.6'N, 81°20.9'W), 7 Feb 2000, D. and W. N. Mathis (2♂; USNM); Archbold Biological Station (27°11.3'N, 81°20.3'W), 7 Mar-12 Nov 1999, 2000, M. A. Deyrup (3♂, 10♀; ABSF).

Neotropical. ARGENTINA. **Salta:** Embarcación (23°12.6'S, 64°05.8'W), 2–6 Feb 1930, R. Golbach (1♂; USNM).

BELIZE. **Belize:** Blackbird Caye (17°19'N, 87°48'W), 27–30 Mar 1993, W. N. Mathis (1♂, 1♀; USNM); Turneffe Islands: Calabash Cays (17°17'N, 87°48'W), 27–30 Mar 1993, W. N. Mathis (41♂, 17♀; USNM). **Stann Creek:** Dangriga (16°58'N, 88°13'W), 3–4 Apr 1993, W. N. Mathis (14♂, 6♀; USNM); Kendal (16°47.4'N, 88°22.9'W), 1 May 1997, R. Faitoute, P. J. Spangler (1♂, 1♀; USNM); Man of War Cay (17°13'N, 87°54'W), 8–15 Nov 1987, D. and W. N. Mathis (2♂; USNM); Placencia Lagoon, Rum Point (16°32.8'N, 88°22.1'W), 4–5 Nov 1987, D. and W. N. Mathis (22♂, 30♀; USNM); Salt Creek (12 km N Dangriga; 17°13.4'N, 88°18.6'W), 28 Mar 1988, W. N. Mathis (2♀; USNM); Sittee River, Possum Point Biological Station (16°52.1'N, 88°22.5'W), 6 Nov 1987, D. and W. N. Mathis (7♂, 1♀; USNM); Twin Cays (south end of East Island, West Bay; 19°49.5'N, 88°06.2'W), Jan-19 Mar 1987, 1988, W. N. Mathis, C. Feller (2♂, 2♀; USNM); Wee Wee Cay (16°45.9'N, 88°08.6'W), Jan-9 Nov 1987, 1988, D. and W. N. Mathis, C. Feller (36♂, 26♀; USNM).

BOLIVIA. **Beni:** Huachi, Mulford Biological Expedition (14°14'S, 63°31'W), Sep 1921, W. M. Mann (3♂; ANSP). **La Paz:** Chulumani (2 km S; 16°23.5'S, 67°31.8'W; 1750 m), 9–10 Mar 2001, W. N. Mathis (12♂, 13♀; USNM); Guanay (15°29.8'S, 67°52.7'W), 460 m), 13 Mar 2001, W. N. Mathis (10♂, 2♀; USNM); Guanay (8 km E; 15°30.3'S, 67°50.8'W; 510 m), 13 Mar 2001, W. N. Mathis (4♂, 2♀; USNM); Guanay (9 km NW; 15°29.5'S, 67°53.8'W; 850 m), 13 Mar 2001, W. N. Mathis (7♂, 7♀; USNM); Guanay (3 km E; 15°30.2'S, 67°52.3'W; 500 m), 14 Mar 2001, W. N. Mathis (15♂, 1♀; USNM); Guanay (22 km SE; 15°17.8'S, 68°15.6'W; 540 m), 17 Mar 2001, W. N. Mathis (1♂; USNM); Mapiri (15°18.6'S, 68°13'W; 720 m), 15–17 Mar 2001; S. D. Gaimari, W. N. Mathis (18♂, 4♀; USNM); San Pedro (3 km NE; 16°S, 67°35.3'W; 780 m), 12 Mar 2001, W. N. Mathis (1♂; USNM); Tajlihui (15°40.8'S, 67°41.7'W; 590 m), 12 Mar 2001, W. N. Mathis (12♂, 3♀; USNM).

BRAZIL. **Amazonas:** Igarapé Cabeça Branca (ca. 40 km N Manaus; 02°35.1'S, 60°01.9'W; 65 m), 8 May 2010, D. and W. N. Mathis (5♂, 2♀; INPA, USNM); Reserva Ducke (02°55.8'S, 59°58.5'W; 40 m), 5 May 2010, D. and W. N. Mathis (1♂; INPA, USNM). **Paraná:** Antonina (25°28'S, 48°41.3'W; 13 m), 3 Feb 2010, D. and W. N. Mathis (7♂; DZUP, USNM); Antonina (25°27.1'S, 48°41.1'W; beach; Ponta da Pita), 15 Feb 2010, D. and W. N. Mathis (4♂; DZUP, USNM); Bocaiúva do Sul (25°16.6'S, 48°58.5'W; 770 m), 16 Feb Jan 2010, D. and W. N. Mathis (2♂; DZUP, USNM); Castro (Parque Lacustre; 24°47.4'S, 50°0.3'W; 990 m), 25 Dec 2009, D. and W. N. Mathis (17♂; DZUP, USNM); Castro (8 km N; 24°45.3'S, 49°58.9'W; 1010 m), 25–26 Dec 2009, D. and W. N. Mathis (1♂, 1♀; USNM); Curitiba, Universidade Federal do Paraná, Reserva Biológica (25°26.9'S, 49°14'W; 915 m), 22 Dec-12 Apr 2009, 2010, D. and W. N. Mathis (43♂, 9♀; DZUP, USNM); Matinhos (Rio da Onça; 25°47.1'S, 48°31.6'W; 3 m), 27 Jan-25 Mar 2010, D. and W. N. Mathis (23♂, 1♀; DZUP, USNM); Matinhos (Rio da Onça; 25°47.4'S, 48°31.6'W; 3 m), 12 Nov 2010, D. and W. N. Mathis (6♂; DZUP, USNM); Matinhos (N.; 25°46.4'S, 48°30.8'W; 1 m; beach/estuary), 30 Jan-25 Mar 2010, D. and W. N. Mathis (13♂; DZUP, USNM); Paranaguá (25°30.8'S, 48°29.9'W; 3 m), 23 Jan 2010, D. and W. N. Mathis (2♂; DZUP, USNM); Prainha (5 km S Matinhos; 25°51.2'S, 48°33.6'W; beach), 15 Nov 2010, D. and W. N. Mathis (3♂; DZUP, USNM); Parque Igauçu (25°33.4'S, 49°13.6'W; 880 m), 16 Jan-11 Feb 2010, D. and W. N. Mathis (22♂, 1♀; DZUP, USNM). **Rio de Janeiro:** Ilha da Marambaia (23°03.6'S, 43°59.1'W), 4 Sep 2000, W. N. Mathis (7♂, 3♀; USNM). **Santa Catarina:** Barra Velha (26°38'S, 48°40.9'W; beach), 29 Apr 2010, D. and W. N. Mathis (5♂; DZUP, USNM). **São Paulo:** Ubatuba, Praia do Estaleiro (23°20.5'S, 44°53'W; beach), 30 Mar 2010, D. and W. N. Mathis (2♂; USNM).

CHILE. **Tarapacá:** Pica (20°29.3'S, 69°19.7'W), Sep/Oct 1966, M. E. Irwin, E. Medina (1♂; USNM).

COSTA RICA. **Alajuela:** Bijagua, Volcán Tenorio (10°43.8'N, 85°05.9'W; 700 m), 19–27 Aug 2002, J. D. Gutierrez (14♂, 5♀; INBio); Caño Negro (10°30.2'N, 84°24'W; 20 m), 2–23 Apr 1995, R. Villalobos (3♂, 2♀; INBio); Rincón de la Vieja, San Gerrardo (10°47.2'N, 85°17.6'W; 600 m), 16–18 May 2002, D. Briceño (1♀; INBio); Río Surubres (09°56.1'N, 84°35'W; Bonnefil farm; 245 m; sweeping), 20 Oct 1909, P. P. Calvert (2♂, 1♀; ANSP); Turrúcares (09°57.6'N, 84°19.2'W), 22 Dec 1909, P. P. Calvert (5♂, 5♀; ANSP). **Cartago:** Cartago (09°51.4'N, 83°55.2'W), 4 Jul-27 Oct 1909, P. P. Calvert (9♂, 3♀; ANSP); Juan Viñas (09°53.6'N, 83°15.3'W), 28 Apr 1910, P. P. Calvert (2♂; ANSP); La Suiza (09°51.5'N, 83°37.5'W), 28 Jun 2001, W. N. Mathis (7♂, 3♀; ANSP, USNM); Pejibaye (09°48.1'N, 83°42.7'W; La Reserva Biológica del Copal; 1090 m), 4 Apr 2005, J. Azofeifa, D. Briceño (3♂, 2♀; INBio); Peralta (09°58.1'N, 83°36.9'W), 24 Mar 1910, P. P. Calvert (2♂; ANSP). **Guanacaste:** Bagaces Fortuna Z. P. Miravalles (10°43.1'N, 84°51.3'W; Sendero Cabro Muco; 980 m), 8–31 Jul 2002, J. D. Gutierrez (17♂, 8♀; INBio); Cerro Vista al Mar (10°10'N, 85°35'W; 900 m), 23 Jun 2001, A. Freidberg (1♂, 2♀; USNM); Filadelfia (Río Tempisque; 10°26.7'N, 85°32.9'W), 18 Jan 1910, P. P. Calvert (4♂, 4♀; ANSP); Volcán Miravalles, Quebrada Santa Fé (10°44.9'N, 85°09.2'W; 980 m), 17–19 Jul 2002, J. D. Gutierrez (4♂; INBio); Playa Puerto Soley (11°02.5'N, 85°40.1'W; beach), 16 Jun 2003, D. and W. N. Mathis (1♂; USNM); Liberia, Sendero Lupo (10°38.1'N, 85°26.4'W; 300 m), 10–11 Jul 2002, D. Briceño (9♂, 13♀; INBio); Liberia, Sendero Los Borrachos (10°38.1'N, 85°26.4'W; 300 m), 3–5 Oct 2002, D. Briceño (2♀; INBio); Nandayure, Carmona, Finca Agua Fria, Rio Nandayure (09°59.6'N, 85°15.1'W; 15–50 m), 14 Feb 2006, W. Porras (3♂, 4♀; INBio); Nandayure, Costa de Oro (09°59'N, 85°19.1'W; 0–50 m), 6 Apr 2004, W. Porras (1♂; INBio); Nandayure, Estero Casa Caletas (09°46.6'N, 85°15.5'W; 0–50 m), 21 Mar 2006, W. Porras (1♀; INBio); Nandayure, Finca Pochote (10°03.2'N, 85°28'W; 100–200 m), 5 May 2004, B. Gamboa (1♀; INBio); Nosara, Río Nosara (09°59'N, 85°39'W; 0–5 m), 15 Jun 2004, M. Moraga (1♀; INBio); Palo Verde (10°23'N, 85°20'W; 0–30 m), 16 Nov 2004, W. Porras (1♀; INBio); Parque Nacional Santa Rosa (Estación; Camino Cafetal; 10°51.5'N, 85°36.7'W; 300 m), 3–5 Aug 2002, D. Briceño (5♂; INBio); Puerto Cortés Balsar, La Tigra (10°10'N, 84°30'W), 5 Jun 2005, J. D. Gutierrez (1♂, 2♀; INBio); San Antonio (09°59.5'N, 85°18.4'W; 8 m), 4 Jul 2005, J. D. Gutierrez (1♂, 4♀; INBio); Santa Cruz (14 km S; 10°10.4'N, 85°35.7'W; 180 m), 23 Jun 2001, D. and W. N. Mathis (24♂, 2♀; USNM); Santa Rosa (10°49.7'N, 85°42.3'W; 300 m), 10 Aug 2002, 16–18 May 2002, D. Briceño (1♀; INBio); Santa Rosa, Bosque San Emílio (10°48.9'N, 85°36.9'W; 300 m), 10 Aug 2002, D. Briceño (3♂, 7♀; INBio); Sendero Rancho Capú, Río Piedras, Volcán Tenorio (10°23'N, 85°13'W; 740 m), 13–28 Aug 2002, J. D. Gutierrez (5♂; INBio); Sector el Hacha, Finca el Oro, 5 km SW Hacienda Alemania (11°00'N, 85°33'W; 400 m), 14–19 Apr 2002, D. Briceño (1♂; INBio); Volcán Tenorio, Montezuma (10°40.4'N, 85°0.9'W; 1000 m), 6 May 2002, J. D. Gutierrez (1♂; INBio). **Heredia:** Santo Domingo (Parque INBio; 09°59'N, 85°06'W), 23 Jun 2001, D. and W. N. Mathis (9♂, 1♀; USNM); Santo Domingo (Parque INBio; 09°59'N, 85°06'W; laguna), 24 Feb 2003, J. Azofeifa (1♂, 1♀; INBio; INBio). **Limón:** Cahuita (09°44.3'N, 82°50.4'W; beach), 28 Jun 2001, A. Freidberg (1♂; USNM); Puerto Vargas (09°43.9'N, 82°48.9'W; beach), 28 Jun 2001, W. N. Mathis (2♂; USNM); Puerto Vargas, Talamanca (09°43.9'N, 82°48.9'W; beach), 9–10 Dec 2001, E. Rojas (2♂, 4♂; INBio; INBio); Parque Nacional Cahuita, Sector Puerto Vargas (09°44'N, 82°49'W; 1 m), 27–28 Jun 2001, J. D. Gutierrez (4♂, 2♀; INBio); (4♂, 3♀; INBio); Río Banano (09°50.5'N, 82°56'W), 9 Nov 1909, P. P. Calvert (1♀; ANSP); Tortugero, Río Agua Fria, Sendero Real (10°32.3'N, 83°30.1'W; 100 m), 17 Aug 2004, Y. Cardenas (1♂; INBio); Westfalia (4 km S; 9°54.5'N, 82°59'W; beach), 27 Jun 2001, W. N. Mathis (1♀; USNM). **Puntarenas:** Bahia Gigante (Río Lajas; 09°53.8'N, 84°56'W; beach), 22 Jun 2001, D. and W. N. Mathis (3♂; USNM); Bosque Esquinas (08°44'N, 83°17'W; 200 m), May 1994, M. Segura (1♀; INBio); Cabuye (Río Lajas; 9°37'N, 85°04.8'W), 20 Jun 2001, D. and W. N. Mathis (9♂, 2♀; USNM); Chomes (10°2.7'N, 84°54.7'W), 19 Jun 2001, W. N. Mathis (3♂, 5♀; USNM); Cóbano (3 km W; 09°40.2'N, 85°06.8'W), 21 Jun 2001, D. and W. N. Mathis (14♂, 1♀; USNM); Corcovado, La Leona (08°27.3'N, 82°30.3'W; 100–300 m), 26 Jul 2003, M. Moraga (1♂; INBio); Dominical (6 km S; 09°13.1'N, 83°49.8'W; waterfall), 12 Jun 2003, D. and W. N. Mathis (6♂; USNM); Drake (08°41.4'N, 83°40.1'W; beach), 12 Aug 2001, D. and W.N. Mathis (2♂; USNM); Estación Sirena (08°28.8'N, 83°35.4'W; 0–100 m), 20–27 Mar 1995, A. Azofeifa (1♂; INBio); Isla de San Lucas (09°56.8'N, 84°54.2'W), 10 Mar 2005, M. Moraga (2♂; INBio); Isla de San Lucas, Playa El Coco (09°56.7'N, 84°53.8'W), 9 Mar 2005, M. Moraga (1♂; INBio); Jacó (5 km E; 09°34.7'N, 84°06.4'W), 10 Jun 2003, D. and W. N. Mathis (8♂, 2♀; USNM); Llano Bonito (09°39.9'N, 84°06.4'W; 50–100 m), 28 Apr 2001, W. Porras (1♂; INBio); Malpais (09°37.6'N, 85°09.1'W; beach), 21 Jun 2001, D. and W. N. Mathis (1♂, 2♀; USNM); Montezuma (1 km S; 09°39'N, 85°04.3'W), 20 Jun 2001, D. and W. N. Mathis (15♂, 3♀; USNM); Palustrino Corral de Piedra (10°14.1'N, 85°19.9'W; 0–100 m), 10 Jul 2003, Y. Cardenas (1♂; INBio); Parrita (09°31.2'N, 84°19.6'W; 8–9 m), 10 Jun 2003, D. and W. N. Mathis (1♂, 3♀; USNM); Playa Jacó (09°36.5'N, 84°37.4'W; beach), 13 Jun 2003, D. and W. N. Mathis (10♂, 1♀; USNM); Rancho Quemado (08°41.5'N, 83°34.3'W; 200 m), Aug 1991, F. Quesada (1♂; INBio); Rincón (5 km S; 08°42.1'N, 83°30.8'W; 95 m), 10–11 Aug 2001, D. and W.N. Mathis (28♂, 9♀; USNM); Río Corcovado (08°34.2'N, 83°36.5'W; 0–10 m), 22 Mar 1955, M. A. Zumbado (1♂; INBio); Río Tigre (08°32.1'N, 83°22.1'W; 100 m), 23 Nov 1996, A. Azofeifa (1♀; INBio); San Pedrillo (08°37.2'N, 83°44.1'W), 12–14 Aug 2001, D. and W.N. Mathis (29♂, 1♀; USNM). **San José:** El Rodeo (09°54.6'N, 84°16.2'W; 1860 m), 26 Jun 2001, D. and W. N. Mathis (27♂, 4♀; USNM); Río Paraíso (09°33.8'N, 84°07.4'W; 350–400 m), 15–17 Feb 2003, D. and W. N. Mathis (1♀; USNM); Río Virilla (near Colon; 9°55.3'N, 84°16'W), 26 Jun 2001, D. and W. N. Mathis (2♂; USNM); Zurquí (10°02.8'N, 84°04'W), 19 Feb 2003, D. and W. N. Mathis (1♂; USNM).

ECUADOR. **Azuay:** Río Rircay (03°20'S, 79°19'W), 31 Oct 1954, R. Levi-Castillo (4♂; USNM). **Guayas:** Río Bobo (01°53.8'S, 79°42'W), Aug 1955, R. Levi-Castillo (1♂; USNM). **Loja:** Catamayo (03°59'S, 79°21'W), Dec 1955, R. Levi-Castillo (1♂; USNM).

GUYANA. Conservation of Ecological Interactions and Biotic Associations (CEIBA; ca. 40 km S Georgetown; 06°29.9'N, 58°13.1'W), 13–21 Apr 1994, 1995, 1997, W. N. Mathis (8♂, 13♀; USNM); Dubulay Ranch (small creek; 05°40.9'N, 57°51.5'W), 10 Apr 1994, W. N. Mathis (2♂, 1♀; USNM); Dubulay Ranch, Berbice River (05°40.9'N, 57°51.5'W), 9–11 Apr 1994, W. N. Mathis (1♂, 1♀; USNM); Hope Beach (06°44.7'N, 57°57.3'W), 20 Aug 1997, W. N. Mathis (2♂; USNM); Georgetown (06°48.6'N, 58°8.6'W; 340 m), 20–29 Aug 1997, W.N. Mathis (12♂, 1♀; USNM); Karanambo (03°45.1'N, 59°18.6'W), 31 Mar-1 Dec 1994, 2001, W. N. Mathis (52♂, 12♀; USNM); Karanambo, Rupununi River (ox bow; 03°45.1'N, 59°18.6'W), 2 Apr-2 Dec 1994, 2001, W. N. Mathis (8♂, 9♀; USNM); Karanambo (forest swamp; 03°44'N, 59°20.4'W; 95 m), 5 Dec 2010, W. N. Mathis (2♂, 1♀; USNM); Kumu River and Falls (25 km SE Lethem in Kanuku Mountains; 03°15.9'N, 59°43.6'W), 28–30 Apr 1995, W. N. Mathis (2♂; USNM); Lethem, Takatu River (03°22.6'N, 59°48.2'W), 4 Apr 1994, W. N. Mathis (14♂, 8♀; USNM); Mahaica (3 km E; 06°43.5'N, 57°56.6'W), 14 Apr 1994, W. N. Mathis (2♂; USNM); Moco-Moco (30 km E Lethem in Kanuku Mountains; 03°18.2'N, 59°39.0'W), 3–29 Apr 1994, 1995, W. N. Mathis (18♂, 8♀; USNM); Paramakatoi (04°42'N, 59°42.8'W), 24–25 Aug 1997, W. N. Mathis (14♂, 13♀; USNM); Pirara Ranch, Cashew Lake (03°36.7'N, 59°40.5'W), 23–27 Apr 1995, W. N. Mathis (2♂; USNM); Pirara Ranch and River (03°32.1'N, 59°40.5'W), 24–25 Apr 1995, W. N. Mathis (11♂, 14♀; USNM); Wiruni River (05°46.6'N, 58°01'W), 11 Apr 1994, W. N. Mathis (11♂, 9♀; USNM).

HONDURAS. **Cortés:** San Pedro Sula (8 km S; 15°25.7'N, 88°01.4'W), 25–26 Sep 1995, D. and W. N. Mathis (7♂; USNM).

MEXICO. **Tamaulipas:** Tampico (22°17.8'N, 97°50.8'W), Jul 1906, F. C. Bishop (1♂; ANSP). **Veracruz-Llave:** Ciudad Alemán (18°11.8'N, 96°05.1'W; 100 m), 3 May 1985, W. N. Mathis (1♂, 3♀; USNM); Ocotal Chico (18°15.6'N, 94°51.5'W; 600 m), 4–5 May 1985, W. N. Mathis (2♂, 3♀; USNM).

NICARAGUA. **León:** La Cruz de la India (5 km SW; 12°43.2'N, 86°19.8'W; 215 m), 22 Jun 2007, N. E. Woodley (2♂, 2♀; USNM).

PANAMA. **Panama:** Fort Clayton (09°01'N, 79°34.2'W), 23 Apr 1923, R. C. Shannon (1♂; ANSP).

PARAGUAY. **Cordillera:** San Bernardino (25°16'S, 57°19.4'W), Feb-Mar 1906, Babarczy (1♀; ANSP).

PERU. **Cuzco:** Paucartambo, Atalaya (Río Alto Madre de Dios; 12°53.3'S, 71°21.6'W; 600 m), 4 Sep 1988, W. N. Mathis (12♂, 15♀; USNM); Paucartambo, Atalaya (2 km W; 12°54.3'S, 71°22.4'W), 4 Sep 1988, W. N. Mathis (6♂, 1♀; USNM). **Madre de Dios:** Diamante (Río Alto Madre de Dios; 12°19.9'S, 70°57.5'W; 400 m), 7 Sep 1988, W. N. Mathis (20♂, 8♀; USNM); Río Manu, Cocha Salvador (11°59.9'S, 71°13.9'W 300m), 14 Sep 1988, W. N. Mathis (1♂; USNM); Río Manu, Erika (near Salvación; 12°50.7'S, 71°23.3'W; 550 m), 5–6 Sep 1988, W. N. Mathis (1♂, 1♀; USNM); Río Manu, Pakitza (11°56.6'S, 71°16.9'W; 250 m), 9–23 Sep 1988, W. N. Mathis (1♂; USNM).

TRINIDAD and TOBAGO. Tobago. **St. George:** Blanchisseuse (beach; 10°48'N, 61°19'W), 25 Jun 1993, W. N. Mathis (1♂; USNM). **St. John:** Bloody Bay River (11°18'N, 60°38'W), 14 Jun 1993, W. N. Mathis (1♂; USNM); Charlotteville (beach; 11°19.5'N, 60°32.9'W), 16–18 Apr-10–16 Jun 1993, 1994, D. and W. N. Mathis (38♂, 27♀; USNM); Charlotteville (2 km S; 11°19'N, 60°33'W), 10 Jun 1993, 1994, W. N. Mathis (2♂, 2♀; USNM); Charlotteville (5 km S; Hermitage River and beach; 11°18.9'N, 60°34.2'W), 22 Apr-10–11 Jun 1993, 1994, W. N. Mathis (11♂, 2♀; USNM); Parlatuvier (creek; 11°17.9'N, 60°35'W), 14 Jun 1993, W. N. Mathis (7♂, 1♀; USNM); Speyside (11°18'N, 60°32'W), 13–15 Jun 1993, W. N. Mathis (2♂; USNM); Speyside (Doctor River; 1 km NW; 11°18'N, 60°32'W), 12–13 Jun 1993, W. N. Mathis (2♂, 2♀; USNM); Speyside (Doctor River; 11°18.2'N, 60°31'W), 19 Apr 1994, D. and W. N. Mathis (2♂, 1♀; USNM). **St. Paul:** Argyle Falls (11°15'N, 60°35'W), 21 Apr 1994, W. N. Mathis (1♀; USNM); Kendall (11°14.3'N, 60°35.7'W), 21 Apr 1994, W. N. Mathis (3♂, 1♀; USNM); Roxborough (6 km NNW; 11°16'N, 60°35.4'W), 20 Apr 1994, W. N. Mathis (9♂, 6♀; USNM).

Trinidad. **Caroni:** San Rafael (2 km N; 10°34'N, 61°15'W; forest reserve), 22 Jun 1993, W. N. Mathis (1♀; USNM); Talparo (2 km N, 10°31'N, 61°17'W), 22 Jun 1993, W. N. Mathis (2♂; USNM). **Mayaro:** Plaisance (5 km N; 10°20.3'N, 60°59.5'W), 23 Mar 1985, G. F. and J. F. Hevel (3♀; USNM). **St. Andrew:** Lower Manzanilla (5 km S; 10°28'N, 61°03'W), 20 Jun 1993, W. N. Mathis (3♂, 12♀; USNM); Valencia (1 km W; 10°39'N, 61°13'W), Aripo River, 20 Jun 1993, W. N. Mathis (1♂, 2♀; USNM). **St. George:** Arima (8 km N; 10°41'N, 61°18'W), Verdant Vale, 19 Jun 1993, W. N. Mathis (1♀; USNM); Mount St. Benedict (10°39'N, 61°24'W; creek near base), 19 Jun 1993, W. N. Mathis (5♂; USNM). **St. Patrick:** Chatham (beach; 10°05'N, 61°44'W), 25 Jun 1993, W. N. Mathis (2♂, 2♀; USNM). **Victoria:** Basse Terre (7 km E; 10°07'N, 61°14'W), 27 Jun 1993, W. N. Mathis (3♂; USNM).

West Indies. BARBADOS. **Christ Church:** Graeme Hall Swamp (13°04.2'N, 59°34.7'W), 31 Aug-12 Sep 1996, 1997, D. and W. N. Mathis (7♂, 7♀; USNM); Oistins (13°03.9'N, 59°32.7'W), 22 May 1996, D. and W. N. Mathis, H. B. Williams (1♂, 1♀; USNM); Rockley Beach (13°04.3'N, 59°35.2'W), 21 May-11 Sep 1996, 1997, D. and W. N. Mathis, H. B. Williams (43♂, 17♀; USNM). **St. Andrew:** Bawdens Ponds (13°14.8'N, 59°34.9'W), 2 Sep 1997, W. N. Mathis (21♂, 8♀; USNM); Bawdens River-Swans (13°14.2'N, 59°35.3'W), 11 Sep 1996, W. N. Mathis (8♂, 4♀; USNM); Baxters (13°13.2'N, 59°34.1'W), 21 May-11 Sep 1996, D. and W. N. Mathis, H. B. Williams (18♂, 7♀; USNM); Belleplaine (13°14.8'N, 59°33.6'W), 21 May-1 Sep 1996, 1997, D. and W. N. Mathis, H. B. Williams (15♂, 10♀; USNM); Long Pond (13°15.1'N, 59°33.3'W), 21 May-11 Sep 1996, 1997, D. and W. N. Mathis, H. B. Williams (8♂, 4♀; USNM); Turner’s Hall Woods (13°13.8'N, 59°34.7'W), 21 May 1996, D. and W. N. Mathis, H. B. Williams (10♂, 3♀; USNM); Walkers Bridge (13°15'N, 59°34.5'W), 11 Sep 1996, W. N. Mathis (8♂, 2♀; USNM). **St. Joseph:** Joes River (13°12.8'N, 59°32.3'W), 10 Sep 1996, W. N. Mathis (19♂, 7♀; USNM). **St. Peter:** Six Mens Bay (13°16.5'N, 59°38.8'W), 22 May-12 Sep 1996, D. and W. N. Mathis, H. B. Williams (3♂; USNM). **St. Philip:** Gemswick (13°05'N, 59°28.5'W), 31 Aug 1997, W. N. Mathis (18♂, 9♀; USNM); **St. Thomas:** Farmers (13°12.8'N, 59°35.5'W), 2 Sep 1997, W. N. Mathis (3♂, 2♀; USNM).

CUBA. **Cienfuegos:** Jardin Botánico (22°7.5'N, 80°19.2'W), 13 Dec 1994, W. N. Mathis (4♂, 2♀; USNM); Topes de Collantes (5 km WNW; 21°56.5'N, 80°2.3'W; 600 m), 11 Dec 1994, W. N. Mathis (2♂, 1♀; USNM). **Guantánamo:** Baracoa (road to; 20°21'N, 74°30.1'W), 28 Feb 1992, M. von Tschirnhaus (1♂, 3♀; USNM). **Havana:** Ojo de Aqua (23°54.6'N, 82°29.1'W), 8 Dec 1994, W. N. Mathis (6♂, 7♀; USNM); San Antonio de los Baños (22°54.9'N, 82°29.3'W), 8 Dec 1994, W. N. Mathis (2♂, 1♀; USNM). **Holguín:** Holguín (N; 20°55.5'N, 76°15.8'W), Feb 1992, M. von Tschirnhaus (1♀; USNM); Santa Lucia (3 km N; 21°03.3'N, 76°59.7'W), 22 Feb 1992, M. von Tschirnhaus (1♂, 1♀; USNM). **Pinar del Rio:** Soroa (22°47.7'N, 83°W), 4–6 Dec 1994, W. N. Mathis (21♂, 13♀; USNM); Soroa (2 km E; 22°47.7'N, 83°W), 29 Apr 1983, W. N. Mathis (1♂, 1♀; USNM); Soroa (2 km NW; 22°48.6'N, 83°1.0'W), 4–5 Dec 1994, W. N. Mathis (4♂; USNM). **Sancti Spiritus:** Topes de Collantes (21°55.2'N, 80°02'W; 350 m), 10 Dec 1994, W. N. Mathis (3♂, 1♀; USNM); Topes de Collantes (21°54.4'N, 80°01.4'W; 670 m), 9–11 Dec 1994, W. N. Mathis (1♂, 3♀; USNM).

DOMINICA. Cabrits Swamp (15°35'N, 61°29'W), 2225 Mar-19 Jun 1989, 1991, D. and W. N. Mathis (9♂, 7♀; USNM); Clarke Hall (15°24.3'N, 61°24.1'W), 1–8 Aug 1964, T. J. Spilmann (1♂; USNM); Coulibistri (15°27.4'N, 61°26.9'W), 21 Mar 1989, W. N. Mathis (2♂; USNM); Dublanc (15°30.9'N, 61°29.8'W), 21 Mar 1989, W. N. Mathis (43♂, 10♀; USNM); Hampstead River (15°34.5'N, 61°22.3'W), 22 Mar 1989, W. N. Mathis (3♂, 1♀; USNM); Rosalie (15°22.3'N, 61°15.3'W), 23 Mar 1989, W. N. Mathis (2♂, 5♀; USNM); Toucari (15°36.6'N, 61°27.8'W), 21 Mar 1989, W. N. Mathis (8♂, 4♀; USNM); Toucari (2 km S; 15°36.3'N, 61°27.7'W), 21 Mar 1989, W. N. Mathis (8♂, 10♀; USNM).

DOMINICAN REPUBLIC. **Azua:** near Pueblo Viejo (18°24.8'N, 70°44.7'W), 19 May 1998, D. and W. N. Mathis (4♂, 1♀; USNM); Padre Las Casas (8 km NE; 18°46'N, 70°53'W; 580 m), 3–4 Oct 1991, R. Davidson, J. E. Rawlins, S. Thompson, C. Young (3♂; CMP). **Barahona:** Cabral (canals E of Cabral; 18°15.2'N, 71°13.4'W), 16 May 1995, W. N. Mathis (1♂; USNM); Cortico, La Mina (18°06.7'N, 71°13.4'W; 1300 m), 23 Mar 1999, W. N. Mathis (15♂, 2♀; USNM); Los Patos (17°57.6'N, 71°10.9'W), 15 May 1995, W. N. Mathis (5♂, 1♀; USNM); Ojeda (17°58.2'N, 71°10.6'W), 22 Mar 1999, W. N. Mathis (1♂; USNM); Paraíso (5 km N; 18°01.5'N, 71°11.6'W; 150 m), 21 Mar 1999, W. N. Mathis (1♂; USNM). **Dajabon:** Loma de Cabrera (10 km S; 19°20'N, 71°37'W; 650 m), 12 Jul 1992, R. Davidson, J. E. Rawlins, S. Thompson, C. Young (19♂, 7♀; CMP). **El Seibo:** Pedro Sáchez (18°51.4'N, 69°6.5'W), 26 May 1998, D. and W. N. Mathis (2♂; USNM); Rincón (near; 18°45.3'N, 68°55.7'W), 12 May 1995, W. N. Mathis (1♂, 3♀; USNM). **Hato Mayor:** Hato Mayor (5.5 km E; 18°46.4'N, 69°12.5'W), 26 May 1998, D. and W. N. Mathis (1♂; USNM). **Independencia:** Duvergé (2 km S; 18°22'N, 71°31.4'W), 24 Mar 1000, W. N. Mathis (14♂, 1♀; USNM); La Descubierta (18°34.1'N, 71°43.8'W), 25 Mar 1999, W. N. Mathis (12♂, 1♀; USNM); Los Bolos (18°37.8'N, 71°39.2'W; 1050 m), 25 Mar 1999, W. N. Mathis (1♀; USNM); Puerto Escondido (18°19.6'N, 71°35'W; 1370 m), 24 Mar 1999, W. N. Mathis (9♂, 1♀; USNM). **La Altagracia:** Boca de Chavon (7 km NNE; Río Chavon; 18°28'N, 68°52'W; 20 m), 3 Jul 1992, R. Davidson, J. E. Rawlins, S. Thompson, C. Young (1♀; CMP). **La Vega:** Constanza (ca. 14 km SE; 18°51.4'N, 70°41.2'W; 1505 m), 15 May 1998, D. and W. N. Mathis (1♂; USNM); El Rio (9.5 km E; 19°0.9'N, 70°33.5'W; 980 m), 6–24 May 1995, 1997, W. N. Mathis (2♂, 1♀; USNM); Jarabacoa (1–2 km S; 19°06.9'N, 70°37'W; 520 m), 8–21 May 1995, 1997, W. N. Mathis (5♂, 7♀; USNM); La Cienega de Manabao (19°03.9'N, 70°51.8'W; 1050 m), 28 Mar 1999, W. N. Mathis (1♂; USNM); Loma del Casabito (19°03'N, 71°31'W; 1390 m), 3 Nov 2002, J. E. Rawlins, C. Staresinic, Thompson, C. Young, W. A. Zanol (1♀; CMP); Río Camu (3.5 km NW La Vega; 19°13.7'N, 70°35.2'W; 100 m), 10–18 May 1995, 1997, W. N. Mathis (6♂; USNM). **Pedernales:** Cabo Rojo (30 km N; 18°07'N, 71°39'W; 1070 m), 23–24 Jul 1990, J. E. Rawlins, S. Thompson, C. Young (10♂, 9♀; CMP); Cabo Rojo (26 km N; 18°06'N, 71°38'W; 730 m), 31 Jul 1990, J. E. Rawlins, S. Thompson, C. Young (3♂, 5♀; CMP); La Abeja (38 km NNW Cabo Rojo; 18°09'N, 71°38'W; 1160 m), 13 Jul 1987, J. E. Rawlins, R. Davidson (2♀; CMP); Río Mulito (13 km N Pedernales; 18°09'N, 71°46'W; 230 m; riparian woodland), 17 Jul 1990, J. E. Rawlins, S. Thompson, C. Young (1♂; CMP). **Puerto Plata:** Rio Camu (14 km E Puerto Plata; 19°41.9'N, 70°37.5'W), 23 May 1998, D. and W. N. Mathis (2♂; USNM); Río Pérez (near Imbert; 19°44.1'N, 70°50.2'W), 24 May 1998, D. and W. N. Mathis (4♂; USNM). **San Cristobal:** Río Haina (18°25.9'N, 70°00.4'W), 27 May 1998, D. and W. N. Mathis (6♂, 2♀; USNM).

GRENADA. **St. Andrew:** Balthazar (12°07.7'N, 61°39.3'W), 15 Sep 1997, W. N. Mathis (3♂; USNM); Grand Étang (lake; 12°05.6'N, 61°41.7'W), 14 Sep 1997, W. N. Mathis (14♂, 3♀; USNM); La Force Bridges (12°07.6'N, 61°39.8'W), 13 Sep 1997, W. N. Mathis (7♂, 2♀; USNM). **St. George:** Grand Anse (12°01.3'N, 61°45.6'W), 15 Sep 1996, W. N. Mathis (5♂, 3♀; USNM); Point Salines Airport (W end; 12°00.3'N, 61°47.7'W), 12–19 Sep 1996, 1997, W. N. Mathis (9♂, 3♀; USNM); True Blue Beach (11°59.9'N, 61°46.1'W), 15 Sep 1996, W. N. Mathis (2♂, 3♀; USNM); Vendôme (1 km E; 12°04.8'N, 61°42.2'W), 17 Sep 1996, W. N. Mathis (1♂, 1♀; USNM). **St. John:** Concord Falls (12°07.1'N, 61°43'W), 12–21 Sep 1996, 1997, W. N. Mathis (5♂, 3♀; USNM); Concord Valley (12°06.9'N, 61°43.9'W), 14 Sep 1996, W. N. Mathis (1♂; USNM); Palmiste (12°08.7'N, 61°44.4'W), 21 Sep 1996, W. N. Mathis (11♂, 1♀; USNM); Palmiste Lake (12°08.3'N, 61°44'W), 19 Sep 1996, W. N. Mathis (2♂, 1♀; USNM). **St. Patrick:** Bathway Beach (12°12.6'N, 61°36.7'W), 13–20 Sep 1996, 1997, W. N. Mathis (23♂; USNM).

JAMAICA. **Clarendon:** Grantham (18°09.3'N, 77°23.8'W; 340 m), 16 Apr 2000, W. N. Mathis (5♂, 2♀; USNM). **Manchester:** Mandeville (18°03.5'N, 77°31.9'W), 7–13 May 1996, D. and W. N. Mathis, H. B. Williams (1♂; USNM). **Portland:** Berridale (18°06.5'N, 76°20'W), Rio Grande River, 25 Apr 2000, W. N. Mathis (2♂; USNM); Hollywell (18°05.5'N, 76°43.6'W; 70 m), 27 Apr 2000, W. N. Mathis (2♂; USNM); Long Bay (2.3 km W; 18°06.5'N, 76°20'W), 24 Apr 2000, W. N. Mathis (7♂, 2♀; USNM); Reach Falls (Drivers River; 18°01.9'N, 76°18.7'W; 70 m), 25 Apr 2000, W. N. Mathis (1♂; USNM); Reach (4 km N; 18°03.6'N, 76°20.4'W), 15 May 1996, D. and W. N. Mathis, H. B. Williams (5♂; USNM). **St. Andrew:** Cinchona (18°04.4'N, 76°39.3'W; 1400 m), 29 Apr 2000, W. N. Mathis (6♂, 1♀; USNM); Clydsdale (18°04.9'N, 77°40.2'W; 1030 m), 29 Apr 2000, W. N. Mathis (1♂, 2♀; USNM); Mavis Bank (1.7 km E; 18°02.4'N, 77°39.5'W; 575 m), Yallahs River, 21–22 Apr-1 May 2000, W. N. Mathis (13♂, 6♀; USNM); Silver Hill Gap (18°05.1'N, 76°41.2'W; 980 m), 26 Apr 2000, W. N. Mathis (1♀; USNM); Silver Hill Gap (18°05.3'N, 76°43'W; 940 m), 29 Apr 2000, W. N. Mathis (1♂; USNM). **St. Ann:** Runaway Bay (18°27.4'N, 77°19.6'W), Feb 1969, W. W. Wirth (1♂, 1♀; USNM). **St. Elizabeth:** Brae River (2 km S; 18°04.2'N, 77°39.5'W), 10 May 1996, D. and W. N. Mathis, H. B. Williams (9♂, 2♀; USNM); Elim (18°07.1'N, 77°40.5'W), 14 Apr 2000, W. N. Mathis (3♂, 1♀; USNM); near Port Kaiser (17°52.3'N, 77°34.9'W), 8 May 1996, D. and W. N. Mathis, H. Williams (1♀; USNM). **St. Mary:** Annotto Bay (18°16.2'N, 76°46.2'W), 25 Feb 1969, W. W. Wirth (2♂; USNM). **St. Thomas:** Bath Fountain Spring (17°57.6'N, 76°21.3'W), 15 May 1996, D. and W. N. Mathis, H. B. Williams (1♂; USNM); Bath River, Bath (17°56.8'N, 76°21.6'W), 16 May 1996, D. and W. N. Mathis, H. B. Williams (3♂; USNM). **Trelawny:** Rio Bueno (18°28.7'N, 77°28.3'W), 21 Feb 1969, W. W. Wirth (1♂; USNM). **Westmoreland:** Negril Beach (8 km E; 18°16.4'N, 78°21.2'W; fresh marsh), 12 Mar 1970, W. W. Wirth (1♂, 5♀; USNM); Negril Beach (18°16.4'N, 78°21.2'W; mangrove, rocky shore), 13 Mar 1970, W. W. Wirth (1♂; USNM).

PUERTO RICO. Adjuntas (18°09.8'N, 66°43.2'W), 22 Sep 1995, D. and W. N. Mathis (2♀; USNM); Arroyo (17°56.9'N, 66°03.7'W), Feb 1899, A. Busck (1♀; paratype; USNM); Fajardo (18°19.5'N, 65°39.1'W), Feb 1899, A. Busck (1♂, 1♀; paratypes; USNM); Fajardo, Las Crosbas, Seven Seas Beach (18°23'N, 65°37'W), 17 Feb 1996, W. E. Steiner, J. M. Swearingen (9♂, 2♀; USNM); Maricao (18°11.1'N, 66°58.9'W), 21 Sep 1995, D. and W. N. Mathis (2♂, 1♀; USNM). Playa de Guayanilla (18°0.4'N, 66°46.1'W), 19 Sep 1995, D. and W. N. Mathis (4♂, 1♀; USNM); Utuado (18°15.9'N, 66°42'W), Jan 1899, A. Busck (1♂; paratype; USNM).

ST. LUCIA. Anse la Raye, Anse Galet (1 km SSW Anse la Raye; 13°56'N, 61°03'W; 50 m), 21–30 Jun 1991, J. E. Rawlins, S. A. Thompson (3♂; CMP); Castries (13°59.6'N, 61°0.4'W), Oct 1967, N. L. H. Krauss (2♂; USNM); Castries (5 km S; 13°59'N, 60°00'W), 16 Jun 1991, D. and W. N. Mathis (4♂, 3♀; USNM); Dauphin Boguis (1.6 km S Marquis; 14°01'N, 60°55'W), 17 Jun 1991, D. and W. N. Mathis (3♂, 2♀; USNM); Fond St. Jacques (13°50'N, 61°02'W), 13–14 Jun 1991, D. and W. N. Mathis (9♂, 12♀; USNM); Micoud (13°49'N, 60°54'W), 15 Jun 1991, D. and W. N. Mathis (4♂; USNM); Soufrière Botanical Garden (13°51'N, 61°04'W), 12 Jun 1991, D. and W. N. Mathis (33♂, 11♀; USNM); Sulphur Spring (13°50'N, 61°03'W), 14 Jun 1991, D. and W. N. Mathis (3♂, 1♀; USNM).

ST. VINCENT. **Charlotte:** Montreal (13°12'N, 61°11'W), 26 Mar-3 Sep 1989, 1991, 1997, A. Freidberg, D. and W. N. Mathis (18♂, 5♀; USNM); South Rivers (13°14.6'N, 61°09.3'W), 8 Sep 1997, W. N. Mathis (6♂, 1♀; USNM); Yambou River (13°09.8'N, 61°08.7'W), 8–10 Sep 1997, W. N. Mathis (1♂; USNM). **St. Andrew:** Buccament Bay (near beach; 13°11'N, 61°16'W), 25–28 Mar 1989, W. N. Mathis (2♂, 5♀; USNM); Camden Park (13°10.2'N, 61°14.7'W), 4 Sep 1997, W. N. Mathis (3♂; USNM); Layou (13°12'N, 61°17'W), 8 Jun 1991, D. and W. N. Mathis (2♀; USNM); Vermont (13°13'N, 61°13'W), 5–8 Sep 1997, W. N. Mathis (9♂, 1♀; USNM). **St. George:** Kingston, Botanical Garden (13°9.7'N, 61°13.7'W), 25–27 Mar 1989, W. N. Mathis (4♂; USNM); Yambou Head (13°09.8'N, 61°08.6'W), 27 Mar 1989, W. N. Mathis (1♀; USNM). **St. Patrick:** Cumberland River (3 km E Spring Village; 13°15'N, 61°14'W), 10 Jun 1991, D. and W. N. Mathis (5♂, 7♀; USNM); Hermitage (13°15'N, 61°12.9'W), 9 Sep 1997, W. N. Mathis (6♂, 2♀; USNM); Palmiste Park (13°12.7'N, 61°14.9'W), 5 Sep 1997, W. N. Mathis (15♂, 2♀; USNM); Wallilabou (beach; 13°15'N, 61°16'W), 27 Mar-8 Jun 1989, 1991, D. and W. N. Mathis (2♀; USNM).

##### Distribution

**Figure 95.**
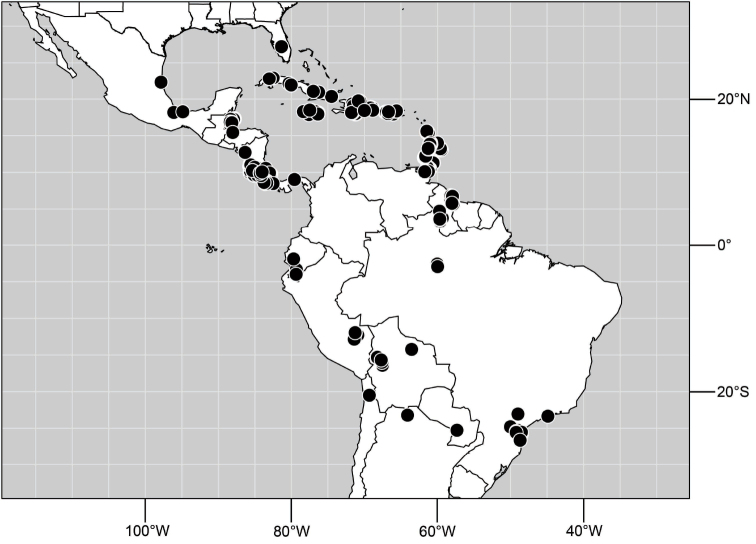
Distribution of *Hydrochasma incisum* (Coquillett).

(Fig. 95). Nearctic: United States (Florida). Neotropical: Argentina (Salta), Belize (Belize, Stann Creek), Bolivia (Beni, La Paz), Brazil (Amazonas, Paraná, Rio de Janeiro, Santa Catarina, São Paulo), Chile (Tarapacá), Costa Rica (Cartago, Guanacaste, Heredia, Limón, Puntarenas, San José), Ecuador (Azuay, Guayas, Loja), Guyana, Honduras (Cortés), Mexico (Tamaulipas, Veracruz-Llave), Nicaragua (León), Panama (Panama), Paraguay (Cordillera), Peru (Cuzco, Madre de Dios), Trinidad and Tobago, West Indies (Barbados, Cuba, Dominica, Dominican Republic, Grenada, Jamaica, Puerto Rico, St. Lucia, St. Vincent).

##### Remarks.

This is a widespread and common species in much of the Neotropics. We have relied primarily on structures of the male terminalia to accurately identify this species, especially the elongated, slender, ventral epandrial process, which distinguishes this species. This structure is often partially or wholly exposed, allowing for identification in dried, pinned males.

We have observed slight variation in some specimens, usually whether the aedeagal apex is slightly inflated or not, but also in the length of the ventral epandrial process. We interpret this variation to be intraspecific, which may be an artifact in part of how specimens were preserved.

#### 17. Hydrochasma kaieteur sp. n.

http://zoobank.org/2DFE442B-EA52-4264-9A1B-F2BE46D0EFFB

http://species-id.net/wiki/Hydrochasma_kaieteur

Figs 96 –100

##### Diagnosis.

**Figures 96–99.**
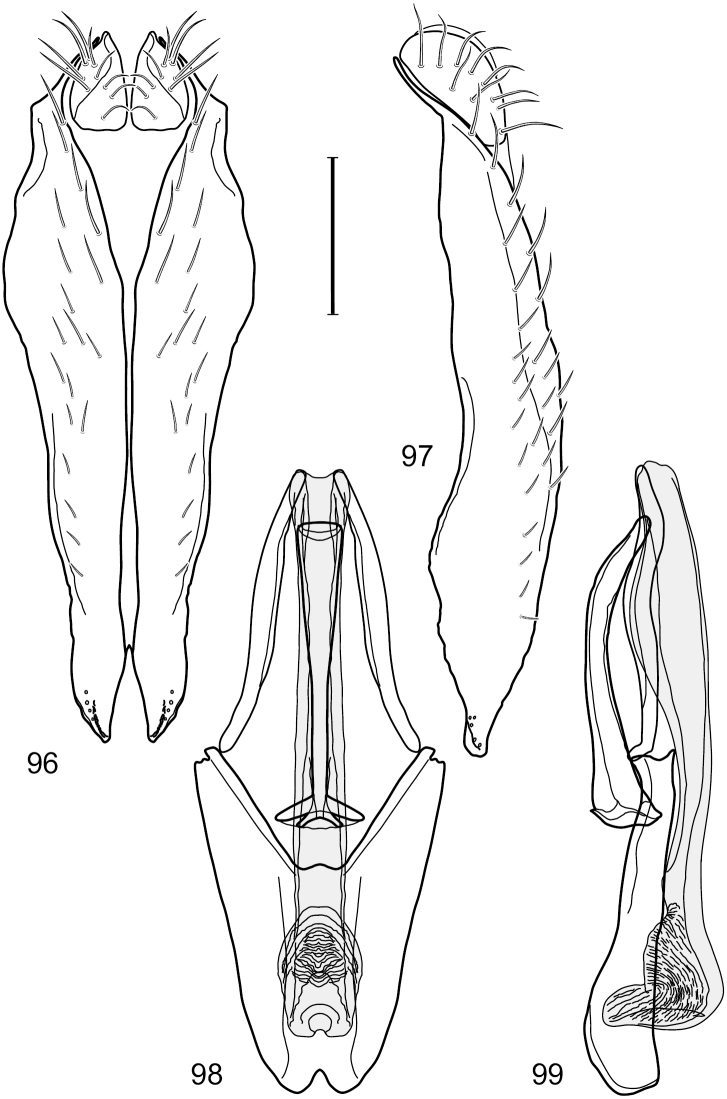
*Hydrochasma kaieteur* sp. n. (Guyana: Dubalay Ranch, Berbice River) **96** epandrium and cerci, posterior view **97** same, lateral view **98** internal structures of male terminalia (aedeagus [shaded], phallapodeme, gonite, hypandrium), ventral view **99** same, lateral view. Scale bar = 0.1 mm.

This species is distinguished from other congeners by the following combination of characters: Small shore flies, body length 1.25–1.80 mm. *Head*: Antenna mostly dark gray; parafacial silvery white, concolorous with facial coloration; gena-to-eye ratio 0.17–0.18. *Thorax*: Wing with costal vein ratio 0.65–0.67; M vein ratio 0.54–0.58. Forecoxa mostly gray to silvery gray, some yellowish coloration at ventral apex; forefemur lacking row of spine-like setulae along anteroventral surface; tibiae mostly gray; hindtibia lacking a long, spur-like seta ventroapically. *Abdomen*: Tergites 3–4 with moderately deep, gray to silvery gray wedges at lateral margins; tergite 5 of male mostly to entirely gray, sometimes with posterior margin darkened. Male terminalia (Figs 96–99): Epandrium generally elongate, almost 3 times longer than wide, and setulose, in posterior view (Fig. 96) with dorsal arch attenuate, not connected, dorsal 1/3 somewhat rectangular, ventral 2/3 tapered with lateral margins shallowly sinuous to a pointed ventral margin, ventromedial apex incised, narrowly V-shaped, in lateral view (Fig. 97) very narrowly developed dorsally laterad of cerci, thereafter ventrally becoming slightly wider to midlength, then becoming wider at ventral 1/3, before becoming tapered to pointed ventral margin; cerci short, length less than twice width, in posterior view (Fig. 96) hemispherical with pointed dorsal apex and truncate ventral margin, in lateral view (Fig. 97) almost evenly semicircular; aedeagus in lateral view (Fig. 99) relatively simple, narrowly tubular except for expanded, mostly membranous apex, length 6–7× width, in ventral view (Fig. 98) tubular; phallapodeme in lateral view (Fig. 99) spatulate with width of keel equal to width of phallapodeme, keel or spatulate end at end toward hypandrium, rest of phallapodeme tapered, curved subapically toward aedeagus, in ventral view (Fig. 98) linear, with slightly and narrowly developed T-shaped extensions; gonite in lateral view (Fig. 99) bar-like, narrowly linear, sinuous, in ventral view (Fig. 98) short, somewhat shallowly zig-zag; hypandrium in lateral view (Fig. 99) robustly bar-like width about equal to aedeagus, in ventral view (Fig. 98) somewhat rectangular with broadly and deeply emarginate posterior margin and with anterior margin as 2 blunted rounded processes and a moderately deep and narrowly incised medial portion.

##### Type material.

The holotype male of *Hydrochasma kaieteur* is labeled “**GUYANA.** Kaieteur Fal[l]s 5°10.5'N, 59°26.9'W[,] 21–24 August 1997[,] Wayne N. Mathis/USNM ENT 00080329 [plastic bar code label]/HOLOTYPE ♂ *Hydrochasma kaieteur* Mathis & Zatwarnicki, USNM [red].” The holotype is double mounted (minuten in a block of plastic), is in excellent condition, and is deposited in the USNM. Thirty paratypes (20♂, 10♀; USNM) bear the same label data as the holotype.

##### Type locality.

Guyana. Kaieteur Falls (05°10.5'N, 59°26.9'W).

##### Other specimens examined.

Neotropical. ARGENTINA. **Formosa:** Ingeniero Juárez (23°54.7'S, 61°51.8'W), 2 Jan 1949, R. Golbach (1♂; USNM).

BRAZIL. **Paraná:** Antonina (25°28.4'S, 48°40.9'W; beach/mangal), 9 Apr 2010, D. and W. N. Mathis (3♂; DZUP, USNM); Antonina (25°27.1'S, 48°41.1'W; beach; Ponta da Pita), 15 Feb 2010, D. and W. N. Mathis (3♂; DZUP, USNM); Matinhos (N.; 25°47.4'S, 48°31.6'W; 1 m; beach/estuary), 30 Jan-9 Apr 2010, D. and W. N. Mathis (3♂; DZUP, USNM); Morretes (25°28'S, 43°59.1'W), 29 Aug 2000, D. and W. N. Mathis (14♂, 7♀; USNM); Paranaguá (Rio Itiberê; 25°31.4'S, 48°30.3'W; 3 m), 23 Jan 2010, D. and W. N. Mathis (12♂, 2♀; DZUP, USNM); Prainha (5 km S Matinhos; 25°51.2'S, 48°33.6'W; beach), 15 Nov 2010, D. and W. N. Mathis (11♂; DZUP, USNM). **Santa Catarina:** Barra Velha (26°38'S, 48°40.9'W; beach), 29 Apr 2010, D. and W. N. Mathis (2♂; DZUP, USNM).

GUYANA. Conservation of Ecological Interactions and Biotic Associations (CEIBA; ca. 40 km S Georgetown; 06°29.9'N, 58°13.1'W), 13–21 Apr 1994, 1995, W. N. Mathis (1♀; USNM); Karanambo (Rupununi River; 03°45.1'N, 59°18.6'W; 85m), 1–3 Dec 2010, W. N. Mathis (20♂; USNM); Karanambo, Rupununi River (ox bow; 03°45'N, 59°17.5'W; 85m), 2 Dec 2010, W. N. Mathis (1♂; USNM); Kato, Chiung River (04°39.7'N, 59°50.0'W), 1 May 1995, W. N. Mathis (8♂, 2♀; USNM) Kumo Pond (20 km S Lethem; 03°16.7'N, 59°43.5'W), 30 Apr 1995, W. N. Mathis (2♀; USNM); Kumu River and Falls (25 km SE Lethem in Kanuku Mountains; 03°15.9'N, 59°43.6'W), 28–30 Apr 1995, W. N. Mathis (2♂, 5♀; USNM); Mahaica (3 km E; 06°43.5'N, 57°56.6'W), 14 Apr 1994, W. N. Mathis (1♂, 5♀; USNM); Mahaica (06°42.8'N, 57°55.6'W), 14–22 Apr 1994, 1995, W. N. Mathis (8♂, 14; USNM); Menzies Landing (05°10.1'N, 59°29.5'W), 23 Aug 1997, W. N. Mathis (7♂, 4♀; USNM); Paramakatoi (04°42'N, 59°42.8'W), 24–25 Aug 1997, W. N. Mathis (6♂, 2♀; USNM).

##### Distribution

**Figure 100.**
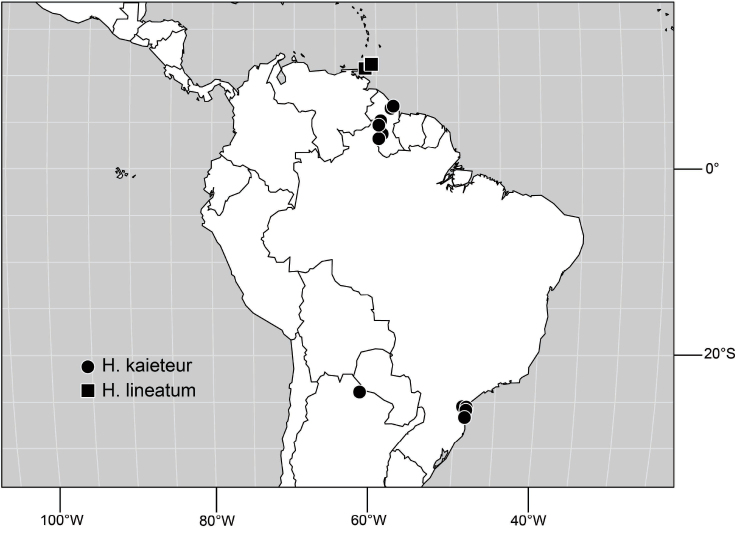
Distribution of *Hydrochasma kaieteur* sp. n. and *Hydrochasma lineatum* sp. n.

(Fig. 100). Neotropical: Argentina (Formosa), Brazil (Paraná, Santa Catarina), Guyana.

##### Etymology.

The species epithet, *kaieteur*, is the name of the world-famous falls in Guyana where this species was collected. The name is a noun in apposition.

##### Remarks.

This species is distinguished from congeners, especially those of the *incisum* group, by the shape of structures of the male terminalia, in particular the epandrium in posterior view and the robust, V-shaped hypandrium in ventral view.

#### 18. Hydrochasma miguelito sp. n.

http://zoobank.org/19737E27-1ED5-4814-A2DD-8D0AFB3AEBE6

http://species-id.net/wiki/Hydrochasma_miguelito

Figs 101 –105

##### Diagnosis.

**Figures 101–104.**
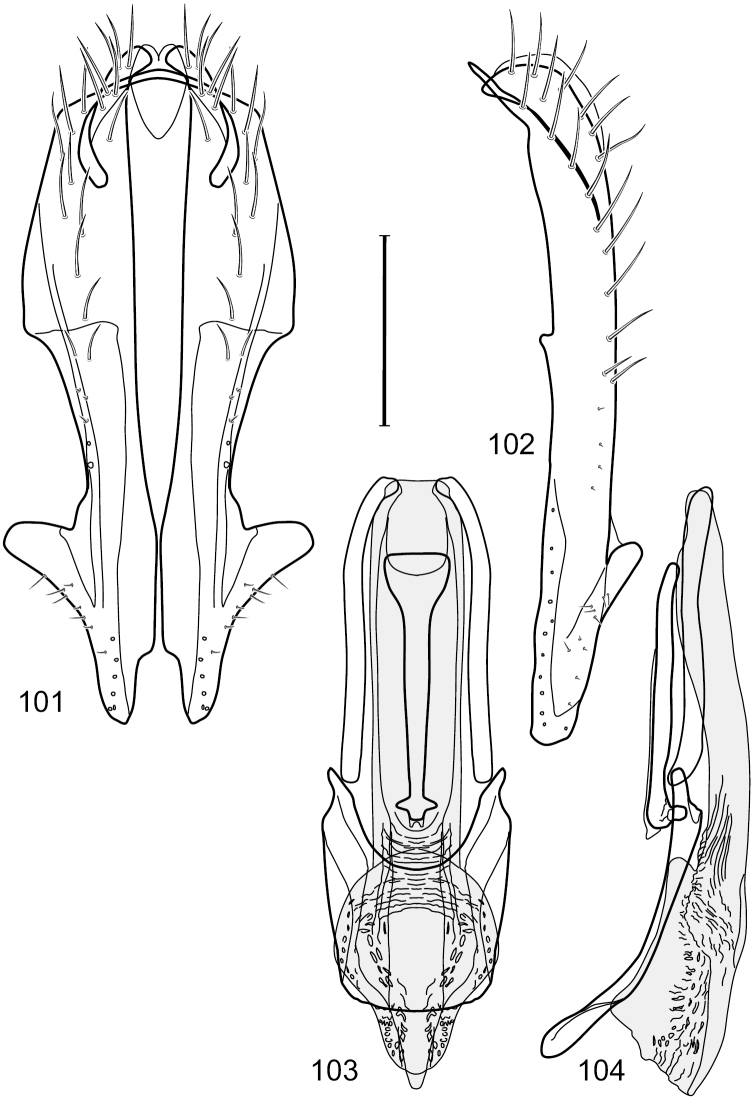
*Hydrochasma miguelito* sp. n. (Honduras. Cortés: San Pedro Sula) **101** epandrium and cerci, posterior view **102** same, lateral view **103** internal structures of male terminalia (aedeagus [shaded], phallapodeme, gonite, hypandrium), ventral view **104** same, lateral view. Scale bar = 0.1 mm.

This species is distinguished from congeners by the following combination of characters: Small to moderately small shore flies, body length 1.60–2.05 mm. *Head*: Not subglobose or broad, oral opening small. At least pedicel black, otherwise antennal coloration variable; parafacial silvery white, concolorous with facial coloration; gena-to-eye ratio 017–0.18. *Thorax*: Mesonotum grayish tan to golden brown. Wing with costal vein ratio 0.66–0.68; M vein ratio 0.51–0.54. Forecoxa mostly silvery gray to gray with some yellowish coloration at ventral apex; forefemur lacking a distinctive, comb-like row of stout setulae along anteroventral surface; tibiae mostly gray; hindtibia lacking a long, spur-like seta ventroapically. *Abdomen*: Tergites 3–4 with deep, gray to silvery gray wedges at lateral margins; tergite 5 of male mostly to entirely gray, sometimes with posterior margin darkened. Male terminalia (Figs 101–104): Combined structures generally moderately elongate, in posterior view height about twice width; epandrium with dorsal arch narrowly connected above cerci; dorsal half more or less generally sparsely setulose, ventral half with sparse, very short setulae; in posterior view (Fig. 101) as an inverted, angulate, moderately narrow U, ventral epandrial extension narrowed to just before greatly expanded, apical third, apical portion broad as an arrow point with broad, medial incision apically, in lateral view (Fig. 102) narrowly elongate, parallel-sided except for extended base of arrow point; aedeagus in lateral view (Fig. 104) elongate, about 5× longer than greatest width, tubular, becoming wider and more membranous apically, in ventral view (Fig. 103) with basal 2/3 narrow, parallel sided, apical third abruptly enlarged, bulbous, slightly tapered apically; phallapodeme in lateral view (Fig. 104) narrowly elongate, almost parallel sided, hypandrial end slightly expanded, in ventral view (Fig. 103) narrowly I-shaped with expanded end toward aedeagus and with subapical, short crossbar at hypandrial end; gonite in lateral view (Fig. 104) narrowly elongate, very shallowly sinuous, bar-like, in ventral view (Fig. 103) more conspicuously sinuous; hypandrium in lateral view (Fig. 104) narrowly elongate, shallowly curved, in ventral view (Fig. 103) with anterior 2/3 somewhat quadrate, anterior margin broadly truncate, posterior margin deeply, widely emarginate with short, lateral apices.

##### Type material.

The holotype male of *Hydrochasma miguelito* is labeled “**HONDURAS.** Cortés: San Pedro Sula (8 km S)[,] 15°25.7'N, 88°01.4'W[,] 25–26 September1995[,] Dianne & W.N.Mathis/USNM ENT 00138962 [plastic bar code label]/HOLOTYPE ♂ *Hydrochasma miguelito* Mathis & Zatwarnicki, USNM [red].” The holotype is double mounted (minuten in a block of plastic), is in excellent condition, and is deposited in the USNM. Seven paratypes (3♂, 4♀; USNM) bear the same label data as the holotype.

##### Type locality.

Honduras. Cortés: San Pedro Sula (8 km S; 15°25.7'N, 88°01.4'W).

##### Other specimens examined.

Neotropical. COSTA RICA. **Guanacaste:** Nandayure, Río Morote, Finca Palmichal (10°03'N, 85°12'W; 0–50 m), 26 Mar 2006, W. Porras (1♂; INBio). **Puntarenas:** Bosque Esquinas (08°44'N, 83°17'W; 200 m), May 1994, M. Segura (1♂; INBio); Jacó (5 km E; 9°34.7'N, 84°35.6'W), 10 Jun 2003, D. and W. N. Mathis (4♂; USNM).

##### Distribution

**Figure 105.**
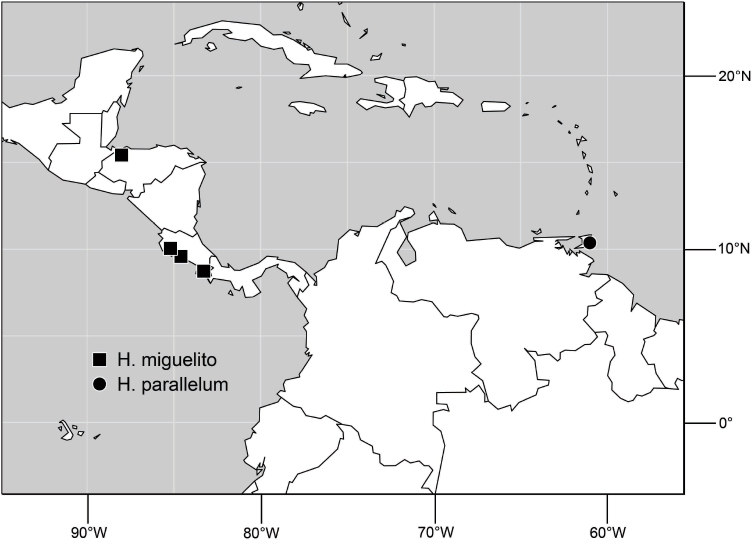
Distribution of *Hydrochasma miguelito* sp. n. and *Hydrochasma parallelum* sp. n.

(Fig. 105). Neotropical: Costa Rica (Guanacaste, Puntarenas), Honduras (Cortés).

##### Etymology.

The species epithet, *miguelito*, is to recognize Michael W. Mathis who is also known as “Don Miguel” or “Miguelito.” Michael guided us while conducting field work in Honduras where this species was collected. We are treating *miguelito* as a noun in apposition.

##### Remarks.

Structures of the male terminalia readily distinguish this species from congeners, especially those of the *incisum* group. Certainly unique to this species is the greatly expanded apical portion of the ventral, epandrial process, which is like a broad arrowhead. The expanded, ventral, epandrial process can be seen in ventral and somewhat in lateral views. The rectangular hypandrium that has a moderately deep and wide posterior emargination is also characteristic.

#### 19. Hydrochasma octogonum sp. n.

http://zoobank.org/2252598F-643F-4AF1-BAAD-73AA37204B09

http://species-id.net/wiki/Hydrochasma_octogonum

Figs 106 –110

Hydrochasma incisum of authors, *nec* Coquillett (misidentification). Cresson 1942: 113 [generic combination; list Florida]. Mathis and Zatwarnicki 1995: 182–183 [in part; world catalog].

##### Diagnosis.

**Figures 106–109.**
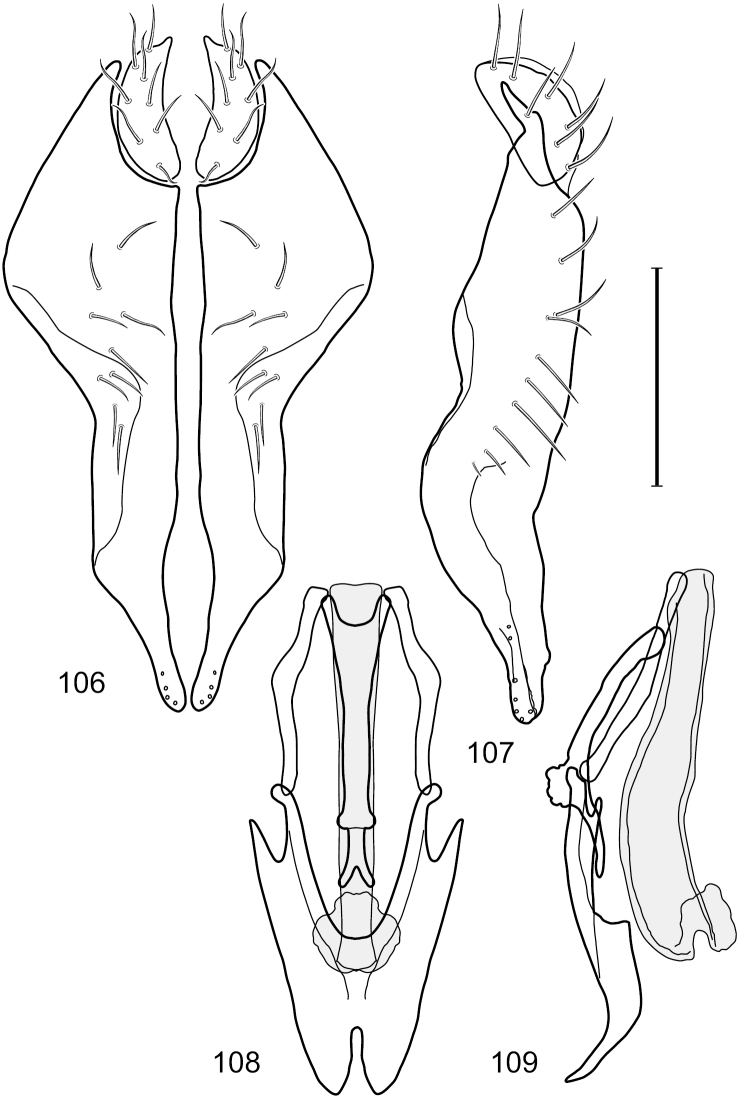
*Hydrochasma octogonum* sp. n. (Ecuador. Manabí: Pichincha) **106** epandrium and cerci, posterior view **107** same, lateral view **108** internal structures of male terminalia (aedeagus [shaded], phallapodeme, gonite, hypandrium), ventral view **109** same, lateral view. Scale bar = 0.1 mm.

This species is distinguished from other congeners by the following combination of characters: Small shore flies, body length 1.25–1.80 mm. *Head*: Antenna mostly yellowish, at most with dorsal surface of pedicel and basal flagellomere dark; face, parafacial, and gena almost unicolorous, yellowish tan to tan, not contrasted; gena-to-eye ratio 0.23–0.25. *Thorax*: Wing with costal vein ratio 0.79–0.81; M vein ratio 0.0.49–0.53; apex of wing acutely pointed, faintly infuscate at apex. Forecoxa mostly gray to whitish gray to yellowish toward apex; forefemur lacking a comb-like row of stout setulae along anteroventral surface; tibiae mostly gray. *Abdomen*: Tergites 3–4 with distinct, lateral, silvery-gray wedges, wedge on tergite 3 frequently very shallow, little evident, wedge on tergite 4 frequently narrow; tergite 5 of male mostly gray, with posterior margin darkened. Male terminalia (Figs 106–109): Combined structures generally elongate, in posterior view height about twice width, sparsely setulose; epandrium with dorsal arch above cerci attenuated, not connected, in posterior view (Fig. 106) with dorsal half forming a diamond with a U-shaped cercal cavity dorsally, ventral portion with each lateral half robustly developed, parallel sided then apical nearly half tapered to ventral apex, apex pointed, narrowly incised medially along entire length from cercal cavity to apex, in lateral view (Fig. 107) very shallow L-shaped, obtuse angle, ventral 1/3 tapered to narrowly rounded apex; cerci moderately short, height more than twice width, widely semi-hemispherical (Fig. 106), pointed dorsally, not attached lateroventrally or ventrally with epandrium; aedeagus in lateral view (Fig. 109) very elongate, almost 6× longer than width, tubular, shallowly curved, apex rounded and with a subapical rectangular extension, in ventral view (Fig. 108) mostly tapered from base apical, rounded expansion; phallapodeme in lateral view (Fig. 109) narrowly elongate with extended keel small, short and barely extended, skewed, irregularly rectangular on portion toward attachment with hypandrium, in ventral view (Fig. 108) an elongate, moderately narrow Y with arms of Y very short; gonite in lateral view (Fig. 109) narrow, elongate, bar-like, very shallowly and obtusely angulate, in ventral view (Fig. 108) shallowly angulate; hypandrium in lateral view (Fig. 109) moderately elongate, very shallow, tapered to curved point anteriorly, in ventral view (Fig. 108) moderately broadly developed, posterior emargination robustly U-shaped, anterior margin bilobed with medial narrow, short incision.

##### Type material.

The holotype male of *Hydrochasma octogonum* is labeled “ECUADOR. Pich-incha[,] Manabi[,] August 1955/Collr.Levi-Castillo/USNM ENT 00118298 [plastic bar code label]/HOLOTYPE ♂ *Hydrochasma octogonum* Mathis & Zatwarnicki, USNM [red].” The holotype is double mounted (glued to a paper triangle)), is in excellent condition, and is deposited in the USNM. Eight paratypes (6♂, 2♀; USNM) bear the same label data as the holotype. Other paratypes are as follows: ECUADOR. **Guayas:** Río Bobo (01°53.8'S, 79°42'W), Aug 1955, R. Levi-Castillo (3♂, 1♀; USNM).

##### Type locality.

Ecuador. Manabí: Pichincha (01°01'S, 79°49'W).

##### Other specimens examined.

Neotropical. ECUADOR. **Manabí:** La Palma (0°45.7'S, 80°30.7'W), Aug 1955, R. Levi-Castillo (1♂; USNM).

##### Distribution

**Figure 110.**
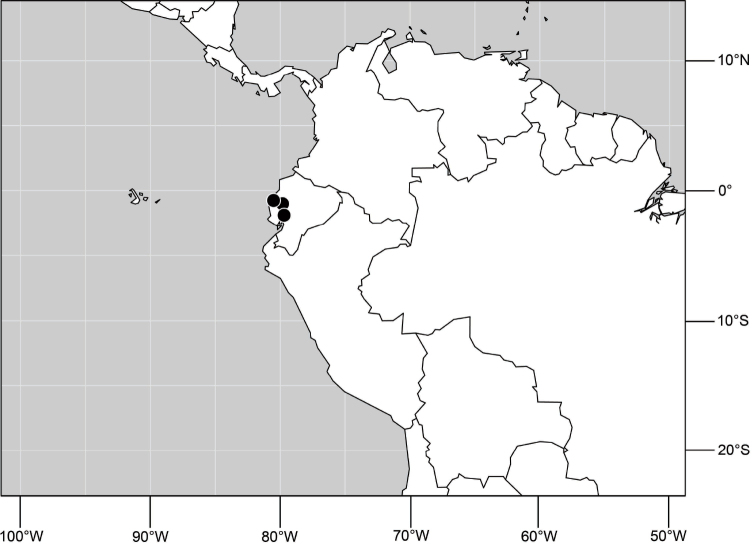
Distribution of *Hydrochasma octogonum* sp. n.

(Fig. 110). Neotropical: Ecuador (Guayas, Manabí).

##### Etymology.

The species epithet, *octogonum*, is of Latin derivation and means eight sided, referring to the eight-sided polygon of the epandrium in posterior view.

##### Remarks.

Externally, this species is very similar to *Hydrochasma incisum* and has often been misidentified as that species in collections. Structures of the male terminalia are quite different, however, and readily distinguish between these two species. In addition, the apex of the wing in *Hydrochasma octogonum* is acutely angulate, and the vertex of the angle, the apex, is faintly to conspicuously infuscate. In specimens of *Hydrochasma incisum*, the apex is narrowly rounded and hyaline, typical of most congeners.

#### 20. Hydrochasma parallelum sp. n.

http://zoobank.org/121FBDE1-8331-4DC8-AA59-E05A77D0D64E

http://species-id.net/wiki/Hydrochasma_parallelum

Figs 105 , 111–114

##### Diagnosis.

**Figures 111–114.**
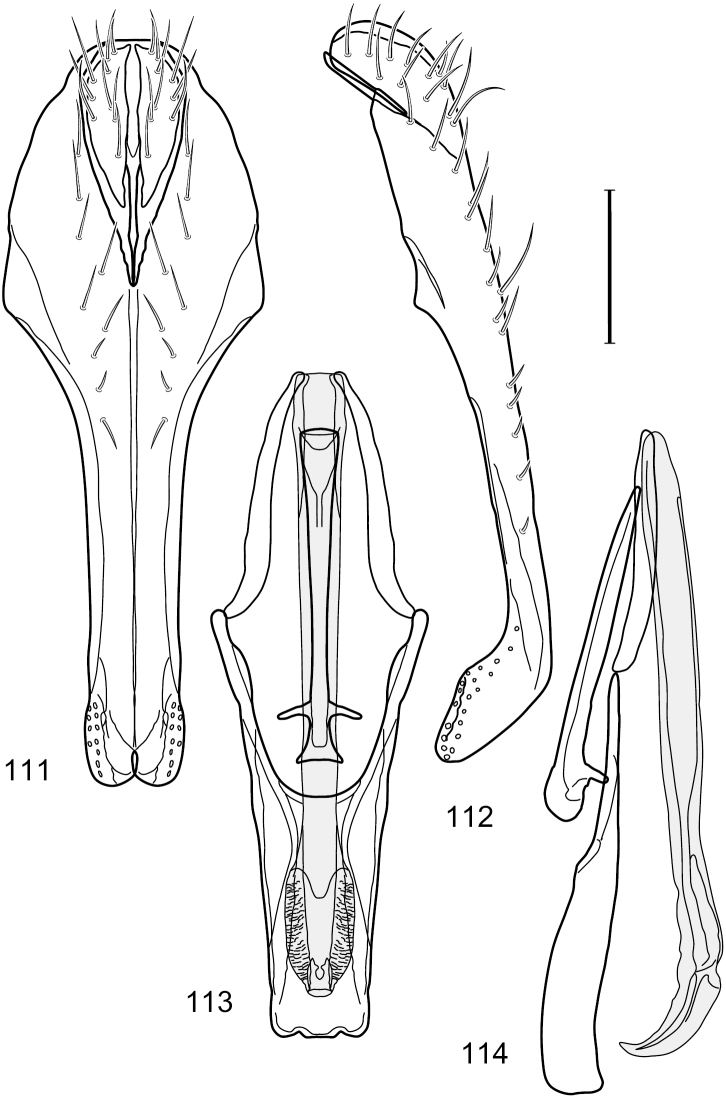
*Hydrochasma parallelum* sp. n. (Trinidad: St. Andrew: Low Manzanilla) **111** epandrium and cerci, posterior view **112** same, lateral view **113** internal structures of male terminalia (aedeagus [shaded], phallapodeme, gonite, hypandrium), ventral view **114** same, lateral view. Scale bar = 0.1 mm.

This species is distinguished from other congeners by the following combination of characters: Small to moderately small shore flies, body length 1.65–2.30 mm. *Head*: Antenna mostly dark gray; parafacial silvery white, concolorous with facial coloration; gena-to-eye ratio 0.17–0.18. *Thorax*: Wing with costal vein ratio 0.77–0.81; M vein ratio 0.47–0.51. *Abdomen*: Tergites with moderately distinctive, deep, gray to silvery gray wedges along lateral margin of darkened coloration; male tergite 5 gray. Male terminalia (Figs 111–114): Combined structures generally moderately elongate, in posterior view height almost 3× width, generally moderately setulose dorsally, setulae sparse or lacking ventrally; epandrium lacking dorsal arch above cerci, in posterior view (Fig. 111) with apical 2/3 abruptly narrowed, parallel sided, apical process not wider than extended process, apex with very narrow apicomedial cleft, in lateral view (Fig. 112) elongate, narrow with basal 3/4 straight, apical 1/4 abruptly curved anteriorly, slight expanded, apex rounded; aedeagus in lateral view (Fig. 114) very elongate, narrow, mostly parallel sided, apical 1/8 curved anteriorly, apex pointed, in ventral view (Fig. 113) very narrow, elongate, apical 1/4 slightly expanded, apex narrowly rounded; phallapodeme in lateral view (Fig. 114) narrow, elongate, rod-like, hypandrial end slight expanded with a subapical, small, digitiform process, thereafter tapered to a point toward aedeagal base, in ventral view (Fig. 113) elongate, narrow, truncate at both ends, with a subapical crossbar; gonite in lateral view (Fig. 114) as a very shallow curved, rod-like process, slightly shorter in length than phallapodeme, in ventral view (Fig. 113) shallowly sinuous, tapered at both apices; hypandrium in lateral view (Fig. 114) narrowly developed, anterior half narrowly rectangular, thereafter posteriorly tapered to a point, in ventral view (Fig. 113) moderately deeply V-shaped, with base of V rectangular, robustly developed, extended lateral arms, thin, elongate, slightly oriented laterally, anterior margin truncate.

##### Type material.

The holotype male of *Hydrochasma parallelum* is labeled “**TRINIDAD.** St. Andr[ew].: Low[er]. Manzanilla (16km S, 10°22'N, 61°01'W)[,] 20Jun1993, WNMathis/USNM ENT 00285975 [plastic bar code label]/HOLOTYPE ♂ *Hydrochasma parallelum* Mathis & Zatwarnicki, USNM [red].” The holotype is double mounted (minuten in a block of plastic), is in excellent condition, and is deposited in the USNM. Five paratypes (1♂, 4♀; USNM) bear the same label data as the holotype.

##### Type locality.

Trinidad and Tobago. Trinidad. St. Andrew: Lower Manzanilla (16 km S; 10°22'N, 61°01'W).

##### Distribution

(Fig. 105). Neotropical: Trinidad and Tobago.

##### Etymology.

The species epithet, *parallelum*, is of Latin derivation and means parallel sided, referring to the parallel sided extensions of the epandrium.

##### Remarks.

Structures of the male terminalia of this species are similar to those of *Hydrochasma dolabrutum* but are distinguished from the latter species by having a comparatively more slender extended epandrial process (best seen in posterior view). Moreover, the hypandrium in general is thinner, more slenderly developed, and the anterior margin is truncate rather than being broadly rounded as in *Hydrochasma dolabrutum*.

#### 21. Hydrochasma peniculum sp. n.

http://zoobank.org/6550C161-49C7-4CFD-9881-E98119F3B1E0

http://species-id.net/wiki/Hydrochasma_peniculum

Figs 115 –119

##### Diagnosis.

**Figures 115–118.**
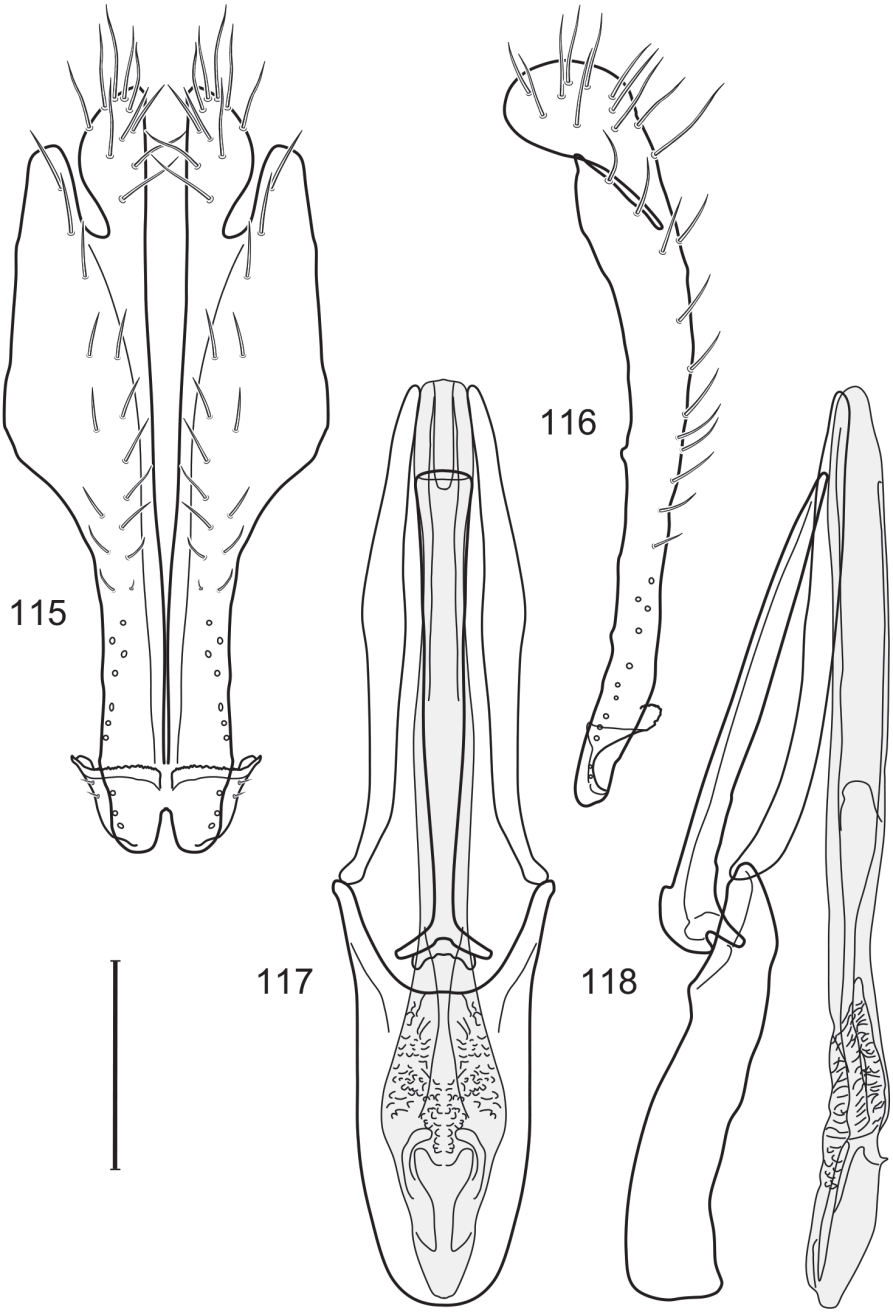
*Hydrochasma peniculum* sp. n. (Cuba. Cienfuegos, Jardin Botánico) **115** epandrium and cerci, posterior view **116** same, lateral view **117** internal structures of male terminalia (aedeagus [shaded], phallapodeme, gonite, hypandrium), ventral view **118** same, lateral view. Scale bar = 0.1 mm.

This species is distinguished from other congeners by the following combination of characters: Small shore flies, body length 1.40–1.90 mm. *Head*: Antenna mostly dark gray; parafacial silvery white, concolorous with facial coloration; gena-to-eye ratio 0.16–0.18. *Thorax*: Wing with costal vein ratio 0.73–0.74; M vein ratio 0.51–0.53. Forecoxa usually mostly yellow (apical 1/2–2/3); tergites 3–4 with lateral wedges, sometimes deeply inset. *Abdomen*: Tergites 3–4 with moderate deep gray wedges at lateral margin of darkened dorsum, wedge of tergite 4 deeper; tergite 5 of male mostly gray, with posterior margin darkened. Male terminalia (Figs 115–118): Combined structures generally moderately elongate, in posterior view height almost 2.5× width, generally setulose dorsally, setulae sparse or lacking ventrally; epandrium lacking a dorsal arch above cerci, in posterior view (Fig. 115) with apical 1/3–1/2 abruptly narrowed, mostly parallel sided, phallic-like with apex expanded, hood-like, apicomedially with narrow, short cleft, in lateral view (Fig. 116) generally very shallowly arched, elongate, narrow, wider basally; cerci moderately short, height nearly twice width, semi-hemispherical (Fig. 115), attached ventrally with epandrium; aedeagus in lateral view (Fig. 118) very elongate, length about 10–12× width, tubular, mostly parallel sided, apex narrowly truncate, in ventral view (Fig. 117) mostly parallel sided on basal 2/3–3/4, apical 1/4–1/3 narrowly diamond-like, expanded subapically, apex narrowly rounded; phallapodeme in lateral view (Fig. 118) narrowly elongate, slightly expanded toward hypandrial end, thereafter gradually tapered to aedeagal base, in ventral view (Fig. 117) an elongate, narrowly T-shaped process with narrow crossbar at hypandrial end, thereafter slightly expanded toward aedeagal base, apex almost truncate; gonite in lateral view (Fig. 118) rod-like with slight arch on basal half, otherwise straight, parallel sided, in ventral view (Fig. 117) very narrow, elongate, widest medially, shallowly flared laterally toward base; hypandrium in lateral view (Fig. 118) elongate, more or less narrowly rectangular with posterior end tapered to a point, in ventral view (Fig. 117) length almost twice width, produced posteriorly as 2 narrow, lateral process, posterior margin moderately deeply emarginate, U-shaped, anterior margin bluntly rounded.

##### Type material.

The holotype male of *Hydrochasma peniculum* is labeled “DOM[INICAN].R[E]P[UBLIC]. Pedernales: Pedernales 18°01.8'N, 71°44.7'W, 19–20 Mar 1999, W. N. Mathis/USNM ENT 00089078 [plastic bar code label]/HOLOTYPE ♂ *Hydrochasma peniculum* Mathis & Zatwarnicki, USNM [red].” The holotype is double mounted (minuten in a block of plastic), is in excellent condition, and is deposited in the USNM. Twenty-seven paratypes (24♂, 3♀; USNM) bear the same label data as the holotype. Other paratypes are as follows: DOMINICAN REPUBLIC. **Barahona:** Baoruco (beach and river; 18°04.6'N, 71°05.5'W), 19 May 1998, D. and W. N. Mathis (4♂; USNM); Cabral (canals E of Cabral; 18°15.2'N, 71°13.4'W), 16 May 1995, W. N. Mathis (2♂, 1♀; USNM); Cabral (2 km E of Cabral; 18°15.2'N, 71°12'W), 16 May 1995, W. N. Mathis (2♂; USNM); Cortico, El Limo (18°06.8'N, 71°13.9'W; 1350 m), 23 Mar 1999, W. N. Mathis (1♂; USNM); Cortico, La Mina (18°06.7'N, 71°13.4'W; 1300 m), 23 Mar 1999, W. N. Mathis (6♂, 1♀; USNM); Ojeda (17°58.2'N, 71°10.6'W), 22 Mar 1999, W. N. Mathis (8♂; USNM). **Pedernales:** Pedernales (14 km E; 17°57.9'N, 71°38.4'W; cenotes), 20 Mar 1999, W. N. Mathis (7♂, 1♀; USNM).

##### Type locality.

Dominican Republic. Pedernales: Pedernales (18°01.8'N, 71°44.7'W).

##### Other specimens examined.

Neotropical. BRAZIL. **Amazonas:** Manaus, Universidade Federal do Amazonas (03°05.9'S, 59°58.2'W; 50 m), 7 May-22 Jun 1982, 2010, D. and W. N. Mathis, J. A. Rafael (1♀; INPA); Manaus, INPA (03°05.9'S, 59°59.1'W; 60 m), 4 May 2010, D. and W. N. Mathis (21♂; INPA, USNM); Reserva Ducke (02°55.8'S, 59°58.5'W; 40 m), 5 May 2010, D. and W. N. Mathis (11♂, 1♀; INPA, USNM).

TRINIDAD and TOBAGO. Tobago. **St. John:** Charlotteville (5 km S; Hermitage River and beach; 11°18.9'N, 60°34.2'W), 22 Apr-11 Jun 1993, 1994, D. and W. N. Mathis (5♂, 6♀; USNM).

West Indies. ANTIGUA. near airport (17°08.2'N, 61°47.6'W), 19 Mar 1989, W. N. Mathis (6♂, 4♀; USNM).

CUBA. **Cienfuegos:** Jardin Botánico (22°7.5'N, 80°19.2'W), 13 Dec 1994, W. N. Mathis (13♂, 5♀; USNM); Topes de Collantes (5 km WNW; 21°56.5'N, 80°2.3'W; 600 m), 11 Dec 1994, W. N. Mathis (1♂; USNM). **Havana:** Ojo de Aqua (23°54.6'N, 82°29.1'W), 8 Dec 1994, W. N. Mathis (2♂; USNM). **Matanzas:** Playa Larga (22°15.9'N, 81°09.9'W), 1 May 1983, W. N. Mathis (5♂, 23♀; USNM).

DOMINICAN REPUBLIC. **Azua:** Puerto Viejo (18°20.9'N, 70°50.4'W), 14 May 1995, W. N. Mathis (7♂, 6♀; USNM); Puerto Viejo (near; 18°24.8'N, 70°44.7'W), 19 May 1998, W. N. Mathis (3♂, 1♀; USNM). **El Seibo:** El Seibo (5 km E; 18°44.73'N, 68°59.2'W; 120 m), 12 May 1995, W. N. Mathis (2♂; USNM); Rincón (near; 18°45.3'N, 68°55.7'W), 12 May 1995, W. N. Mathis (4♂, 1♀; USNM); Pedro Sánchez (18°51.4'N, 69°06.5'W), 26 May 1998, W. N. Mathis (1♂; USNM).. **Independencia:** Baitoa (18°24.2'N, 71°35.7'W), 20 May 1998, D. and W. N. Mathis (2♂; USNM); Los Bolos (18°37.8'N, 71°39.2'W; 1050 m), 25 Mar 1999, W. N. Mathis (1♂, 1♀; USNM). **La Vega:** El Río (9.5 km E; 19°0.9'N, 70°33.5'W; 980 m), 6 May 1995, W. N. Mathis (1♂; USNM); Río Camu (3.5 km NW La Vega; 19°13.7'N, 70°35.2'W; 100 m), 10 May 1995, 1997, D. and W. N. Mathis (8♂, 1♀; USNM); Jarabacoa (1–2 km S; 19°06.9'N, 70°37'W; 520 m), 8–21 May 1995, 1998, D. and W. N. Mathis (10♂, 1♀; USNM); Salto Baiguate (near Jarabacoa; 19°05.5'N, 70°36.9'W; 570 m), 9 May 1995, W. N. Mathis (1♂; USNM). **Monseñor Nouel:** dam nr Rodeo (18°53.1'N, 70°33.5'W), 22 May 1998, D. and W. N. Mathis (1♂; USNM). **Peravia:** Río Ocoa (San José Ocoa; 18°31.7'N, 70°30.4'W), 21 May 1998, D. and W. N. Mathis (2♂; USNM). **Puerto Plata:** Río Camu (14 km E Puerto Plata; 19°41.9'N, 70°37.4'W), 17–23 May 1995, 1997, D. and W. N. Mathis (10♂, 1♀; USNM); Río Pérez (near Imbert; 19°44.1'N, 70°50.2'W), 24 May 1998, D. and W. N. Mathis (1♂; USNM).

GRENADA. **St. Andrew:** Pearls Airport (12°08.7'N, 61°36.6'W), 17 Sep 1996, W. N. Mathis (2♂, 3♀; USNM).

JAMAICA. **Clarendon:** Barnswell Beach (17°45.'N, 77°08.5'W), 13 May 1996, D. and W. N. Mathis, H. B. Williams (1♂, 1♀; USNM); Farquhars Beach (17°50.9'N, 77°22.8'W), 9 May 1996, D. and W. N. Mathis, H. B. Williams (2♂, 4♀; USNM); Grantham (18°09.3'N, 77°23.8'W; 340 m), 16 Apr 2000, W. N. Mathis (4♂, 2♀; USNM); Milk River Bath (17°51'N, 77°22'W; mangroves), 11 Mar 1970, T. Farr, W. W. Wirth (2♂, 11♀; USNM); Rest (3.5 km N; 17°54.1'N, 77°21.1'W), 9 May 1996, D. and W. N. Mathis, H. B. Williams (3♂; USNM). **Manchester:** Mandeville (near; 18°03.5'N, 77°31.9'W), 15–18 Apr 2000, W. N. Mathis (3♂, 2♀; USNM); near Warwick (17°54.1'N, 77°25.5'W), 7 May 1996, D. and W. N. Mathis, H. B. Williams (8♂, 1♀; USNM). **Portland:** Berridale (18°06.5'N, 76°20'W), Rio Grande River, 25 Apr 2000, W. N. Mathis (4♂; USNM); Hollywell (18°05.5'N, 76°43.6'W; 70 m), 27 Apr 2000, W. N. Mathis (1♂; USNM); Reach Falls (Drivers River; 18°01.9'N, 76°18.7'W; 70 m), 25 Apr 2000, W. N. Mathis (1♂; USNM). **St. Andrew:** Mavis Bank (1.7 km E; 18°02.4'N, 77°39.5'W; 575 m), Yallahs River, 21–22 Apr-1 May 2000, W. N. Mathis (5♂, 3♀; USNM). **St. Elizabeth:** Black River (18°01.4'N, 77°51.1'W), 11 May 1996, D. and W. N. Mathis, H. B. Williams (1♂, 1♀; USNM); Brae River (18°05.2'N, 77°39.3'W), 10 May 1996, D. and W. N. Mathis, H. B. Williams (1♂; USNM); Elim (18°07.1'N, 77°40.5'W), 14 Apr 2000, W. N. Mathis (11♂, 2♀; USNM); near Port Kaiser (17°52.3'N, 77°34.9'W), 8 May 1996, D. and W. N. Mathis, H. B. Williams (10♂, 3♀; USNM); Wally Wash Great Pond (17°57.9'N, 77°48.6'W), 19 Apr 2000, W. N. Mathis (5♂; USNM). **St. Thomas:** Yallahs River (mouth; 17°53'N, 76°35.6'W), 14 May 1996, D. and W. N. Mathis, H. B. Williams (9♂, 2♀; USNM).

PUERTO RICO. Adjuntas (18°09.8'N, 66°43.2'W), 22 Sep 1995, D. and W. N. Mathis (1♂, 1♀; USNM); Jayuya (2 km E; Río Saliente; 18°12.8'N, 66°33.9'W), 22 Sep 1995, D. and W. N. Mathis (1♂, 3♀; USNM); Maricao (4 km WNW; 18°10.7'N, 66°59.6'W), 21 Sep 1995, D. and W. N. Mathis (9♂, 5♀; USNM); Punta Jacinto (near Guanica; 17°57'N, 66°52.6'W), 20 Sep 1995, D. and W. N. Mathis (3♂; USNM); Río Hoconuco (18°7.6'N, 67°2.6'W), 20 Sep 1995, D. and W. N. Mathis (3♂, 3♀; USNM).

##### Distribution

**Figure 119.**
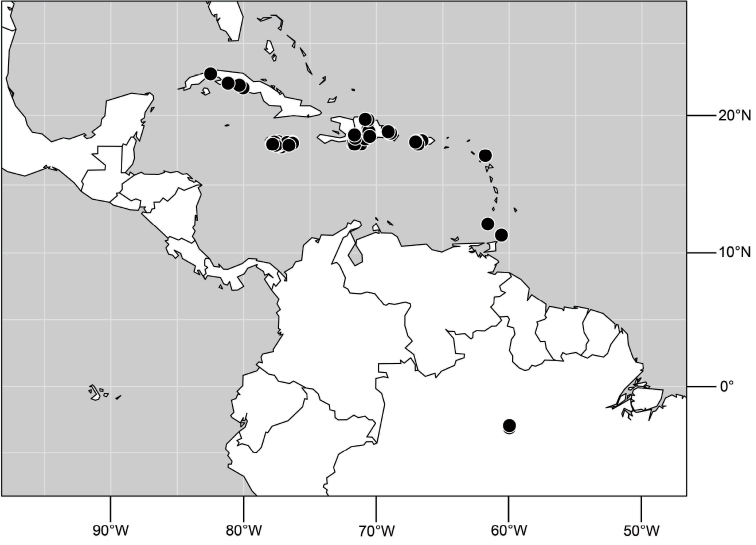
Distribution of *Hydrochasma peniculum* sp. n.

(Fig. 119). Neotropical: Brazil (Amazonas), Trinidad and Tobago, West Indies (Antigua, Cuba, Dominican Republic, Grenada, Jamaica, Puerto Rico).

##### Etymology.

The species epithet, *peniculum*, is of Latin derivation and means tail or penis, referring to the shape of the epandrial extensions.

##### Remarks.

This species is similar to *Hydrochasma incisum* in having the dorsal half of the epandrium somewhat rectangular and in lacking a dorsal connecting band above the cerci, and the ventral portion, the ventral, epandrial process, is distinctly narrower in both species. This species is distinguished from *Hydrochasma incisum*, however, in having a parallel-sided ventral epandrial process that is not as long but is more robustly developed than in *Hydrochasma incisum*, and the apical portion is more expanded.

#### 22. Hydrochasma simplicum sp. n.

http://zoobank.org/3DE5780E-6559-4238-AD2D-5A24270D982C

http://species-id.net/wiki/Hydrochasma_simplicum

Figs 120–123 , 131

##### Diagnosis.

**Figures 120–123.**
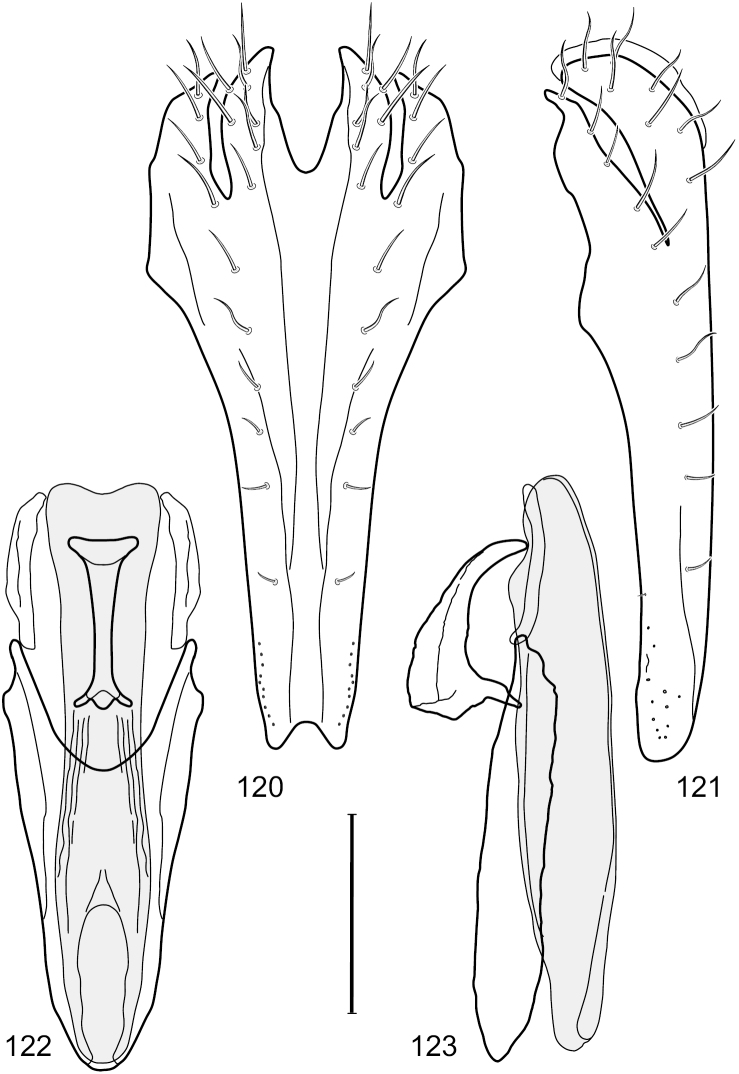
*Hydrochasma simplicum* sp. n. (Costa Rica. Cartago: Parque Nacional Barbilla, Río Dantas) **120** epandrium and cerci, posterior view **121** same, lateral view **122** internal structures of male terminalia (aedeagus [shaded], phallapodeme, gonite, hypandrium), ventral view **123** same, lateral view. Scale bar = 0.1 mm.

This species is distinguished from congeners by the following combination of characters: Moderately small shore flies, body length 2.15 mm. *Head*: Antenna mostly dark gray; parafacial silvery white, concolorous with facial coloration; gena-to-eye ratio 0.17. *Thorax*: Wing with costal vein ratio 0.72–0.78; M vein ratio 0.52–0.54. Forefemur lacking a distinctive, comb-like row of stout setulae along anteroventral surface; tibiae mostly gray. *Abdomen*: Tergites 3–4 with wedge-shaped silvery-gray areas. Male terminalia (Figs 120–123): Combined structures generally elongate, in posterior view height more than twice width, generally sparsely setulose dorsally, setulae becoming sparse or lacking ventrally; epandrium lacking dorsal arch above cerci, in posterior view (Fig. 120) with basal 1/2-2/3 quadrate, thereafter ventrally narrowed as a taper to apex, apex with medioapical, small, U-shaped notch, in lateral view (Fig. 121) very elongate, moderately narrow with apical 3/4 straight, nearly parallel sided, basal 1/3 wider, somewhat rectangular; aedeagus in lateral view (Fig. 123) tubular, very elongate, narrow, more than 6 times longer than wide, mostly parallel sided, apical portion tapered to broad point, ventral view (Fig. 122) narrow, elongate, apical 1/8 tapered to rounded apex; phallapodeme in lateral view (Fig. 123) irregularly crescent shaped, more elongate and narrow toward base of aedeagus, keel elongate along most of phallapodeme but not extended more than width of phallapodeme, process toward hypandrium short, pointed, in ventral view (Fig. 122) somewhat I-shaped with unequal crossbars; gonite in lateral view (Fig. 123) as a very shallowly curved to sinuous, rod-like process, about equal in length to phallapodeme, in ventral view (Fig. 122) very shallowly curved to straight, slightly tapered at both apices; hypandrium in lateral view (Fig. 123) narrowly developed, parallel sided, length 3/4 that of aedeagus, in ventral view (Fig. 122) length about twice basal width, tapered from posterior to rounded, more narrowed anterior margin, deeply and narrowly notched anteromedially, posterior margin moderately deeply U-shaped, lateral margins slightly flared laterally.

##### Type material.

The holotype male of *Hydrochasma simplicum* is labeled “COSTA RICA. Prov. Limón. P[arque]. N[acional]. Barbilla, Sector Casas Negra, Orilla Río Dantas, 300m, 15DEC 2002, E. Rojas, Red de Golpe. L N 219900 598400 #704204/INB0003814812 INBIOCRI COSTA RICA [plastic bar code label]/HOLOTYPE ♂ *Hydrochasma simplicum* Mathis & Zatwarnicki, USNM [red].” The holotype is double mounted (minuten in a block of plastic), is in good condition (some setae of the head are misoriented or missing; abdomen removed and dissected), and is deposited in INBio. Paratypes are as follows: COSTA RICA. **Cartago:** Parque Nacional Barbilla, Sendero Principal, Río Dantas (09°58.5'N, 83°26.5'W; 370 m), 8 Jul 2002, E. Rojas (1♂; INBio); Río Pacuare, Sendero el Felino (09°43.6'N, 83°30.8'W; 400 m), 7 Sep 2002, E. León, E. Rojas (1♂; USNM).

##### Type locality.

Costa Rica. Limón: Parque Nacional Barbilla, Sector Casas Negras, (10°01.2'N, 83°26.2'W; 300 m).

##### Distribution

(Fig. 131). Neotropical: Costa Rica (Cartago, Limón).

##### Etymology.

The species epithet, *simplicum*, is of Latin derivation and means simple, unmixed, or single, referring to the simplistic structures of the male terminalia.

##### Remarks.

Among congeners, especially those of the *incisum* group, this species is distinguished by its simplicity, hence its species name. The epandrium, including the ventral epandrial processes, are simple, mostly straight and unadorned, as is the aedeagus, gonite, and hypandrium (Figs 120–123).

#### 23. Hydrochasma urnulum sp. n.

http://zoobank.org/3D748A83-0291-4227-8657-52D6C9748F40

http://species-id.net/wiki/Hydrochasma_urnulum

Figs 124 – 131 , 143

##### Diagnosis.

This species is distinguished from other congeners by the following combination of characters: Small shore flies, body length 1.20–1.65 mm. *Head*: Antenna mostly dark gray; parafacial silvery white, concolorous with facial coloration; gena-to-eye ratio 0.14–0.16. *Thorax*: Wing with costal vein ratio 0.69–0.71; M vein ratio 0.53–0.55. Forecoxa mostly silvery gray to gray, with some yellowish coloration at ventral apex. Hind tibia with a small apicoventral seta. *Abdomen*: Tergites 3–4 with moderately deep, gray wedges along lateral margin of darkened coloration (Fig. 143). Male terminalia (Figs 127–130): Epandrium generally elongate, setulae moderately sparse though some elongate on dorsal portion, in posterior view (Fig. 127) with dorsal arch attenuate, not connected, dorsal half more or less rectangular, thereafter ventrally narrowed with ventral epandrial extensions narrowly developed, somewhat parallel sided to rectangular-shaped, slightly broader apex, in lateral view (Fig. 128) moderately elongate, anterior margin conspicuously sinuous, apex tapered, somewhat narrowly rounded; cerci short, length in lateral view (Fig. 127) slightly more than twice width, hemispherical; aedeagus in lateral view (Fig. 130) relatively simple, narrowly tubular, length 5× greatest width, shallowly curved subapically, in ventral view (Fig. 129) also tubular, narrow, elongate; phallapodeme in lateral view (Fig. 130) linear, elongate, very shallowly curved, extended keel narrow and short, as a slight bump, in ventral view (Fig. 83) T-shaped with crossbar at hypandrial end and gradually expanding toward aedeagal end, the end truncate; gonite in lateral view (Fig. 130) narrowly elongate, bar-like, more curved than phallapodeme, in ventral view (Fig. 129) narrowly bar-like, elongate, nearly straight; hypandrium in lateral view (Fig. 130) elongate, robustly narrow, length about 2/3 that of aedeagus, shallowly angulate, in ventral view (Fig. 129) elongate, U-shaped with anterior margin moderately narrowly rounded, lateral margins nearly parallel sided, posterior margin deeply incised, each arm tapered to narrow posterior process.

**Figures 124–126.**
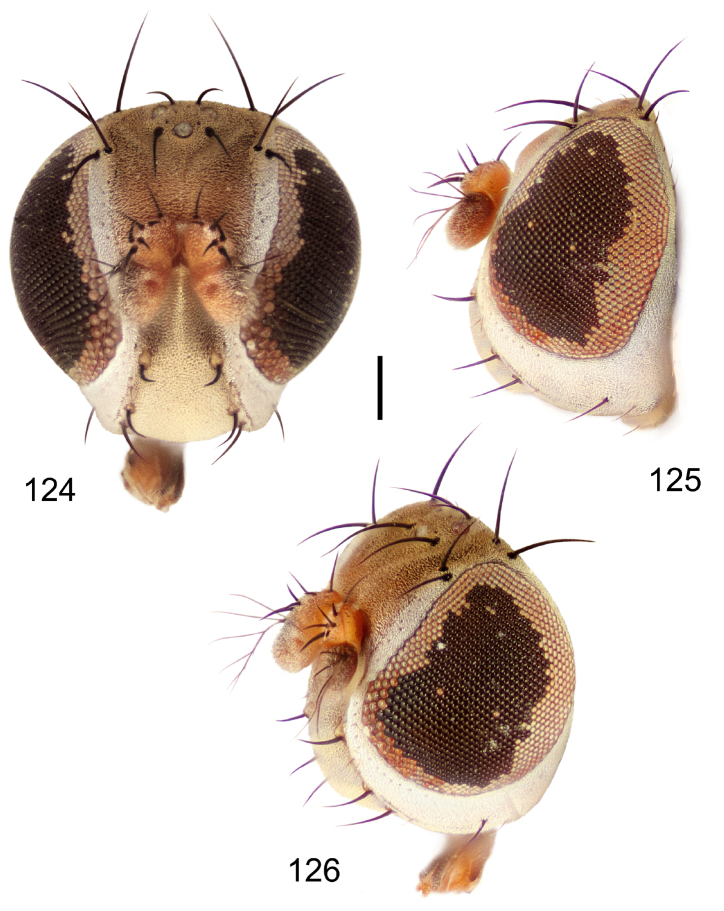
*Hydrochasma urnulum* sp. n. (Dominican Republic. LaVega: Jarabacoa) **124** head, anterior view **125** same, lateral view **126** same, oblique view. Scale bar = 0.1 mm.

**Figures 127–130.**
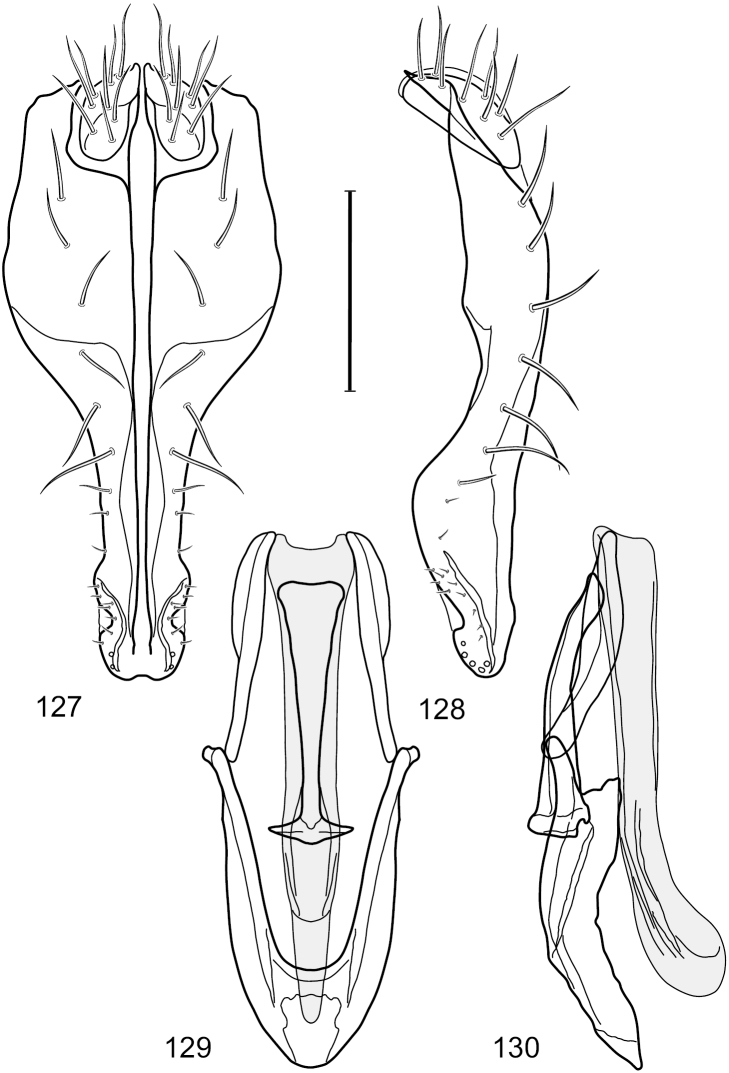
*Hydrochasma urnulum* sp. n. (Dominican Republic. Barahona: Baoruco - beach and river) **127** epandrium and cerci, posterior view **128** same, lateral view **129** internal structures of male terminalia (aedeagus [shaded], phallapodeme, gonite, hypandrium), ventral view **130** same, lateral view. Scale bar = 0.1 mm.

##### Type material.

The holotype male of *Hydrochasma urnulum* is labeled “DOMINICAN RP. Puerto Plata (14 km E), R. Camu[,] 19°41.9'N, 70°37.5'W[,] 23May1998, WNMathis/USNM ENT 00088078 [plastic bar code label]/HOLOTYPE ♂ *Hydrochasma urnulum* Mathis & Zatwarnicki, USNM [red].” The holotype is double mounted (minuten in a block of plastic), is in excellent condition, and is deposited in the USNM. Fifteen paratypes (11♂, 4♀; USNM) bear the same label data as the holotype.

##### Type locality.

Dominican Republic. Puerto Plata: Río Camu (14 km E Puerto Plata; 19°41.9'N, 70°37.5'W).

##### Other specimens examined.

Neotropical. West Indies. COSTA RICA. **Guanacaste:** Bagaces Fortuna Z. P. Miravalles (10°43.1'N, 84°51.3'W; Sendero Cabro Muco; 980 m), 8–31 Jul 2002, J. D. Gutierrez (2♂, 4♀; INBio).

CUBA. **Cienfuegos:** Jardin Botánico (22°7.5'N, 80°19.2'W), 13 Dec 1994, W. N. Mathis (2♂; USNM). **Sancti Spiritus:** Topes de Collantes (21°54.4'N, 80°01.4'W; 670 m), 9–11 Dec 1994, W. N. Mathis (1♂, 2♀; USNM).

DOMINICAN REPUBLIC. **La Vega:** El Río (9.5 km E; 19°0.9'N, 70°33.5'W; 980 m), 6 May 1995, W. N. Mathis (1♂; USNM); Jarabacoa (1–2 km S; 19°06.9'N, 70°37'W; 520 m), 8–21 May 1995, W. N. Mathis (2♂, 5♀; USNM). **Puerto Plata:** Río Pérez (near Imbert; 19°44.1'N, 70°50.2'W), 24 May 1998, D. and W. N. Mathis (3♂; USNM).

JAMAICA. **St. Thomas:** Bath River, Bath (17°56.8'N, 76°21.6'W), 16 May 1996, D. and W. N. Mathis, H. B. Williams (3♂, 1♀; USNM).

PUERTO RICO. Adjuntas (18°09.8'N, 66°43.2'W), 22 Sep 1995, D. and W. N. Mathis (1♂; USNM); Maricao (18°11.1'N, 66°58.9'W), 21 Sep 1995, D. and W. N. Mathis (1♂; USNM).

##### Distribution

**Figure 131.**
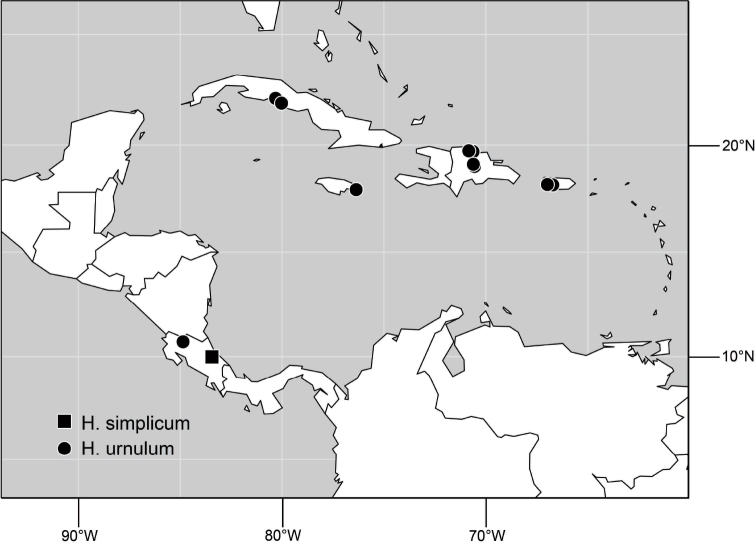
Distribution of *Hydrochasma simplicum* sp. n. and *Hydrochasma urnulum* sp. n.

(Fig. 131). Neotropical: Costa Rica (Guanacaste), West Indies (Cuba, Dominican Republic, Jamaica, Puerto Rico).

##### Etymology.

The species epithet, *urnulum*, is of Latin derivation and means urn, referring to the urn-shaped hypandrium in ventral view.

##### Remarks.

Like *Hydrochasma glochium*, *Hydrochasma urnulum* has an expanded apical portion of the extended epandrial process but only slightly so (best seen in posterior view). The hypandrium of *Hydrochasma urnulum* is deeply and thinly V-shaped in ventral view with a distinct, U-shaped, posteromedial emargination (Fig. 129). In males of *Hydrochasma glochium*, the expanded apical portion of the epandrium is more pronounced (Fig. 86), and the hypandrium is V-shaped (Fig. 88) with each arm robustly developed.

### The *leucoproctum* Group

**Species included.** *Hydrochasma andeum* sp. n., *Hydrochasma annae* sp. n., *Hydrochasma aquia* Mathis & Zatwarnicki, *Hydrochasma avanae* Mathis & Zatwarnicki, *Hydrochasma capsum* sp. n., *Hydrochasma edmistoni* sp. n., *Hydrochasma garvinorum* Mathis & Zatwarnicki, *Hydrochasma leucoproctum* (Loew), *Hydrochasma robustum* sp. n., *Hydrochasma sagittarium* sp. n., *Hydrochasma lineatum* sp. n.

**Diagnosis.** This species group is distinguished from others within *Hydrochasma* by the following combination of characters: Head: Oral opening small, not gaping. Thorax: Hindtibia lacking a prominent, spur-like, ventral, subapical seta. Abdomen: Tergites with a sharply demarcated, straight lateral line, lacking lateral wedges. Male terminalia: Ventral epandrial extensions usually somewhat narrow and parallel-sided, at least at base; ventral margin of epandrium variable, if bifurcate, gap narrow and relatively shallow.

#### Key to species of the *leucoproctum* group

**Table:** 

1	Forecoxa mostly to entirely yellowish; face mostly creamy yellow distinctly contrasting in coloration with white to silvery white parafacial, lateral margin of face, immediately adjacent to parafacial dark brown (except *Hydrochasma sagittarium* which has a non-contrasting facial color)	2
–	Forecoxa mostly gray to blackish gray, at most only slightly yellowish; facial coloration at most only slightly distinct from parafacial	4
2	Parafacial and face essentially the same color, not distinctly contrasted; ventral extensions of epandrium with apical 1/3 arrow-like in posterior view (Fig. 191)	*Hydrochasma sagittarium* sp. n.
–	Parafacial white, contrasted with more tan colored face; ventral extensions of epandrium with apical 1/3 broad, apex almost truncate (Figs 157, 186)	3
3	Tergite 5 of male with a large, oval black spot toward posterior margin	*Hydrochasma capsum* sp. n.
–	Tergite 5 of male with at most posterior margin blackish, lacking a large, oval black spot	*Hydrochasma robustum* sp. n.
4	Ventral extensions of epandrium with subapical, shallowly V-shaped structure that bears setulae; apex of ventral epandrial extensions in posterior view irregularly shaped (Fig. 162)	*Hydrochasma edmistoni* sp. n.
–	Ventral extensions of epandrium lacking a subapical, shallowly V-shaped structure that bears setulae; apex of ventral epandrial extensions in posterior view evenly tapered to apex	5
5	Ventral extensions of epandrium very elongate, nearly 3× longer than basal width, parallel sided, apical quarter tapered to point (Fig. 180); hypandrium in ventral view (Fig. 182) 3× longer than wide	*Hydrochasma lineatum* sp. n.
–	Ventral extensions of epandrium moderately elongate, at most length twice basal width	6
6	Ventral extensions of epandrium shallowly sinuous, apical quarter parallel sided, apex rounded (Fig. 132); hypandrium in ventral view (Fig. 134) twice as long as wide	*Hydrochasma andeum* sp. n.
–	Ventral extensions of epandrium straight or tapered, not shallowly sinuous	7
7	Fifth tergite slate black, similar to coloration of medial area on tergites 1–4	*Hydrochasma garvinorum* Mathis & Zatwarnicki
–	Fifth tergite silvery gray, similar to coloration along lateral margins of preceding tergites	8
8	Fifth tergite with posterior margin dark, slate black, similar to coloration of medial area on tergites 1–4; medial coloration on tergites 1–4 wide, occupying most of dorsum, dark, grayish to slate black (Fig. 185)	*Hydrochasma leucoproctum* (Loew)
–	Fifth tergite, including posterior margin, uniformly silvery gray; medial coloration on tergites 1–4 narrow, sometimes only a stripe, slightly darker than color of lateral margins	9
9	Hindtibia bearing an apical black seta ventroapically	*Hydrochasma avanae* Mathis & Zatwarnicki
–	Hindtibia with only pale, yellowish setae ventroapically	10
10	Aedeagus with apical, membranous portion invested with numerous cuticular spicules (Figs 146–147)	*Hydrochasma aquia* Mathis & Zatwarnicki
–	Aedeagus with apical, membranous portion plain, lacking cuticular spicules (Figs 139–140)	*Hydrochasma annae* sp. n.

#### 24. Hydrochasma andeum sp. n.

http://zoobank.org/7C965723-2394-4924-BE8B-EA86383A2033

http://species-id.net/wiki/Hydrochasma_andeum

Figs 132 –136

##### Diagnosis.

**Figures 132–135.**
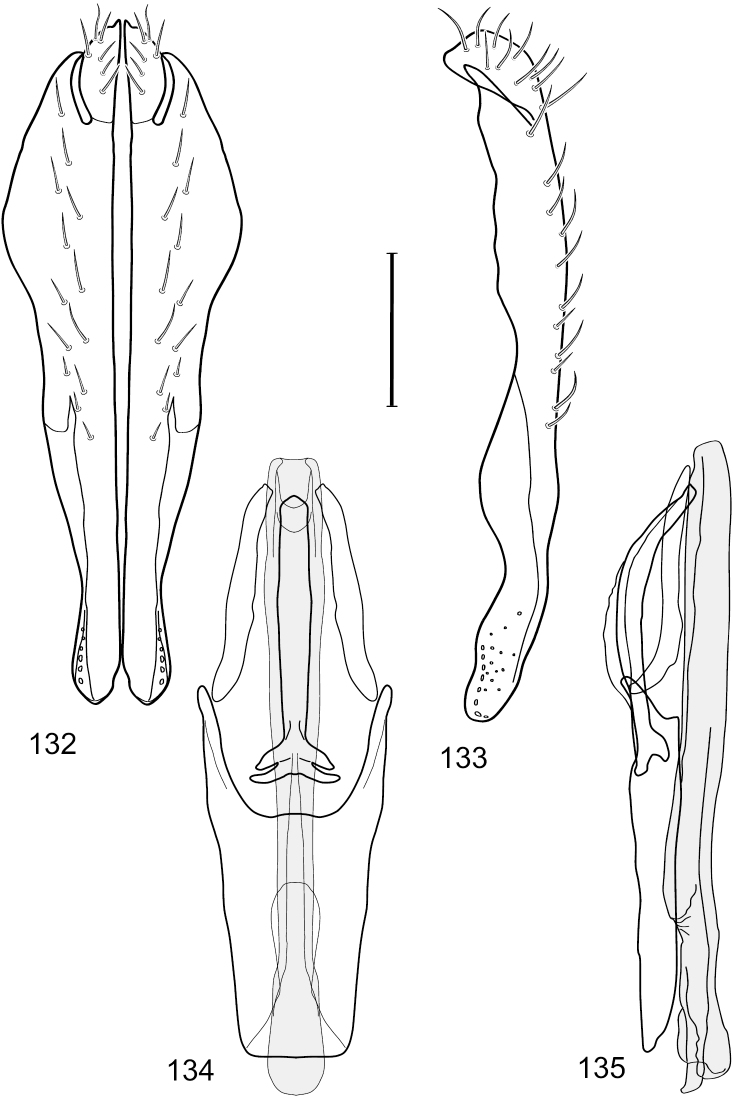
*Hydrochasma andeum* sp. n. (Ecuador. Manabi: Pietro Carbo) **132** epandrium and cerci, posterior view **133** same, lateral view **134** internal structures of male terminalia (aedeagus [shaded], phallapodeme, gonite, hypandrium), ventral view **135** same, lateral view. Scale bar = 0.1 mm.

This species is distinguished from congeners by the following combination of characters: Small shore flies, body length 1.55–1.90 mm. *Head*: Subglobose, very broad ventrally, oral opening comparatively large; at least pedicel black, otherwise antennal coloration variable; parafacial silvery white, concolorous with facial coloration; parafacial silvery white, concolorous with face; gena-to-eye ratio 0.13–0.16. *Thorax*: Mesonotum tan to golden brown; pleural area gray. Wing with costal vein ratio 0.72–0.75; M vein ratio 0.48–0.49. Forecoxa mostly yellowish, with some silvery gray areas basally and laterally; forefemur lacking a distinctive, comb-like row of stout setulae along anteroventral surface; tibiae mostly gray; hindtibia lacking a long, spur-like seta ventroapically. *Abdomen*: Tergites 2–4 with lateral margin of distinctly darkened area essentially straight, sharply contrasted with gray lateral and ventral surfaces. Male terminalia (Figs 132–135): Epandrium generally elongate, length nearly 3× width, elongate, in posterior view (Fig. 132) elongate diamond shaped, ventral portion double length of dorsal portion, both tapered; cerci fused ventrally with epandrium, height about twice width in lateral view, in lateral view (Fig. 133), elongate, narrow, anterior margin sinuous, posterior very shallowly sinuous; aedeagus in lateral view (Fig. 135) elongate, narrow, tubular, almost parallel sided, in ventral view (Fig. 134) also tubular, elongate narrow; phallapodeme in lateral view (Fig. 135) elongate, narrow, curved, slightly wider medially, epandrial end shallow bifurcate, in ventral view (Fig. 134) elongate, T-shaped with double crossbars, these pointed; gonite in lateral view (Fig. 135) elongate, narrow, curved toward hypandrial end, in ventral view (Fig. 134) elongate, narrow, bar-like; hypandrium in lateral view (Fig. 135) elongate, narrow, tapered at both ends, in ventral view (Fig. 134) robustly U-shaped with greatly enlarged, thick, anterior portion of U, anterior margin truncate, posterior margin deeply emarginate, emargination U-shaped.

##### Type material.

The holotype male of *Hydrochasma andeum* is labeled “ECUADOR: Guayas Pr. Boliche (14.5 km S.)[,] 14 Jan 1978[,] Wayne N. Mathis/USNM ENT 00117965 [plastic bar code label]/HOLOTYPE ♂ *Hydrochasma andeum* Mathis & Zatwarnicki, USNM [red].” The holotype is double mounted (minuten in a block of plastic), is in excellent condition, and is deposited in the USNM. Seven paratypes (1♂, 6♀; USNM) bear the same label data as the holotype. Other paratypes are as follows: ECUADOR. **Guayas:** Pedro Carbo (01°48.7'S, 80°13.9'W), 11 Jan 1978, W. N. Mathis (5♂ in glycerin; 1♂, 3♀; USNM). **Manabí:** Bahia de Caraquez (0°36.5'S, 80°25.8'W), 10 Jan 1978, W. N. Mathis (3♂, 2♀; USNM).

##### Type locality.

Ecuador. Guayas: Boliche (02°07.7'S, 79°35.5'W).

##### Other specimens examined.

Neotropical. ECUADOR. **Azuay:** Río Rircay (03°20'S, 79°19'W), 31 Oct 1954, R. Levi-Castillo (1♂, 1♀; USNM). **El Oro:** El Guabo (03°13'S, 79°47.7'W), Dec 1955, R. Levi-Castillo (1♀; USNM); El Pasaje (03°19.6'S, 79°48.3'W), 1 Nov 1954, R. Levi-Castillo (1♂; USNM); Machala (03°15.6'S, 79°57.8'W), Dec 1955, R. Levi-Castillo (2♂, 3♀; USNM), Puerto Bolivar (03°16'S, 80°W), Dec 1955, R. Levi-Castillo (2♂, 3♀; USNM). **Guayas:** Balao (02°55'S, 79°49'W), Dec 1955, R. Levi-Castillo (1♂, 2♀; USNM); Barranco Chico (02°03.5'S, 79°48.2'W), 28 Aug 1955, R. Levi-Castillo (2♂, 3♀; USNM); Chobo (02°09'S, 79°38'W), Jun 1955, R. Levi-Castillo (8♂, 4♀; USNM); Cone (02°10.1'S, 79°38'W), Jun 1955, R. Levi-Castillo (3♂, 3♀; USNM); Gala (03°0.7'S, 79°44.3'W), Dec 1955, R. Levi-Castillo (1♂, 1♀; USNM); Naranjal (02°40'S, 79°37'W), Dec 1955, R. Levi-Castillo (2♂, 3♀; USNM); Quatro Hermanitos Experimental Farm (4 km WNW Guayaquil; 02°10.4'S, 79°56.2'W), R. Levi-Castillo (1♀; USNM); Taura (02°18.6'S, 79°43.9'W), Dec 1955, R. Levi-Castillo (1♂; USNM); Tenguel (02°59.9'S, 79°47.3'W), Dec 1955, R. Levi-Castillo (15♂, 8♀; USNM). **Loja:** Catamayo (03°59'S, 79°21'W), Dec 1955, R. Levi-Castillo (21♂, 12♀; USNM); Vilcabamba (04°15.7'S, 79°13.3'W), Dec 1955, R. Levi-Castillo (1♂; USNM). **Manabí:** Bandurria (0°45.7'S, 80°30.7'W), Aug 1955, R. Levi-Castillo (1♂, 2♀; USNM); Estero Balsa (02°02'S, 80°43'W), 9 Sep 1955, R. Levi-Castillo (2♂; USNM); Guale (01°37.9'S, 80°14.3'W), 23 Apr 1955, R. Levi-Castillo (3♂, 2♀; USNM); La Palma (0°45.7'S, 80°30.7'W), Aug 1955, R. Levi-Castillo (2♂, 1♀; USNM). **Pichicha:** Guare, Los Ríos (01°41'S, 79°42'W), Aug 1955, R. Levi-Castillo (5♂, 4♀; USNM).

##### Distribution

**Figure 136.**
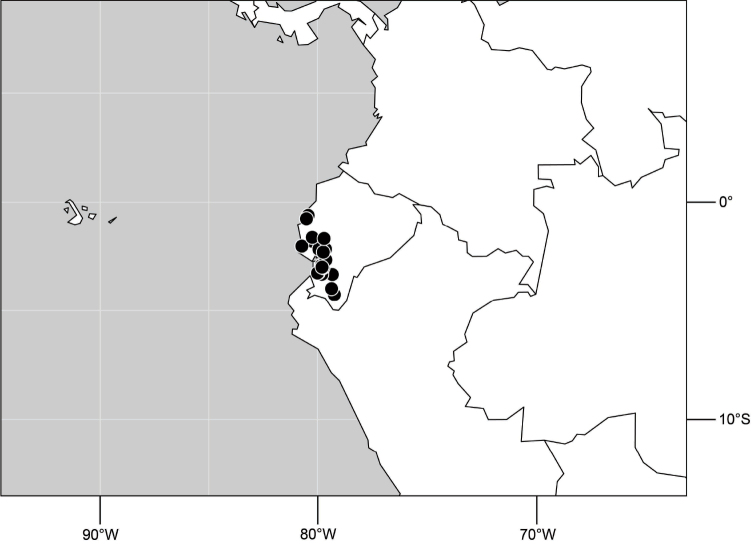
Distribution of *Hydrochasma andeum* sp. n.

(Fig. 136). Neotropical: Ecuador (Azuay, El Oro, Guayas, Loja, Manabí, Pichicha).

##### Etymology.

The species epithet, *andeum*, is a Latinized word that refers to the general area in South America where this species was collected. The word is treated as a noun in apposition.

##### Remarks.

This species is similar and evidently closely related to *Hydrochasma annae*, especially the similar shapes of their respective epandriums. It is distinguished from *Hydrochasma annae* by having more sinuous margins of the epandrium in posterior and lateral views (Figs 132–133) and in the more robustly developed hypandrium that is thickly developed on the anterior two-thirds. This species is known thus far only from Ecuador, and *Hydrochasma annae* is from western United States.

#### 25. Hydrochasma annae sp. n.

http://zoobank.org/ED9D3074-6AEF-428D-AEEB-A9A6DC9211D1

http://species-id.net/wiki/Hydrochasma_annae

Figs 137 –141

##### Diagnosis.

**Figures 137–140.**
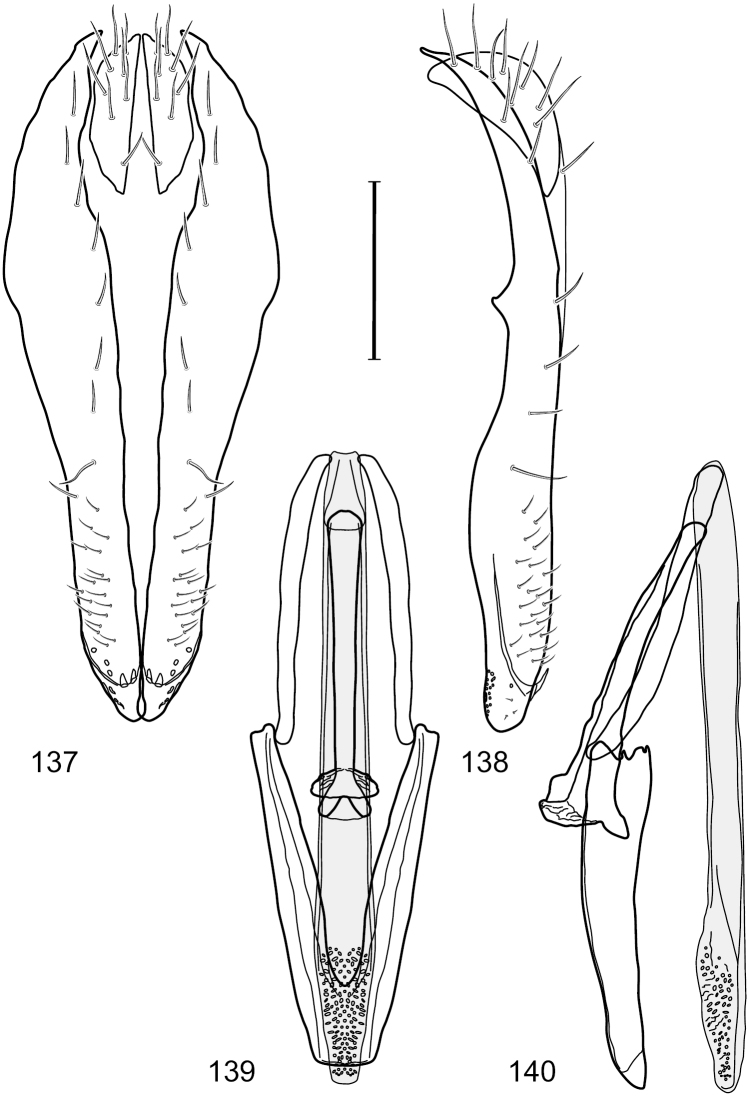
*Hydrochasma annae* sp. n. (USA. Utah. Grand: Green River) **137** epandrium and cerci, posterior view **138** same, lateral view **139** internal structures of male terminalia (aedeagus [shaded], phallapodeme, gonite, hypandrium), ventral view **140** same, lateral view. Scale bar = 0.1 mm.

This species is distinguished from congeners by the following combination of characters: Small to moderately small shore flies, body length 1.75–2.20 mm. *Head*: Antenna mostly dark gray; parafacial silvery white, concolorous with facial coloration. Gena moderately high, height usually slightly higher than length of basal flagellomere; gena-to-eye ratio 0.24–0.26. *Thorax*: Wing hyaline with costal vein ratio 0.75–0.76; M vein ratio 0.51–0.53. Forecoxa gray to silvery gray; hindtibia with only pale, yellowish setae ventroapically. *Abdomen*: Tergites 2–4 with brownish gray, relatively narrow, medial stripe, lateral margin of darkened areas lacking wedge-like markings; tergite 5 of male gray. Male terminalia (Figs 137–140): Combined structures generally elongate, in posterior view (Fig. 137) height about 2.5× width, narrowly diamond shaped; epandrium with dorsal arch above cerci attenuate, not connected, becoming slightly wider to dorsal 1/3, thereafter ventrally tapered to shallowly bilobed ventral apex, generally bearing setulae, in lateral view (Fig. 138) mostly parallel sided with anterior shallowly sinuous, apical 1/3 tapered to moderately rounded apex; cerci moderately long, height slightly more than twice width (Figs 137); aedeagus in lateral view (Fig. 140) very elongate, narrow, with length of sclerotized, basal portion about 10× or more width, apical, membranous portion not greatly expanded or ballooned apically, at most slightly swollen, in ventral view (Fig. 139) also narrow and very elongate, almost parallel sided; phallapodeme in lateral view (Fig. 140) narrow, elongate, unevenly clavate toward hypandrium, keel slightly developed, apical width, including keel, slightly more than greatest aedeagal width, in ventral view (Fig. 139) with hypandrial end narrowly as a double T (2 bars), thereafter toward base of aedeagus bar-like, parallel sided; gonite in lateral view (Fig. 140) narrow, elongate, very shallowly sinuous, in ventral view (Fig. 139) narrow, bar-like with apices slightly curved in opposite directions; hypandrium in lateral view (Fig. 140) elongate, moderately shallow, width slightly more than 1/4 length, very shallowly curved, in ventral view (Fig. 139) generally V-shaped with posterior margin very deeply V-shaped, anterior margin moderately narrow, truncate.

##### Type material.

The holotype male of *Hydrochasma annae* is labeled “**USA** [UNITED STATES]. **UT.** Grand: Green River (15.3 km N; 39°7'N, 110°6.6'W; 1255 m), 30 Jul 2007, D.&W.N.Mathis/USNM ENT 00117970 [plastic bar code label]/HOLOTYPE ♂ *Hydrochasma annae* Mathis & Zatwarnicki, USNM [red].” The holotype is double mounted (minuten in a block of plastic), is in excellent condition, and is deposited in the USNM. Seven paratypes (3♂, 4♀; USNM) bear the same label data as the holotype. Other paratypes are as follows: UTAH. **Emery:** Green River (3 km N; 38°59'N, 110°09'W), 30 Jul-5 Aug 1992, 2007, D. and W. N. Mathis (1♂, 3♀; USNM); Green River (Green River; 38°59.6'N, 110°08.5'W; 1240 m), 19–31 Jul 1988, 2007, D. and W. N. Mathis (1♂, 2♀; USNM). **Grand:** Thompson Spring (8.9 km N Thompson Springs; 39°02.3'N, 109°43.4'W; 1740 m), 1–16 Aug 2007, 2008, D. and W. N. Mathis (3♂, 4♀; USNM).

##### Type locality.

United States. Utah. Grand: Swasey Beach (15.3 km N Green River; 39°07'N, 110°06.6'W; Green River; 1255 m).

##### Other specimens examined.

Nearctic. UNITED STATES. CALIFORNIA. **Imperial:** Yuma (32°44'N, 114°37'W), 3–5 May 1918, J. C. Bradley (1♂, 2♀; CU).

COLORADO. **Montezuma:** McPhee Reservoir (37°29.7'N, 108°33'W; 2115 m), 3 Aug 2007, D. and W. N. Mathis (1♀; USNM).

MISSOURI. **Boone:** Columbia (38°57.1'N, 92°20'W), 26 May-8 Jun 1906, C. R. Crosby (1♂, 6♀; ANSP). **Jackson:** Atherton (39°11.2'N, 94°18.3'W), (1♀; ANSP).

NEW MEXICO. **Catron:** Gila River (33°13.6'N, 106°15.1'W; 1750 m), 15 Aug 2007, D. and W. N. Mathis (1♂; USNM). **Grant:** Mimbres River (NM Highway 61 and Royal John Mine Road; 32°43.8'N, 107°52'W; 1665 m), 1–22 Aug 2008, 2009, D. and W. N. Mathis, T. Zatwarnicki (33♂, 13♀; USNM). **Lincoln:** Capitan (3.2 km E; Salado Creek; 33°32.6'N, 105°32.3'W; 1890 m), 10 Aug 2007, D. and W. N. Mathis (1♂; USNM). **Sandoval:** La Cueva (Junction of Highways 126 & 4; 35°52'N, 106°38.4'W; 2342 m), 15 Jun 2011, D. and W. N. Mathis (2♂; USNM). **Valencia:** Río Puerco (34°47.8'N, 106°59.5'W; 1575 m), 9 Aug 2007, D. and W. N. Mathis (1♂; USNM).

SOUTH DAKOTA. **Bon Homme:** Springfield (42°51.2'N, 97°53.8'W), 26 Jun 1924 (1♂, 1♀; ANSP).

TEXAS. **Galveston:** Galveston Island (25°10'N, 95°05'W), 14 May 1993, D. and W. N. Mathis (2♀; USNM). **St. Augustine:** Rayburn Park (31°04'N, 94°05'W), 15 May 1993, D. and W. N. Mathis (3♂, 2♀; USNM).

WASHINGTON. **Franklin:** Columbia River N of Pasco (46°21.5'N, 119°15.5'W), 29 Jul 1998, W. N. Mathis (7♂, 1♀; USNM); Ringold (4.8 km N; 46°30.9'N, 119°15.3'W), 2 Jul 1988, D. and W. N. Mathis (1♀; USNM).

##### Distribution

**Figure 141.**
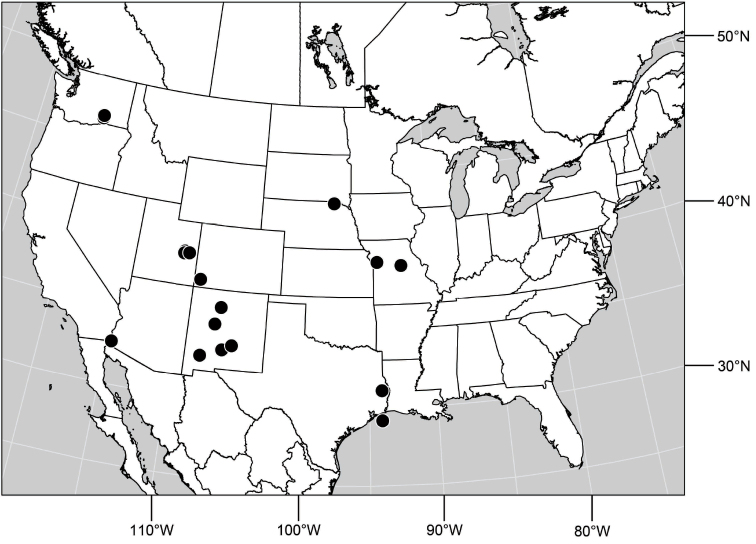
Distribution of *Hydrochasma annae* sp. n.

(Fig. 141). Nearctic: United States (California, Colorado, Missouri, New Mexico, South Dakota, Texas, Utah, Washington).

**Figures 142–143.**
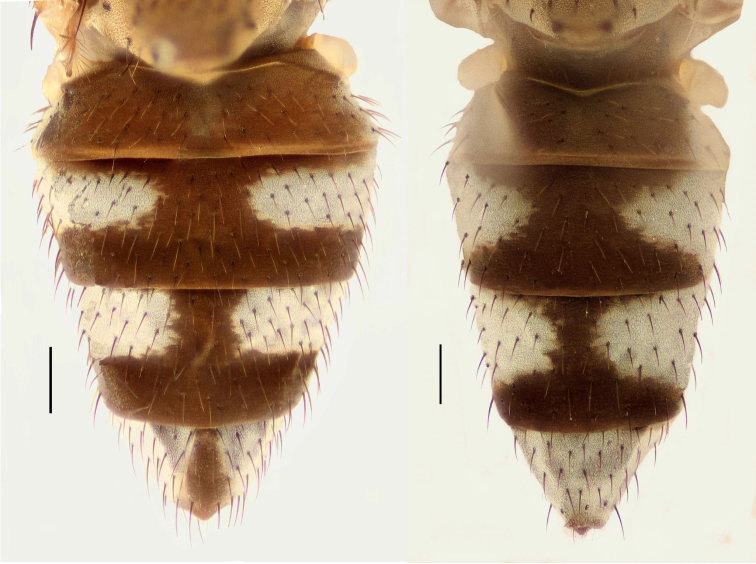
Abdomens of males, dorsal view. **142** *Hydrochasma dolabrutum* sp. n. (Jamaica. Clarendon: Barnswell Beach) **143** *Hydrochasma urnulum* sp. n. (Dominican Republic. LaVega: Jarabacoa). Scale bar = 0.1 mm.

##### Etymology.

The species epithet, *annae*, is a feminine genitive patronym to recognize Ms. AnnaLee Thayn who inspired us while collecting in the Southwest.

##### Remarks.

Externally, this species is very similar and evidently closely related to *Hydrochasma andeum* and *Hydrochasma avanae* but can be distinguished from either of these two species by having a hindtibia with only yellowish setulae ventrally near the apex, less sinuous margins of the epandrium, and by having a more delicately developed hypandrium that is deeply V-shaped in ventral view (Fig. 139).

#### 26. Hydrochasma aquia

Mathis & Zatwarnicki

http://species-id.net/wiki/Hydrochasma_aquia

Figs 144 –148

Hydrochasma aquia Mathis & Zatwarnicki 2010: 110 [United States. Virginia. Stafford: Aquia Harbour, Lions Park (38°27'N, 77°23.3'W); HT ♂, USNM].

##### Diagnosis.

**Figures 144–147.**
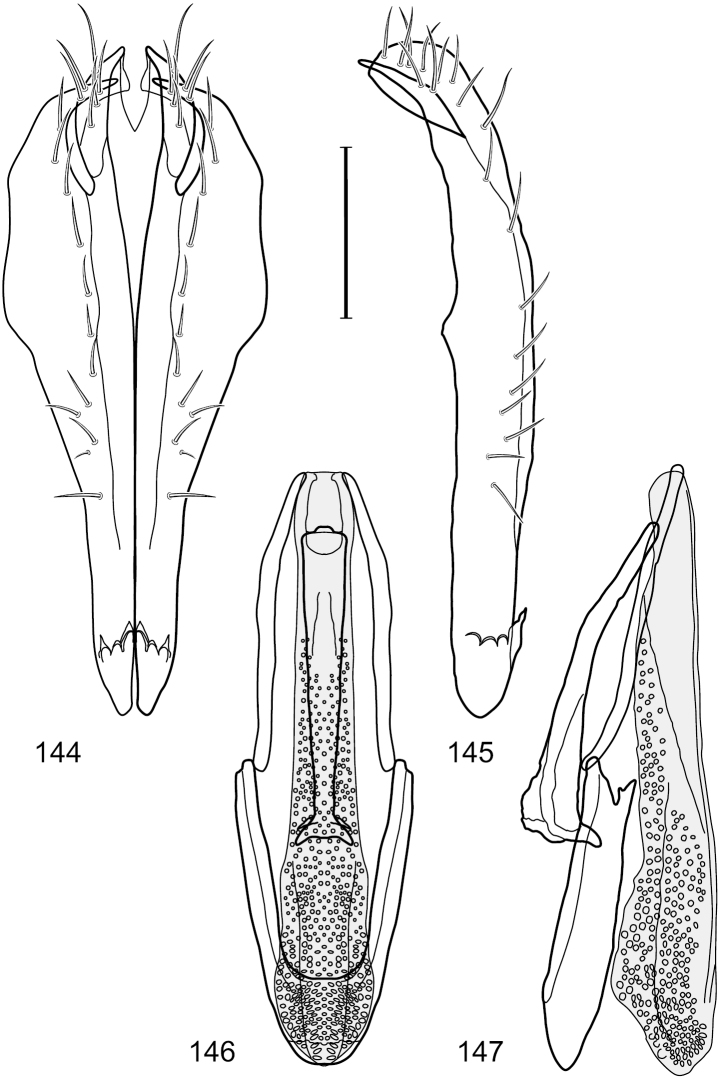
*Hydrochasma aquia* Mathis and Zatwarnicki (USA. Virginia. Stafford: Aquia Harbour, Lions Park) **144** epandrium and cerci, posterior view **145** same, lateral view **146** internal structures of male terminalia (aedeagus [shaded], phallapodeme, gonite, hypandrium), ventral view **147** same, lateral view. Scale bar = 0.1 mm.

This species is distinguished from congeners by the following combination of characters: Small shore flies, body length 1.50–1.95 mm. *Head*: Antenna mostly dark gray; parafacial silvery white, concolorous with facial coloration. Gena moderately high, height usually slightly higher than length of basal flagellomere; gena-to-eye ratio 0.28–0.30. *Thorax*: Wing hyaline; costal vein ratio 0.65–0.72; M vein ratio 0.56–0.60. Hindtibia with only pale, yellowish setae apicoventrally. *Abdomen*: Tergites lacking wedge-like marking laterally but tergites 2–4 with narrow to moderately wide medial area extensively dark slate gray to black; tergite 5 light gray to silvery gray with undifferentiated posterior margin, uniformly colored; medial coloration on tergites 1–4 narrow, sometimes only a stripe, slightly darker than color of lateral margins. Male terminalia (Fig. 144–147): Combined structures generally elongate, in posterior view (Fig. 144) height more than twice width, dorsal portion semi-rectangular, ventral portion narrowed; epandrium with dorsal arch above cerci attenuate, not connected, dorsal half more or less roundly rectangular, slightly higher than wide, thereafter ventrally tapered to narrow, almost parallel-sided processes, apex with taper more angulate, dorsal 2/3 bearing setulae, in lateral view (Fig. 145) mostly parallel sided with anterior margin with a midlength shallow prominence, dorsal half shallowly curved anteriorly, apical half parallel sided, straight, apex moderately narrowly rounded; cerci moderately short, height not quite twice width (Fig. 144), narrowly attached ventrally tenuously with epandrium; aedeagus in lateral view (Fig. 147) very elongate, sclerotized portion very narrow, with length almost 10× or more width, anteroapical, membranous portion only slightly expanded, width not much more than lateral width of hypandrium, membranous portion with numerous cuticular spicules, in ventral view (Fig. 146) also narrow and very elongate, almost parallel sided basally, membranous apical portion becoming wider apically, apex rounded; phallapodeme in lateral view (Fig. 147) narrow, elongate, unevenly clavate toward hypandrium, keel slightly developed, apical width, including keel, slightly less than greatest aedeagal width, in ventral view (Fig. 146) with hypandrial end narrowly linear as a T, with arms of T short and curved, thereafter toward base of aedeagus bar-like, slightly expanding; gonite in lateral view (Fig. 147) very narrow, elongate, very shallowly sinuous, in ventral view (Fig. 146) narrow, bar-like with apices toward base of aedeagus shallowly curved medially; hypandrium in lateral view (Fig. 147) elongate, moderately shallow, width more or less uniform and slightly less than 1/4 length, nearly straight, in ventral view (Fig. 146) generally deeply V-shaped with posterior margin very deeply V-shaped, anterior margin moderately narrow, bluntly rounded.

##### Type material.

The holotype male is labeled “**USA. V[IRGINI]A.** Stafford: Aquia Harbour, Lions Park (38°27'N, 77°23.3'W), 21 Jul 2008, D. & W. N. Mathis/USNM ENT 00118100 [plastic bar code label]/HOLOTYPE ♂ *Hydrochasma aquia* W. Mathis & T. Zatwarnicki USNM [red].” The holotype is double mounted (minuten in a block of plastic), is in excellent condition, and is deposited in the USNM. Sixteen paratypes (15♂, 1♀; USNM) bear the same label data as the holotype. Other paratypes are as follows:

VIRGINIA. **Culpeper:** Lake Pelham (38°27.8'N, 78°02.7'W), 28 Apr 2006, D. and W. N. Mathis (1♀; USNM). **Fairfax:** Great Falls (quarry; 38°59.1'N, 77°14.8'W; 50 m), 13 Jun 2007, D. and W. N. Mathis (3♂; USNM); Turkey Run (mouth; 38°57.9'N, 77°09.4'W), 25 May-7 Sep 2006, 2007, 2008, D. and W. N. Mathis (5♂, 1♀; USNM). **Henry:** Martinsville Reservoir (36°44.7'N, 79°52.2'W), 17 May 2005, D. and W. N. Mathis (1♀; USNM). **Madison:** Criglersville (1.6 km W; 38°28.4'N, 78°19.9'W; 185 m; Robinson River), 1 Jul 2005, D. and W. N. Mathis (1♂, 1♀; USNM). **Patrick:** Meadows of Dan (36°44.2'N, 80°22.9'W), 18 May 2005, D. and W. N. Mathis (2♂; USNM); Woolwine (36°47.4'N, 80°16.7'W; 300 m), 17 May 2005, D. and W. N. Mathis (1♂, 1♀; USNM). **Spotsylvania:** Rappahannock River (38°18.8'N, 77°32.5'W), 3 Jul 2007, D. and W. N. Mathis (1♂, 1♀; USNM). **Stafford:** Aquia Creek (38°29.1'N, 77°23.8'W), 6 Jun 2005, D. and W. N. Mathis (1♂; USNM); Falmouth (38°19.2'N, 77°28.1'W; Rappahannock River; 9 m), 26 May-6 Jun 2007, D. and W. N. Mathis (2♂, 2♀; USNM).

##### Type locality.

United States. Virginia. Stafford: Aquia Harbour, Lions Park (38°27'N, 77°23.3'W).

##### Other specimens examined.

Nearctic. UNITED STATES. DELAWARE. **Kent:** Woodland Beach (39°19.9'N, 75°28.3'W; beach), 18 May 2006, D. and W. N. Mathis (1♂; USNM).

DISTRICT OF COLUMBIA. Rock Creek, Milkhouse Ford (38°57.9'N, 77°02.9'W), 18 May 2007, D. and W. N. Mathis (1♀; USNM).

IOWA. **Louisa:** Oakville (42°09.8'N, 91°0.8'W), 10 Aug 1960, D. L. Deonier (12♂, 15♀; USNM).

WEST VIRGINIA. **Hardy:** Mathias (38°52.6'N, 78°52'W; 465 m), 13 Jul 2007, D. and W. N. Mathis (1♀; USNM); Trout Pond (38°57.4'N, 78°44.2'W; 595 m), 13 Jul 2007, D. and W. N. Mathis (1♀; USNM). **Mercer:** Ceres (Kee Reservoir; 37°18.4'N, 81°10.4'W; 757 m), 24 Sep 2007, D. and W. N. Mathis (2♂; USNM). **Summers:** Hinton (37°41.8'N, 80°53'W; New River; 427 m), 26 Sep 2007, D. and W. N. Mathis (1♂; USNM).

##### Distribution

**Figure 148.**
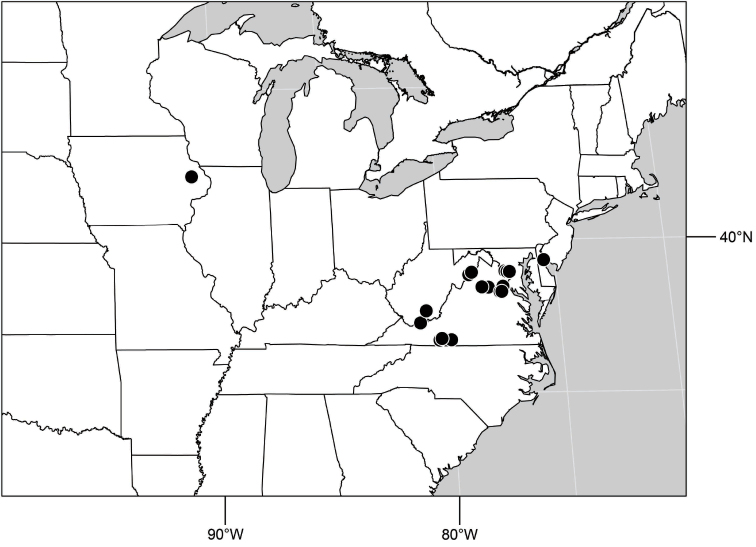
Distribution of *Hydrochasma aquia* Mathis and Zatwarnicki.

(Fig. 148). Nearctic: United States (Delaware, District of Columbia, Iowa, Virginia, West Virginia).

##### Etymology.

The species epithet, *aquia*, is a noun in apposition and is the name for the general area, including Aquia Creek and Aquia Harbour, where the type series of this species was collected. Aquia is the transliterated name of a Native American village (Powhatan Confederacy) located near mouth of Aquia Creek in present day Stafford County, Virginia. The name, which was originally spelled phonetically as “Quiyough,” apparently means gulls, a common bird on the creek.

##### Remarks.

This is a widespread species that is relatively common in the Nearctic Region. Externally, this species is very similar and evidently closely related to *Hydrochasma andeum*, *Hydrochasma avanae*, and *Hydrochasma annae* but can be distinguished from these species by having a hindtibia that bears only yellowish setulae ventrally near the apex, a tapered ventral epandrial process, and by having a more delicately developed hypandrium that is deeply U-shaped in ventral view (Fig. 146).

#### 27. Hydrochasma avanae

Mathis & Zatwarnicki

http://species-id.net/wiki/Hydrochasma_avanae

Figs 149 –153

Hydrochasma avanae Mathis and Zatwarnicki 2010: 113 [United States. Utah. Grand: Swasey Beach (15.3 km N Green River; 39°07'N, 110°06.6'W; Green River; 1255 m); HT ♂, USNM].

##### Diagnosis.

**Figures 149–152.**
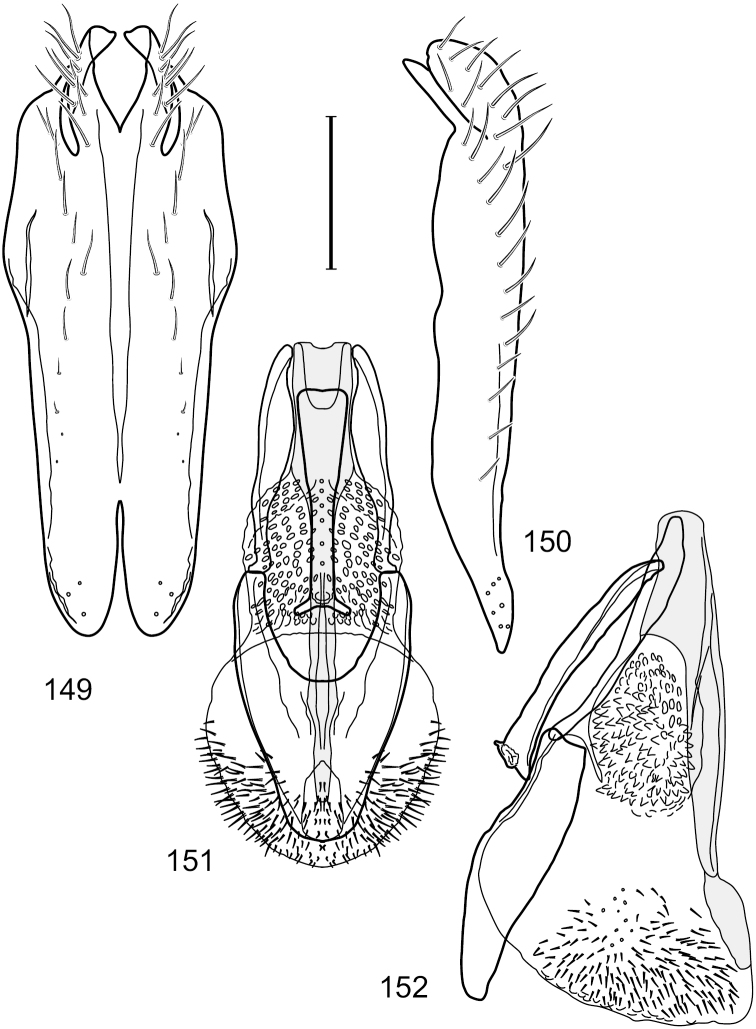
*Hydrochasma avanae* Mathis and Zatwarnicki (USA. Virginia. Fairfax: Turkey Run) **149** epandrium and cerci, posterior view **150** same, lateral view **151** internal structures of male terminalia (aedeagus [shaded], phallapodeme, gonite, hypandrium), ventral view **152** same, lateral view. Scale bar = 0.1 mm.

This species is distinguished from congeners by the following combination of characters: Small shore flies, body length 1.30–1.85 mm. *Head*: Antenna mostly dark gray; parafacial silvery white, concolorous with facial coloration. Gena moderately high, height usually slightly higher than length of basal flagellomere; gena-to-eye ratio 0.71–0.73. *Thorax*: Wing hyaline; costal vein ratio 0.66–0.82; M vein ratio 0.45–0.50. Hindtibia bearing an apical black seta ventrally. *Abdomen*: Tergites lacking wedge-like marking laterally but tergites 2–4 with wide medial area extensively dark slate gray to black; tergite 5 light gray to silvery gray with undifferentiated posterior margin, uniformly colored; medial coloration on tergites 1–4 narrow, sometimes only a stripe, slightly darker than color of lateral margins. Male terminalia (Figs 149–152): Combined structures generally elongate, in posterior view (Fig. 149) height about 2.5× width; epandrium with dorsal arch above cerci attenuate, not connected, dorsal 1/3–1/2 somewhat quadrate with angles rounded, ventral portion as 2 digitiform, thick, parallel lobes that connect subapically for a short distance, ventral margin moderately narrowly rounded with deep, very narrow, apical incision medially, dorsal half bearing setulae, in lateral view (Fig. 150) mostly parallel sided with anterior shallowly sinuous, apical 1/3 tapered to sharply rounded apex; cerci short, in lateral view height about twice width (Fig. 150), narrowly semicircular, not attached with epandrium; aedeagus in lateral view (Fig. 152) elongate, with length of sclerotized portion about 5× width, with greatly enlarged apical and ventral, membranous portion, shape in lateral view (Fig. 152) triangular, with apex broad and shallowly arched, thereafter toward base tapered to narrow, bluntly rounded margin, membrane bearing sub-basally numerous scale-like spicules, in ventral view (Fig. 151) also showing expanded apex, basal 1/3 narrowed, thereafter with middle portion ballooning out to more than twice basal width, apical 1/3 greatly expanded, broadly obovate; phallapodeme in lateral view (Fig. 152) narrow, elongate, evenly shallowly arched, keel weakly developed, barely evident at end toward attachment with hypandrium, in ventral view (Fig. 151) with hypandrial end narrowly T-shaped, thereafter toward base of aedeagus gradually expanded to blunt apex; gonite in lateral view (Fig. 152) narrow, elongate, bar-like with slightly curvature, in ventral view (Fig. 151) moderately broad but with apices narrowed, especially end toward hypandrium; hypandrium in lateral view (Fig. 152) elongate, moderately shallow, width slightly more than 1/4 length, very slightly angulate, in ventral view (Fig. 151) generally V-shaped with posterior margin deeply U-shaped, anterior margin moderately narrowly rounded.

##### Type material.

The holotype male is labeled “**USA**. **UT[AH].** Grand: Green River (15.3 km N; 39°7'N, 110°6.6'W; 1255 m), 15 Aug 2008,D.&W.N.Mathis/USNM ENT 00118101 [plastic bar code label]/HOLOTYPE ♂ *Hydrochasma avanae* W. Mathis & T. Zatwarnicki USNM [red].” The holotype is double mounted (minuten in a block of plastic), is in excellent condition, and is deposited in the USNM. Forty-one paratypes (32♂, 9♀; USNM) bear the same label data as the holotype. Other paratypes are as follows: UTAH. **Emery:** Green River (3 km N; 38°59'N, 110°09'W), 5 Aug 1992, D. and W. N. Mathis (7♂, 4♀; USNM); Green River (Green River; 38°59.6'N, 110°08.5'W; 1240 m), 31 Jul 2007, D. and W. N. Mathis (6♂, 1♀; USNM); San Rafael River (22.5 km SW Green River; 38°55.7'N, 110°24.5'W; 1270 m), 31 Jul 2007, D. and W. N. Mathis (1♀; USNM). **Grand:** Crystal Geyser (14.5 km SE Green River; 38°56.3'N, 110°08.1'W), 15 Aug 2008, D. and W. N. Mathis (27♂, 1♀; USNM); Swasey Beach (15.3 km N Green River; 39°07'N, 110°06.6'W; Green River; 1255 m), 29 May-30 Jul 2007, 2008, D. and W. N. Mathis (7♂, 1♀; USNM); Thompson Spring (8.9 km N Thompson Springs; 39°02.3'N, 109°43.4'W; 1740 m), 1-16 Aug 2007, 2008, D. and W. N. Mathis (9♂, 4♀; USNM).

##### Type locality.

United States. Utah. Grand: Swasey Beach (15.3 km N Green River; 39°07'N, 110°06.6'W; Green River; 1255 m).

##### Other specimens examined.

Nearctic. UNITED STATES. ARIZONA. **Mojave:** Littlefield (36°53.1'N, 113°55.8'W), 1 May 2002, D. and W. N. Mathis (4♂, 4♀; USNM).

CALIFORNIA. **San Bernardino:** Afton (35°02.3'N, 116°22.9'W), 28 Apr 2002, D. and W. N. Mathis (1♀; USNM).

GEORGIA. **Floyd:** Lavender (34°17.5'N, 85°20.5'W), 23 Aug 1910, J. C. Bradley (1♂; ANSP). **Tift:** Tifton (31°27'N, 83°30.5'W), 6 Sep-Oct 1896 (1♂, 2♀; ANSP).

ILLINOIS. **Cook:** Chicago (41°53'N, 87°37.9'W) (1♂, 1♀; ANSP).

INDIANA. **Tippecanoe:** Lafayette (40°25'N, 86°52.5'W), 24 May-5 Sep 1916, E. W. Stafford (2♂; ANSP).

MICHIGAN. **Kalamazoo:** Kalamazoo (42°17.5'N, 85°35.2'W), 10 May 1936, C. W. Sabrosky (1♂; ANSP).

NEVADA. **Clark:** Las Vegas Wash (36°05.4'N, 114°58.7'W), 11 Apr 2005, D. and W. N. Mathis (2♂; USNM).

NEW MEXICO. **Catron:** Gila River (33°13.6'N, 106°15.1'W; 1750 m), 15 Aug 2007, D. and W. N. Mathis (4♂; USNM). **Chaves:** Roswell (9 km E; Pecos River; 33°23.8'N, 104°24'W; 1060 m), 10 Aug 2007, D. and W. N. Mathis (7♂, 3♀; USNM). **Grant:** Bill Evans Lake (32°52.1'N, 108°34.5'W; 1416 m), 14 Aug 2007, D. and W. N. Mathis (1♂; USNM); Mimbres River (NM Hwy. 61 & Royal John Mine Road; 32°43.8'N, 107°52'W; 1665 m), 13-22 Aug 2007, 2009, D. and W. N. Mathis, T. Zatwarnicki (4♂, 4♀; USNM). **Lincoln:** Capitan (3.2 km E; Salado Creek; 33°32.6'N, 105°32.3'W; 1890 m), 10 Aug 2007, D. and W. N. Mathis (11♂; USNM). **Otero:** Alamagordo (32°54'N, 105°57.6'W), 6-12 May 1902 (1♂, 1♀; ANSP). **Valencia:** Río Puerco (34°47.8'N, 106°59.5'W; 1575 m), 9 Aug 2007, D. and W. N. Mathis (4♂, 3♀; USNM).

OKLAHOMA. **Pontotoc:** Ada (34°46.5'N, 96°40.7'W), 18 Jul 1937, Standish-Kaiser (1♂; ANSP).

TENNESSEE. **Shelby:** Meeman Shelby State Park (Mississippi River; 35°22.2'N, 90°04.5'W; 73 m), 10 Jun 2004, W. N. Mathis (8♂, 4♀; USNM).

TEXAS. **Augustine:** Rayburn Park (31°04'N, 95°05'W), 15 May 1993, D. and W. N. Mathis (1♀; USNM). **Fort Bend:** Sugar Land (29°37.2'N, 95°38.1'W), 11 Jul 1933, R. Nabors, C. W. Sabrosky (1♂; ANSP). **Hidalgo:** Bentsen, Rio Grande Valley State Park (26°10.4'N, 98°23'W), 30- Nov-2 Dec 1978, E. E. Gris sell, A. S. Menke (1♀; USNM). **Jim Wells:** Mathis (7.5 km S; Nueces River; 28°02.2'N, 97°52.2'W; 15 m), 6 Jun 2004, W. N. Mathis (4♂, 1♀; USNM). **Kimble:** Junction (South Llano River; 30°29.6'N, 99°45.1'W; 510 m), 4 Jun 2004, W. N. Mathis (1♀; USNM). **Travis:** Austin (Zilker Park; 30°15.8'N, 97°46.3'W), 2 Jun 2004, W. N. Mathis (2♂, 1♀; USNM).

UTAH. **Garfield:** Willow Tank, Hurricane Wash (37°23.2'N, 111°08'W), 22 May 2001, D. and W. N. Mathis (5♂, 1♀; USNM). **Kane:** Drip Tank Canyon (37°19.4'N, 111°31.8'W), 15 May 2001, D. and W. N. Mathis (2♂; USNM); Kanab (6.5 km N; 37°08.7'N, 112°32.4'W), 14 May 2001, D. and W. N. Mathis (1♀; USNM). **San Juan:** Blanding (Reservoir 4; 37°39.9'N, 109°29.7'W; 1975 m), 20 Aug 2013, D. and W. N. Mathis (7♂, 5♀; USNM); Blanding, Recapture Reservoir (37°39.9'N, 109°26.5'W; 1840 m), 23 Jul 2013, D. and W. N. Mathis (7♂, 4♀; USNM); Johnson Creek meadow (22 km N Blanding: 37°47.5'N, 109°30.5'W; 2384 m), 20 Aug 2013, D. and W. N. Mathis (1♀; USNM).

VIRGINIA. **Essex:** Tappahannock (37°55.8'N, 76°51.4'W; Rappahannock River), 18 Sep 2004, D. and W. N. Mathis (1♂; USNM). **Fairfax:** Great Falls (Patowmack Canal; 39°00.1'N, 77°15.2'W), 3 Oct 2006, D. and W. N. Mathis (1♂; USNM); Turkey Run (mouth; 38°57.9'N, 77°09.4'W), 10 Jul-7 Sep 2006, 2007, 2008, D. and W. N. Mathis, H. B. Williams (9♂, 3♀; USNM). **Patrick:** Meadows of Dan (36°44.2'N, 80°22.9'W), 18 May 2005, D. and W. N. Mathis (2♀; USNM). **Stafford:** Aquia Harbour, Lions Park (38°27'N, 77°23.3'W), 21 Jul-4 Sep 2006, 2008, D. and W. N. Mathis (5♂; USNM); Aquia Landing (38°23.2'N, 77°19'W), 7 May 2005, D. and W. N. Mathis (2♂, 1♀; USNM); Falmouth (38°19.2'N, 77°28.1'W; Rappahannock River; 9 m), 26 May-30 Jun 2007, D. and W. N. Mathis (4♂; USNM).

WASHINGTON. **Franklin:** Columbia River N of Pasco (46°21.5'N, 119°15.5'W), 29 Jul 1998, W. N. Mathis (6♂; USNM); Lake Kahlotus (46°39'N, 118°32'W), 25 Jul 1992, D. and W. N. Mathis (1♀; USNM); Levey (46°05'N, 118°52'W; Snake River), 27 Jul 1992, D. and W. N. Mathis (27♂, 8♀; USNM); Ringold (NW Pasco; 46°30.4'N, 119°15.3'W; Columbia River), 30 Jul 1998, D. and W. N. Mathis (2♂; USNM); Ringold (4.8 km N; 46°30.9'N, 119°15.3'W), Columbia River, 2 Jul 1988, D. and W. N. Mathis (1♂, 1♀; USNM); Snake River, Levey Landing (46°05'N, 118°52'W), 27 Jul 1992, D. and W. N. Mathis (29♂, 7♀; USNM). **Klickitat:** Glenn Wood, Klickitat River (45°41.8'N, 121°17.5'W), 17 Jun 1917, A. L. Melander (1♀; ANSP). **Whitman:** Pullman (3.2 km NW; 46°44'N, 117°10.9'W), 11 Jul 1988, D. and W. N. Mathis (1♂; USNM).

WEST VIRGINIA. **Mercer:** Ceres (Kee Reservoir; 37°18.4'N, 81°10.4'W; 757 m), 24 Sep 2007, D. and W. N. Mathis (2♂; USNM). **Summers:** Hinton (37°41.8'N, 80°53'W; New River; 427 m), 26 Sep 2007, D. and W. N. Mathis (2♂, 2♀; USNM). **Wyoming:** R. D. Bailey Lake (37°35.7'N, 81°46.8'W; 324 m), 25 Sep 2007, D. and W. N. Mathis (1♂, 1♀; USNM).

##### Distribution

**Figure 153.**
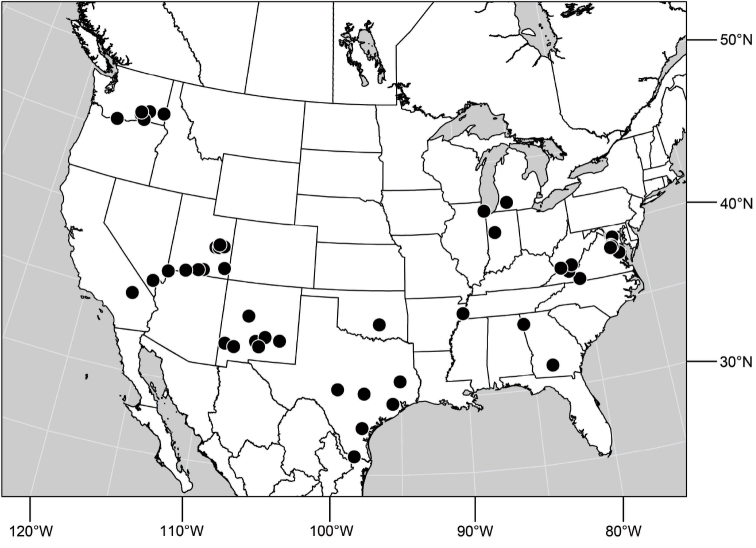
Distribution of *Hydrochasma avanae* Mathis and Zatwarnicki.

(Fig. 153). Nearctic: United States (Arizona, California, Georgia, Illinois, Indiana, Michigan, Nevada, New Mexico, Oklahoma, Tennessee, Texas, Utah, Virginia, Washington, West Virginia).

##### Etymology.

The species epithet, *avanae*, is a feminine genitive patronym to recognize Ms. Avan Thayn who guided us to the type locality.

##### Remarks.

This is a widespread species that is relatively common in the Nearctic Region. Externally, this species is very similar and evidently closely related to *Hydrochasma andeum* and *Hydrochasma annae* but can be distinguished from either of these two species by having a hindtibia that bears a black setula ventrally near the apex, a nearly parallel-sided ventral epandrial process, and by having a more robustly developed hypandrium, especially the anterior two-thirds (Fig. 151). In addition, the apical portion of the aedeagus is frequently greatly inflated (best seen in lateral view; Fig. 152)

#### 28. Hydrochasma capsum sp. n.

http://zoobank.org/C805867D-2672-42C3-9BB9-5AD506F45651

http://species-id.net/wiki/Hydrochasma_capsum

Figs 154 – 161 , 184

##### Diagnosis.

This species is distinguished from other congeners by the following combination of characters: Small to moderately small shore flies, body length 1.25–2.15 mm. *Head*: Antenna mostly dark gray; parafacial silvery white, concolorous with facial coloration; gena-to-eye ratio 0.16–0.17. *Thorax*: Wing with costal vein ratio 0.68–0.71; M vein ratio 0.60–0.62. Forecoxa mostly to entirely yellow, at most with basal margin gray to silvery gray. *Abdomen*: Tergites 1–4 with dorsum extensively grayish black to slate black, sharply contrasted along an even line with gray to silvery gray lateral margins (margins sometimes on venter), lacking gray wedges along lateral margins; tergite 5 of male with gray anterior margin, posterior portion with a large, medial, oval, black spot (Fig. 184). Male terminalia (Figs 157–160): Combined structures generally moderately elongate, in posterior view (Fig. 157) height slightly more than twice width, generally setulose but with setulae on ventral 1/3 smaller than those on dorsal 2/3; epandrium with dorsal arch above cerci roundly truncated, not connected, in posterior view (Fig. 157) with wide medial area membranous, narrowest at midlength, sclerotized ventral portion (along margin) tapered to sharp point, ventral margin very broadly rounded, in lateral view (Fig. 158) with posterior margin more or less evenly curved, anterior margin with a shallow protuberance at ventral 1/3; cerci moderately long, height nearly twice width, narrowly semi-hemispherical (Fig. 157), not attached lateroventrally with epandrium; aedeagus in lateral view (Fig. 160) complex, robust, moderately deeply bilobed, base wide, width just slightly less than length of gonite, anterior lobe narrow, bearing hairs on apicoposterior margin, posterior lobe wider, membranous, in ventral view (Fig. 159) relatively narrow on basal 1/3, thereafter slighter wider, apex broadly rounded; phallapodeme in lateral view (Fig. 160) elongate, narrow, rod-like, mostly parallel sided, apex at hypandrial end, widened, in ventral view (Fig. 159) an elongate, T-shaped process with width of crossbar about 1/3 length, vertical portion parallel sided, straight; gonite in lateral view (Fig. 160) narrow, elongate, bar-like, with shallow zigzag medially, in ventral view (Fig. 159) bar-like, slightly sinuous; hypandrium in lateral view (Fig. 160) pointed anteriorly, becoming wider posteriorly, in ventral view (Fig. 159) robustly W-shaped with medioanterior cleft short, depth less than crossbar of phallapodeme, each lateral portion wide, robustly developed, posterior emargination widely V-shaped.

**Figures 154–156.**
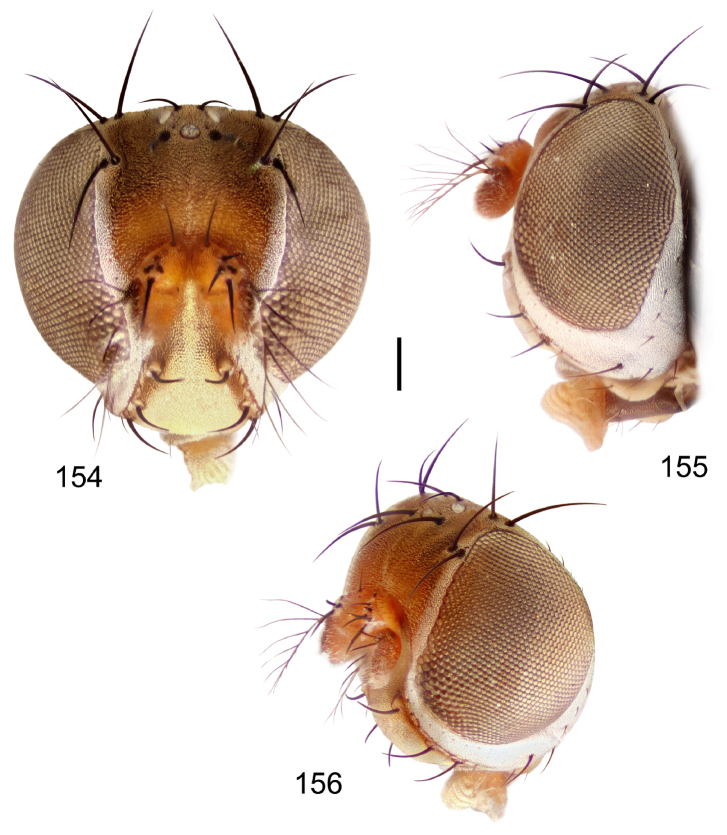
*Hydrochasma capsum* sp. n. (Puerto Rico. Maricao) **154** head, anterior view **155** same, lateral view **156** same, oblique view. Scale bar = 0.1 mm.

**Figures 157–160.**
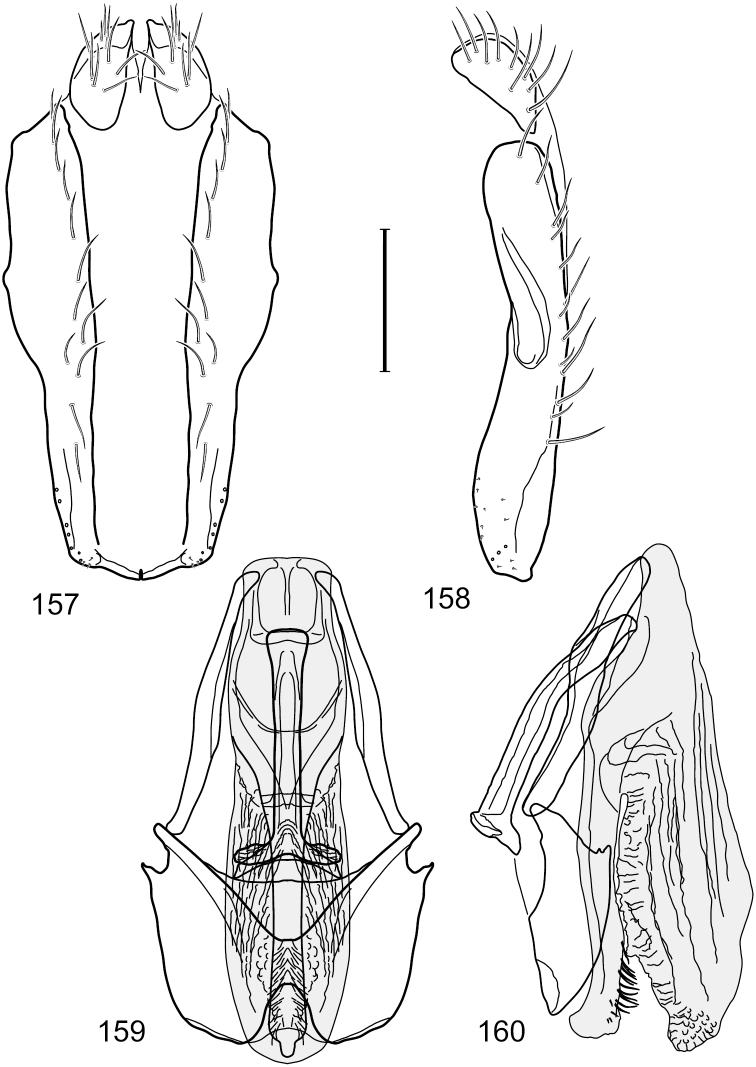
*Hydrochasma capsum* sp. n. (Puerto Rico. Rio Hoconuco) **157** epandrium and cerci, posterior view **158** same, lateral view **159** internal structures of male terminalia (aedeagus [shaded], phallapodeme, gonite, hypandrium), ventral view **160** same, lateral view. Scale bar = 0.1 mm.

##### Type material.

The holotype male of *Hydrochasma capsum* is labeled “ECUADOR. Prt.Or[e]ll[a]na: RíoTiputini (0°38.2'S, 76°8.9'W)[,] 12–26Aug 1999, W. N. Mathis, A. Baptista, M. Kotrba/USNM ENT 00117968 [plastic bar code label]/HOLOTYPE ♂ *Hydrochasma capsum* Mathis & Zatwarnicki, USNM [red].” The holotype is double mounted (minuten in a block of plastic), is in excellent condition, and is deposited in the USNM. Sixty-three paratypes (58♂, 5♀; USNM) bear the same label data as the holotype.

##### Type locality.

Ecuador. Orellana: RíoTiputini (0°38.2'S, 76°8.9'W).

##### Other specimens examined.

Neotropical. BOLIVIA. **La Paz:** Guanay (3 km E; 15°30.2'S, 67°52.3'W; 500 m), 14 Mar 2001, W. N. Mathis (3♂; USNM); Mapiri (15°18.6'S, 68°13'W; 720 m), 15–17 Mar 2001; S. D. Gaimari, W. N. Mathis (1♀; USNM).

BRAZIL. **Paraná:** Matinhos (N.; 25°46.4'S, 48°30.8'W; 3 m; beach/estuary), 25 Mar 2010, D. and W. N. Mathis (1♂; DZUP, USNM). **São Paulo:** Ubatuba, Praia Puruba (23°21'S, 44°55.6'W; beach), 29–30 Mar 2010, D. and W. N. Mathis (18♂, 2♀; USNM).

COSTA RICA. **Cartago:** Pejibaye (09°48.1'N, 83°42.7'W; La Reserva Biológica del Copal; 1090 m), 4 Apr 2005, J. Azofeifa, D. Briceño (2♂, 8♀; INBio). **Guanacaste:** Bagaces Fortuna Z.P. Miravalles (10°43.1'N, 84°51.3'W; Sendero Cabro Muco; 980 m), 8–31 Jul 2002, J. D. Gutierrez (2♂, 3♀; INBio); Parque Nacional Santa Rosa (Bosque San Emilio; 10°50.6'N, 85°36.8'W; 300 m), 12–14 Jun 2002, D. Briceño (1♂; INBio).

ECUADOR. **Pastaza:** Puyo (01°29'S, 77°59.7'W; black light), 16 May 1977, D. R. Givens, P. J. Spangler (54♂, 29♀; USNM).

GUYANA. Conservation of Ecological Interactions and Biotic Associations (CEIBA; ca. 40 km S Georgetown; 06°29.9'N, 58°13.1'W), 13–21 Apr 1994, 1995, 1997, W. N. Mathis (7♂; USNM); Georgetown (06°48.6'N, 58°08.6'W), 20–29 Aug 1997, W. N. Mathis (2♀; USNM); Karanambo, Rupununi River (ox bow; 03°45.1'N, 59°18.6'W), 2 Apr 1994, W. N. Mathis (1♂, 1♀; USNM); Kato, Chiung River (04°39.7'N, 59°50.0'W), 1 May 1995, W. N. Mathis (5♂, 5♀; USNM); Kumu River and Falls (25 km SE Lethem in Kanuku Mountains; 03°15.9'N, 59°43.6'W), 4–30 Apr 1994, 1995, W. N. Mathis (4♂, 3♀; USNM); Wiruni River (05°46.6'N, 58°01'W), 11 Apr 1994, W. N. Mathis (1♂, 4♀; USNM).

TRINIDAD and TOBAGO. Tobago. **St. John:** Charlotteville (5 km S; Hermitage River and beach; 11°18.9'N, 60°34.2'W), 10 Jun 1993, D. and W. N. Mathis (1♂; USNM).

West Indies. CUBA. **Cienfuegos:** Topes de Collantes (5 km WNW; 21°56.5'N, 80°2.3'W; 600 m), 11 Dec 1994, W. N. Mathis (1♂, 2♀; USNM). **Pinar del Rio:** Soroa (22°47.7'N, 83°W), 27–28 Apr 1983, W. N. Mathis (1♂; USNM). **Sancti Spiritus:** Topes de Collantes (21°54.4'N, 80°01.4'W; 670 m), 9–11 Dec 1994, W. N. Mathis (4♂, 14♀; USNM).

DOMINICAN REPUBLIC. **Barahona:** Baoruco (beach and river; 18°04.6'N, 71°05.5'W), 19 May 1998, D. and W. N. Mathis (6♂; USNM); Paraíso (5 km N; 18°01.5'N, 71°11.6'W; 150 m), 21 Mar 1999, W. N. Mathis (1♂; USNM); San Rafael (18°01.9'N, 71°08.4'W), 22 Mar 1999, W. N. Mathis (1♂; USNM). **El Seibo:** Pedro Sáchez (18°51.4'N, 69°6.5'W), 26 May 1998, D. and W. N. Mathis (1♂; USNM). **Independencia:** Duvergé (2 km S; 18°22'N, 71°31.4'W), 24 Mar 1999, D. and W. N. Mathis (5♂, 1♀; USNM); Puerto Escondido (18°19.6'N, 71°35'W; 1370 m), 24 Mar 1999, D. and W. N. Mathis (1♂; USNM). **La Vega:** El Rio (9.5 km E; 19°0.9'N, 70°33.5'W; 980 m), 6–24 May 1995, 1998, D. and W. N. Mathis (16♂, 3♀; USNM); Constanza (15.2 km SE; 18°60.3'N, 70°40.9'W; 1580 m), 7 May 1995, W. N. Mathis (1♂; USNM); Jarabacoa (1–2 km S; 19°06.9'N, 70°37'W; 520 m), 8–21 May 1995, 1998, D. and W. N. Mathis (3♂, 4♀; USNM); La Cienega de Manabao (19°03.9'N, 70°51.8'W; 1050 m), 28 Mar 1999, W. N. Mathis (14♂, 5♀; USNM); Jarabacoa (5 km S; 19°05.8'N, 70°36.5'W; 640 m), 8–20 May 1995, W. N. Mathis (1♀; USNM); Río Camu (3.5 km NW La Vega; 19°13.8'N, 70°35.2'W; 100 m), 18 May 1998, D. and W. N. Mathis (1♂, 1♀; USNM); Salto Baiguate (near Jarabacoa; 19°05.5'N, 70°36.9'W; 570 m), 9 May 1995, W. N. Mathis (8♂, 2♀; USNM); Salto Guasara (near Jarabacoa; 19°04.4'N, 70°42.1'W; 680 m), 9 May 1995, W. N. Mathis (4♂; USNM). **Monseñor Nouel:** near Jima (19°01.6'N, 70°28.8'W; 670 m), 24 May 1998, D. and W. N. Mathis (2♂; USNM). **Pedernales:** Alcoa Road (km 30; N Cabo Rojo; 18°07.3'N, 71°35.8'W; 1080 m), 20 Mar 1999, W. N. Mathis (1♂, 1♀; USNM); Pedernales (19 km N; 18°09.2'N, 71°44.8'W; 230 m), 20 Mar 1999, W. N. Mathis (4♂; USNM). **Puerto Plata:** Río Camu (14 km E Puerto Plata; 19°41.9'N, 70°37.4'W), 17–23 May 1995, 1998, D. and W. N. Mathis (6♂; USNM).

JAMAICA. **Clarendon:** Grantham (18°09.3'N, 77°23.8'W; 340 m), 16 Apr 2000, W. N. Mathis (3♂, 2♀; USNM). **Manchester:** Mandeville (18°03.5'N, 77°31.9'W), 7–13 May 1996, D. and W. N. Mathis, H. B. Williams (2♂, 2♀; USNM). **Portland:** Crystal Springs (18°12.5'N, 76°37.9'W), 18 May 1996, D. and W. N. Mathis, H. B. Williams (1♂, 1♀; USNM); Berridale (18°06.5'N, 76°20'W), Rio Grande River, 25 Apr 2000, W. N. Mathis (4♂, 1♀; USNM); Green Hills (18°05.7'N, 76°43'W; 780 m), 28 Apr 2000, W. N. Mathis (1♂; USNM). **St. Andrew:** Mavis Bank (1.7 km E; 18°02.4'N, 77°39.5'W; 575 m), Yallahs River, 21–22 Apr-1 May 2000, W. N. Mathis (7♂, 7♀; USNM); Mavis Bank (4.3 km SE; 18°01.4'N, 76°38.1'W; 480 m); Yallahs River, 22–23 Apr 2000, W. N. Mathis (3♂, 1♀; USNM); Mavis Bank (near coffee factory; 18°01.4'N, 76°39.7'W; waterfall), 21–23 Apr 2000, W. N. Mathis (1♂, 2♀; USNM); Silver Hill Gap (18°05.3'N, 76°43'W; 940 m), 29 Apr 2000, W. N. Mathis (1♂; USNM). **St. Elizabeth:** Ys Falls (18°09.3'N, 77°49.5'W), 17–18 Apr 2000, W. N. Mathis (7♂, 5♀; USNM). **St. Mary:**Annotto Bay (marsh), 25 Feb 1969, W. W. Wirth (2♂, 1♀; USNM). **St. Thomas:** Bath Fountain Spring (17°57.6'N, 76°21.3'W), 15 May 1996, D. and W. N. Mathis, H. B. Williams (2♂, 1♀; USNM); Bath River, Bath (17°56.8'N, 76°21.6'W), 16 May 1996, D. and W. N. Mathis, H. B. Williams (2♂; USNM); Hagley Gap (1 km E; 18°00.1'N, 76°36.7'W), 16 May 1996, D. and W. N. Mathis, H. B. Williams (2♂, 1♀; USNM); Mt. Lebanus (17°58.2'N, 76°32.7'W), 16 May 1996, D. and W. N. Mathis, H. B. Williams (2♂; USNM); Yallahs River (mouth; 17°53'N, 76°35.6'W), 14 May 1996, D. and W. N. Mathis, H. B. Williams (4♂, 2♀; USNM).

PUERTO RICO. Maricao (18°11.1'N, 66°58.9'W), 21 Sep 1995, D. and W. N. Mathis (9♂, 7♀; USNM); Maricao, Los Viveros (18°10.5'N, 66°59.2'W), 21 Sep 1995, D. and W. N. Mathis (1♂; USNM); Río Hoconuco (18°7.6'N, 67°2.6'W), 20 Sep 1995, D. and W. N. Mathis (6♂, 1♀; USNM).

ST. LUCIA. Dauphin Boguis (1.6 km S Marquis; 14°01'N, 60°55'W), 17 Jun 1991, D. and W. N. Mathis (1♂, 1♀; USNM).

##### Distribution

**Figure 161.**
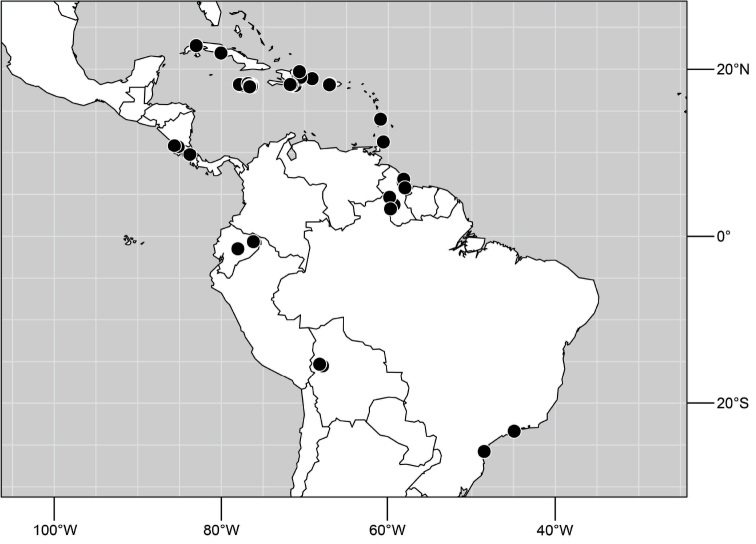
Distribution of *Hydrochasma capsum* sp. n.

(Fig. 161). Neotropical: Bolivia (La Paz), Brazil (Paraná, São Paulo), Costa Rica (Cartago, Guanacaste), Ecuador (Orellana, Pastaza), Guyana, Trinidad and Tobago, West Indies (Cuba, Dominican Republic, Jamaica, Puerto Rico, St. Lucia).

##### Etymology.

The species epithet, *capsum*, is of Latin derivation and means case, referring to the rectangular, case-like shape of the male terminalia in posterior view.

##### Remarks.

This species is closely related to *Hydrochasma robustum*, especially the similar shapes of their respective epandriums in posterior view, but it can be distinguished from that species by the more broadly developed hypandrium in ventral view (wider than long) and the lack of basal, hypandrial notches.

#### 29. Hydrochasma edmistoni sp. n.

http://zoobank.org/4287AC4A-A5A0-44B1-872A-9FEF6C7B9F89

http://species-id.net/wiki/Hydrochasma_edmistoni

Figs 162 –166

##### Diagnosis.

**Figures 162–165.**
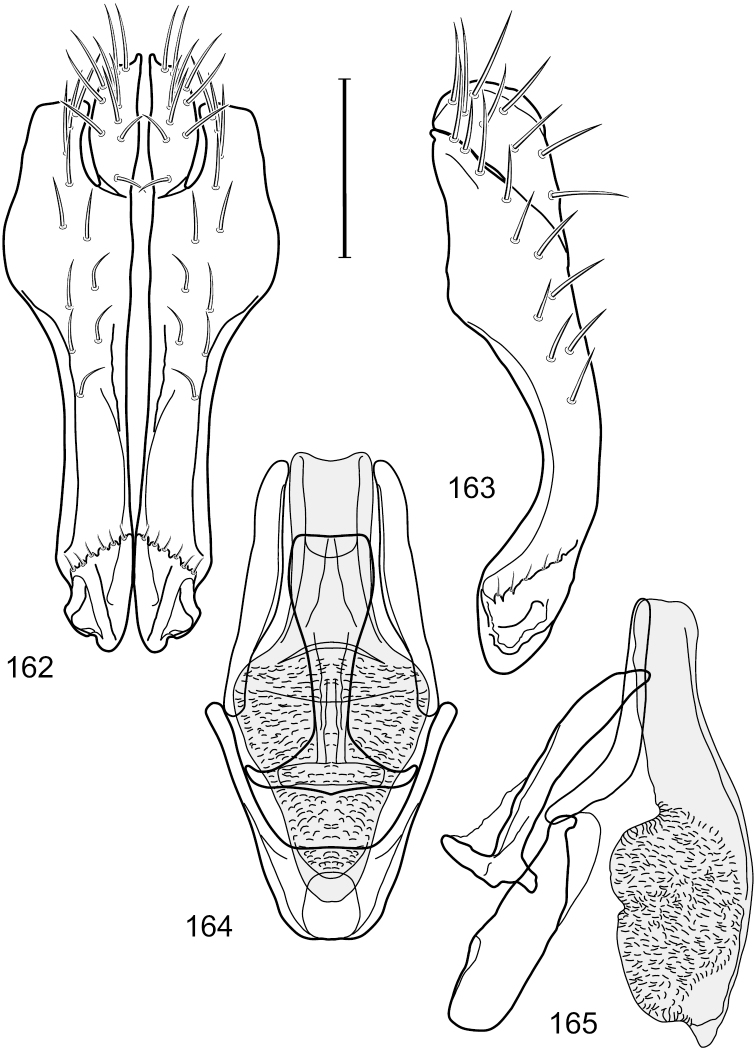
*Hydrochasma edmistoni* sp. n. (Dominican Republic. La Vega: La Vega) **162** epandrium and cerci, posterior view **163** same, lateral view **164** internal structures of male terminalia (aedeagus [shaded], phallapodeme, gonite, hypandrium), ventral view **165** same, lateral view. Scale bar = 0.1 mm.

This species is distinguished from other congeners by the following combination of characters: Small shore flies, body length 1.55–1.75 mm. *Head*: Antenna mostly dark gray; parafacial silvery white, concolorous with facial coloration; gena-to-eye ratio 0.16–0.17. *Thorax*: Wing with costal vein ratio 0.67–0.69; M vein ratio 0.52–0.54. Forecoxa mostly blackish gray to gray. *Abdomen*: Tergites 1–4 extensively brownish black to blackish gray dorsally, lacking lateral wedges. Male terminalia (Figs 162–165): Combined structures generally moderately elongate, in posterior view height twice width, excluding cerci, generally setulose dorsally, setulae sparse or lacking ventrally; epandrium with dorsal arch above cerci interrupted, not connected, in posterior view (Fig. 162) with basal half rectangular with angles rounded, apical half narrowed, extended as abutting, narrow, almost parallel sided processes until irregularly rounded apex, rounded apex with broad, shallow triangular extension; ventral extensions of epandrium with subapical, shallowly V-shaped structure that bears setulae, in lateral view (Fig. 163) generally moderately deeply arched with curvature more evident medially, apex rounded; cerci moderately short, height nearly twice width, semi-hemispherical (Fig. 162), attached ventrally with epandrium; aedeagus in lateral view (Fig. 165) elongate, about 4× longer than subapical width, tubular on basal half, mostly parallel sided, expanded on apical half, in ventral view (Fig. 164) almost diamond shaped with longer basal extension being parallel sided, apex rounded; phallapodeme in lateral view (Fig. 165) narrowly elongate, moderately expanded toward anterior margin, keel as a narrow, digitiform process, in ventral view (Fig. 164) an elongate T with arms moderately wide, extended vertical shaft expanded to aedeagal base, this end truncate; gonite in lateral view (Fig. 165) narrowly elongate, bar-like, very shallowly V-shaped, in ventral view (Fig. 164) narrowly elongate, slightly swollen medially; hypandrium in lateral view (Fig. 165) elongate and comparatively narrow, width less than aedeagal base, posterior margin tapered to a slightly recurved point, anterior margin truncate, in ventral view (Fig. 164) deeply V-shaped, with vertex robustly developed and extended arms narrow, elongate anterior margin bluntly rounded.

##### Type material.

The holotype male of *Hydrochasma edmistoni* is labeled “*DOMINICAN REPUBLIC. Azua*: near Pueblo Viejo (18°24.8'N, 70°44.7'W), 19 May 1998, D. and W. N. Mathis/USNM ENT 00087658 [plastic bar code label]/HOLOTYPE ♂ *Hydrochasma edmistoni* Mathis & Zatwarnicki, USNM [red].” The holotype is double mounted (minuten in a block of plastic), is in excellent condition, and is deposited in the USNM. Paratypes are as follows: DOMINICAN REPUBLIC. **La Vega:** Río Camu (3.5 km NW La Vega; 19°13.8'N, 70°35.2'W; 100 m), 18 May 1998, D. and W. N. Mathis (1♂; USNM).

##### Type locality.

Dominican Republic. Azua: near Pueblo Viejo (18°24.8'N, 70°44.7'W).

##### Other specimens examined.

Neotropical. West Indies. JAMAICA. **St. Andrew:** Mavis Bank (1.7 km E; 18°02.4'N, 77°39.5'W; 575 m), Yallahs River, 21–22 Apr-1 May 2000, W. N. Mathis (4♂, 1♀; USNM).

##### Distribution

**Figure 166.**
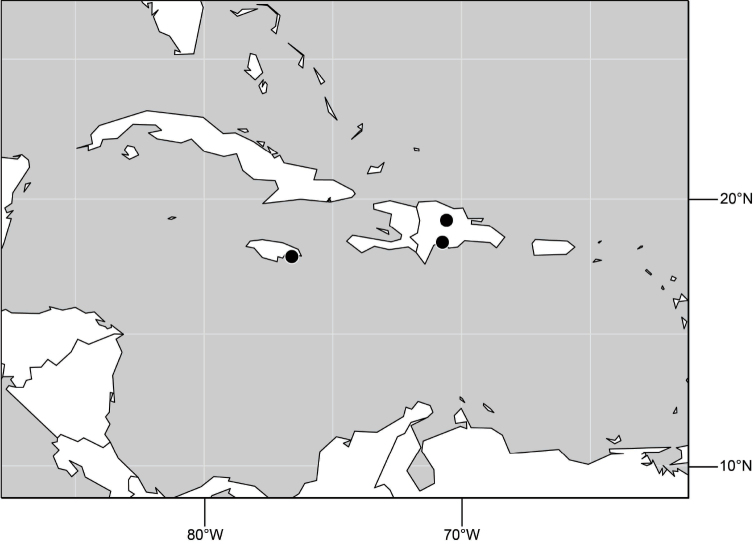
Distribution map of *Hydrochasma edmistoni* sp. n.

(Fig. 166). Neotropical: West Indies (Dominican Republic, Jamaica).

##### Etymology.

The species epithet, *edmistoni*, is a genitive Latin patronym to honor Father James F. Edmiston, O. F. M., a former colleague in the study of shore flies and a personal friend. Jim passed away on Sunday, May 18, 2008, and is still greatly missed.

##### Remarks.

Although similar to congeners of the *leucoproctum* group, this species is readily distinguished from them by the shape of the epandrium, especially the ventral epandrial process that is parallel-sided and has a uniquely shaped apical one-fourth. Subapically, the ventral epandrial process bears a broad and shallowly V-shaped row of tiny setulae. The hypandrium is V-shaped with a moderately well-developed anterior base and a U-shaped, posteromedial, hypandrial emargination. The posterior hypandrial arms are slightly divergent and delicately developed.

#### 30. Hydrochasma garvinorum

Mathis & Zatwarnicki

http://species-id.net/wiki/Hydrochasma_garvinorum

Figs 167 –171

Hydrochasma garvinorum Mathis & Zatwarnicki, 2010: 117 [United States. Virginia. *Rappahannock*: Hazel River (NW Culpeper; 38°33.8'N, 78°11.6'W, 171 m). HT ♂, USNM].

##### Diagnosis.

**Figures 167–170.**
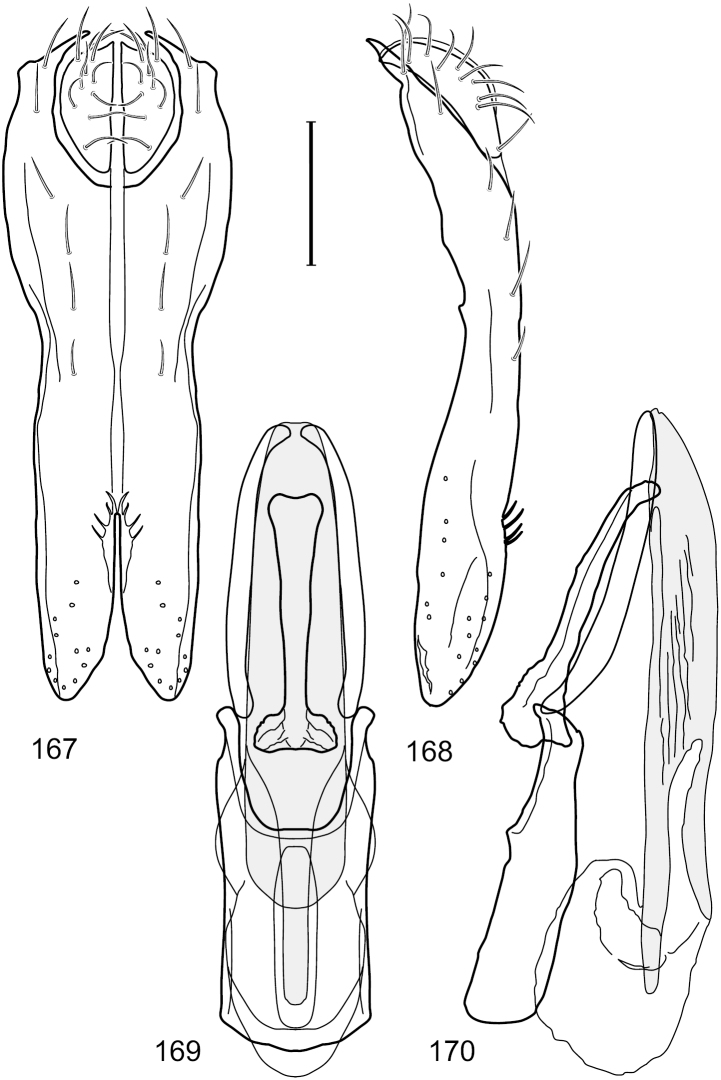
*Hydrochasma garvinorum* Mathis & Zatwarnicki (USA. Virginia. Stafford: Falmouth, Rappahannock River) **167** epandrium and cerci, posterior view **168** same, lateral view **169** internal structures of male terminalia (aedeagus [shaded], phallapodeme, gonite, hypandrium), ventral view **170** same, lateral view. Scale bar = 0.1 mm.

This species is distinguished from congeners by the following combination of characters: Small shore flies, body length 1.50–1.90 mm. *Head*: Antenna mostly dark gray, pedicel extensively yellow, basal flagellomere yellowish ventrobasally; parafacial silvery white, concolorous with facial coloration. Gena moderately high, height usually slightly higher than length of basal flagellomere; gena-to-eye ratio 0.13–0.15. *Thorax*: Wing hyaline; costal vein ratio 0.40–0.50; M vein ratio 0.50–0.52. Hindtibia bearing an apicoventral, black setula. *Abdomen*: Tergites lacking wedge-like marking laterally but tergites 2–4 with wide medial area extensively dark slate gray to black; tergite 5 slate colored, similar to coloration of medial area on tergites 1–4. Male terminalia (Figs 167–170): Combined structures generally elongate, in posterior view height nearly 3× width; epandrium with dorsal arch thinly tapered laterally, not connected medially above cerci, epandrium in posterior view (Fig. 167) with dorsal half somewhat quadrate, bearing some setulae medially, shallowly pedunculate near mid length, ventral portion generally lacking setulae, as 2, nearly parallel-sided, elongate processes, ventral margin V shaped, deeply incised medially, in lateral view (Fig. 168) as an elongate, shallowly curved structure, widest subapically, apex pointed; cerci short, height not quite twice width (Fig. 167), not attached lateroventrally with epandrium; aedeagus in lateral view (Fig. 170) elongate, over 5× longer than width, basal 3/4 tubular, thinly cigar-shaped, apical 1/4 membranous, expanded, in ventral view (Fig. 169) essentially parallel sided, apex pointed; phallapodeme in lateral view (Fig. 170) narrow, elongate, unevenly spatulate with widened keel end toward hypandrium, opposite end slightly tapered and curved, keel short and narrow, in ventral view (Fig. 169) narrowly T-shaped with arms short and right angled; gonite in lateral view (Fig. 170) narrow, elongate, bar-like, shallowly curved, in ventral view (Fig. 169) very shallowly curved; hypandrium in lateral view (Fig. 170) elongate, moderately shallow, anterior 2/3 parallel sided, narrowly rectangular, in ventral view (Fig. 169) rectangular with posterior margin deeply U-shaped, anterior margin broadly rounded to truncate.

##### Type material.

The holotype male is labeled “**USA. V[IRGINI]A.** Rappahannock: Hazel Riv[,](38°33.8'N, 78°11.6'W; 171m), 24 Jul 2008, Dianne & Wayne N. Mathis/USNM ENT 00117954 [plastic bar code label]/HOLOTYPE ♂ *Hydrochasma garvinorum* W. Mathis & T. Zatwarnicki USNM [red].” The holotype is double mounted (minuten in a block of plastic), is in excellent condition, and is deposited in the USNM. Twenty-eight paratypes (25♂, 3♀; USNM) bear the same label data as the holotype. Other paratypes are as follows:

VIRGINIA. **Chesterfield:** Pocahontas State Park (37°23.1'N, 77°32.4'W), 11 May 2002, D. and W. N. Mathis (5♂, 2♀; USNM). **Culpeper:** Lake Pelham (38°27.8'N, 78°02.7'W), 28 Apr 2006, D. and W. N. Mathis (1♀; USNM). **Fairfax:** Dead Run (mouth; 38°58'N, 77°10.4'W), 24 Apr 2006, D. and W. N. Mathis (1♂; USNM); Dead Run (swamp; 38°57.8'N, 77°10.3'W), 4 May 2006, D. and W. N. Mathis (5♂, 8♀; USNM); Great Falls (Patowmack Canal; 39°00.1'N, 77°15.2'W), 29 Aug 2007, D. and W. N. Mathis (1♂; USNM); Turkey Run (38°57.8'N, 77°09.4'W), 4 May 2006, D. and W. N. Mathis (3♀; USNM); Turkey Run (mouth; 38°57.9'N, 77°09.4'W), 20 Apr-23 Jul 2006, 2007, 2008, D. and W. N. Mathis, H. B. Williams, T. Zatwarnicki (13♂, 12♀; USNM). **Prince William:** Prince William Forest Park, South Quantico Creek (38°34'N, 77°22'W), 10 Jul–13 Aug, D. and W. N. Mathis (15♂, 5♀; USNM). **Rappahannock:** Hazel River (NW Culpeper; 38°33.8'N, 78°11.6'W, 171 m), 28 Jun 2008, W. N. Mathis, T. Zatwarnicki (10♂, 1♀; USNM). **Spotsylvania:** Rappahannock River (38°18.8'N, 77°32.5'W), 15 Apr 2006, D. and W. N. Mathis (2♂, 2♀; USNM). **Stafford:** Aquia Harbour (38°27.7'N, 77°23.3'W), 15 May-21 Jul 1988, 2000, D. and W. N. Mathis (13♂, 14♀; USNM); Aquia Harbour, Lions Park (38°27'N, 77°23.3'W), 10 Apr-30 May 2005, 2006, 2007, 2008, D. and W. N. Mathis (14♂, 10♀; USNM); Falmouth (38°19.2'N, 77°28.1'W; Rappahannock River; 9 m), 11 Apr-30 Jun 2007, 2008, D. and W. N. Mathis (14♂, 6♀; USNM). **Independent City:** Fredericksburg (Rappahannock River; 38°18.3'N, 77°27.5'W), 14 Apr 2006, D. and W. N. Mathis (1♂, 1♀; USNM); Fredericksburg (Alum Park; 38°17.4'N, 77°28.9'W), 30 Apr 2007, D. and W. N. Mathis (3♂, 5♀; USNM).

##### Type locality.

United States. Virginia. Rappahannock: Hazel River (NW Culpeper; 38°33.8'N, 78°11.6'W, 171 m).

##### Other specimens examined.

Nearctic. UNITED STATES. CALIFORNIA. **Sonoma:** Mesa Grande (38°28.4'N, 123°01.5'W), Jun 1908, J. P. Baumberger (1♀; ANSP).

DISTRICT OF COLUMBIA. Rock Creek, Boundary Bridge (38°59.2'N, 77°03.2'W), 18 May 2007, D. and W. N. Mathis (4♂, 6♀; USNM); Rock Creek, Milkhouse Ford (38°57.9'N, 77°02.9'W), 18 May 2007, D. and W. N. Mathis (1♂, 3♀; NPSRC, USNM).

GEORGIA. **Decatur:** Spring Creek (30°51.3'N, 84°35.1'W), 16–29 Jul 1912 (1♂; ANSP).

IDAHO. **Bonner:** Sandpoint (48°15.2'N, 116°37.5'W), 27 Aug 1918, A. L. Melander (1♀; ANSP).

MARYLAND. **Prince George’s:** College Park (38°58.8'N, 76°56.2'W), 5 Sep 1977, W. N. Mathis (1♀; USNM).

MASSACHUSETTS. **Worcester:** Athol (42°35'N, 72°13'W), 31 Jul 1953, A. L. Melander (1♀; ANSP).

MINNESOTA. **Houston:** La Crescent (43°49.2'N, 91°21.1'W), 16 Jun 1925, C. B. Phillip (1♀; ANSP).

NEW JERSEY. **Camden:** Ashland (39°51.8'N, 75°0.4'W), 13 May (1♀; ANSP).

OHIO. **Lorain:** Beaver Creek near Amherst (42°24'N, 82°14'W), 22 Aug 1977, B. A. Steinly (1♀; USNM).

PENNSYLVANIA. **Montgomery:** Lansdale (40°14.5'N, 75°17'W), 12 Jul 1908 (1♀; ANSP); Narberth (3.2 km N; 40°01'N, 75°15.6'W), 9 Sep 1915, E. T. Cresson, Jr. (1♀; ANSP).

SOUTH DAKOTA. **Bon Homme:** Springfield (42°51.2'N, 97°53.8'W), 26 Jun 1924 (1♀; ANSP).

TENNESSEE. **Blount:** Cades Cove (spring; 35°35.6'N, 83°50.5'W), 12 Jun 2008, D. and W. N. Mathis (1♂; USNM).

##### Distribution

**Figure 171.**
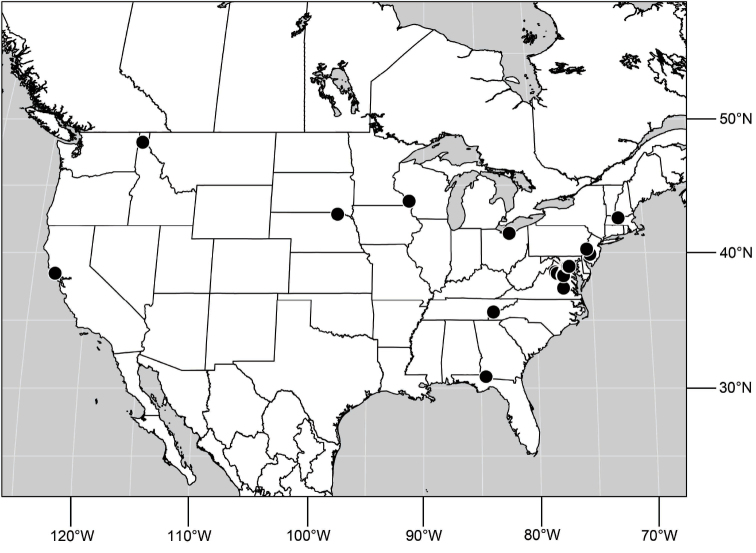
Distribution of *Hydrochasma garvinorum* Mathis and Zatwarnicki.

(Fig. 171). Nearctic: United States (California, District of Columbia, Georgia, Idaho, Maryland, Massachusetts, Minnesota, New Jersey, Ohio, Pennsylvania, South Dakota, Tennessee, Virginia).

##### Etymology.

The species epithet, *garvinorum*, is a pleural Latin genitive patronym to honor John Robert and Melodee Garvin (née Bodell), who guided us to the type locality on the Hazel River, which is located in the foothills of the Blue Ridge during the summer of 2008.

##### Remarks.

This species is similar to *Hydrochasma annae* but is distinguished from it by the more robustly developed ventral epandrial process in posterior and lateral views (Figs 167–168), and the rectangular shaped hypandrium that has a moderately deep, posteromedial hypandrial emargination.

#### 31. Hydrochasma leucoproctum

(Loew)

http://species-id.net/wiki/Hydrochasma_leucoproctum

Figs 172 – 179 , 185

Discocerina leucoprocta Loew, 1861: 355 [United States. “Maryland”; HT ♀, MCZ (11148)]. Aldrich 1905: 626 [Nearctic catalog]. Jones 1906: 178 [catalog]. Deonier 1964: 122 [key to species of Iowa]; 1965: 501 [habitats and abundance in Iowa].
Hydrochasma leucoproctum . Cresson 1942: 113 [generic combination, as *Hydrochasma leucostoma*]; 1946: 141 [list, Guatemala, Panama, Bolivia]. Wirth and Stone 1956: 468 [key, California]. Wirth 1965: 739 [Nearctic catalog]; 1968: 8 [Neotropical catalog]. Mathis and Zatwarnicki 1995: 183 [world catalog]. Mathis and Mathis 2008: 180–181 [shore flies of Plummers Island].

##### Diagnosis.

**Figures 172–174.**
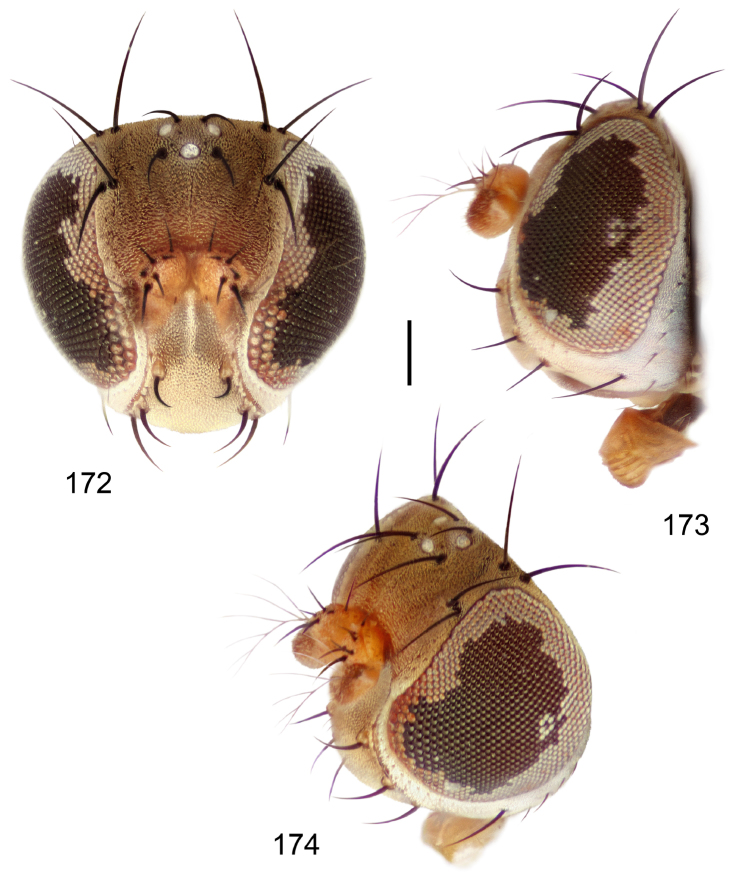
*Hydrochasma leucoproctum* (Loew) (St. Vincent. St. Patrick: Cumberland Bay) **172** head, anterior view **173** same, lateral view **174** same, oblique view. Scale bar = 0.1 mm.

**Figures 175–178.**
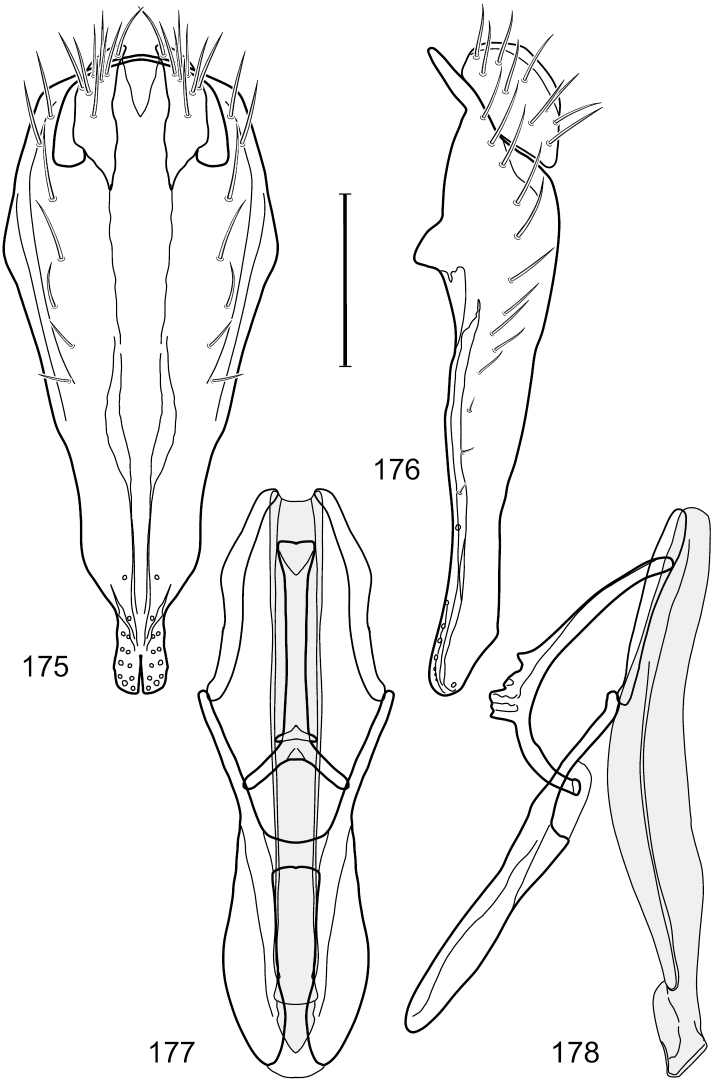
*Hydrochasma leucoproctum* (Loew) (St. Vincent. St. Patrick: Cumberland Bay) **175** epandrium and cerci, posterior view **176** same, lateral view **177** internal structures of male terminalia (aedeagus [shaded], phallapodeme, gonite, hypandrium), ventral view **178** same, lateral view. Scale bar = 0.1 mm.

This species is distinguished from congeners by the following combination of characters: Small to moderately small shore flies, body length 1.50–1.95 mm. *Head*: Antenna mostly yellowish; parafacial silvery white, contrasted with mostly yellowish face; face yellowish, extreme lateral margins brownish black; facial coloration at most only slightly distinct from parafacial (Figs 172–174). Gena moderately high, height usually slightly higher than length of basal flagellomere, gena-to-eye ratio 0.19–0.21. *Thorax*: Wing hyaline; with costal vein ratio 0.63–0.65; M vein ratio 0.46–0.50. Forecoxa mostly gray to blackish gray, at most only slightly yellowish; hindtibia lacking a ventral, spur-like seta near apex. *Abdomen*: Tergites 1–4 with dorsum entirely brownish black, with lateral margin sharply demarcated from silvery gray to gray portion, lacking grayish wedges laterally on tergites 2–4; tergite 5 light gray to silvery gray (Fig. 185), similar to coloration along lateral margins of preceding tergites but with posterior margin blackish brown to slate black, similar to coloration of medial area on tergites 1–4; medial coloration on tergites 1–4 wide, occupying most of dorsum, dark, grayish to slate black. Male terminalia (Figs 175–178): Combined structures generally elongate, in posterior view height more than twice width; epandrium with dorsal arch above cerci thinly developed, in posterior view (Fig. 175) as an inverted U on dorsal half, dorsal portion setulose, middle portion with lateral margins angled medially, generally lacking setulae, ventroapical portion with height about equal to that of cerci, parallel sided, partially bifurcate medially, broadly rounded apically, in lateral view (Fig. 176) with ventral 2/3 narrow, almost parallel sided with subapical, slightly narrowing, apical portion oriented anteriorly slightly, apex rounded; cerci short, height not quite twice width (Fig. 175), tenuously attached lateroventrally with epandrium; aedeagus in lateral view (Fig. 178) elongate, over 5× longer than width, tubular, slightly arched, with apical portion somewhat rectangular, in ventral view (Fig. 177) essentially parallel sided, apex pointed; phallapodeme in lateral view (Fig. 178) narrow, elongate, unevenly C-shaped with more curvature toward attachment with hypandrium, keel short and narrow, extended margin irregularly serrate, in ventral view (Fig. 177) narrowly Y-shaped with arms short and right angled; gonite in lateral view (Fig. 178) narrow, elongate, bar-like, in ventral view (Fig. 177) very shallowly angled at midlength; hypandrium in lateral view (Fig. 178) elongate, shallow, anterior 2/3 parallel sided, in ventral view (Fig. 177) with posterior half divergently U-shaped, anterior portion narrower, plate, rounded around anterior margin.

##### Type material.

The holotype female is labeled “[United States.] Md. [Maryland]/Loew Coll./leucoprocta m[ihi]. [handwritten]/Type 11148 [red; number handwritten]/Hydrochasma leucoproctum (Lw.) WWirth ‘61 [1961] [species name handwritten]/HOLOTYPE Discocerina leucoprocta Loew ♀ [pink; handwritten].” The holotype is double mounted (small pin in a rectangular block of pith), is in good condition (right basal flagellomere missing), and is deposited in the MCZ.

##### Type locality.

United States. “Maryland.”

##### Other specimens examined.

Nearctic. UNITED STATES. DELAWARE. **Kent:** Woodland Beach (39°19.9'N, 75°28.3'W; beach), 18 May 2006, D. and W. N. Mathis (3♂; USNM).

DISTRICT OF COLUMBIA. Chain Bridge (38°55.8'N, 77°6.9'W), 26 May-2 Sep 1916, 1922, W. L. McAtee (2♂, 1♀; USNM); Rock Creek, Boundary Bridge (38°59.2'N, 77°03.2'W), 18 May 2007, D. and W. N. Mathis (2♂; NPSRC, USNM); Washington (38°49.8'N, 77°00.6'W), D. W. Coquillett (1♂; USNM).

FLORIDA. **Marion:** Salt Springs (29°21.1'N, 81°44.1'W), 14 Apr 1989, D. and W. N. Mathis (7♂, 10♀; USNM); Silver Springs (29°13'N, 82°03.5'W), 2 Apr 1932, A. L. Melander (1♂; ANSP). **Pinellas:** St. Petersburg (27°44.3'N, 82°40.8'W), 19 Feb 1924, E. T. Cresson, Jr. (1♀; ANSP).

GEORGIA. **Tifton:** Tifton (31°27'N, 83°30.5'W), 6 Sep-Oct 1896 (8♀; ANSP).

ILLINOIS. **Adams:** Quincy (Mississippi River; 39°57.3'N, 91°25.1'W), 3 Sep 1995, J. F. Edmiston (3♂; USNM).

INDIANA. **Tippecanoe:** Lafayette (40°25'N, 86°52.5'W), 24 May-5 Sep 1916, E. W. Stafford (2♀; ANSP).

KANSAS. **Riley:** Manhattan (39°11'N, 96°34.3'W), 9 Jun 1934, C. W. Sabrosky (1♀; ANSP).

MARYLAND. **Anne Arundel:** Edgewater (6 km S; 38°53'N, 76°33'W; Smithsonian Environmental Research Center), 6 Jul 1976, J. H. Falk (8♂, 4♀; USNM). **Carroll:** Eldersburg (39°24.2'N, 76°57'W), 2 Jun 1985, J. E. Lowry, W. E. Steiner (1♂; USNM). **Garrett:** Savage River (39°30.1'N, 79°06.9'W; 400 m), 23 Aug 2006, D. and W. N. Mathis (1♂, 1♀; USNM); Savage River Reservoir (near Floyd; 39°30.7'N, 79°09.3'W; 420 m), 23 Aug 2006, D. and W. N. Mathis (4♂; USNM). **Montgomery:** Bethesda (38°58.8'N, 77°06'W), 24 May 1969, G. C. Steyskal (1♀; USNM); Plummers Island (38°58.2'N, 77°10.6'W), 30 May-4 Jul 1907, 1914, W. L. McAtee (2♀; USNM). **Prince George’s:** 4 Jul 1954, C. W. Sabrosky (1♀; USNM); Patuxent Wildlife Research Center (39°03'N, 76°48.2'W), 9 Jun 1967, W. W. Wirth (2♂, 3♀; USNM).

MICHIGAN. **Bay:** Bay City (43°35.7'N, 83°53.3'W), 16 Aug 1936, R. R. Dreisbach (2♂, 1♀; ANSP). **Kalamazoo:** Kalamazoo (42°17.5'N, 85°35'W), 10 May 1936, C. W. Sabrosky (1♀; ANSP). **Monroe:** Monroe (41°55'N, 83°23.9'W), 2 Jul 1939, G. C. Steyskal (1♂; ANSP).

NEW JERSEY. **Atlantic:** Absecon (39°24.9'N, 74°29.6'W), 26 Sep 2003, D. and W. N. Mathis (3♂; USNM). **Cape May:** Wildwood (38°59.5'N, 74°48.9'W), 18 Jul 1908 (1♂; ANSP). **Ocean:** Manahawkin (39°41.9'N, 74°15.5'W), 19 Aug 2004, D. and W. N. Mathis (1♀; USNM); Tuckerton (39°36.2'N, 74°20.8'W), 17-18 Jun-19 Aug 2004, W. & D. Mathis (8♂, 5♀; USNM); Tuckerton (16 km N; 39°53.3'N, 74°22.8'W), 26 Sep 2003, D. and W. N. Mathis (1♂; USNM).

NEW MEXICO. **Grant:** Bill Evans Lake (32°52.1'N, 108°34.5'W; 1416 m), 14 Aug 2007, D. and W. N. Mathis (7♂, 1♀; USNM).

OHIO. **Highland:** Rocky Ford State Park (39°10.9'N, 83°24.9'W), 17 Jul 1974, J. Regensberg (1♀; USNM). **Lawrence:** Vesuvius Lake (38°34.6'N, 82°37.5'W), 23 Aug 1974, J. Regensberg (1♀; USNM). **Morgan:** Burr Oak State Park (39°33.3'N, 82°02.8'W), 26 Aug 1974, J. Regensberg (1♂; USNM).

PENNSYLVANIA. **Allegheny:** Jacks Run (40°20.8'N, 79°47.5'W), 14 Jun 1908 (1♂; ANSP). **Delaware:** Swathmore (39°54.1'N, 75°21.1'W), 26 Jun-4 Jul 1908, 1909, 1910, E. T. Cresson, Jr. (26♂, 12♀; ANSP). **Montgomery:** Lansdale (40°14.5'N, 75°17'W), 12 Jul 1908 (2♀; ANSP); Narberth (3.2 km N; 40°0.8'N, 75°15.6'W), 9 Sep 1915, E. T. Cresson, Jr. (2♂, 2♀; ANSP).

TEXAS. **Augustine:** Rayburn Park (31°04'N, 94°05'W), 15 May 1993, D. and W. N. Mathis (1♂, 2♀; USNM). **Jasper:** Boykin Springs (31°05'N, 94°17'W), 15 May 1993, D. and W. N. Mathis (1♂; USNM).

VIRGINIA. **Accomack:** Assateague Island, near Refuge headquarters (37°54.5'N, 75°21.6'W), 19 Sep-3 Oct 2005, 2007, D. and W. N. Mathis (10♂; USNM). **Arlington:** 4-Mile Run (38°50.4'N, 77°02.7'W), 11 Sep 1977, W. N. Mathis (1♀; USNM). **Chesapeake:** Lake Drummond (36°36.2'N, 76°28.1'W), 29 June 1939, A. L. Melander (1♂; ANSP). **Chesterfield:** Pocahontas State Park (37°23.1'N, 77°32.4'W), 11 May 2002, D. and W. N. Mathis (6♂, 1♀; USNM). **Fairfax:** Dead Run (mouth; 38°58'N, 77°10.4'W), 24 Apr 2006, D. and W. N. Mathis (2♂; USNM); Dead Run (swamp; 38°57.8'N, 77°10.3'W), 4 May 2006, D. and W. N. Mathis (2♂, 1♀; USNM); Great Falls (Clay Pond; 39°00.1'N, 77°15.4'W), 12 May-17 Aug 2006, 2007, D. and W. N. Mathis, T. Zatwarnicki (7♂, 3♀; USNM); Great Falls (Patowmack Canal; 39°00.1'N, 77°15.2'W), 20 Jun-3 Oct 2006, 2007, D. and W. N. Mathis (14♂, 3♀; USNM); Great Falls (Potomac River; 39°0.2'N, 77°15.2'W), 13 Sep-4 Oct 2007, D. and W. N. Mathis, H. B. Williams (9♂, 1♀; USNM); Great Falls (quarry; 38°59.1'N, 77°14.8'W; 50 m), 13 Jun 2007, D. and W. N. Mathis (5♂, 1♀; USNM); Great Falls (swamp trail; 38°59.4'N, 77°15.2'W), 12 May 2006, D. and W. N. Mathis (1♂; USNM); Turkey Run (38°57.8'N, 77°09.4'W), 4 May-7 Sep 2006, D. and W. N. Mathis (2♂; USNM); Turkey Run (mouth; 38°57.9'N, 77°09.4'W), 22 May-17 Sep 2006, 2007, 2008, D. and W. N. Mathis, H. B. Williams, T. Zatwarnicki (43♂, 13♀; USNM). **Essex:** Tappahannock (37°55.8'N, 76°51.4'W; Rappahannock River), 18 Sep 2004, D. and W. N. Mathis (1♂, 1♀; USNM). **Henry:** Martinsville Reservoir (36°44.7'N, 79°52.2'W), 17 May 2005, D. and W. N. Mathis (4♂, 2♀; USNM); Martinsville, Smith River (36°39.9'N, 79°53'W; 225 m), 18 May 2005, D. and W. N. Mathis (2♂, 5♀; USNM). **Patrick:** Meadows of Dan (36°44.2'N, 80°22.9'W), 18 May 2005, D. and W. N. Mathis (1♂, 2♀; USNM); Woolwine (36°47.4'N, 80°16.7'W; 300 m), 17 May 2005, D. and W. N. Mathis (4♂; USNM). **Prince William:** Prince William Forest Park, South Quantico Creek (38°34'N, 77°22'W), 10 Jul-13 Aug 1993, D. and W. N. Mathis (4♂, 1♀; USNM). **Rappahannock:** Hazel River (NW Culpeper; 38°33.8'N, 78°11.6'W, 171 m), 28 Jun-24 Jul 2008, D. and W. N. Mathis and T. Zatwarnicki (6♂; USNM). **Roanoke:** Salem (Roanoke River; 37°16.1'N, 80°02.2'W; 300 m), 23 Sep 2007, D. and W. N. Mathis (8♂; USNM). **Spotsylvania:** Rappahannock River (38°18.8'N, 77°32.5'W), 3 Jul-10 Oct 2006, 2007, D. and W. N. Mathis (14♂, 1♀; USNM). **Stafford:** Aquia Creek (38°29.1'N, 77°23.8'W), 6 Jun 2005, D. and W. N. Mathis (5♂, 3♀; USNM); Aquia Harbour (38°27.7'N, 77°23.3'W), 15 May-21 Jul 2000, D. and W. N. Mathis (2♂, 2♀; USNM); Aquia Harbour, Aquia Creek (38°27.8'N, 77°23.1'W), 2 Sep 2006, D. and W. N. Mathis (1♂, 1♀; USNM); Aquia Harbour, Lions Park (38°27'N, 77°23.3'W), 9 May-16 Nov 2003, 2004, 2005, 2006, 2007, 2008, D. and W. N. Mathis (166♂, 17♀; USNM); Aquia Harbour (3 km N Stafford; 38°27'N, 77°23.3'W), 8 Jun-6 Oct 1988, 2003, D. and W. N. Mathis (27♂, 9♀; USNM); Aquia Landing (38°23.2'N, 77°19'W), 5 Nov 2005, D. and W. N. Mathis (1♂; USNM); Falmouth (38°19.2'N, 77°28.1'W; Rappahannock River; 9 m), 18 Apr-30 Jun 2007, 2008, D. and W. N. Mathis (14♂, 3♀; USNM). **Westmoreland:** Popes Creek (G. Washington birthplace; 38°13.7'N, 76°54.6'W), 16 Sep 1994, W. N. Mathis (1♂; USNM); Westmoreland State Park (bank of Potomac River; 38°09.7'N, 76°51.9'W), 9 Jun-25 Aug 1988, 2004, D. and W. N. Mathis (12♂, 6♀; USNM). **Independent City:** Virginia Beach (Little Island; 36°41.6'N, 75°55.5'W), 18 Aug 2006, D. and W. N. Mathis (7♂; USNM).

WEST VIRGINIA. **Hardy:** Baker (39°02.5'N, 78°44.9'W; 405 m), 12 Jul 2007, D. and W. N. Mathis (1♂, 1♀; USNM); Trout Pond (38°57.4'N, 78°44.2'W; 595 m), 13 Jul 2007, D. and W. N. Mathis (2♂, 3♀; USNM). **Morgan:** Third Hill Mountain near Sleepy Creek (39°40.2'N, 78°05.1'W; 190 m), 30 Jul 1939, J. A. G. Rehn and J. W. H. Rehn (1♀; ANSP). **Summers:** Bluestone State Park (37°36.7'N, 80°56.1'W; 440 m), 26 Sep 2007, D. and W. N. Mathis (5♂; USNM); Hinton (37°41.8'N, 80°53'W; New River; 427 m), 26 Sep 2007, D. and W. N. Mathis (7♂, 1♀; USNM). **Wyoming:** R. D. Bailey Lake (37°35.7'N, 81°46.8'W; 324 m), 25 Sep 2007, D. and W. N. Mathis (9♂, 1♀; USNM).

Neotropical. BELIZE. **Stann Creek:** Dangriga (16°58'N, 88°13'W), 3-4 Apr 1993, W. N. Mathis (2♀; USNM).

BRAZIL. **Amazonsas:** Manaus, INPA (03°05.9'S, 59°59.1'W; 60 m), 4 May 2010, D. and W. N. Mathis (1♂, 2♀; INPA, USNM); Reserva Ducke (02°55.8'S, 59°58.5'W; 40 m), 5 May 2010, D. and W. N. Mathis (1♀; INPA, USNM). **Paraná:** Bocaiúva do Sul (25°16.6'S, 48°58.5'W; 770 m), 16 Feb Jan 2010, D. and W. N. Mathis (1♂; DZUP, USNM); Curitiba, Universidade Federal do Paraná, Reserva Biológica (25°26.9'S, 49°14'W; 915 m), 9-11 Dec 2009, D. and W. N. Mathis (1♂, 2♀; DZUP, USNM); Morretes (25°28'S, 48°59.1'W), 29 Aug 2000, D. and W. N. Mathis (1♂; USNM).

COLOMBIA. **Valle de Cauca:** Sevilla (27 km W; 04°14.3'N, 76°01'W), 7 Mar 1955, E. S. Ross, E. I. Schlinger (1♂; USNM).

COSTA RICA. **Alajuela:** Alajuela (10°01'N, 84°13'W; 945 m), 15 Sep 1909, P. P. Calvert (6♂, 1♀; ANSP). **Cartago:** La Suiza (09°51.5'N, 83°37.5'W), 28 Jun 2001, W. N. Mathis (12♂; USNM). **Limón:** Cahuita (09°44.2'N, 82°50.4'W; beach), 28 Jun 2001, A. Freidberg (1♀; USNM); Guandoca, Manzanillo, Desembocadura, Laguna Guandoca (09°36.8'N, 82°40.9'W), 19-25 May 2004, D. Briceño (2♂; INBio); Parque Nacional Cahuita, Sector Puerto Vargas (09°44'N, 82°49'W; 1 m), 27-28 Jun 2001, J. D. Giterrez (4♂, 2♀; INBio); Westfalia (4 km S; 09°54.5'N, 82°59'W; beach), 27 Jun 2001, D. and W. N. Mathis (1♂; USNM). **Puntarenas:** Jacó (5 km E; 09°34.7'N, 84°35.6'W), 10 Jun 2003, D. and W. N. Mathis (2♂, 1♀; USNM); Montezuma (2 km S; 09°38.7'N, 85°04.4'W), 20 Jun 2001, D. and W. N. Mathis (1♂; USNM); Playa Jacó (09°36.5'N, 84°37.4'W; beach), 13 Jun 2003, D. and W. N. Mathis (1♂; USNM); Rincón (5 km S; 08°42.1'N, 83°30.8'W; 95 m), 10-11 Aug 2001, D. and W.N. Mathis (2♂; USNM); San Pedrillo (08°37.2'N, 83°44.1'W), 12-14 Aug 2001, D. and W.N. Mathis (8♂, 1♀; USNM). **San José:** Río S. Paraíso (09°33.8'N, 84°07.4'W; 350-400 m), 15-17 Feb 2003, W. N. Mathis (1♂; USNM).

ECUADOR. **Orellana:** Río Tiputini (0°38.2'S, 76°8.9'W), 12-26 Aug 1999, W. N. Mathis, A. Baptista, M. Kotrba (2♂; USNM).

GUYANA. Kumu River and Falls (25 km SE Lethem in Kanuku Mountains; 03°15.9'N, 59°43.6'W), 4–30 Apr 1994, 1995, W. N. Mathis (3♂, 4♀; USNM). Moco-Moco (30 km E Lethem in Kanuku Mountains; 03°18.2'N, 59°39.0'W), 3-29 Apr 1994, 1995, W. N. Mathis (4♂, 4♀; USNM).

HONDURAS. **Cortés:** Omoa (16°47.8'N, 87°58.4'W), 26 Sep 1995, D. and W. N. Mathis (5♂, 1♀; USNM); Puerto Cortés/Omoa (15°49'N, 87°56.2'W), 26 Sep 1995, D. and W. N. Mathis (1♂; USNM); San Pedro Sula (8 km S; 15°25.7'N, 88°01.4'W), 25-26 Sep 1995, D. and W. N. Mathis (1♂, 1♀; USNM).

MEXICO. **Chiapas:** Finca Prusia (33 km S Jaltenango; 15°49'N, 92°42'W), 10-12 May 1985, W. N. Mathis (1♀; USNM); Jaltenango (21 km S; 15°54'N, 92°40'W), 16 May 1985, W. N. Mathis (1♀; USNM). **Michoacán:** Uruapan (19°25'N, 102°03'W; 1600-1700 m), Aug 1975, N. L. H. Krauss (1♂; USNM). **Morelos:** Cuernavaca (18°56'N, 99°13.9'W), Apr 1965, N. L. H. Krauss (1♀; USNM). **Veracruz-Llave:** Ciudad Alemán (18°10.1'N, 96°06.2'W), 3 May 1985, W. N. Mathis (2♂, 2♀; USNM).

PANAMA. **Panama:** Tabernilla (09°08.9'N, 79°48.7'W), 6 Feb 1911, A. Busch (1♂, 1♀; ANSP).

PERU. **Cuzco:** Paucartambo, Atalaya (Río Alto Madre de Dios; 12°53.1'S, 71°21.6'W; 600 m), 4 Sep 1988, W. N. Mathis (1♀; USNM). **Madre de Dios:** Río Manu, Erika (near Salvación; 12°50.7'S, 71°23.3'W; 550 m), 5–6 Sep 1988, W. N. Mathis (1♂; USNM).

VENEZUELA. **Aragua:** Ocumare de la Costa (2 km N; 10°28.5'N, 67°45.8'W), 31 Mar-2 Apr 1981, A. S. Menke, L. Hollenberg (1♂, 2♀; USNM). **Trujillo:** Sabana Grande (09°26.3'N, 70°46.2'W), 3 Jun 1976, A. S. Menke, D. Vincent (1♂; USNM).

West Indies. DOMINICAN REPUBLIC. **La Vega:** Jarabacoa (1-2 km S; 19°06.9'N, 70°37'W; 520 m), 8-21 May 1995, W. N. Mathis (2♀; USNM). **Puerto Plata:** Río Camu (14 km E Puerto Plata; 19°41.9'N, 70°37.4'W), 17 May 1995, W. N. Mathis (2♂; USNM).

GRENADA. **St. Andrew:** Balthazar (12°07.7'N, 61°39.3'W), 15-19 Sep 1996, 1997, W. N. Mathis (5♂, 2♀; USNM); Grand Étang (lake; 12°05.6'N, 61°41.7'W), 14 Sep 1997, W. N. Mathis (1♂; USNM). **St. George:** Beauséjour Bay (12°05.5'N, 61°44.9'W), 21 Sep 1996, W. N. Mathis (7♂, 4♀; USNM). **St. John:** Concord Valley (12°06.9'N, 61°43.9'W), 14 Sep 1996, W. N. Mathis (6♂, 1♀; USNM); Palmiste (12°08.7'N, 61°44.4'W), 21 Sep 1996, W. N. Mathis (2♂, 1♀; USNM).

PUERTO RICO. Vieques Island (18°08.8'N, 65°26.7'W), Feb 1899, A. Busck (1♀; misidentified paratype of *Discocerina incisa*; USNM).

ST. LUCIA. Castries (5 km S; 13°59'N, 60°00'W), 16 Jun 1991, D. and W. N. Mathis (2♂; USNM); Dauphin Boguis (1.6 km S Marquis; 14°01'N, 60°55'W), 17 Jun 1991, D. and W. N. Mathis (4♂; USNM); Micoud (13°49'N, 60°54'W), 15 Jun 1991, D. and W. N. Mathis (7♂, 2♀; USNM). *St. Patrick*: Poyntzfield (12°10.5'N, 61°37.4'W), 20 Sep 1996, W. N. Mathis (1♀; USNM).

ST. VINCENT. **Charlotte:** Montreal (13°12'N, 61°11'W), 26 Mar-9 Jun 1989, 1991, A. Freidberg, D. and W. N. Mathis (1♀; USNM); Peruvian Vale (13°10.7'N, 61°08.7'W), 6-8 Sep 1997, W. N. Mathis (1♂; USNM); South Rivers (13°14.6'N, 61°09.3'W), 8 Sep 1997, W. N. Mathis (4♂; USNM); Spring (13°11.1'N, 61°08.5'W), 6 Sep 1997, W. N. Mathis (8♂; USNM); Yambou River (13°09.8'N, 61°08.7'W), 8-10 Sep 1997, W. N. Mathis (15♂, 1♀; USNM). **St. Andrew:** Buccament Bay (near beach; 13°11'N, 61°16'W), 25-28 Mar-8 Jun 1989, 1991, D. and W. N. Mathis (1♂, 1♀; USNM); Camden Park (13°10.2'N, 61°14.7'W), 4 Sep 1997, W. N. Mathis (12♂, 2♀; USNM); Layou (13°12'N, 61°17'W), 8 Jun 1991, D. and W. N. Mathis (10♂, 8♀; USNM). **St. David:** Richmond Beach, 28 Mar 1989, W. N. Mathis (3♂, 3♀; USNM). **St. George:** Kingston, Botanical Garden (13°9.7'N, 61°13.7'W), 25-27 Mar 1989, W. N. Mathis (2♂, 2♀; USNM); Yambou Head, 27 Mar 1989, W. N. Mathis (3♂, 2♀; USNM). **St. Patrick:** Cumberland Bay (13°16'N, 61°16'W), 28 Mar-15 Sep 1989, 1991, 1997, A. Freidberg, D. and W. N. Mathis (27♂, 19♀; USNM); Cumberland River (3 km E Spring Village; 13°15'N, 61°14'W), 10 Jun 1991, D. and W. N. Mathis (20♂, 5♀; USNM); Hermitage (13°15'N, 61°12.9'W), 9 Sep 1997, W. N. Mathis (4♂; USNM); Palmiste Park (13°12.7'N, 61°14.9'W), 5 Sep 1997, W. N. Mathis (1♂; USNM); Wallilabou (beach; 13°15'N, 61°16'W), 27 Mar-8 Jun 1989, 1991, D. and W. N. Mathis (11♂, 8♀; USNM).

##### Distribution

**Figure 179.**
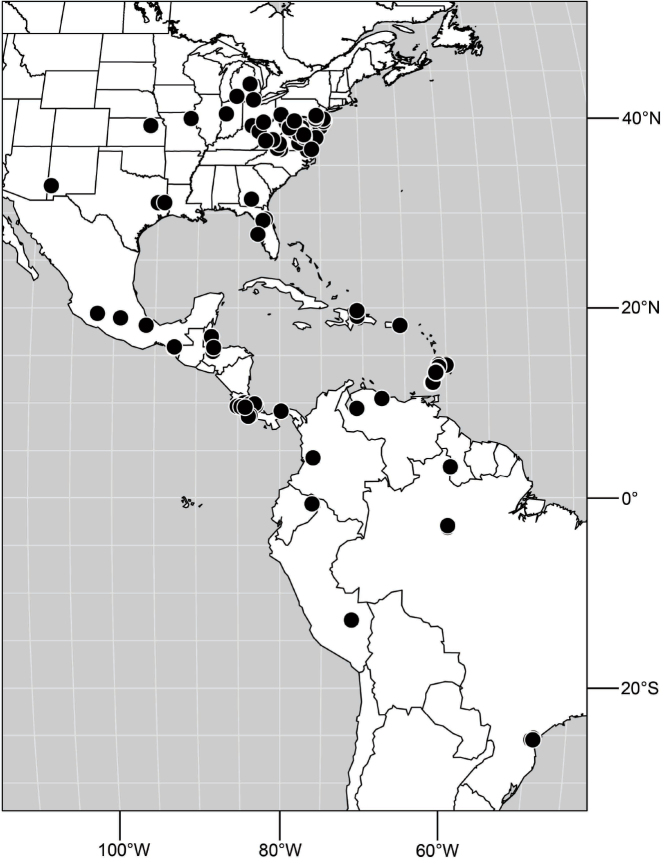
Distribution of *Hydrochasma leucoproctum* (Loew).

(Fig. 179). Nearctic: United States (Delaware, District of Columbia, Florida, Georgia, Illinois, Indiana, Kansas, Maryland, Michigan, New Jersey, New Mexico, Ohio, Pennsylvania, Texas, Virginia, West Virginia). Neotropical: Belize (Stann Creek), Brazil (Amazonas, Paraná), Colombia (Valle de Cauca), Costa Rica (Cartago, Limón, Puntarenas, San José), Ecuador (Orellana), Guyana, Honduras, Mexico (Chiapas, Michoacán, Morelos, Veracruz-Llave), Panama (Panama), Peru (Cuzco, Madre de Dios), Venezuela (Aragua, Trujillo), West Indies (Dominican Republic, Grenada, Puerto Rico, St. Lucia, St. Vincent).

##### Remarks.

This is one of the more common species of the genus in eastern North America and is found from Michigan and Pennsylvania south into northern South America. In the Delmarva States, this is likewise a widespread species, occurring along the coastal plain, Piedmont, Blue Ridge, and Alleghany zones.

This species is distinguished from others of the *leucoproctum* group by the uniquely shaped epandrium, especially the ventral epandrial process, which is generally tapered until the apical portion that is parallel sided. In addition the hypandrium is H-shaped, having both anterior and posterior emarginations.

#### 32. Hydrochasma lineatum sp. n.

http://zoobank.org/66E52FE3-E7C8-417B-A190-2259315E0E84

http://species-id.net/wiki/Hydrochasma_lineatum

Figs 100 , 180–183

##### Diagnosis.

**Figures 180–183.**
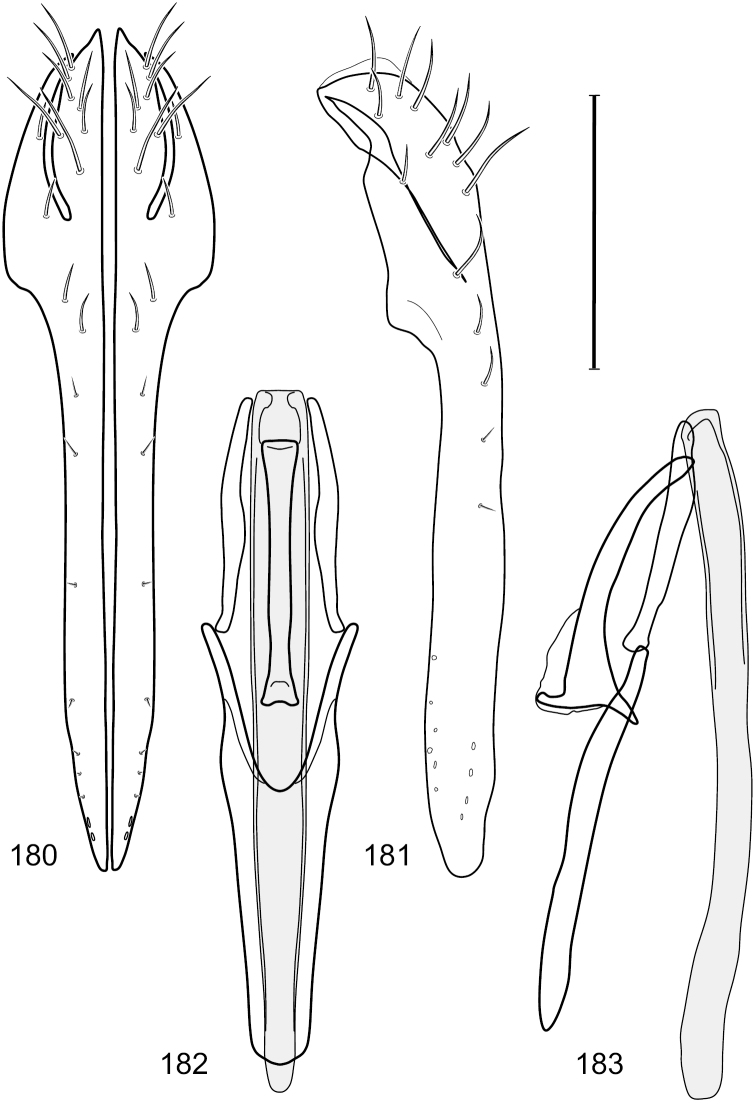
*Hydrochasma lineatum* sp. n. (Trinidad. St. George: Filette) **180** epandrium and cerci, posterior view **181** same, lateral view **182** internal structures of male terminalia (aedeagus [shaded], phallapodeme, gonite, hypandrium), ventral view **183** same, lateral view. Scale bar = 0.1 mm.

This species is distinguished from other congeners by the following combination of characters: Small shore flies, body length 1.20–1.70 mm. *Head*: Antenna mostly dark gray; parafacial silvery white, concolorous with facial coloration; gena-to-eye ratio 0.18–0.19. *Thorax*: Wing with costal vein ratio 0.70–0.72; M vein ratio 0.53–0.56. Forecoxa mostly blackish gray to gray. *Abdomen*: Tergites 1–4 extensively brownish black to blackish gray dorsally, lacking lateral wedges. Male terminalia (Fig. 180–183): Combined structures generally elongate, in posterior view height nearly 4× width, generally sparsely setulose, setulae especially sparse over midsection; epandrium with dorsal arch above cerci interrupted, not connected, in posterior view (Fig. 180) with basal third rectangular with angles rounded, apical 2/3 narrowed, linear, extended as abutting, narrow, almost parallel sided processes, apex tapered to a point, in lateral view (Fig. 181) generally elongate and narrow, ventral 2/3 essentially parallel sided, linear, apex rounded; cerci moderately short, height nearly twice width, semi-hemispherical (Fig. 180), fused ventrally to epandrium; aedeagus in lateral view (Fig. 183) generally very elongate and narrow, narrowly tubular, length over 10× width, mostly parallel sided, in ventral view (Fig. 182) elongate, narrow, tubular, parallel sided, apex rounded; phallapodeme in lateral view (Fig. 183) narrowly elongate, moderately and conspicuously expanded toward anterior margin, keel as a irregularly tapered extension, in ventral view (Fig. 182) clavate with gradually widened basal portion and anterior apex narrowly T-shaped; gonite in lateral view (Fig. 183) narrowly elongate, bar-like, very shallowly arched, in ventral view (Fig. 182) narrowly elongate, very shallowly sinuous; hypandrium in lateral view (Fig. 183) elongate, very shallow, nearly flat and parallel sided, in ventral view (Fig. 182) moderately deeply V-shaped, with anterior portion moderately robustly developed, tapered toward anterior, rounded apex, posterior margin deeply V-shaped.

**Figures 184–185.**
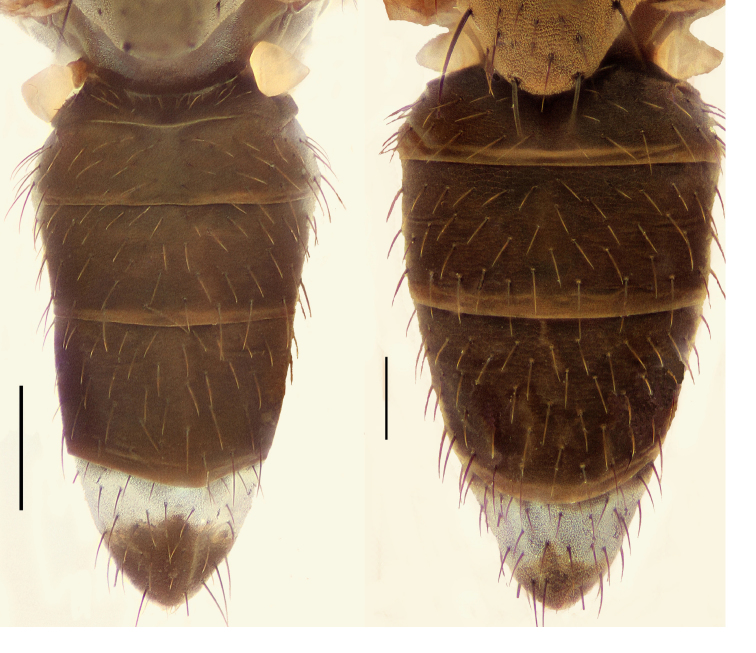
Abdomens of males, dorsal view. **184** *Hydrochasma capsum* sp. n. (Cuba. Sancti Spiritus: Topes de Collantes) **185** *Hydrochasma leucoproctum* (Loew) (St. Vincent: Cumberland Bay). Scale bar = 0.1 mm.

##### Type material.

The holotype male of *Hydrochasma lineatum* is labeled “**TRINIDAD.** St. Georg.: Filette (1 km SE, 10°47'N, 61°21'W), YarraRiver, 25 June 1993,Wayne N.Mathis/USNM ENT 00117969 [plastic bar code label]/HOLOTYPE ♂ *Hydrochasma lineatum* Mathis & Zatwarnicki, USNM [red].” The holotype is double mounted (minuten in a block of plastic), is in excellent condition, and is deposited in the USNM. Seven paratypes (4♂, 3; USNM) bear the same label data as the holotype.

##### Type locality.

Trinidad and Tobago. Trinidad. St. George: Filette (1 km SE; 10°47'N, 61°21'W).

##### Other specimens examined.

Neotropical. TRINIDAD and TABAGO. Tobago. **St. John:** Bloody Bay River (11°18'N, 60°38'W), 14 Jun 1993, W. N. Mathis (1♂; USNM); Charlotteville (5 km S; Hermitage River and beach; 11°18.9'N, 60°34.2'W), 11 Jun 1993, W. N. Mathis (2♀; USNM); Hermitage (11°18.9'N, 60°34.5'W), 22 Apr 1994, D. and W. N. Mathis (1♂; USNM). **St. Paul:** Argyle Falls (11°15'N, 60°35'W), 21 Apr 1994, W. N. Mathis (1♂; USNM).

##### Distribution

(Fig. 100). Neotropical: Trinidad and Tobago (Tobago, Trinidad).

##### Etymology.

The species epithet, *lineatum*, is of Latin derivation and mean linear, referring to the straight, line-like shape of the epandrium in posterior and lateral views.

##### Remarks.

This species is distinguished from congeners of the *leucoproctum* group by the very elongated ventral epandrial processes that are mostly parallel-sided, although tapered subapically to the apex. In addition, the hypandrium is uniquely shaped, being elongated, slender, and with an elongated anterior base that is almost twice the length of the depth of the posteromedial, hypandrial emargination.

#### 33. Hydrochasma robustum sp. n.

http://zoobank.org/78FEE3E7-1E8D-4C3B-A5F4-CA74867B498B

http://species-id.net/wiki/Hydrochasma_robustum

Figs 186 –190

##### Diagnosis.

**Figures 186–189.**
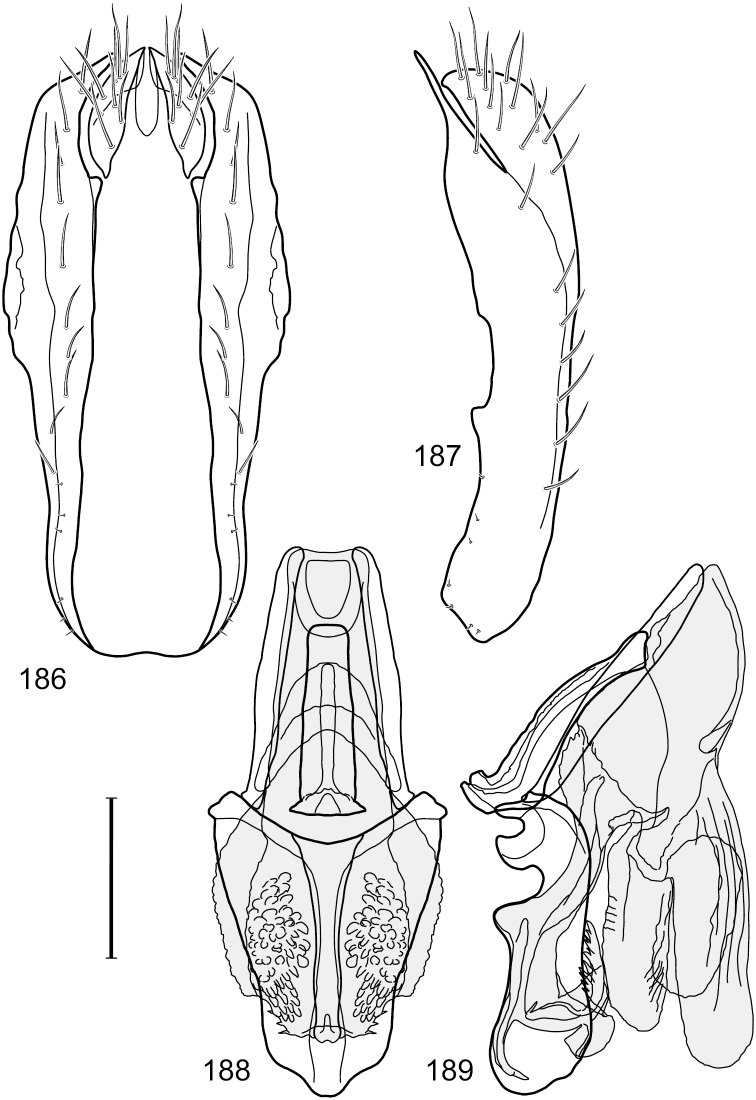
*Hydrochasma robustum* sp. n. (Brazil. Sao Paulo: Praia Puruba) **186** epandrium and cerci, posterior view **187** same, lateral view **188** internal structures of male terminalia (aedeagus [shaded], phallapodeme, gonite, hypandrium), ventral view **189** same, lateral view. Scale bar = 0.1 mm.

This species is distinguished from other congeners by the following combination of characters: Small to moderately small shore flies, body length 1.45–2.10 mm. *Head*: Antenna mostly dark gray; parafacial silvery white, concolorous with facial coloration; gena-to-eye ratio 0.18–0.19. *Thorax*: Wing with costal vein ratio 0.71–0.74; M vein ratio 0.59–0.61. Forecoxa mostly yellow, especially apically, basal portion partially silvery gray. *Abdomen*: Tergites 3–4 with gray wedges laterally, these sometimes deep and wide. Male terminalia (Figs 186–189): Combined structures generally moderately elongate, in posterior view (Fig. 186) more or less rectangular, height slightly more than twice width, generally setulose but with setulae on ventral 1/3 smaller than those on dorsal 2/3; epandrium with dorsal arch above cerci attenuated, not connected, in posterior view (Fig. 186) with wide medial area membranous, widest at midlength, sclerotized ventral portion tapered to sharp point, ventral margin almost truncate, in lateral view (Fig. 187) with posterior margin more or less evenly curved, anterior margin irregular with a shallow protuberance at ventral 1/3; cerci moderately long, height nearly twice width, narrowly semi-hemispherical (Fig. 187), almost attached lateroventrally with epandrium; aedeagus in lateral view (Fig. 189) complex, robust, moderately deeply trilobed, posterior 2 lobes digitiform, anterior lobe narrow, shallowly angulate, apex bilobed, in ventral view (Fig. 188) basal half relatively narrow, thereafter wider, apex narrowed, width subequal to basal width, delicately serrate laterally; phallapodeme in lateral view (Fig. 189) elongate, narrow, rod-like, mostly parallel sided, apex at hypandrial end, widened, in ventral view (Fig. 188) an elongate, robust, T-shaped process with width of crossbar only slightly greater than width of stem; gonite in lateral view (Fig. 189) narrow, elongate, bar-like, nearly straight, in ventral view (Fig. 188) bar-like, nearly straight; hypandrium in lateral view (Fig. 189) robust, irregularly shaped, with 2 basal notches, broadly rounded apically, in ventral view (Fig. 188) widest posteriorly, thereafter anteriorly gradually tapered, anterior margin obtusely narrowed, posterior margin shallowly but broadly emarginate.

##### Type material.

The holotype male of *Hydrochasma robustum* is labeled “**BRAZIL.** São Paulo: Praia Puruba (23°21'S, 44°55.6'W; beach),29Mar2010[,] D. & W. N. Mathis/USNM ENT 00117967 [plastic bar code label]/HOLOTYPE ♂ *Hydrochasma robustum* Mathis & Zatwarnicki, DZUP [red].” The holotype is double mounted (minuten in a block of plastic), is in excellent condition, and is deposited in the DZUP. Twenty paratypes (20♂; USNM) bear the same label data as the holotype.

##### Type locality.

Brazil. São Paulo. Ubatuba, Praia Puruba (23°21'S, 44°55.6'W; beach).

##### Other specimens examined.

Neotropical. ARGENTINA. **Tucumán:** La Cavera (Tafí Viejo; 26°45'S, 65°16'W), 23–28 Nov 1951, M. L. Aczel, R. Golbach (1♂; USNM).

BRAZIL. **Paraná:** Bocaiúva do Sul (25°16.6'S, 48°58.5'W; 770 m), 16 Feb 2010, D. and W. N. Mathis (1♂; USNM); Bocaiúva do Sul (ca. 10 km NW; 25°14.9'S, 49°08.9'W; 890 m), 2–4 Nov 2010, D. and W. N. Mathis (3♂, 5♀; DZUP, USNM); Curitiba, Universidade Federal do Paraná, Reserva Biológica (25°26.9'S, 49°14'W; 915 m), 14 Dec-8 Jan 2009, 2010, D. and W. N. Mathis (4♀; DZUP); Matinhos (N.; 25°46.4'S, 48°30.8'W; 1 m; beach/estuary), 30 Jan-9 Apr 2010, D. and W. N. Mathis (4♂, 1♀; DZUP, USNM); Matinhos (Rio da Onça; 25°47.4'S, 48°31.6'W; 3 m), 12 Nov 2010, D. and W. N. Mathis (8♂, 1♀; DZUP, USNM); Prainha (5 km S Matinhos; 25°51.2'S, 48°33.6'W; beach), 15 Nov 2010, D. and W. N. Mathis (2♂; DZUP, USNM). **São Paulo:** Estação Biológica de Boracéia (23°38.9'S, 45°52.8'W; 850 m), 27 Feb 1967, M. E. Irwin (3♂; USNM); Ubatuba, Praia do Estaleiro (23°20.5'S, 44°53'W; beach), 30 Mar 2010, D. and W. N. Mathis (1♂; DZUP, USNM). **Rio de Janeiro:** Ilha da Marambaia (23°03.6'S, 43°59.1'W), 4 Sep 2000, W. N. Mathis (1♂; USNM).

COSTA RICA. **Alajuela:** Volcán Tenorio, El Pilón (10°44.1'N, 84°59.5'W; 967 m), 9 May 2002, J. D. Gutierrez (1♂; INBio). **Guanacaste:** Bagaces Fortuna Z. P. Miravalles (10°43.1'N, 84°51.3'W; Sendero Cabro Muco; 980 m), 8–31 Jul 2002, J. D. Gutierrez (1♂; INBio); Parque Nacional Santa Rosa (Estación; Camino Cafetal; 10°51.5'N, 85°36.7'W; 300 m), 3–5 Aug 2002, D. Briceño (1♂, 1♀; INBio); Sector el Hacha, Finca el Oro, 5 km SW Hacienda Alemania (11°00'N, 85°33'W; 400 m), 14–19 Apr 2002, D. Briceño (2♂, 4♀; INBio).

ECUADOR. **Napo:** San Francisco de Borja (0°25.4'S, 77°50.5'W; 1610 m), 17 Jan 1978, W. N. Mathis (2♂, 13♀; USNM).

GUYANA. Menzies Landing (05°10.1'N, 59°29.5'W), 23 Aug 1997, W. N. Mathis (1♂; USNM).

West Indies. DOMINICAN REPUBLIC. **Barahona:** Cortico, La Mina (18°06.7'N, 71°13.4'W; 1300 m), 23 Mar 1999, W. N. Mathis (1♂; USNM); Paraíso (6 km NW; Río Nizao; 18°02'N, 71°12'W; 170 m), 21 Mar 1999, W. N. Mathis (1♂; USNM); San Rafael (18°01.9'N, 71°08.4'W), 25–26 Jul 1990, J. E. Rawlins, S. A. Thompson, C. Young (1♂; USNM).

##### Distribution

**Figure 190.**
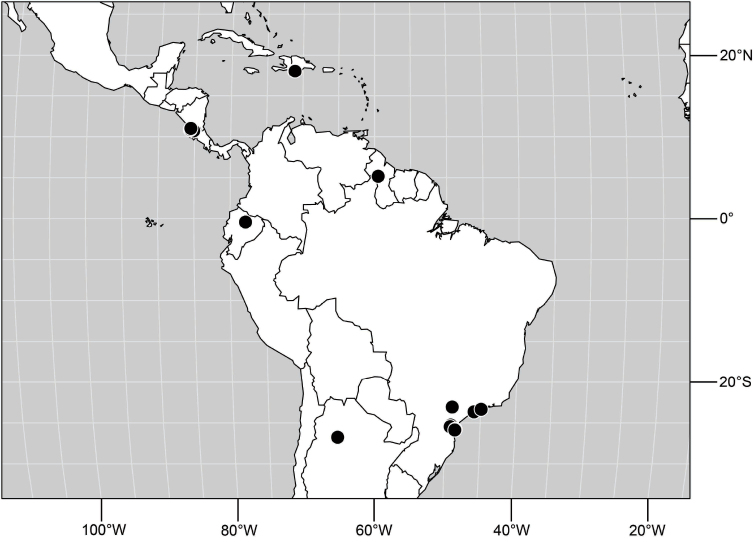
Distribution of *Hydrochasma robustum* sp. n.

(Fig. 190). Neotropical: Argentina (Tucumán), Brazil (Paraná, São Paulo, Rio de Janeiro), Costa Rica (Guanacaste), Ecuador (Napo), Guyana, West Indies (Dominican Republic).

##### Etymology.

The species epithet, *robustum*, is of Latin derivation and means robust, referring to the generally robust structures of the male terminalia.

##### Remarks.

This species is very similar and is evidently closely related to *Hydrochasma capsum*, as evidenced by the similar shapes of their respective epandriums in posterior view. This species can be distinguished from *Hydrochasma capsum* by the more elongated hypandrium in ventral view (longer than wide) and the unique, basal, hypandrial notches (best seen in lateral view; Fig. 189).

#### 34. Hydrochasma sagittarium sp. n.

http://zoobank.org/435DD268-B483-4536-A11A-1271A4349ABF

http://species-id.net/wiki/Hydrochasma_sagittarium

Figs 191 –195

##### Diagnosis.

**Figures 191–194.**
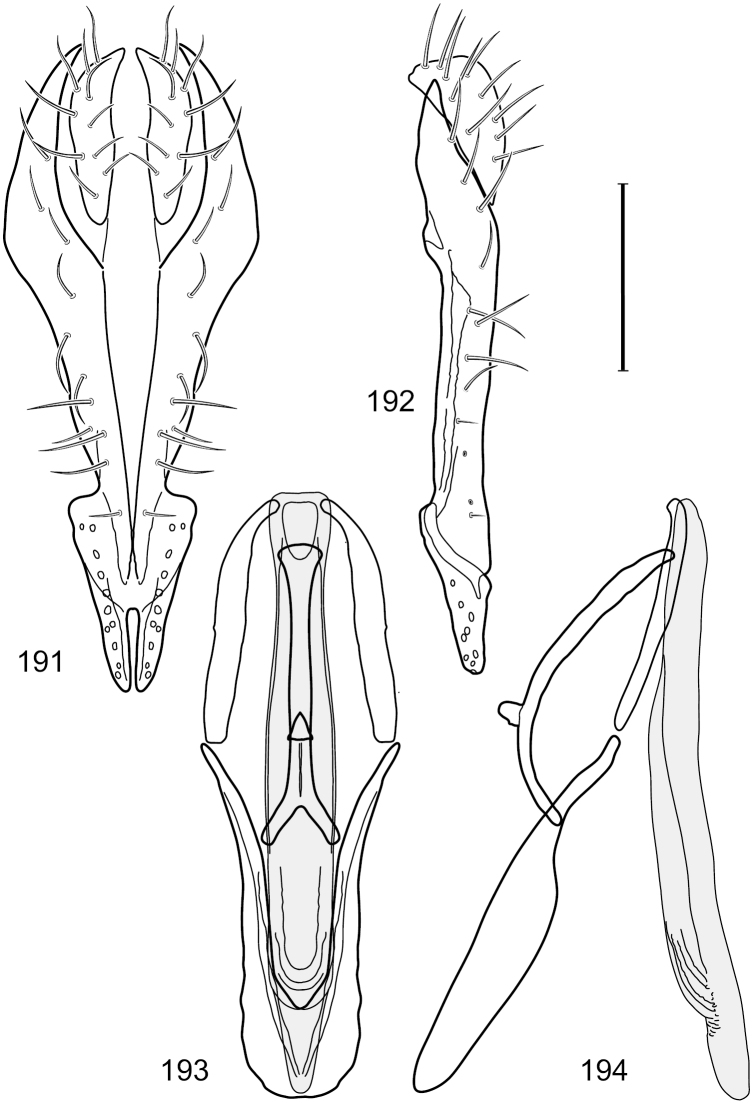
*Hydrochasma sagittarium* sp. n. (Brazil. Paraná: Morretes) **191** epandrium and cerci, posterior view **192** same, lateral view **193** internal structures of male terminalia (aedeagus [shaded], phallapodeme, gonite, hypandrium), ventral view **194** same, lateral view. Scale bar = 0.1 mm.

This species is distinguished from other congeners by the following combination of characters: Small shore flies, body length 1.35–1.75 mm. *Head*: Antenna mostly dark gray; parafacial silvery white, concolorous with facial coloration; gena comparatively short, gena-to-eye ratio 0.10–0.11. *Thorax*: Wing with costal vein ratio 0.67–0.70; M vein ratio 0.49–0.52. Forecoxa mostly yellowish orange; tergites 1–4 blackish gray dorsally. *Abdomen*: Tergites 1–4 broadly slate black on dorsum with sharply demarked gray lateral margin, without wedges, and gray ventral surface, tergite 5 of male gray with slate-black posterior margin. Male terminalia (Figs 191–194): Epandrium generally elongate and setulose, in posterior view (Fig. 191) with dorsal arch attenuate, not connected, dorsal 2/3–3/4 narrowly diamond shaped, widest just ventrad of cerci, thereafter ventrally tapered to arrow-shaped apex, arrow-shaped apex with length twice width, apex with narrowly incised medially, in lateral view (Fig. 192) narrowly elongate, mostly parallel sided, slightly wider at beginning of arrow-shaped apex, apex narrowly rounded; cerci elongate, length about 3× width, medial margin shallowly sinuous (Fig. 191), dorsal apex pointed, oriented medially, narrowly hemispherical, in lateral view (Fig. 192) more robust than posterior view, length slightly more than twice width; aedeagus in lateral view (Fig. 194) relatively simple, narrowly tubular, length 11× width, in ventral view (Fig. 193) also tubular; phallapodeme in lateral view (Fig. 194) linear, conspicuously curved, more so toward hypandrium, extended keel narrow and short, as a slight bump, in ventral view (Fig. 193) narrowly Y-shaped with arms short; gonite in lateral view (Fig. 194) narrowly elongate, bar-like, very shallowly curved, in ventral view (Fig. 193) more robustly developed, with end toward aedeagal base curved medially; hypandrium in lateral view (Fig. 194) with posterior 1/3 narrow, rod-like, anterior 2/3 with width nearly equal to that of aedeagus, in ventral view (Fig. 193) a deeply incised plate, anterior margin broadly rounded, posterior extensions curved slightly laterally.

##### Type material.

The holotype male of *Hydrochasma sagittarium* is labeled “*TOBAGO. St. John*: Parlatuvier (creek; 11°17.9'N, 60°35'W), 14 Jun 1993, W. N. Mathis/USNM ENT 00117966 [plastic bar code label]/HOLOTYPE ♂ *Hydrochasma sagittarium* Mathis & Zatwarnicki, USNM [red].” The holotype is double mounted (minuten in a block of plastic), is in excellent condition, and is deposited in the USNM. Nine paratypes (8♂, 1♀; USNM) bear the same label data as the holotype. Other paratypes are as follows: TRINIDAD and TOBAGO. **St. George:** Arima (8 km N; 10°41'N, 61°18'W), Verdant Vale, 19 Jun 1993, W. N. Mathis (8♂, 3♀; USNM). **St. John:** Charlotteville (2 km S; 11°19'N, 60°33'W), 10 Jun 1993, 1994, W. N. Mathis (2♂, 3♀; USNM) Speyside (1 km NW; Doctor River; 11°18'N, 60°32'W), 12–13 Jun 1993, W. N. Mathis (4♂, 5♀; USNM).

##### Type locality.

Trinidad and Tobago. Tobago: St. John: Parlatuvier (creek; 11°17.9'N, 60°35'W).

##### Other specimens examined.

Neotropical. BELIZE. **Stann Creek:** Sittee Point (16°48.6'N, 88°15.5'W), 28 Apr 1987, R. Faitoute, P. S. Spangler (1♂, 3♀; USNM); Twin Cays, West Bay (16°50'N, 88°06'W), 22 May 1988, W. N. Mathis (1♀; USNM); Wee Wee Cay (16°45.9'N, 88°08.6'W), 6–9 Nov 1987, D. and W. N. Mathis (3♀; USNM).

BRAZIL. **Amazonas:** Manaus, INPA (03°05.9'S, 59°59.1'W; 60 m), 4 May 2010, D. and W. N. Mathis (1♂, 2♀; INPA, USNM); Reserva Ducke (02°55.8'S, 59°58.5'W; 40 m), 5 May 2010, D. and W. N. Mathis (1♀; INPA, USNM). **Paraná:** Antonina (25°27.1'S, 48°41.1'W; beach; Ponta da Pita), 15 Feb 2010, D. and W. N. Mathis (1♂; USNM); Bocaiúva do Sul (25°16.6'S, 48°58.5'W; 770 m), 16 Feb 2010, D. and W. N. Mathis (1♂; DZUP, USNM); Curitiba, Universidade Federal do Paraná, Reserva Biológica (25°26.9'S, 49°14'W; 915 m), 9–11 Dec 2009, D. and W. N. Mathis (1♂, 2♀; DZUP, USNM); Prainha (5 km S Matinhos; 25°51.2'S, 48°33.6'W; beach), 15 Nov 2010, D. and W. N. Mathis (2♂; DZUP, USNM).

COSTA RICA. **Limón:** Parque Nacional Barbilla, Sector Casas Negras, Orilla Río Dantas (09°59.8'N, 83°26.2'W; 300 m), 15 Dec 2002, E. Rojas (1♂; INBio).

ECUADOR. **Napo:** San Francisco de Borja (0°25.4'S, 77°50.5'W; 1610 m), 17 Jan 1978, W. N. Mathis (1♂; USNM). **Pastaza:** Río Puyo (01°40'S, 77°55'W), R. Levi-Castillo (9♂, 11♀; USNM).

PERU. **Cuzco:** Paucartambo, Atalaya (Río Alto Madre de Dios; 12°53.1'S, 71°21.6'W; 600 m), 4 Sep 1988, W. N. Mathis (3♂, 14♀; USNM).

##### Distribution

**Figure 195.**
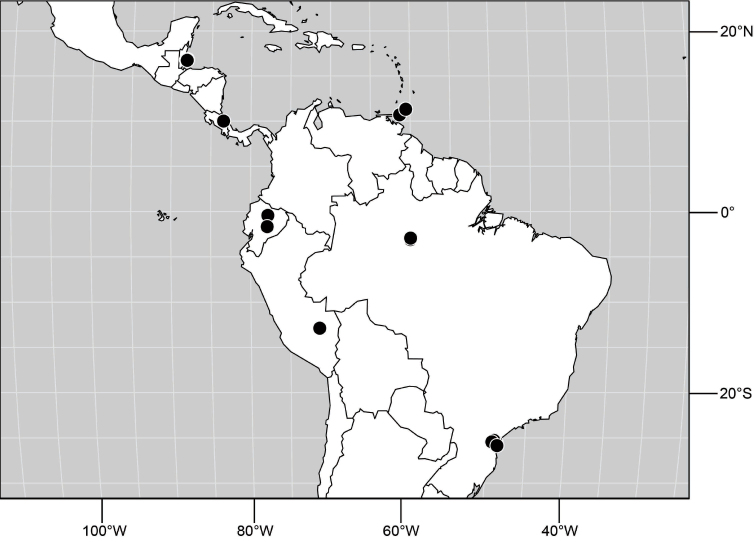
Distribution of *Hydrochasma sagittarium* sp. n.

(Fig. 195). Neotropical: Belize (Stann Creek), Brazil (Amazonas, Paraná), Ecuador (Napo, Pastaza), Peru (Cuzco), Trinidad and Tobago.

##### Etymology.

The species epithet, *sagittarium*, is of Latin derivation and means arrow, referring to arrow-like apex of the epandrium.

##### Remarks.

Although similar to several species in the *leucoproctum* group, this species is distinguished from all others by the distinctly arrowhead-shaped ventral portion of the ventral epandrial process and the deeply V-shaped hypandrium with delicately developed arms. In addition the arched and thinly develop phallapodeme that has a small projection near its midlength is diagnostic.

### Species excluded from *Hydrochasma*

Zatwarnicki and Mathis (2001) published a generic classification for the tribe Discocerinini Cresson and also included a revised generic characterization for the genus *Discocerina* Macquart. In the same paper and as a result of the revised classification, they also dealt with several taxonomic changes, such as transferring *Hecamedoides buccata* Cresson (1930), which had previously been placed in *Hydrochasma* (Cresson 1942), to the genus *Discocerina*. The basis for this transfer was discovery that the phallapodeme is fused with the base of the aedeagus. This fusion is apparently a synapomorphy for these species. More recently and while revising congeners of *Hydrochasma*, we studied the holotype of *Hydrochasma ceraceps* Cresson and discovered that this species too has fusion of the phallapodeme with the base of the aedeagus. Externally, both of these species have some characters of the genus *Hydrochasma*, most notably the high gena. Herein, we revise both of these species, providing descriptions and illustrations of the male terminalia that detail the basis for including both species in the genus *Discocerina*. Structures of the male terminalia, especially fusion of the phallapodeme with the base of the aedeagus, are the primary basis for this their inclusion in *Discocerina*.

#### Discocerina buccata

(Cresson)

http://species-id.net/wiki/Discocerina_buccata

Fig. 196 –199

Hecamedoides buccata Cresson, 1930: 78.
Hydrochasma buccatum . Cresson 1942: 113 [generic combination]. Wirth 1965: 738 [Nearctic catalog]. Mathis and Zatwarnicki 1995: 182 [world catalog].
Discocerina buccata . Zatwarnicki and Mathis 2001: 22 [generic combination].

##### Diagnosis.

**Figures 196–198.**
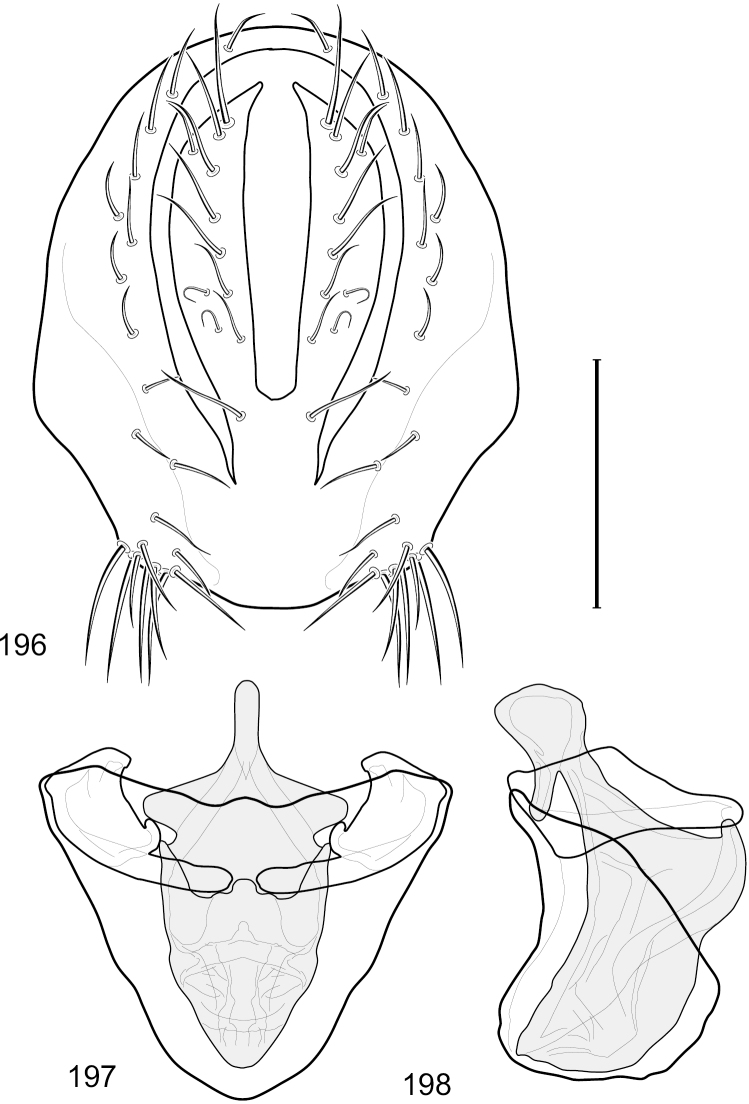
*Discocerina buccata* (Cresson) (USA. Minnesota: Bigstone) **196** epandrium and cerci, posterior view **197** internal structures of male terminalia (aedeagus [shaded], phallapodeme, gonite, hypandrium), ventral view **198** same, lateral view. Scale bar = 0.1 mm.

This species is distinguished from congeners by the following combination of characters: Generally densely microtomentose, whitish gray to blackish gray. Moderately small shore flies, body length 2.55–2.90 mm. *Head*: Frons moderately to densely microtomentose, whitish gray; anterior portion with some reddish orange coloration. Antenna largely black; basal flagellomere more brownish black with basal portion faintly yellowish orange; arista bearing 4–5 dorsal rays. Dorsal facial seta arising from bare spot at ventral margin of antennal groove. Gena high, gena-to-eye ratio 0.33 or larger, height subequal to combined length of pedicel and basal flagellomere. Maxillary palpus yellow. *Thorax*: Mesonotum whitish gray. Forefemur lacking anteroventral row of closely set setulae. *Abdomen*: Dorsum unicolorous, gray, slightly darker and less microtomentose than mesonotum; sternite 5 of male about 3× length of anterior width, gradually becoming wider posteriorly, posterior margin deeply emarginate, bearing numerous, short, setulae medially. Male terminalia (Figs 196–198): Epandrium short, length of portion ventrad of cercal cavity less than cercal height, width in posterior view (Fig. 196) greatest at level of ventral margin of cerci, thereafter turned medially toward ventral margin, ventral margin whole, not bifurcate, moderately wide, width slightly Greater than that of cercal cavity, slightly more produced medioventrally but with a shallow lateral swelling bearing patch of 5–6, moderately long setulae; cerci elongate, narrowly semi-hemispherical, fused ventrally.

##### Type material.

The holotype male of *Hecamedoides buccata* Cresson is labeled “Wildwood N[ew]J[ersey. Cape May Co.] VII, 18, 1908 [18 Jul 1908]/♂/TYPE Hecamedoides BUCCATA E.T.Cresson,Jr. 6367 [red; species and generic names and type number handwritten].” The holotype is double mounted (minuten in a rectangular card), is in excellent condition, and is deposited in the ANSP (6367).

##### Type locality.

NEW JERSEY. Cape May: Wildwood (38°59.5'N, 74°48.9'W).

##### Other specimens examined.

Nearctic. CANADA. NEW BRUNSWICK. Shippigan (47°44.5'N, 64°42.9'W), 14 Jul 1931, J. M. Aldrich (1♂; ANSP).

ONTARIO. Wainfleet (42°55.3'N, 79°22.5'W), 26 Jul 1921, M. C. Van Duzee (1♀; USNM).

QUEBEC. Trois Rivieres (46°21.3'N, 72°34.9'W), 6 Aug 1930, A. L. Melander (1♂; ANSP).

UNITED STATES. ARIZONA. **Apache:** St. Johns (34°30.4'N, 109°20.7'W), 12 Jun 1950 (1♀; USNM). **Mohave:** Adamana Road (34°55.1'N, 114°08.3'W), 12 Jun 1950 (3♂, 4♀; USNM); Littlefield (Virgin River; 36°53.2'N, 113°55.8'W), 7 Apr 1968, W. N. Mathis (1♂; USNM). **Navajo:** Joseph City (34°57.4'N, 110°19.9'W), 12 Jun 1950 (1♂; USNM); Kayenta (36°44.4'N, 110°14.4'W), 14 Apr 2003, W. N. Mathis, T. Zatwarnicki (3♂, 1♀; USNM).

FLORIDA. **Volucia:** Daytona Beach (29°12.6'N, 81°01.4'W), 23 Jan-5 Feb 1936, 1939, A. L. Melander (4♂, 1♀; ANSP, USNM).

KANSAS. **Riley:** Manhattan (39°11'N, 96°34.3'W), 9 Jun 1934, C.W. Sabrosky (1♀; USNM). **Wallace:** Wallace (38°54.7'N, 101°35.5'W), Jul 1885 (1♂; ANSP).

MARYLAND. **Calvert:** Chesapeake Beach (38°41.2'N, 76°32.1'W), 23 Jun-27 Jul 1913, 1931, C. P. Heinrich, F. K. Knab (3♂, 2♀; ANSP, USNM). **St. Mary’s:** Saint George Island (38°07.2'N, 76°29'W; Seaside), 23 Jun 1931, A. L. Melander (1♂; USNM).

MASSACHUSETTS. **Barnstable:** Falmouth (41°33.1'N, 70°36.9'W), 9 Jun-1 Jul 1930, A. L. Melander, A.H. Sturtevant (2♀; USNM); South Yarmouth (41°40'N, 70°11.1'W), 1 Aug 1939, A. L. Melander (1♂, 1♀; USNM).

MICHIGAN. **Bay:** Bay City (43°35.7'N, 83°53.3'W), 30 May 1934, G.C. Steyskal (1♂; ANSP). **Clare:** Clare (43°49.2'N, 84°46.1'W), 12 Jul 1941, R.R. Dreisbach (1♀; USNM). **Hillsdale:** Somerset (42°02.9'N, 84°22.6'W), 12 May 1952, A.H. Sturtevant (1♂; USNM). **Wayne:** Grosse Ile (42°07.7'N, 83°08.7'W), 8 Aug 1954, G.C. Steyskal (5♂, 8♀; USNM).

MINNESOTA. **Bigstone:** (45°22.4'N, 96°31'W), 5 Jun 1938, C. E. Mickel (1♀; USNM).

MONTANA. **Sheridan:** Medicine Lake (48°30.2'N, 104°30'W), 9 Jun 1969, W. W. Wirth (1♀; USNM).

NEBRASKA. **Cherry:** Snake River (42°33.4'N, 101°53.6'W), 2 Jun 1969, W. W. Wirth (3♀; USNM). **Lancaster:** Lincoln (40°48.4'N, 96°40.9'W), 19 Jun 1969, W. W. Wirth (1♀; USNM).

NEVADA. **Clark:** Bunkerville (36°46.4'N, 104°07.7'W), 20 May 1952, A. H. Sturtevant (1♀; USNM).

NEW HAMPSHIRE. **Coos:** Pickham Notch (44°15.4'N, 71°15.2'W), 9 Jul 1931, A. L. Melander (1♀; USNM).

NEW JERSEY. **Cape May:** Wildwood (38°59.5'N, 74°48.9'W), 18 Jul 1908 (3♂, 2♀; ANSP).

NEW MEXICO. **Curry:** Melrose (34°25.8'N, 103°38'W), 22 May 1954, A. H. Sturtevant (1♂; USNM). **Colfax:** Cimarron (36°30.4'N, 104°55.2'W; river margin), 26 May 1969, W. W. Wirth (1♀; USNM). **Taos:** Rio Grande (34°16'N, 106°46.2'W), 6 Jul 1953, W. W. Wirth (2♀; USNM). **Socorro:** Socorro (34°03.5'N, 106°53.5'W), S. D. Williston, 1916 (1♂; USNM).

NEW YORK. **St. Lawrence:** Coles Creek State Park (44°53.5'N, 75°08.5'W), 10 Aug 1976, S. R. Alm (1♂, 1♀; USNM).

NORTH CAROLINA. **Dare:** Nags Head (35°57.5'N, 75°37.5'W), 15 Jul 1959, W. W. Wirth (2♀; USNM).

NORTH DAKOTA. **Bowman:** Bowman-Haley Dam and Reservoir (45°59.5'N, 103°15.4'W; 840 m), 19 Jun 2008, D. and W. N. Mathis (16♂, 2♀; USNM).

OHIO. **Mercer:** Grand Lake, St. Marys (near Montezuma) (40°30.3'N, 84°32.4'W), 26 May 1977, B. A. Steinly (1♂; USNM).

OKLAHOMA. **Alfalfa:** Great Salt Plains (36°43.2'N, 98°10.7'W; salt marsh), 22 May 1969 (1♂; USNM).

PENNSYLVANIA. **Erie:** Presque Isle State Park (40°08.6'N, 80°08.1'W), 6 May 1977, B. A. Steinly (1♂; USNM).

RHODE ISLAND. **Washington:** Watch Hill (41°18.8'N, 71°51'W), 5 Aug 1939, A. L. Melander (1♀; USNM).

SOUTH DAKOTA. **Lawrence:** Beaver Creek (43°02.5'N, 100°09.5'W), 15 Jun 1969, W. W. Wirth (1♀; USNM). **Mellette:** Little White River (43°13'N, 101°0.9'W; river margin), 4 Jun 1969, W. W. Wirth (8♂, 6♀; USNM).

UTAH. **Emery:** Green River (3.3 km N; 39°01.7'N, 110°09.7'W; 1253 m), 19 Jul-5 Aug 1988, 1992, W. N. Mathis (7♂, 2♀; USNM). **Garfield:** Alvey Wash (8.4 km S Escalante; 37°42.3'N, 111°37.6'W; 1880 m), 21 May 2001, D. and W. N. Mathis (1♂, 1♀; USNM); Deer Creek (37°51.2'N, 111°21.1'W; 1762 m), 21 May 2001, D. and W. N. Mathis (2♂, 1♀; USNM); Willow Tank-Hurricane Wash (37°23.2'N, 111°08'W; stock tank), 22 May 2001, D. and W. N. Mathis (4♂, 1♀; USNM). **Grand:** Crystal Geyser (38 56.3'N, 110 8.1'W; 1000 m), 15 Aug. 2008, D. & W. Mathis (1♂; USNM); Green River (15.3 km N; 39°7'N, 110°6.6'W; 1255 m), 30 Jul-15 Aug 2007, 2008, D. and W. Mathis (10♂, 7♀; USNM); Thompson Springs (9 km N, 39°2.3'N, 109°43.4'W; 1740 m) 1 Aug 2007, D. and W. Mathis (20♂, 8♀; USNM). **Kane:** Drip Tank Canyon (37°19.4'N, 111°31.8'W), 15 May 2001, D. and W. N. Mathis (4♂, 1♀; USNM); East Fork of Virgin River (26.6 km N Knab; 37°12.2'N, 112°41.4'W), 18 May 2001, D. and W. N. Mathis (3♂, 1♀; USNM); Kanab Canyon (6.5 km N Kanab; 37°08.7'N, 112°32.4'W), 14 May 2001, D. and W. N. Mathis (3♂, 2♀; USNM); Seaman Wash (24.5 km E Kanab; 37°07'N, 112°15'W), 14 May 2001, D. and W. N. Mathis (1♂; USNM); Sheep Creek (37°29.7'N, 112°04'W), 17 May 2001, D. and W. N. Mathis (4♂, 1♀; USNM); Sheep Creek, junction Skutumpah Road (37°29.7'N, 112°03.9'W; 1800 m), 12 May 2000, K. T. Huntzinger, W. N. Mendel, C. R. Nelson (1♂, 1♀; USNM); Willis Creek (37°29'N, 112°05.8'W), 17 May 2001, D. and W. N. Mathis (3♂, 1♀; USNM). **San Juan:** Blanding, Recapture Reservoir (37°39.9'N, 109°26.5'W; 1840 m), 23 Jul 2013, D. and W. N. Mathis (3♂, 1♀; USNM).

VERMONT. **Chittenden:** Burlington (44°28.9'N, 73°13.5'W), 1 Sep 1937, A. L. Melander (1♀; ANSP).

VIRGINIA. **Accomack:** Assateague Island, Toms Cove (37°53.3'N, 75°20.6'W), 4 Oct 2005, D. and W. N. Mathis (16♂, 8♀; USNM); Assateague Island, Toms Cove (37°53.1'N, 75°20.7'W), 15 Jun 2007, D. and W. N. Mathis (1♀; USNM). **Westmoreland:** Popes Creek (G. Washington birthplace; 38°13.7'N, 76°54.6'W), 16 Sep 1994, W. N. Mathis (11♂, 4♀; USNM). **Independent City:** Virginia Beach City, Cape Henry (36°55.9'N, 76°01.2'W), 28 Jun 1939, A. L. Melander (1♂; USNM).

WYOMING. **Crook:** Alva (near; 44°38.6'N, 104°22.8'W; 1330 m), 20 Jun 2008, D. and W. N. Mathis (♂, ♀; USNM). **Niobrara:** Lusk (64.5 km N; 43°21'N, 104°13'W), Jul 1905 (1♂; ANSP).

##### Distribution

**Figure 199.**
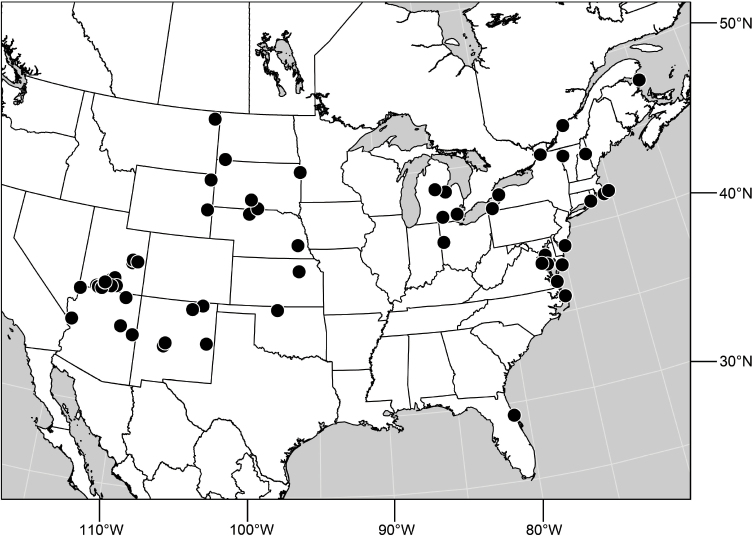
Distribution of *Discocerina buccata* (Cresson).

(Fig. 199). Nearctic: Canada (New Brunswick, Ontario, Quebec). United States (Arizona, Florida, Kansas, Maryland, Massachusetts, Michigan, Minnesota, Montana, Nebraska, Nevada, New Hampshire, New Jersey, New Mexico, New York, North Carolina, North Dakota, Ohio, Oklahoma, Pennsylvania, Rhode Island, South Dakota, Utah, Vermont, Virginia, Wyoming).

##### Remarks.

This species was included in the genus *Hydrochasma* for most of the last century and there is superficial resemblance with species of that genus. Structures of the male terminalia, however, indicate that this species is clearly in the genus *Discocerina* where Zatwarnicki and Mathis (2001) placed the species. The high gena is distinctive among congeners in the Nearctic Region.

#### Discocerina ceraceps

(Cresson) comb. n.

http://species-id.net/wiki/Discocerina_ceraceps

Figs 200–203

Hecamedoides ceraceps Cresson, 1938: 27 [“Brazil”; HT ♂, ANSP (6534)].
Hydrochasma ceraceps . Cresson 1946: 141 [generic combination]. Wirth 1968: 8 [Neotropical catalog]. Mathis and Zatwarnicki 1995: 182 [world catalog].

##### Diagnosis.

**Figures 200–203.**
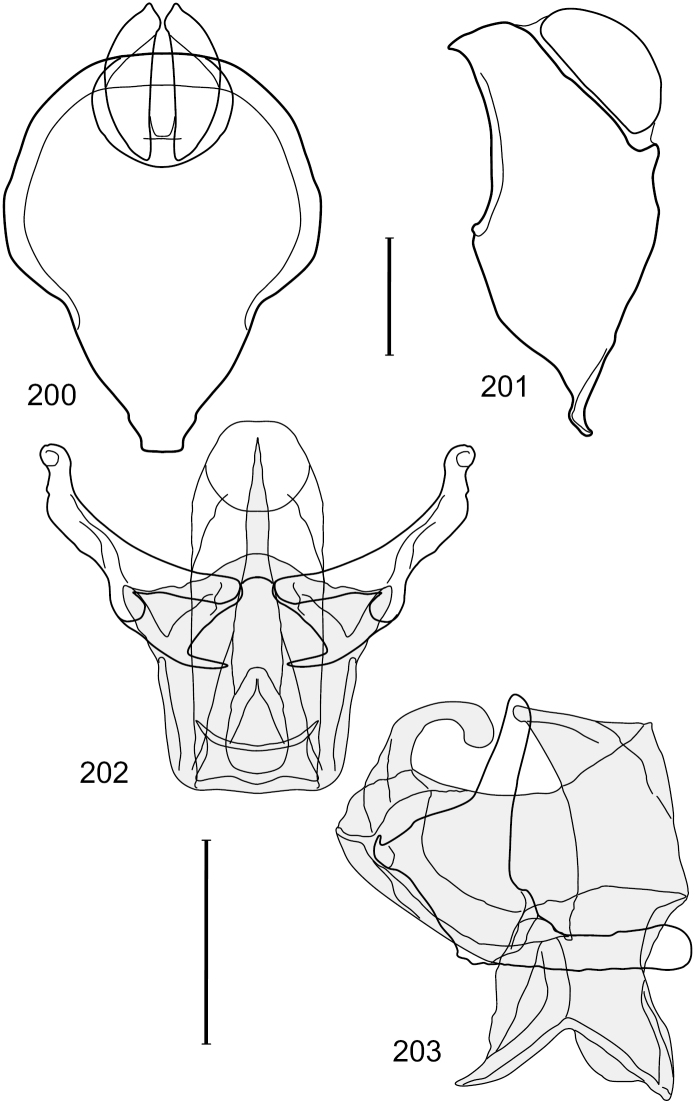
*Discocerina ceraceps* (Cresson) (Brazil, holotype) **200** outline of epandrium and cerci, posterior view **201** same, lateral view **202** internal structures of male terminalia (aedeagus [shaded], phallapodeme, gonite, hypandrium), ventral view **203** same, lateral view. Scale bar = 0.1 mm.

This species is distinguished from congeners by the following combination of characters: Generally densely microtomentose, whitish gray to blackish gray. Moderately small shore flies, body length 2.55 mm. *Head*: Frons mostly yellow, ocellar triangle gray; area immediately laterad of ocellar triangle brownish yellow; fronto-orbits whitish gray; pseudopostocellar setae well developed, length subequal to proclinate fronto-orbital seta. Antenna yellow; arista bearing 5–6 dorsally branching rays. Face mostly faintly yellow, becoming more whitish yellow ventrally and on dorsal portion of antennal grooves; bearing 3 larger setae in vertical row and with a small seta at ventral extent of row. Eye ratio 0.80. Gena high, mostly silvery white, gena-to-eye ratio 0.35. *Thorax*: Mesonotum mostly faintly grayish tan, becoming grayer laterally; pleural areas gray. Wing ratio 0.42; costal vein ratio 0.41; M vein ratio 0.54. Femora gray with extreme apex yellowish, medial surface of hindfemur shiny, reddish yellow; tibiae mostly yellowish with some sparse whitish to whitish gray microtomentum, especially hindtibia medially; tarsi yellow. *Abdomen*: Tergites 2–4 with wide medial stripe bronzish brown, gray laterally; tergite 5 mostly gray, truncate apically, bearing 4 larger apical setulae, length of larger setulae equal to width of tergite at apex. Male terminalia (Figs 200–203): Epandrium in posterior view (Fig. 200) more or less cordate, wide on dorsal half, tapered ventrally to a narrow, truncate ventral apex, dorsal margin broadly connected, width of dorsal connection in lateral view equal to width of a cercus, epandrium in lateral view (Fig. 201) becoming widest at midheight, thereafter tapered to point ventrally; cercus semi-hemispherical in posterior or lateral views (Fig. 200–201), height about twice width, overall height about 1/3 length of epandrium; phallapodeme and aedeagus fused, in ventral view (Fig. 202) rectangular, lateral margins parallel sided, apex truncate, base rounded, in lateral view (Fig. 203) as a fish tail apically; gonite in lateral view (Fig. 203) broadly bifurcate with dorsal and posterior prongs, both narrow, digitiform, ventral prong slightly longer and more robust, in ventral view (Fig. 202) rod-like, shallowly curved, with a midlength, short, bud-like prong.

##### Type material.

The holotype male of *Hecamedoides ceraceps* Cresson is labeled “551/TYPE Hecamedoides CERACEPS E.T.Cresson,Jr. [red; species and generic names handwritten].” The holotype is double mounted (minuten in a rectangular block of pith), is in good condition (some vertigis near exit and entrance of minuten; some mesonotal setae broken; abdomen removed and dissected with the parts in an attached microvial of glycerin), and is deposited in the ANSP (6534).

##### Type locality.

“BRAZIL.”

##### Other specimens examined.

URUGUAY. Montevideo: Montevideo (34°53'S, 56°11'W), 21–22 Jan 1927, F. and M. Edwards (1♀; USNM).

##### Distribution.

*Neotropical*: Brazil, Uruguay (Montevideo).

##### Remarks.

This species and *Discocerina buccata* are apparently closely related, as evidenced by the high gena, which is unique within *Discocerina* and which was also the reason why both species had been placed in *Hydrochasma*. Like *Discocerina buccata* and other congeners in *Discocerina*, however, *Discocerina ceraceps* has the phallapodeme fused with the base of the aedeagus, a synapomorphy for these species and other congeners in *Discocerina*.

## Supplementary Material

XML Treatment for Discocerinini

XML Treatment for Hydrochasma

XML Treatment for Hydrochasma castilloi

XML Treatment for Hydrochasma crenulum

XML Treatment for Hydrochasma digitatum

XML Treatment for Hydrochasma faciale

XML Treatment for Hydrochasma patens

XML Treatment for Hydrochasma rictum

XML Treatment for Hydrochasma sinuatum

XML Treatment for Hydrochasma spinosum

XML Treatment for Hydrochasma viridum

XML Treatment for Hydrochasma williamsae

XML Treatment for Hydrochasma denticum

XML Treatment for Hydrochasma distinctum

XML Treatment for Hydrochasma dolabrutum

XML Treatment for Hydrochasma falcatum

XML Treatment for Hydrochasma glochium

XML Treatment for Hydrochasma incisum

XML Treatment for Hydrochasma kaieteur

XML Treatment for Hydrochasma miguelito

XML Treatment for Hydrochasma octogonum

XML Treatment for Hydrochasma parallelum

XML Treatment for Hydrochasma peniculum

XML Treatment for Hydrochasma simplicum

XML Treatment for Hydrochasma urnulum

XML Treatment for Hydrochasma andeum

XML Treatment for Hydrochasma annae

XML Treatment for Hydrochasma aquia

XML Treatment for Hydrochasma avanae

XML Treatment for Hydrochasma capsum

XML Treatment for Hydrochasma edmistoni

XML Treatment for Hydrochasma garvinorum

XML Treatment for Hydrochasma leucoproctum

XML Treatment for Hydrochasma lineatum

XML Treatment for Hydrochasma robustum

XML Treatment for Hydrochasma sagittarium

XML Treatment for Discocerina buccata

XML Treatment for Discocerina ceraceps
